# Supplementary material for: Landscape-explicit phylogeography illuminates the ecographic radiation of early archosauromorph reptiles
Source: Nat Ecol Evol. 2025 Jun 11;9(7):1138–52. doi: 10.1038/s41559-025-02739-y (PMC12240803; doi:10.1038/s41559-025-02739-y)
Supplement: Supplementary file 1 — Supplementary Figs. 1–65 (ancestral origins, dispersal maps and dendrograms) and Tables 1–3. [file 41559_2025_2739_MOESM1_ESM.pdf]

nature ecology & evolution

Supplementary information https://doi.org/10.1038/s41559-025-02739-y

Landscape-explicit phylogeography illuminates the ecographic radiation of early archosauromorph reptiles

In the format provided by the authors and unedited

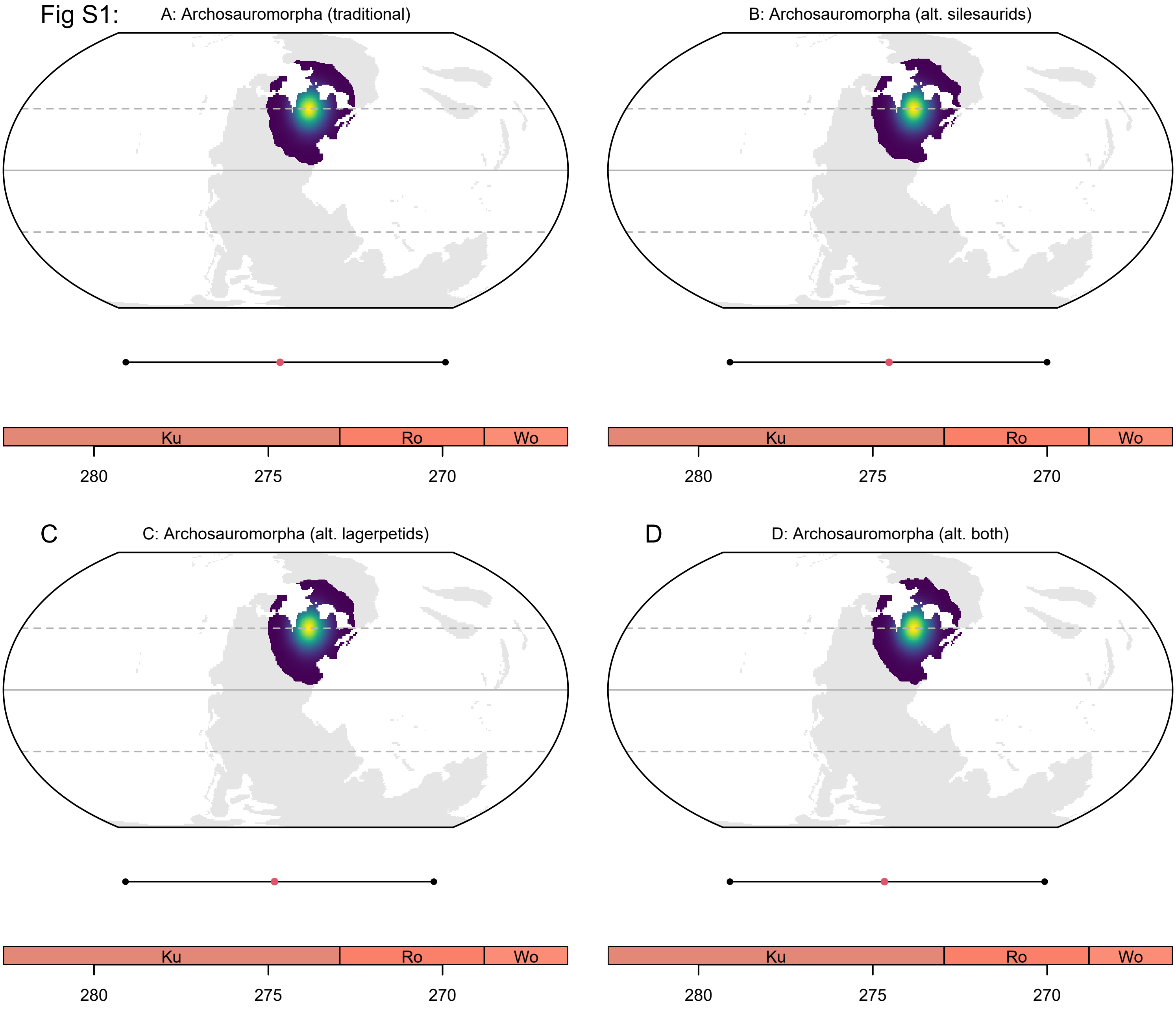
Fig S1:
A: Archosauromorpha (traditional)
B: Archosauromorpha (alt. silesaurids)
Ku
Ro
Wo
280
275
270
C
C: Archosauromorpha (alt. lagerpetids)
D
D: Archosauromorpha (alt. both)

Fig S2:

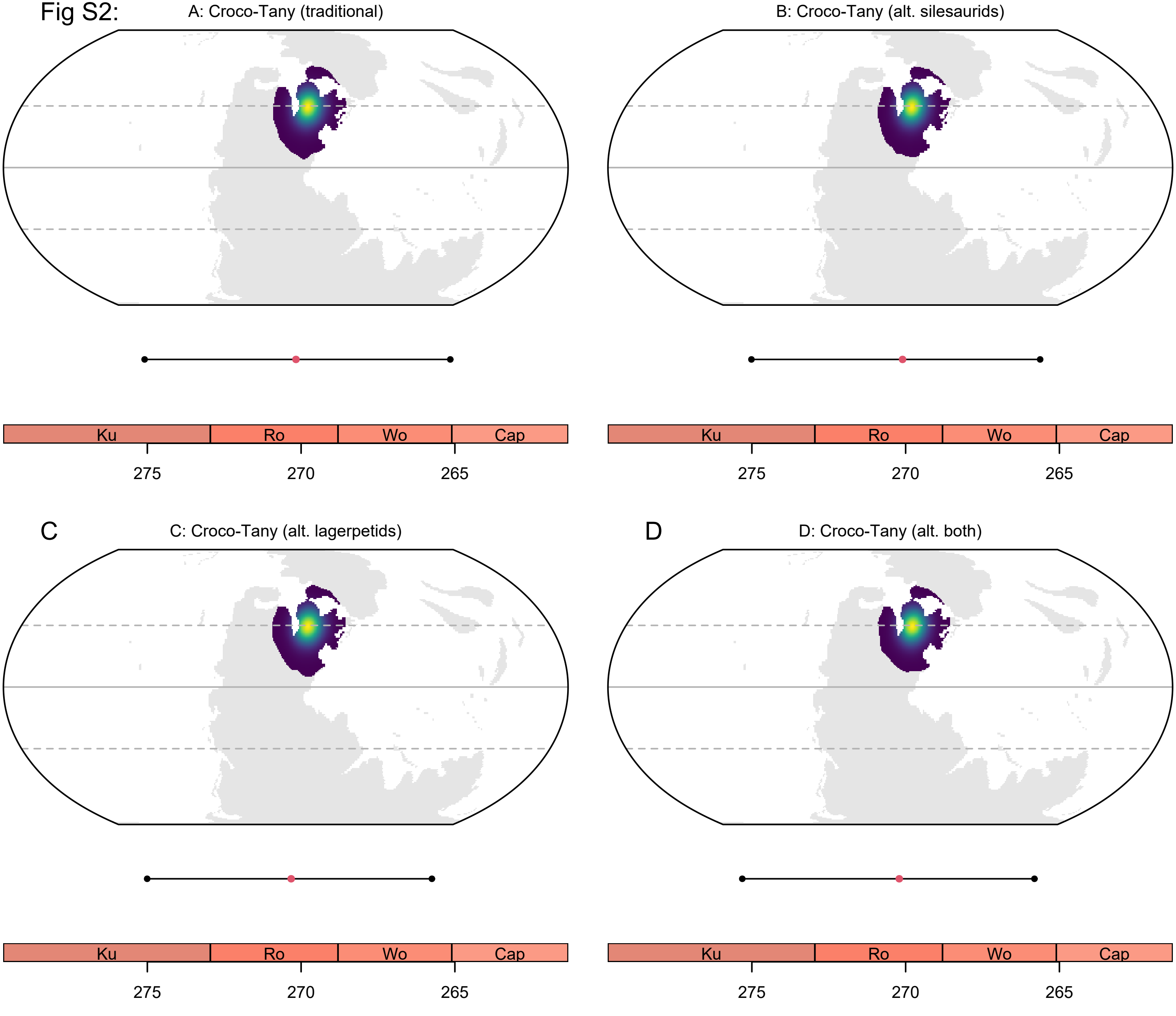

A: Croco-Tany (traditional)
B: Croco-Tany (alt. silesaurids)
Ku
Ro
Wo
Cap
275
270
265
Ku
Ro
Wo
Cap
275
270
265
C
C: Croco-Tany (alt. lagerpetids)
D
D: Croco-Tany (alt. both)
Ku
Ro
Wo
Cap
275
270
265
Ku
Ro
Wo
Cap
275
270
265

Fig S3:

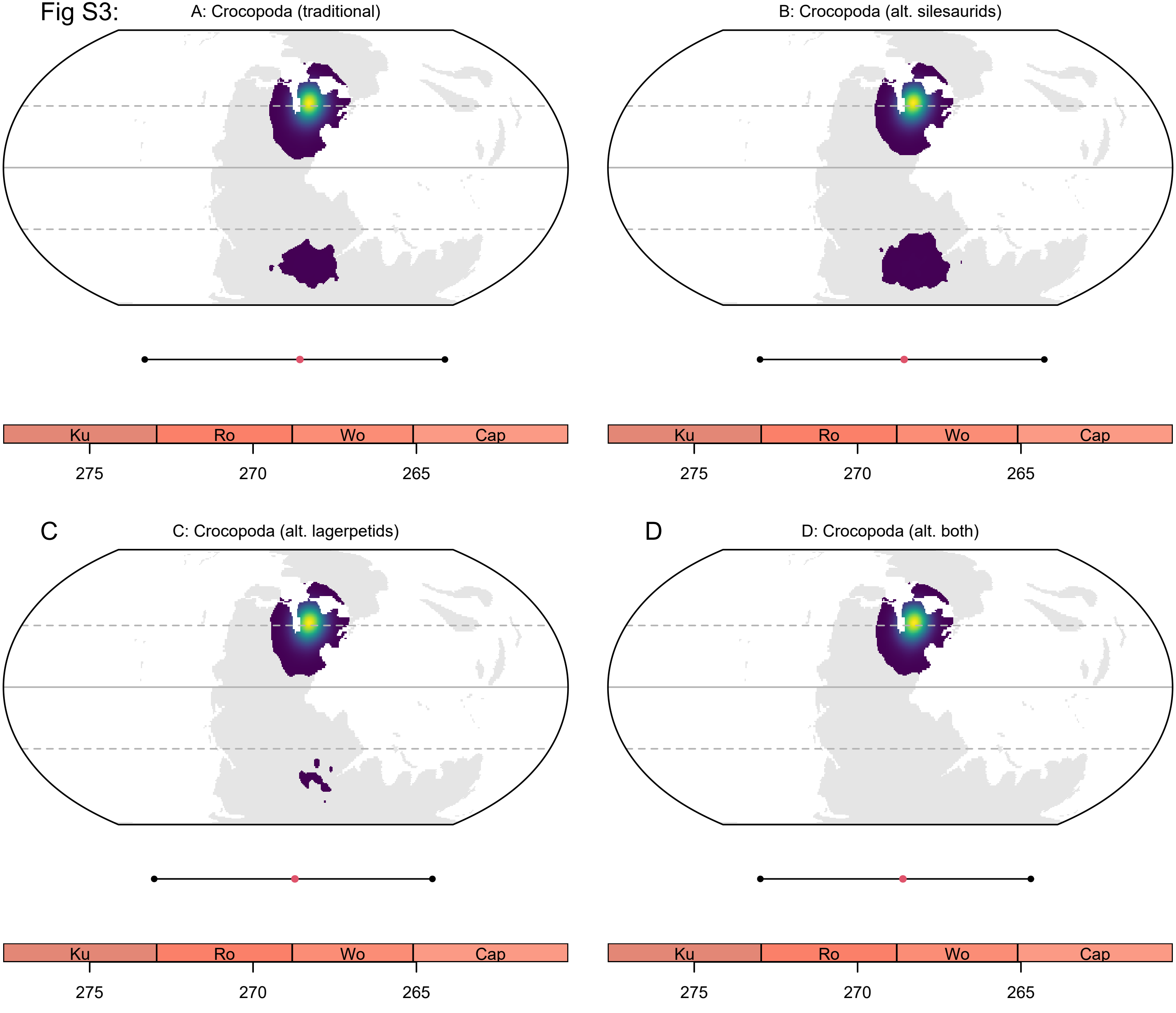
A: Crocopoda (traditional)
B: Crocopoda (alt. silesaurids)
Ku
Ro
Wo
Cap
275
270
265
C
C: Crocopoda (alt. lagerpetids)
D
D: Crocopoda (alt. both)

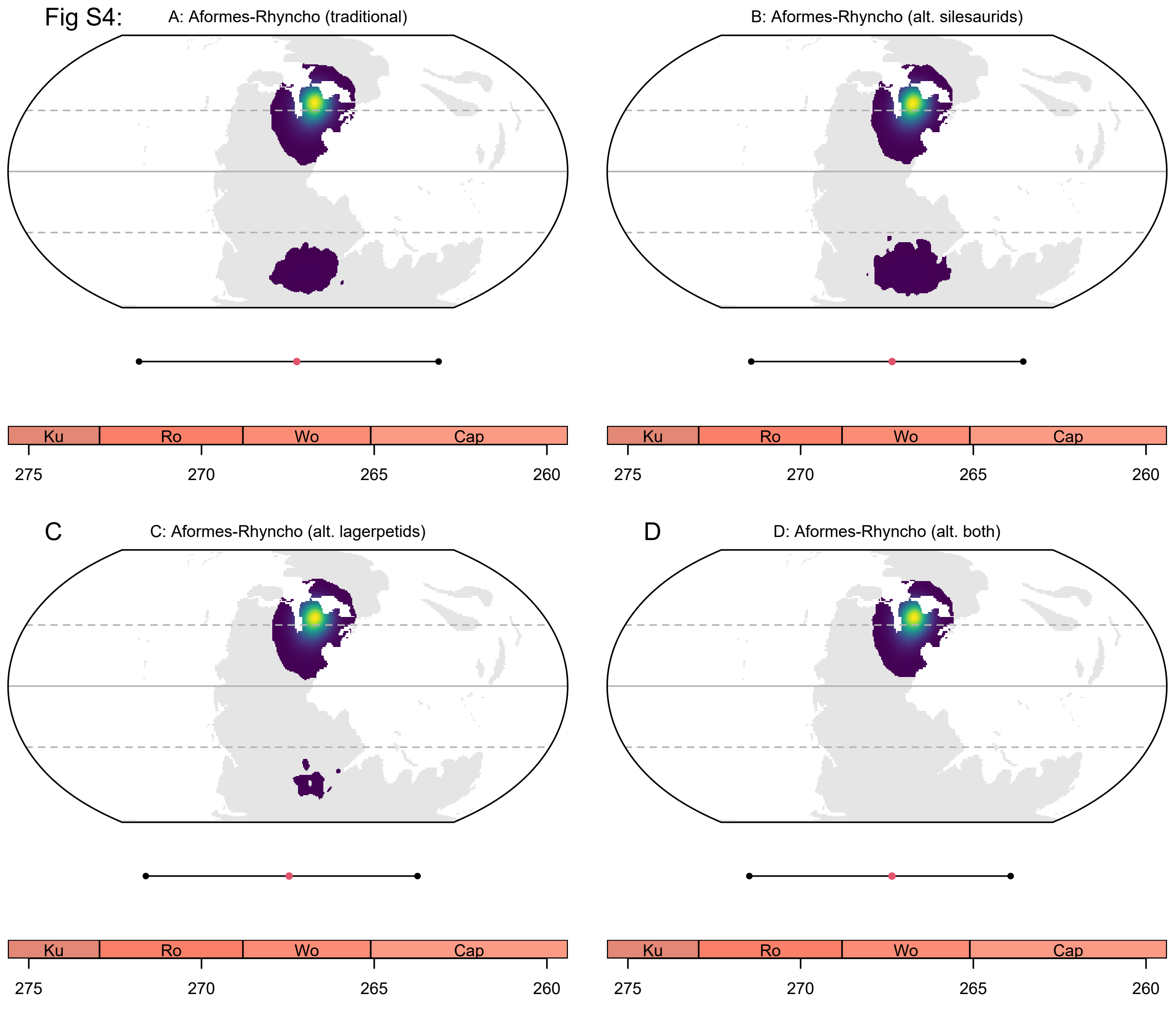
Fig S4:
A: Aformes-Rhyncho (traditional)
B: Aformes-Rhyncho (alt. silesaurids)
C
C: Aformes-Rhyncho (alt. lagerpetids)
D
D: Aformes-Rhyncho (alt. both)
Ku
Ro
Wo
Cap
275
270
265
260

Fig S5:

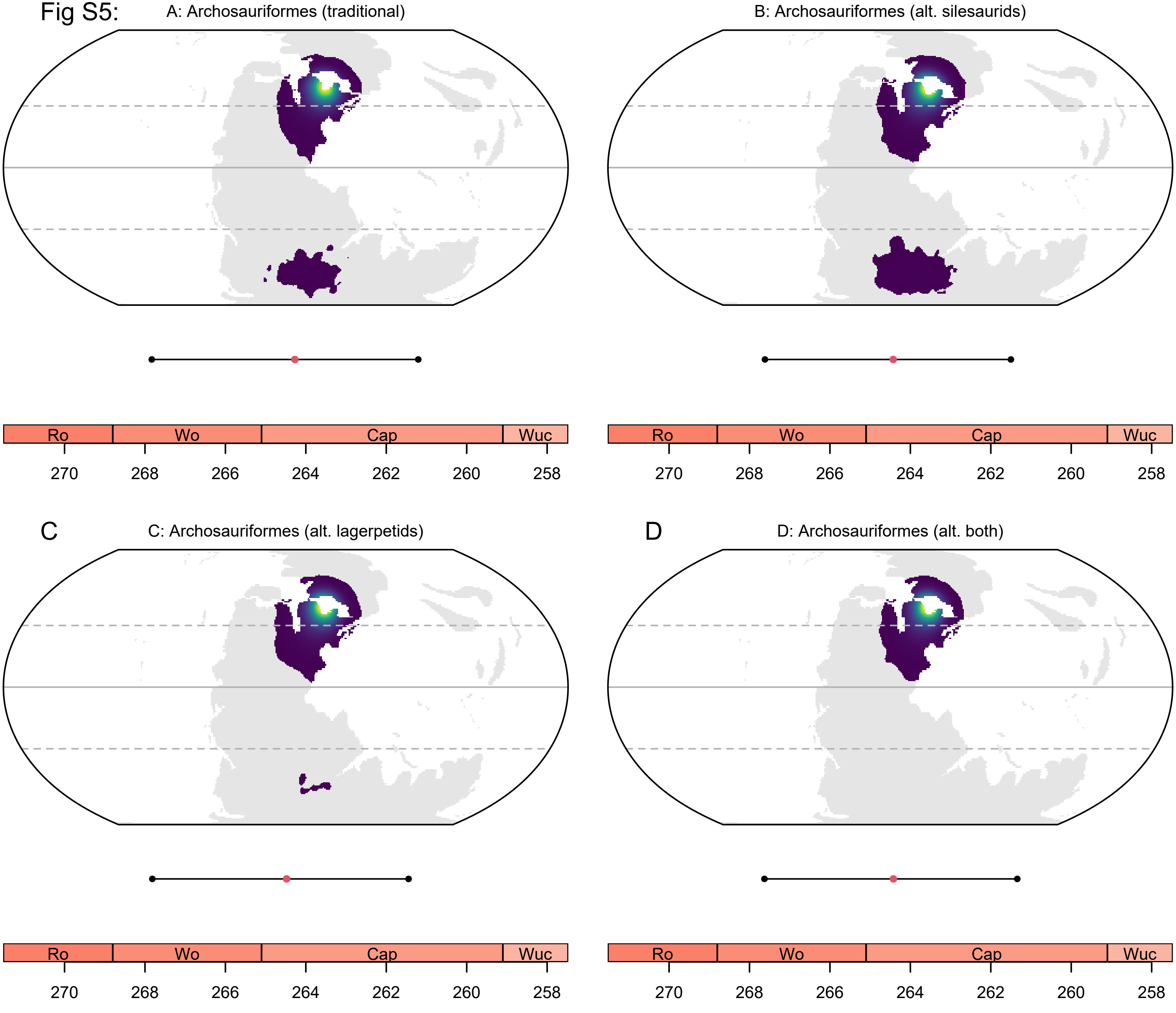
A: Archosauriformes (traditional)
B: Archosauriformes (alt. silesaurids)
C
C: Archosauriformes (alt. lagerpetids)
D
D: Archosauriformes (alt. both)
Ro
Wo
Cap
Wuc
270
268
266
264
262
260
258

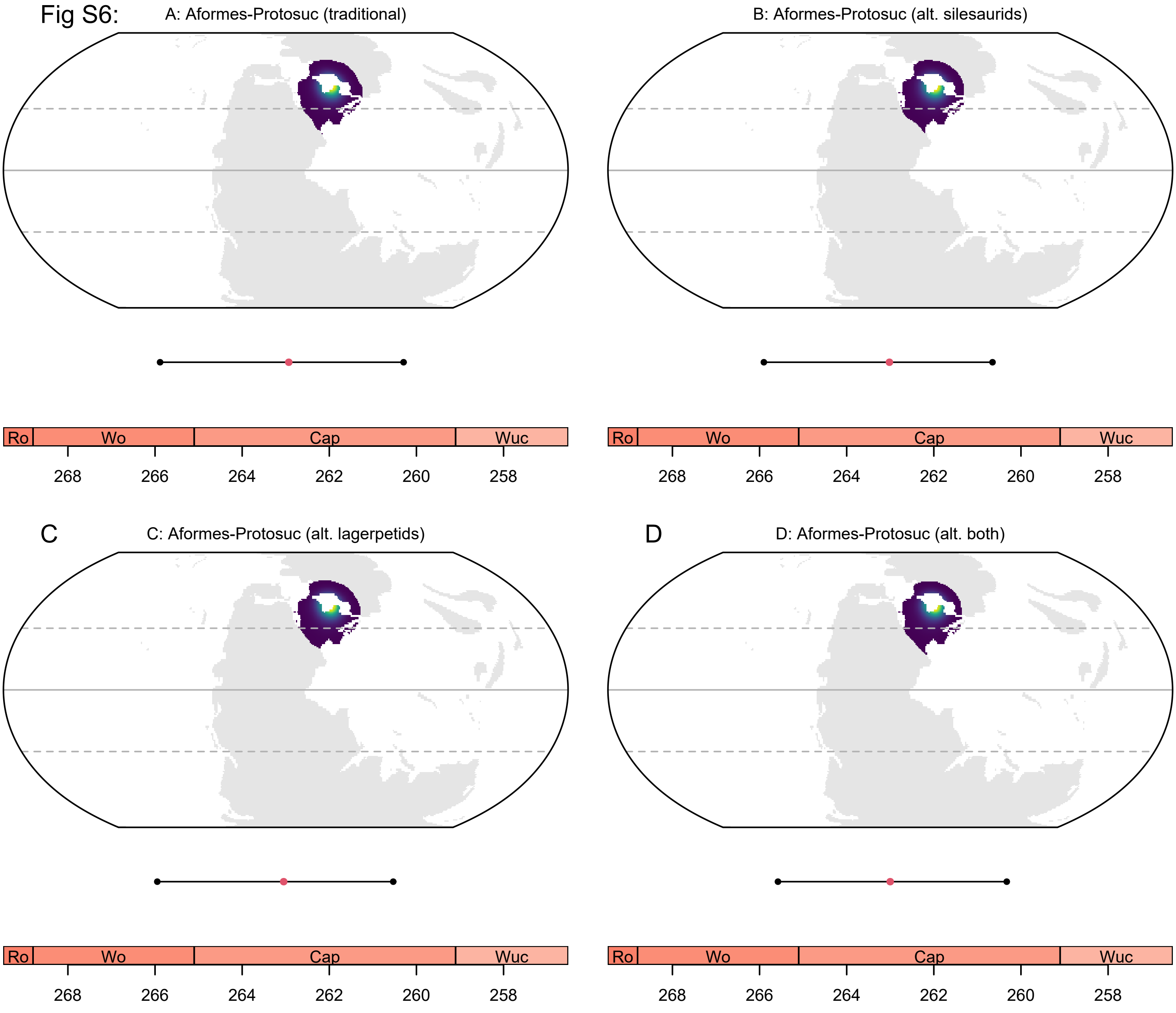
Fig S6:
A: Aformes-Protosuc (traditional)
Ro
Wo
Cap
Wuc
268
266
264
262
260
258
B: Aformes-Protosuc (alt. silesaurids)
Ro
Wo
Cap
Wuc
268
266
264
262
260
258
C
C: Aformes-Protosuc (alt. lagerpetids)
Ro
Wo
Cap
Wuc
268
266
264
262
260
258
D
D: Aformes-Protosuc (alt. both)
Ro
Wo
Cap
Wuc
268
266
264
262
260
258

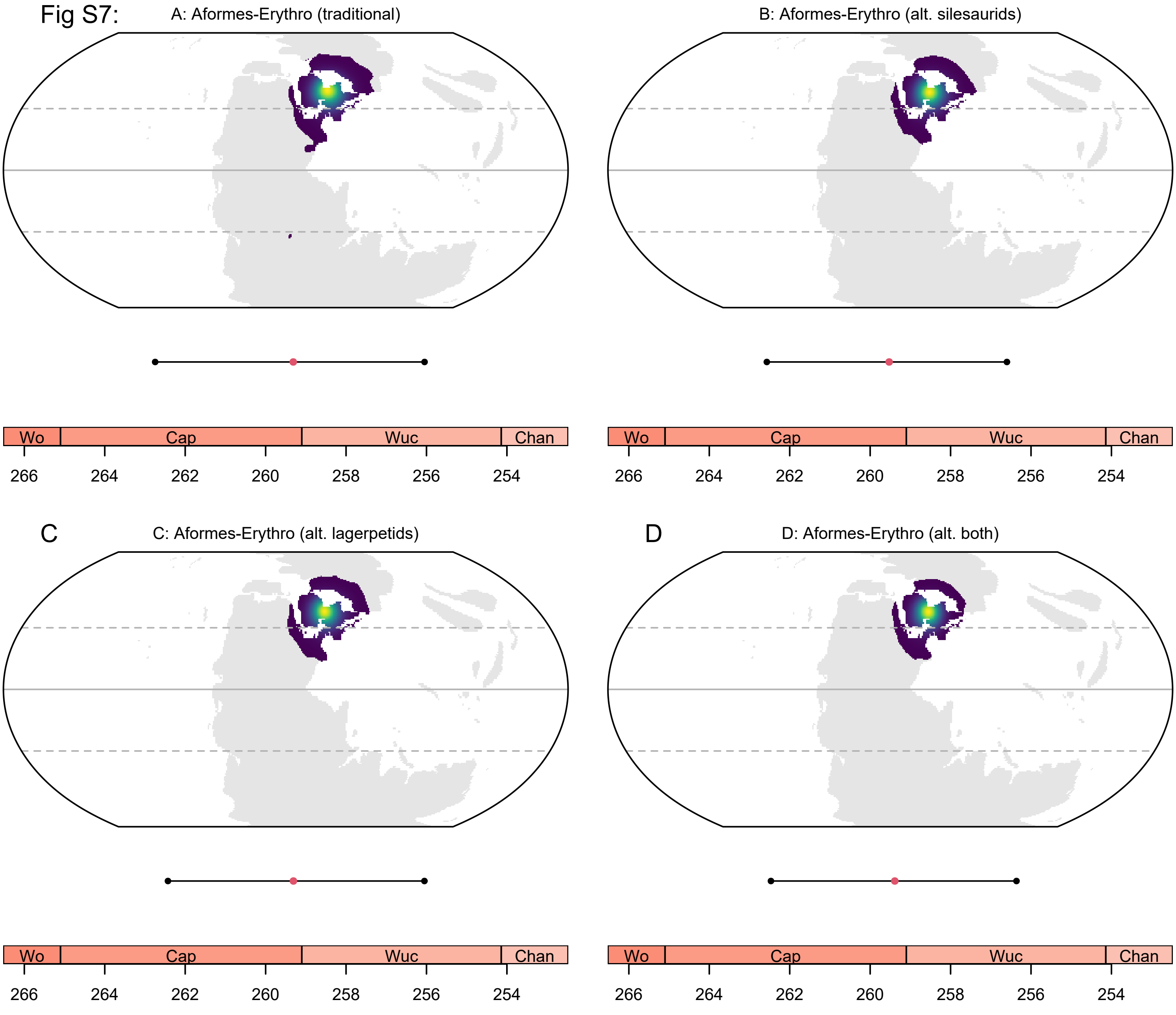
Fig S7:
A: Aformes-Erythro (traditional)
Wo
Cap
Wuc
Chan
266
264
262
260
258
256
254
B: Aformes-Erythro (alt. silesaurids)
Wo
Cap
Wuc
Chan
266
264
262
260
258
256
254
C
C: Aformes-Erythro (alt. lagerpetids)
Wo
Cap
Wuc
Chan
266
264
262
260
258
256
254
D
D: Aformes-Erythro (alt. both)
Wo
Cap
Wuc
Chan
266
264
262
260
258
256
254

Fig S8:

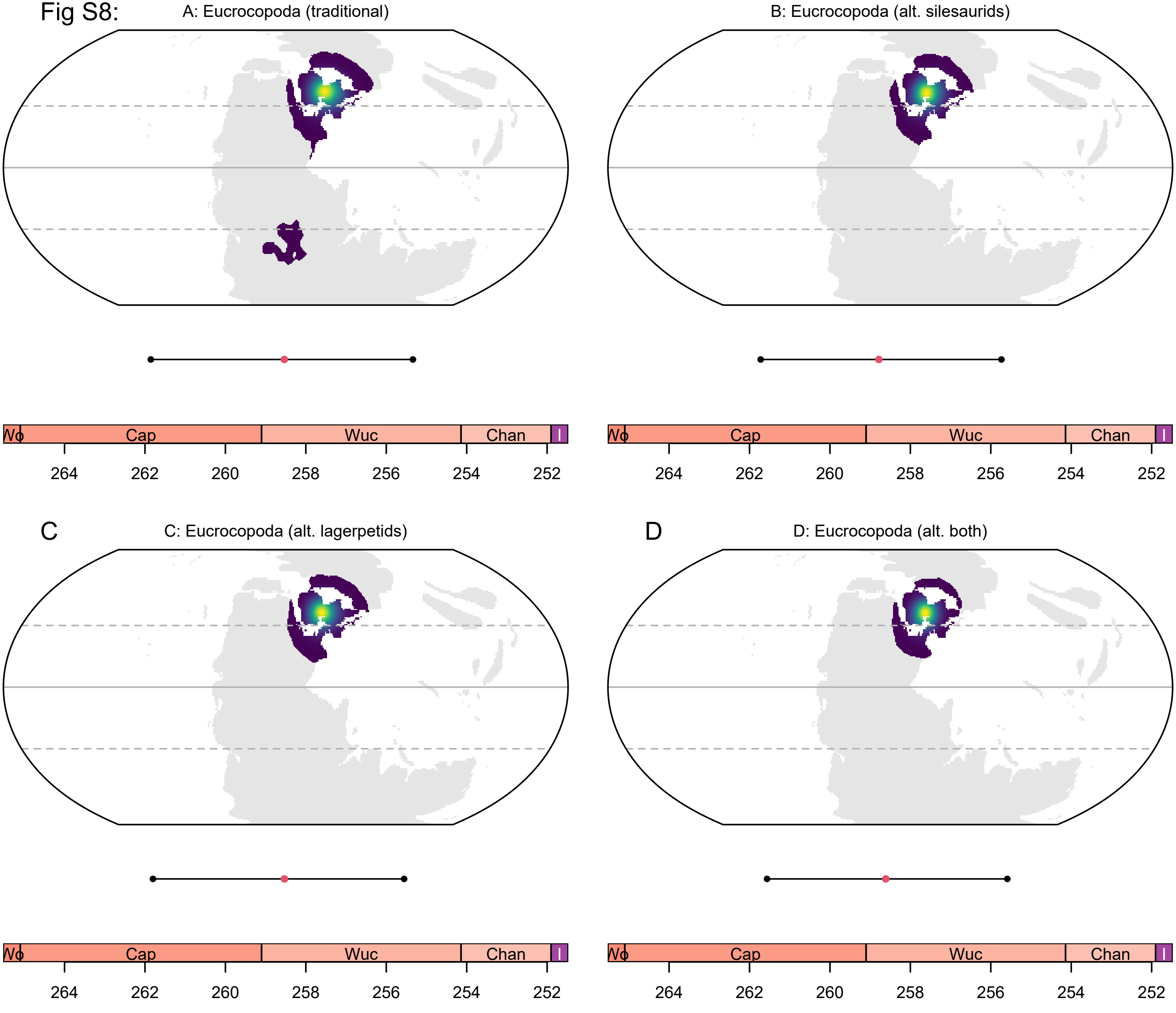
A: Eucrocopoda (traditional)
B: Eucrocopoda (alt. silesaurids)
C
C: Eucrocopoda (alt. lagerpetids)
D
D: Eucrocopoda (alt. both)
Wo
Cap
Wuc
Chan
I
264
262
260
258
256
254
252

Fig S9:

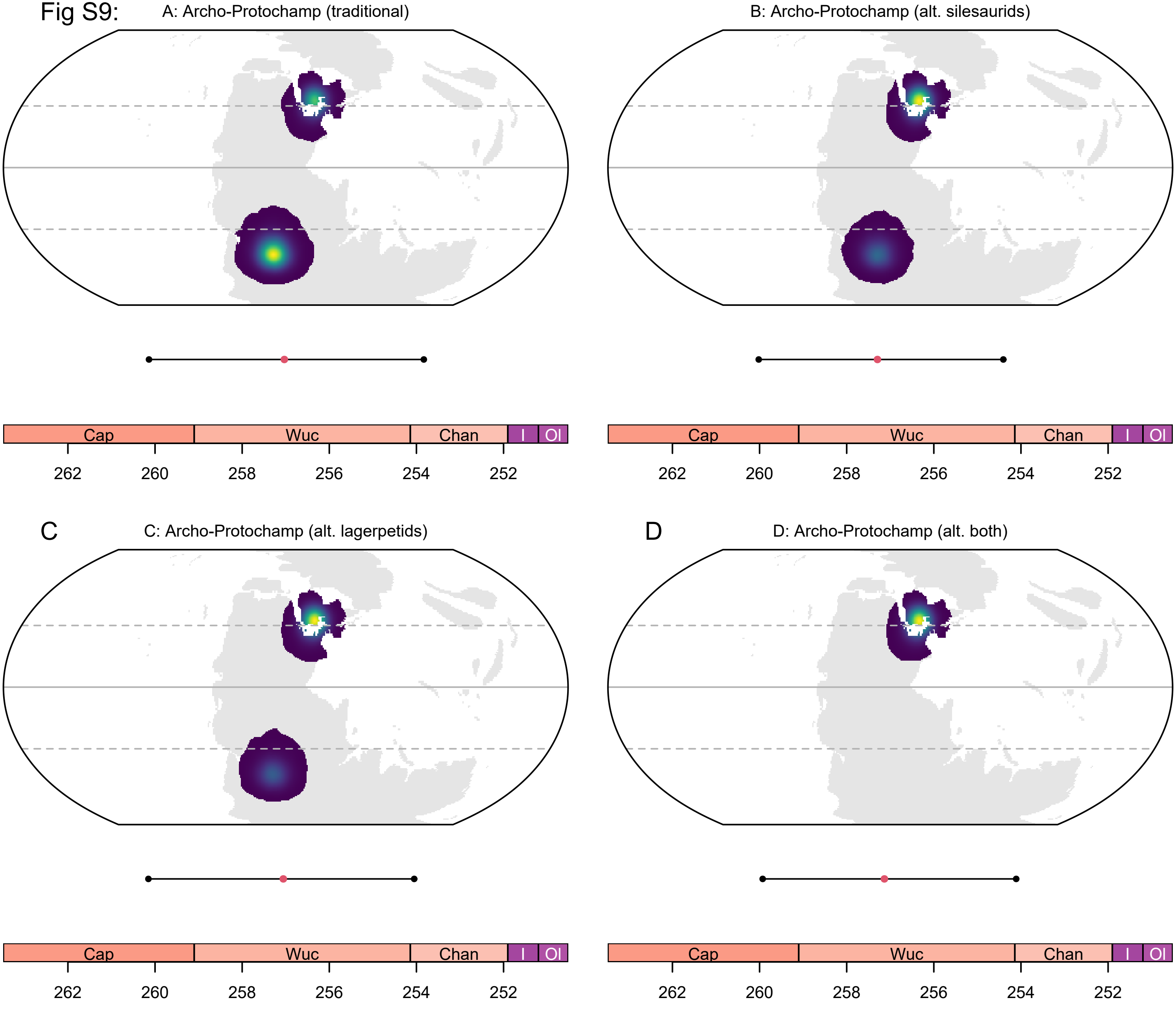

A: Archo-Protochamp (traditional)
B: Archo-Protochamp (alt. silesaurids)
C
C: Archo-Protochamp (alt. lagerpetids)
D
D: Archo-Protochamp (alt. both)
Cap
Wuc
Chan
I
Ol
262
260
258
256
254
252

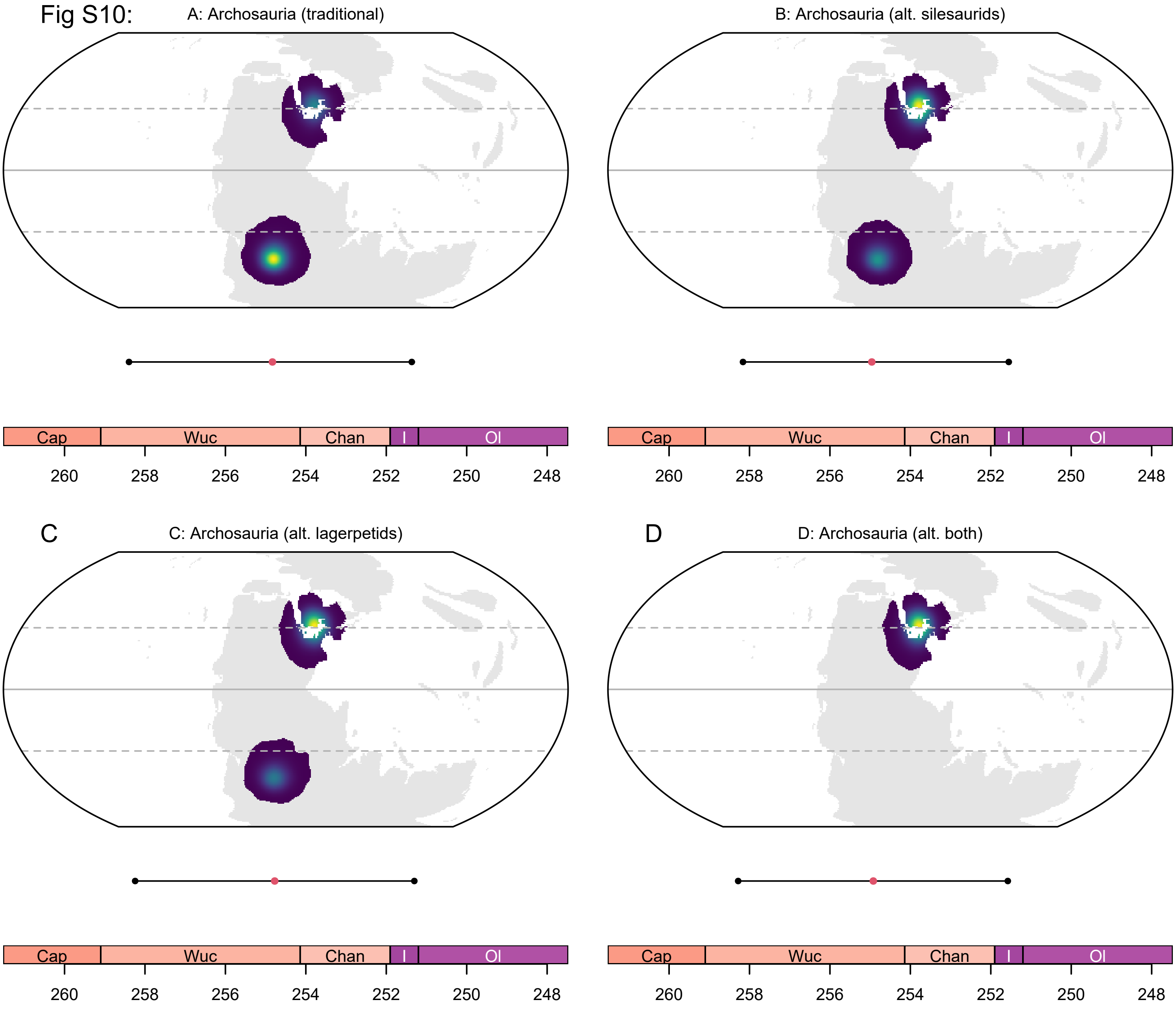
Fig S10:
A: Archosauria (traditional)
B: Archosauria (alt. silesaurids)
C
C: Archosauria (alt. lagerpetids)
D
D: Archosauria (alt. both)
Cap
Wuc
Chan
I
Ol
260
258
256
254
252
250
248

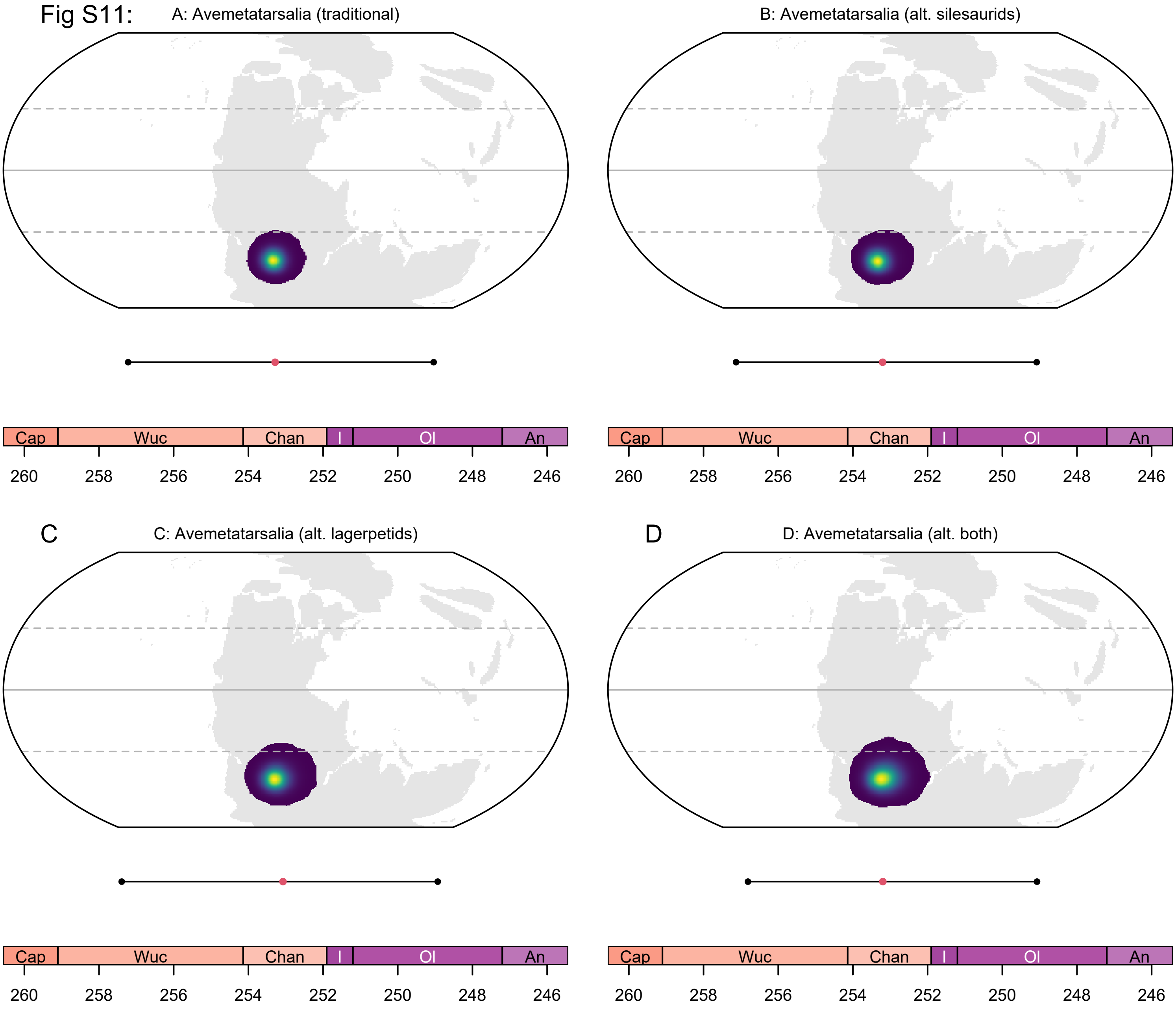
Fig S11:
A: Avemetatarsalia (traditional)
B: Avemetatarsalia (alt. silesaurids)
C
C: Avemetatarsalia (alt. lagerpetids)
D
D: Avemetatarsalia (alt. both)
Cap
Wuc
Chan
I
Ol
An
260
258
256
254
252
250
248
246

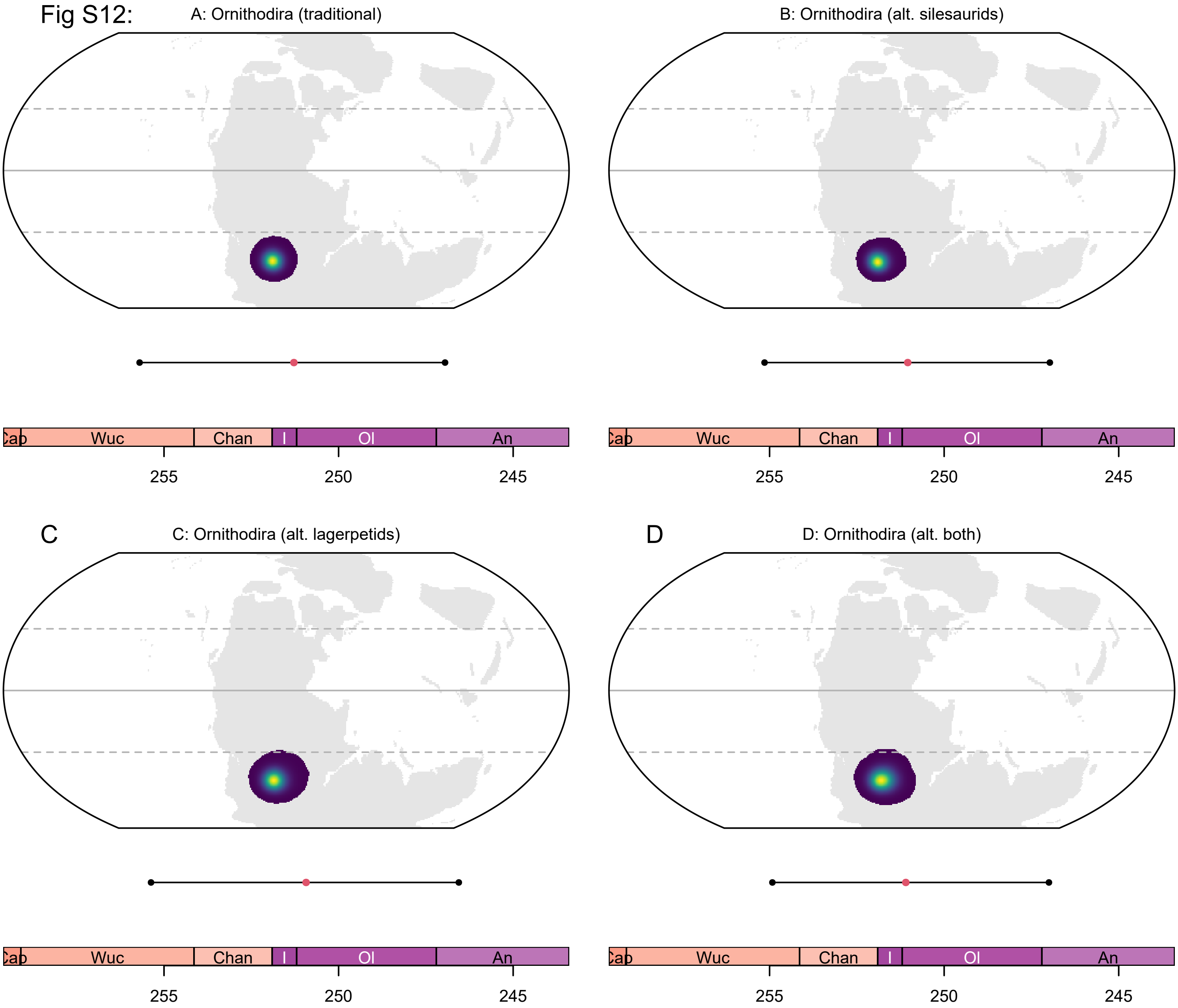
Fig S12:
A: Ornithodira (traditional)
B: Ornithodira (alt. silesaurids)
C
C: Ornithodira (alt. lagerpetids)
D
D: Ornithodira (alt. both)
Cap
Wuc
Chan
I
Ol
An
255
250
245

Fig S13:

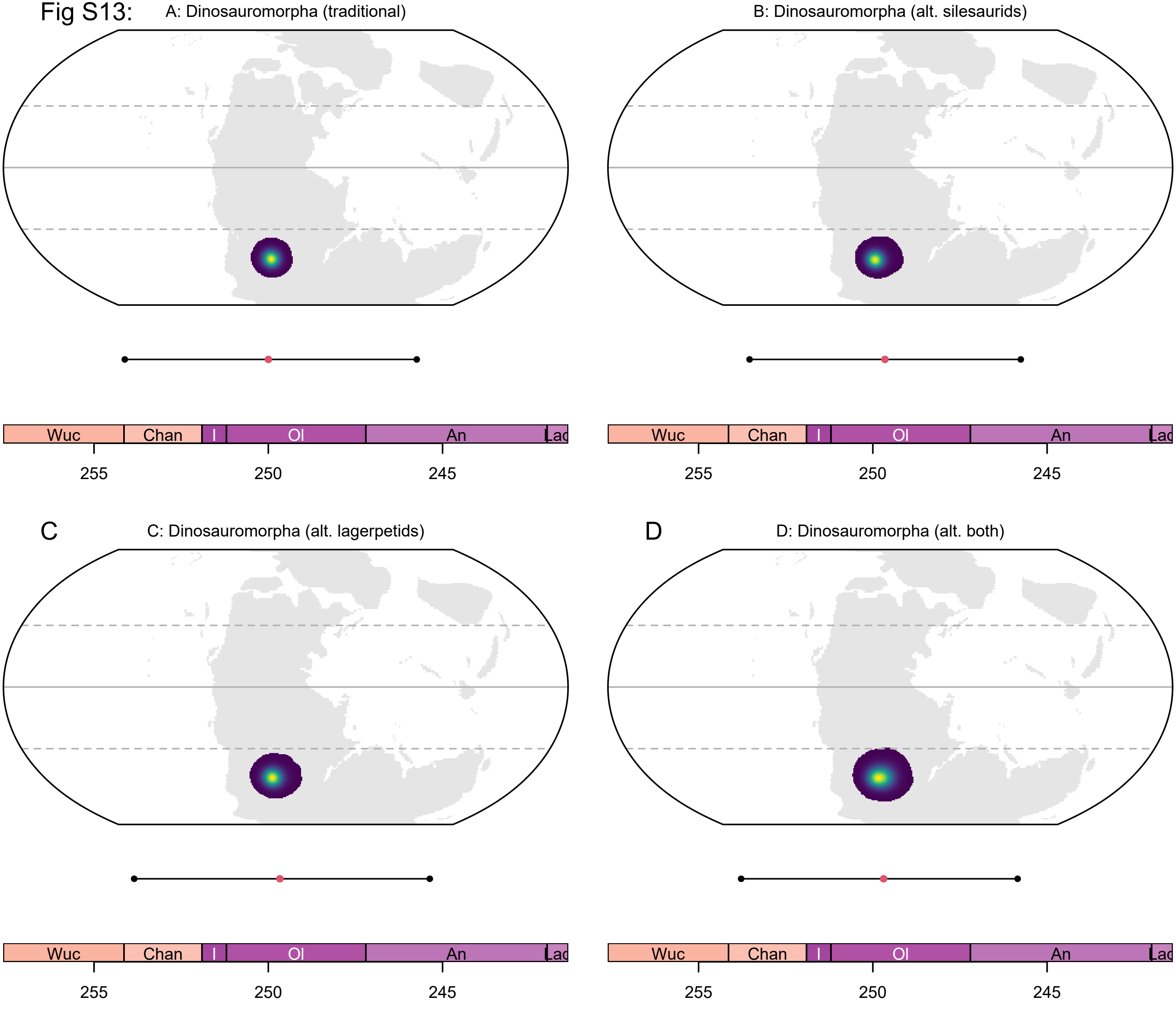
A: Dinosauromorpha (traditional)
B: Dinosauromorpha (alt. silesaurids)
C
C: Dinosauromorpha (alt. lagerpetids)
D
D: Dinosauromorpha (alt. both)
Wuc
Chan
I
Ol
An
Lad
255
250
245

Fig S14:

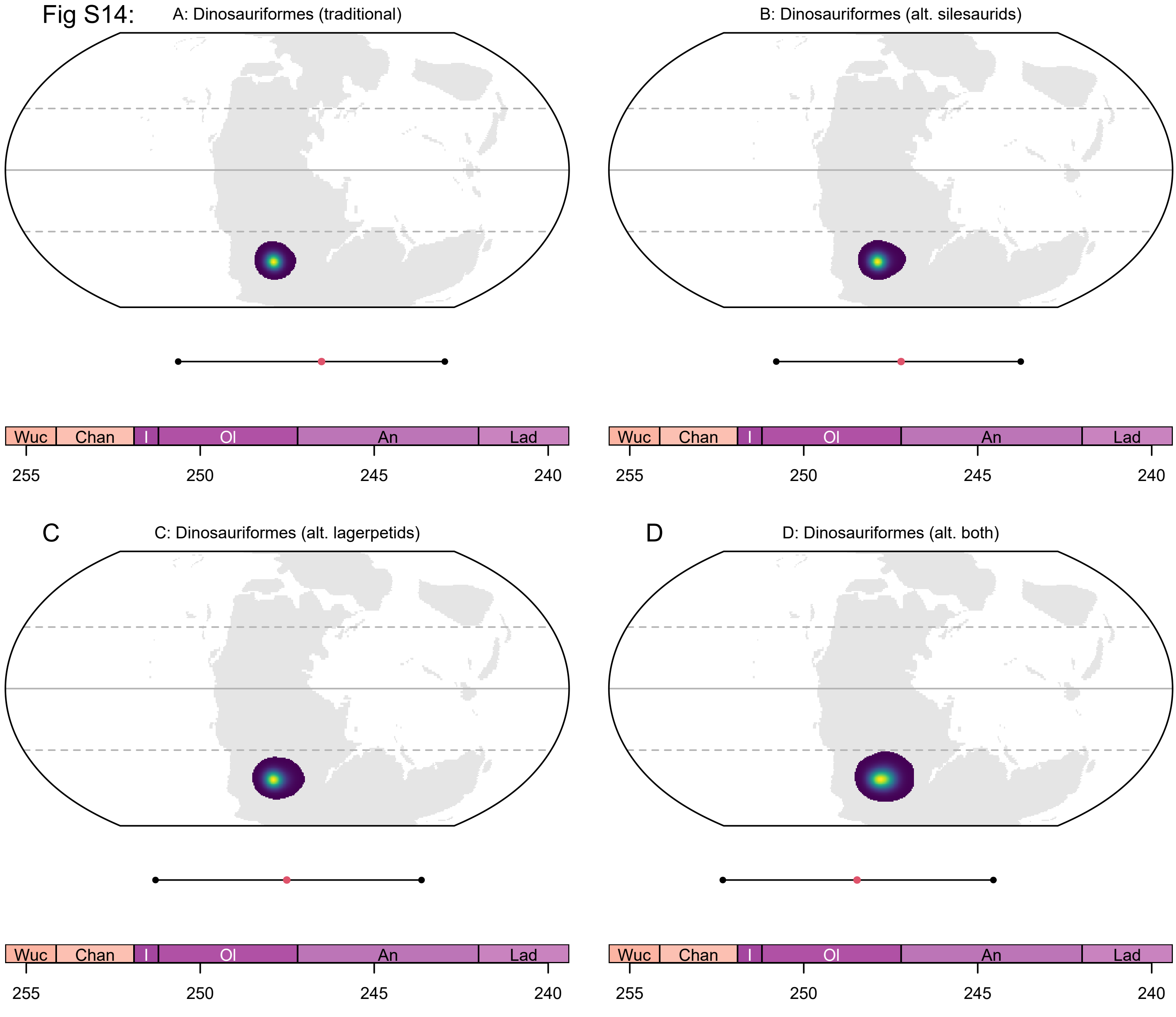
A: Dinosauriformes (traditional)
B: Dinosauriformes (alt. silesaurids)
C
C: Dinosauriformes (alt. lagerpetids)
D
D: Dinosauriformes (alt. both)
Wuc
Chan
I
Ol
An
Lad
255
250
245
240

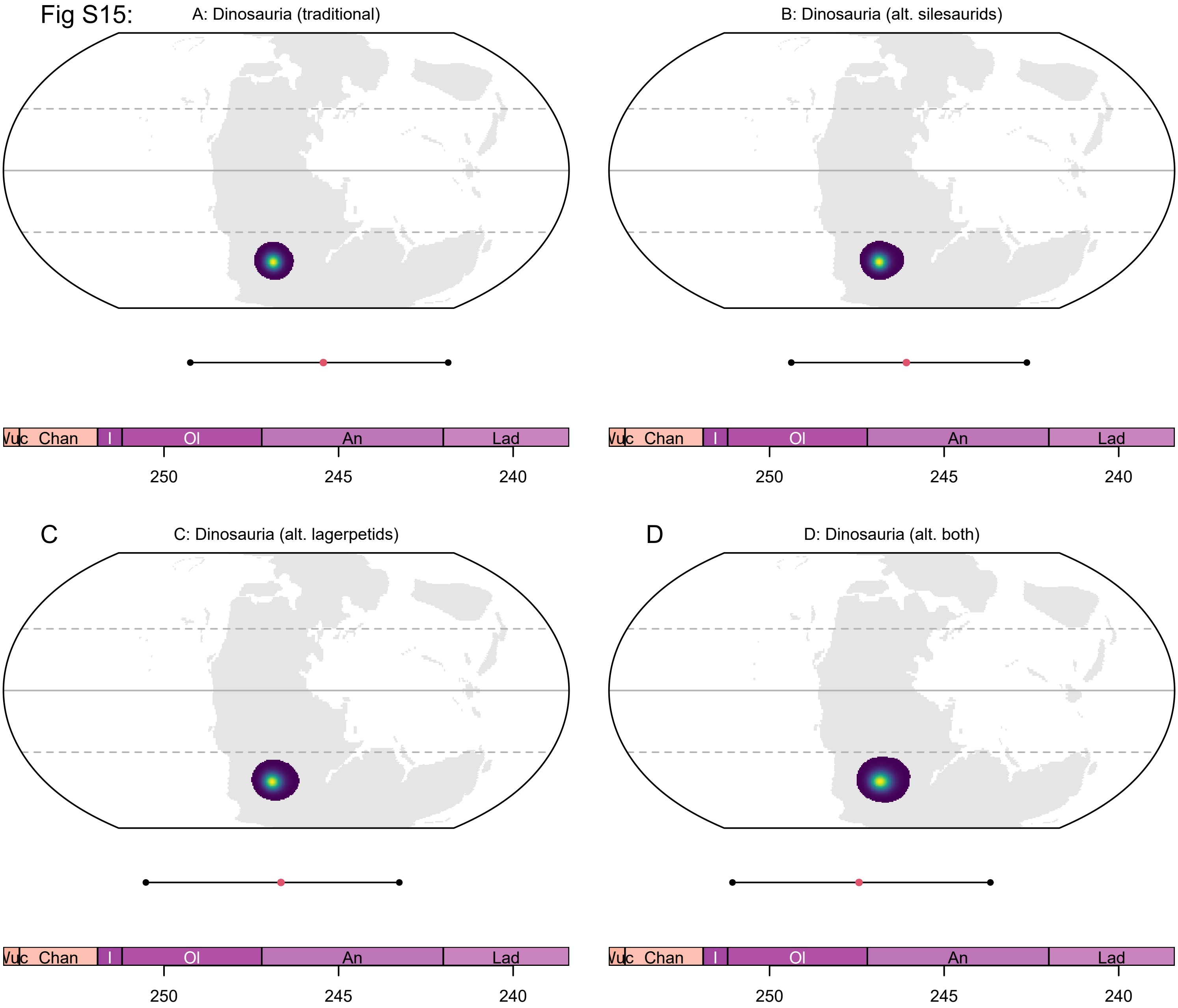
Fig S15:
A: Dinosauria (traditional)
B: Dinosauria (alt. silesaurids)
C
C: Dinosauria (alt. lagerpetids)
D
D: Dinosauria (alt. both)
Wuc Chan I Ol An Lad
250 245 240

Fig S16:

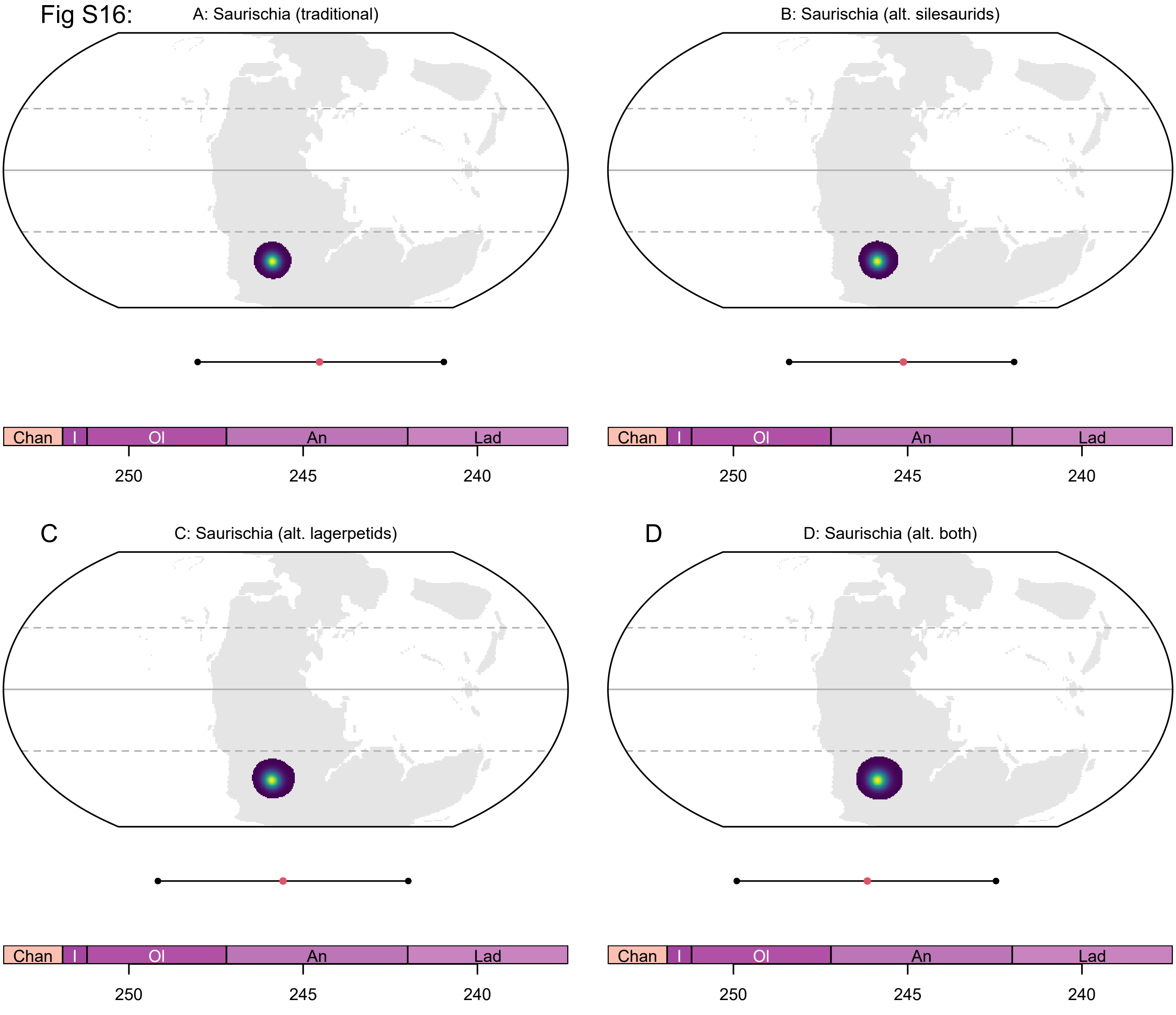
A: Saurischia (traditional)
B: Saurischia (alt. silesaurids)
Chan
I
Ol
An
Lad
250
245
240
C
C: Saurischia (alt. lagerpetids)
D
D: Saurischia (alt. both)
Chan
I
Ol
An
Lad
250
245
240

Fig S17:

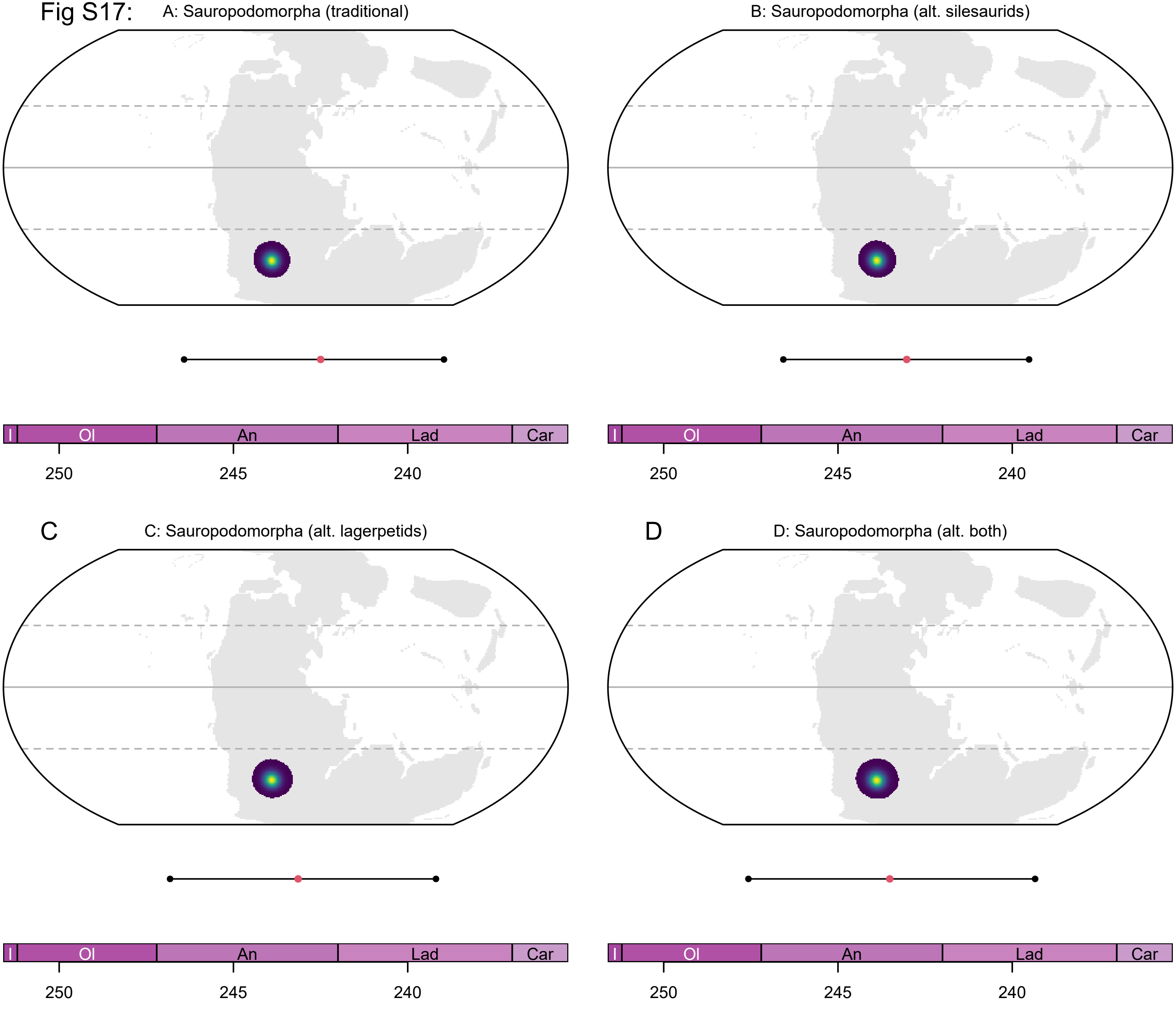

A: Sauropodomorpha (traditional)
B: Sauropodomorpha (alt. silesaurids)
I
Ol
An
Lad
Car
250
245
240
C
C: Sauropodomorpha (alt. lagerpetids)
D
D: Sauropodomorpha (alt. both)

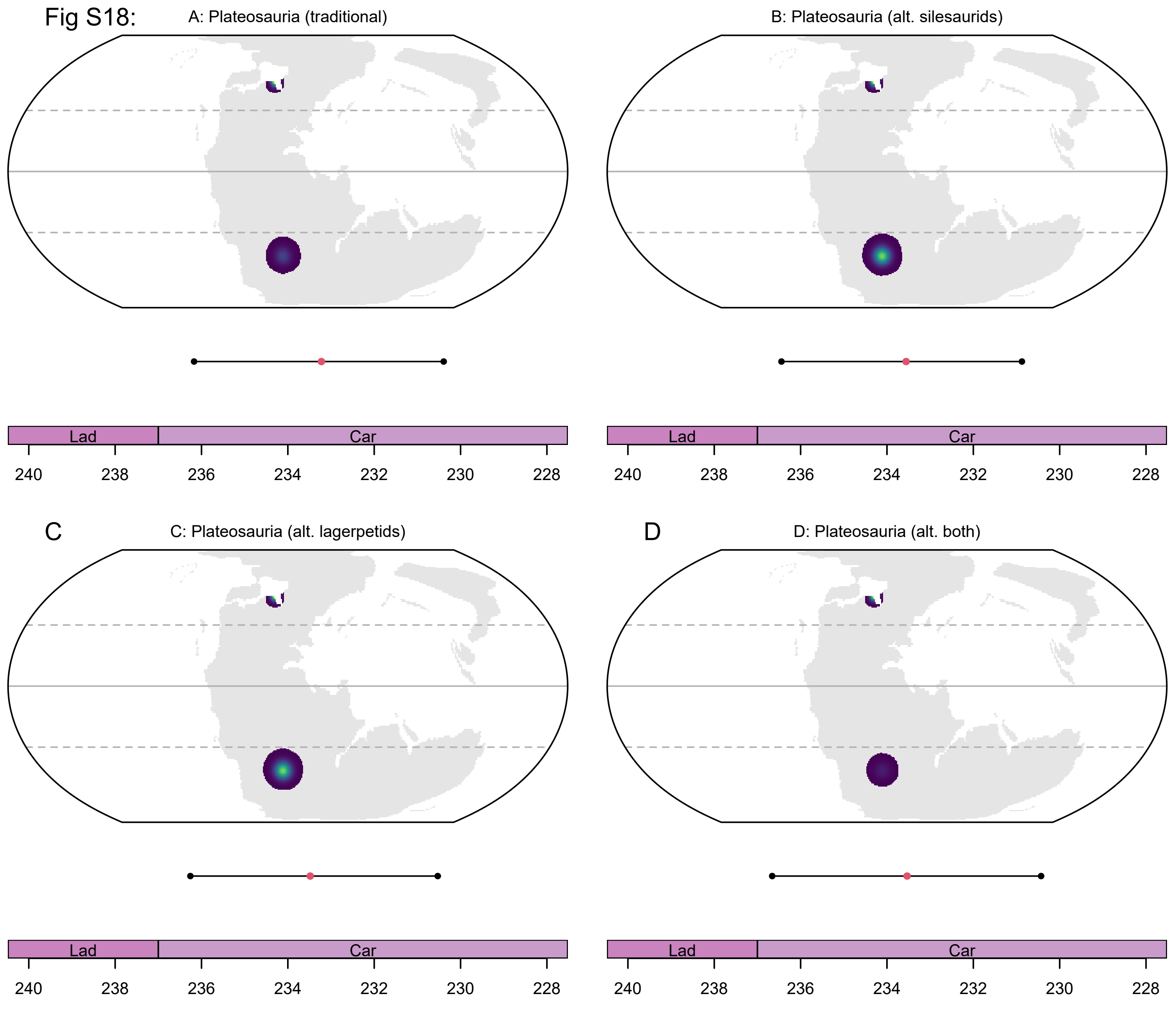
Fig S18:
A: Plateosauria (traditional)
B: Plateosauria (alt. silesaurids)
C
C: Plateosauria (alt. lagerpetids)
D
D: Plateosauria (alt. both)
Lad
Car
240
238
236
234
232
230
228

Fig S19:

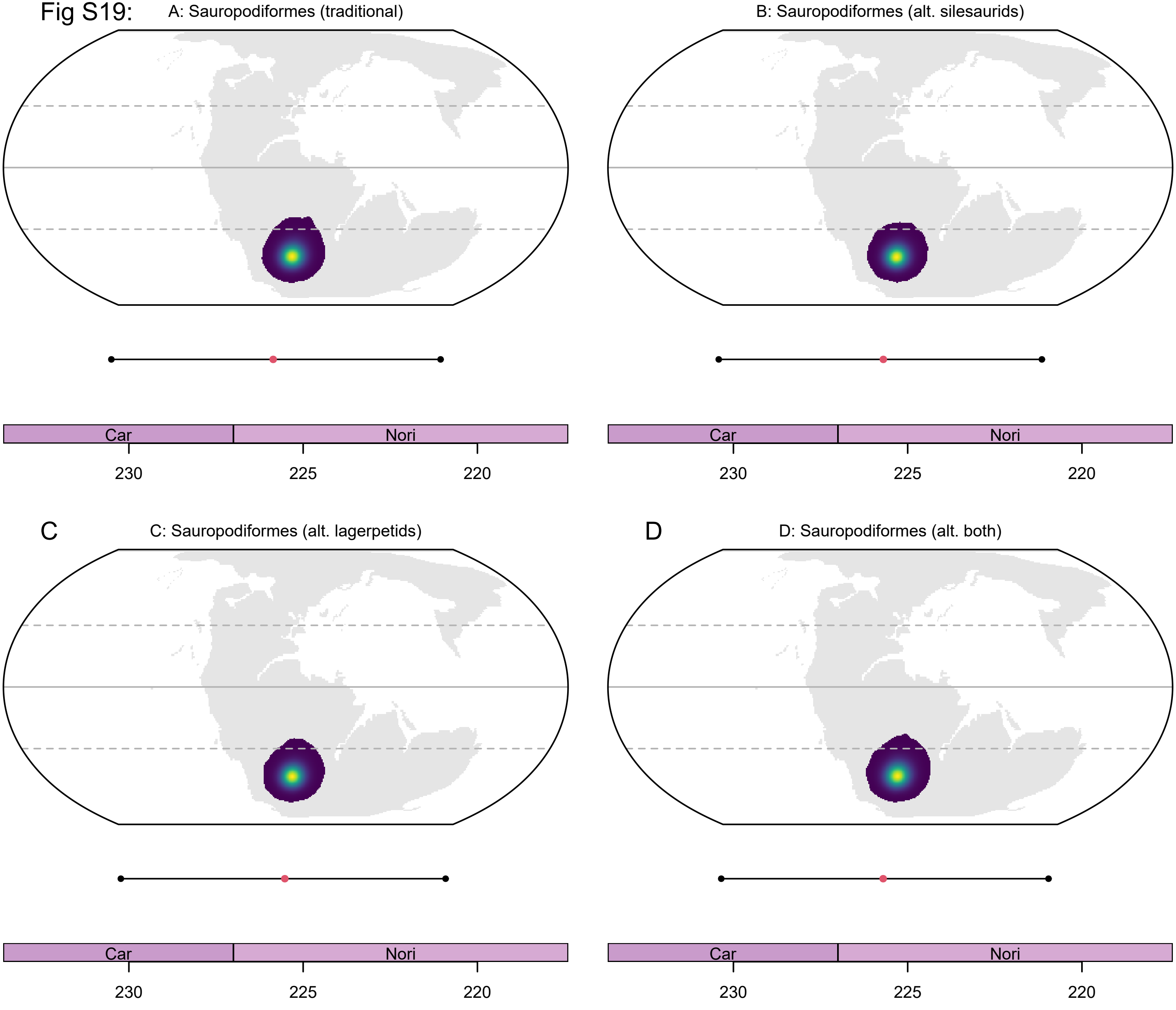
A: Sauropodiformes (traditional)
Car
Nori
230
225
220
B: Sauropodiformes (alt. silesaurids)
Car
Nori
230
225
220
C
C: Sauropodiformes (alt. lagerpetids)
Car
Nori
230
225
220
D
D: Sauropodiformes (alt. both)
Car
Nori
230
225
220

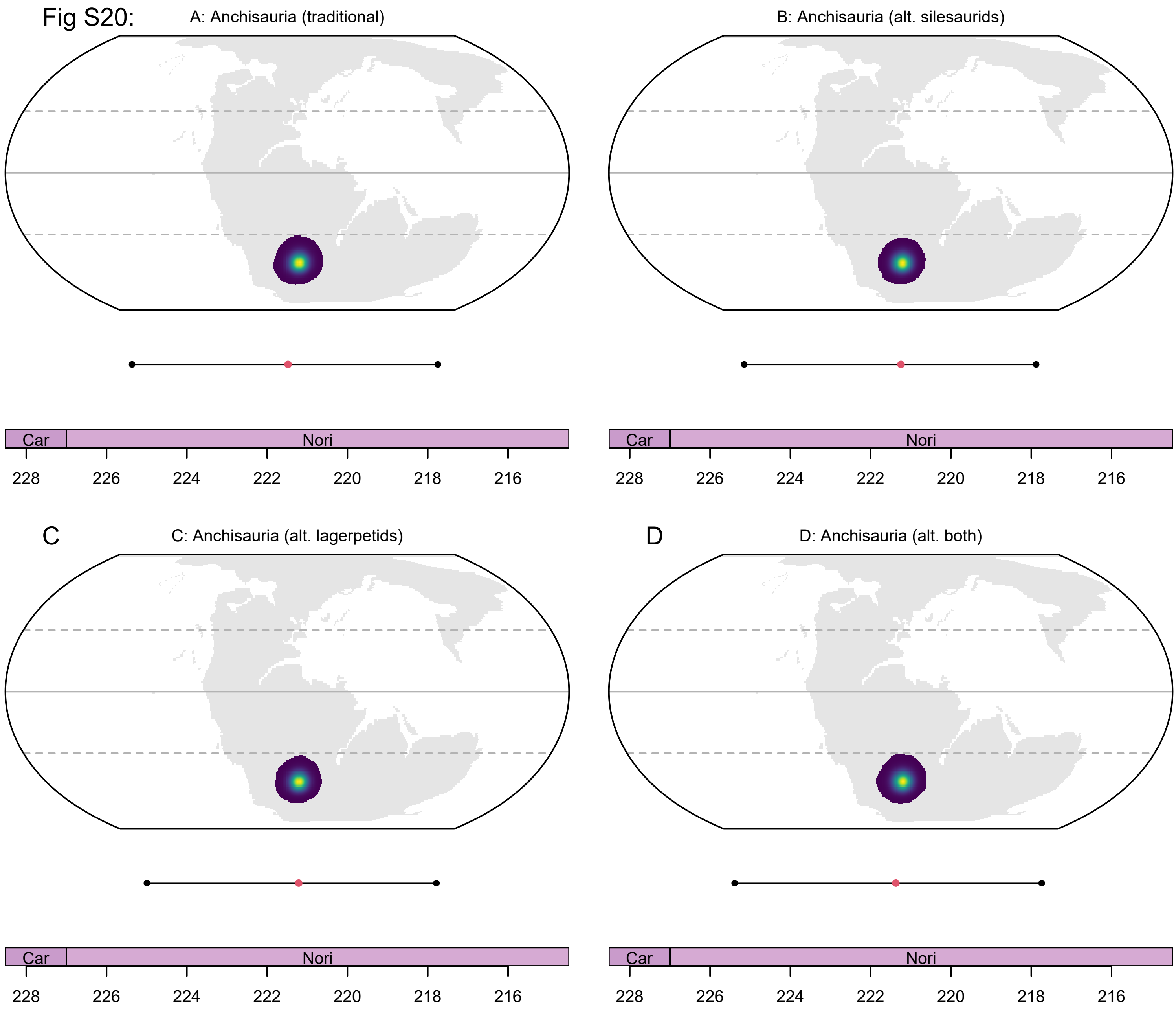
Fig S20:
A: Anchisauria (traditional)
B: Anchisauria (alt. silesaurids)
C
C: Anchisauria (alt. lagerpetids)
D
D: Anchisauria (alt. both)
Car
Nori
228
226
224
222
220
218
216

Fig S21:

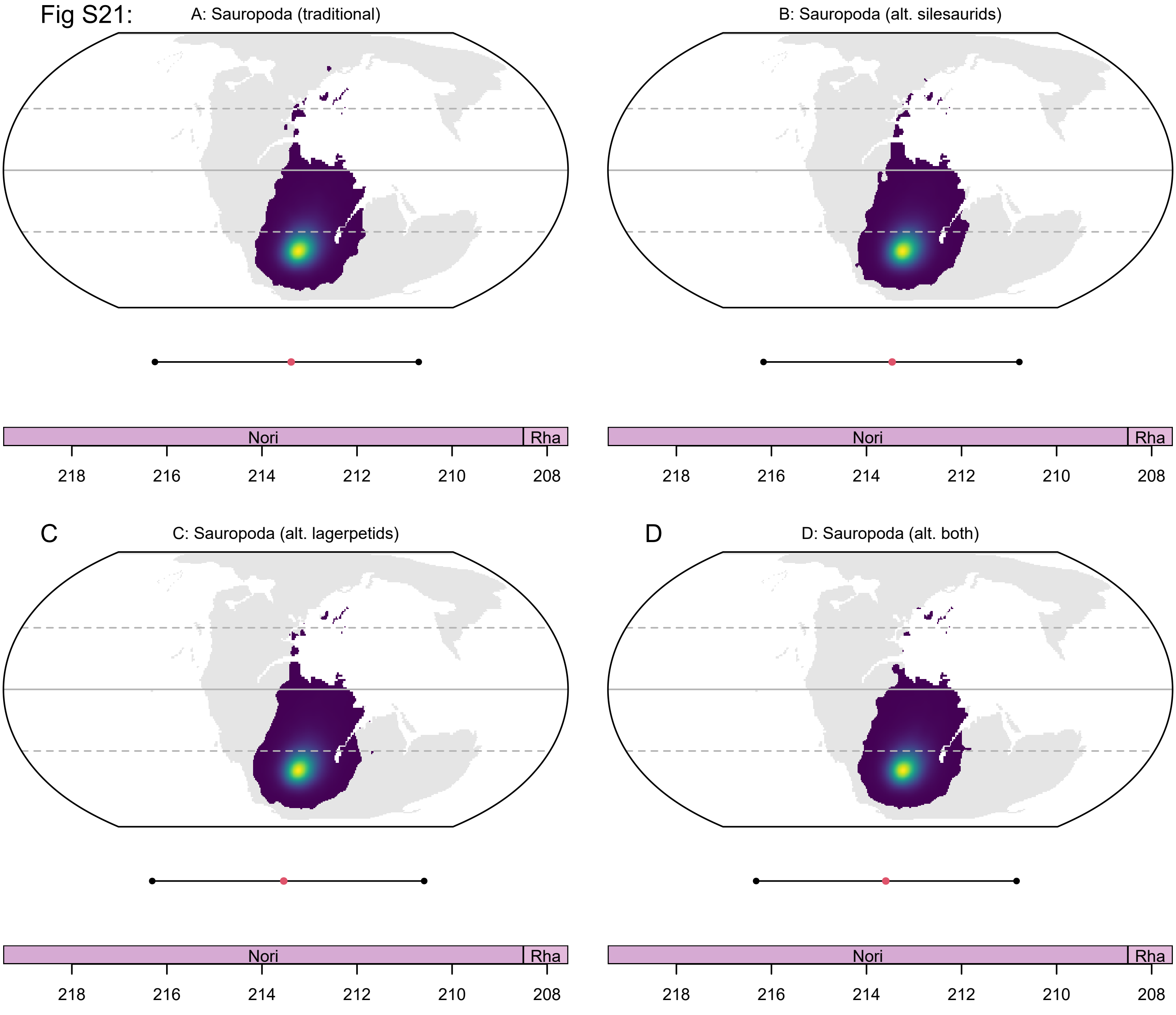
A: Sauropoda (traditional)
B: Sauropoda (alt. silesaurids)
C
C: Sauropoda (alt. lagerpetids)
D
D: Sauropoda (alt. both)
Nori
Rha
218
216
214
212
210
208

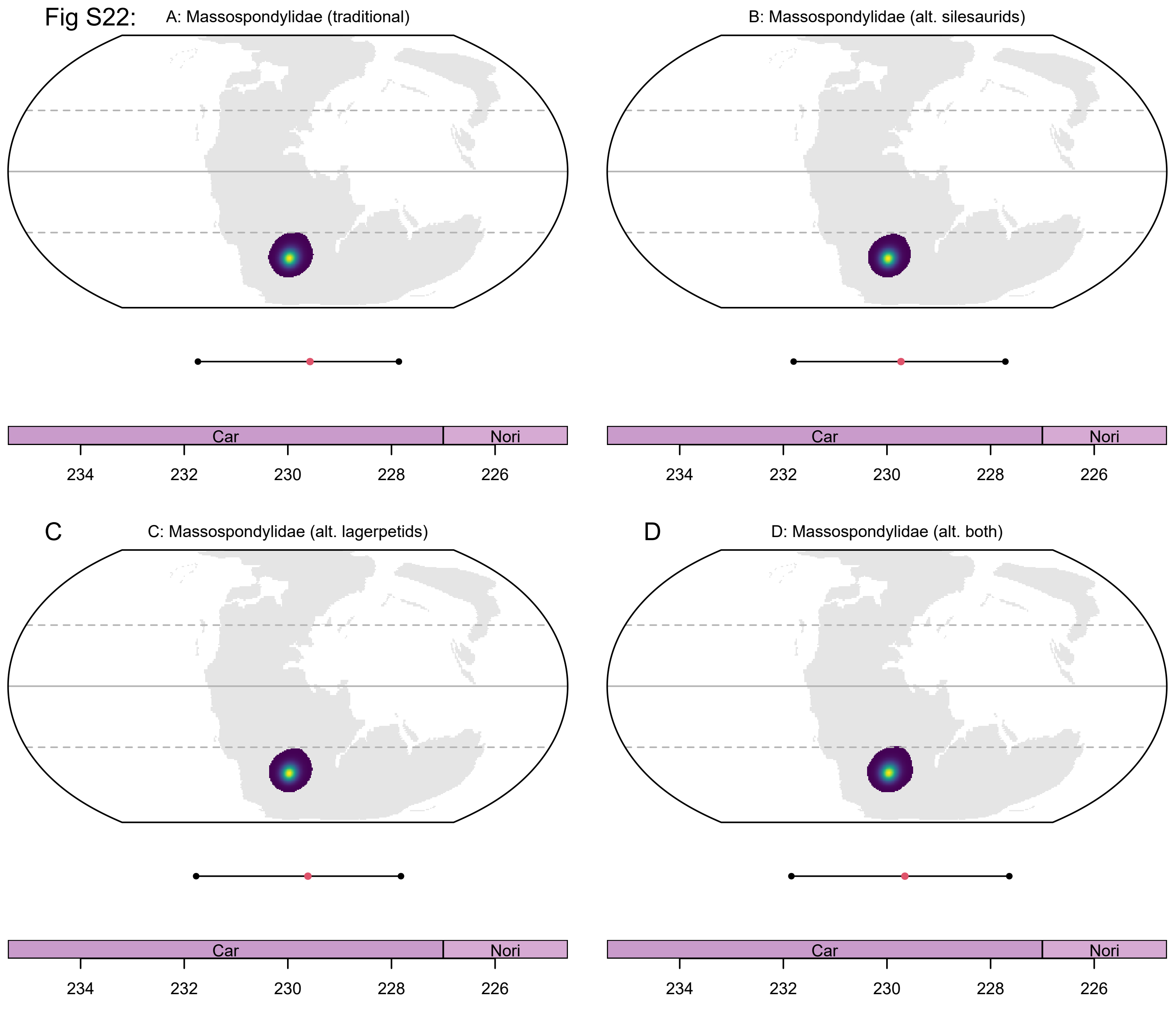
Fig S22:
A: Massospondylidae (traditional)
Car
Nori
234
232
230
228
226
B: Massospondylidae (alt. silesaurids)
Car
Nori
234
232
230
228
226
C
C: Massospondylidae (alt. lagerpetids)
Car
Nori
234
232
230
228
226
D
D: Massospondylidae (alt. both)
Car
Nori
234
232
230
228
226

Fig S23:

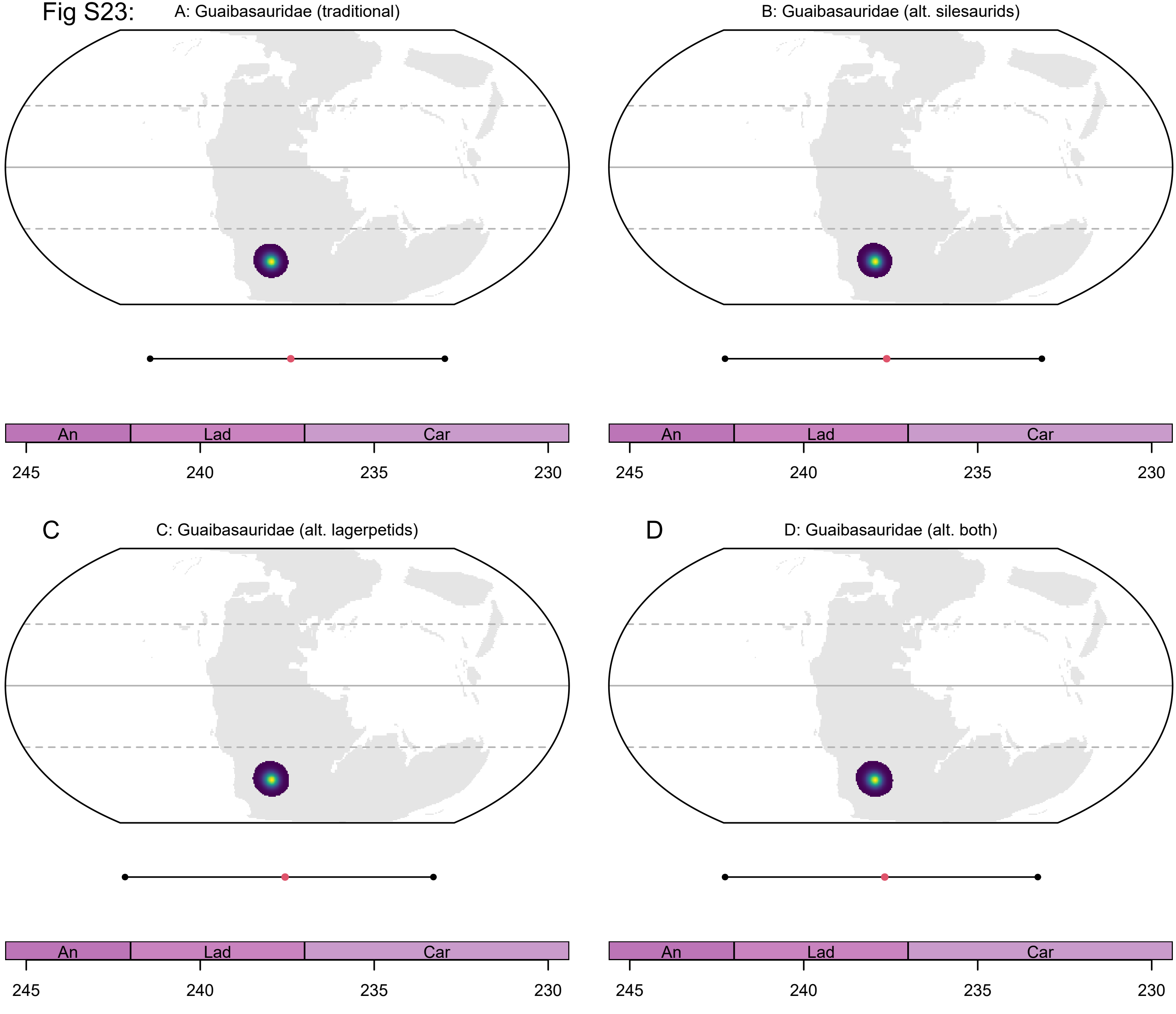

A: Guaibasauridae (traditional)
B: Guaibasauridae (alt. silesaurids)
C
C: Guaibasauridae (alt. lagerpetids)
D
D: Guaibasauridae (alt. both)
An
Lad
Car
245
240
235
230

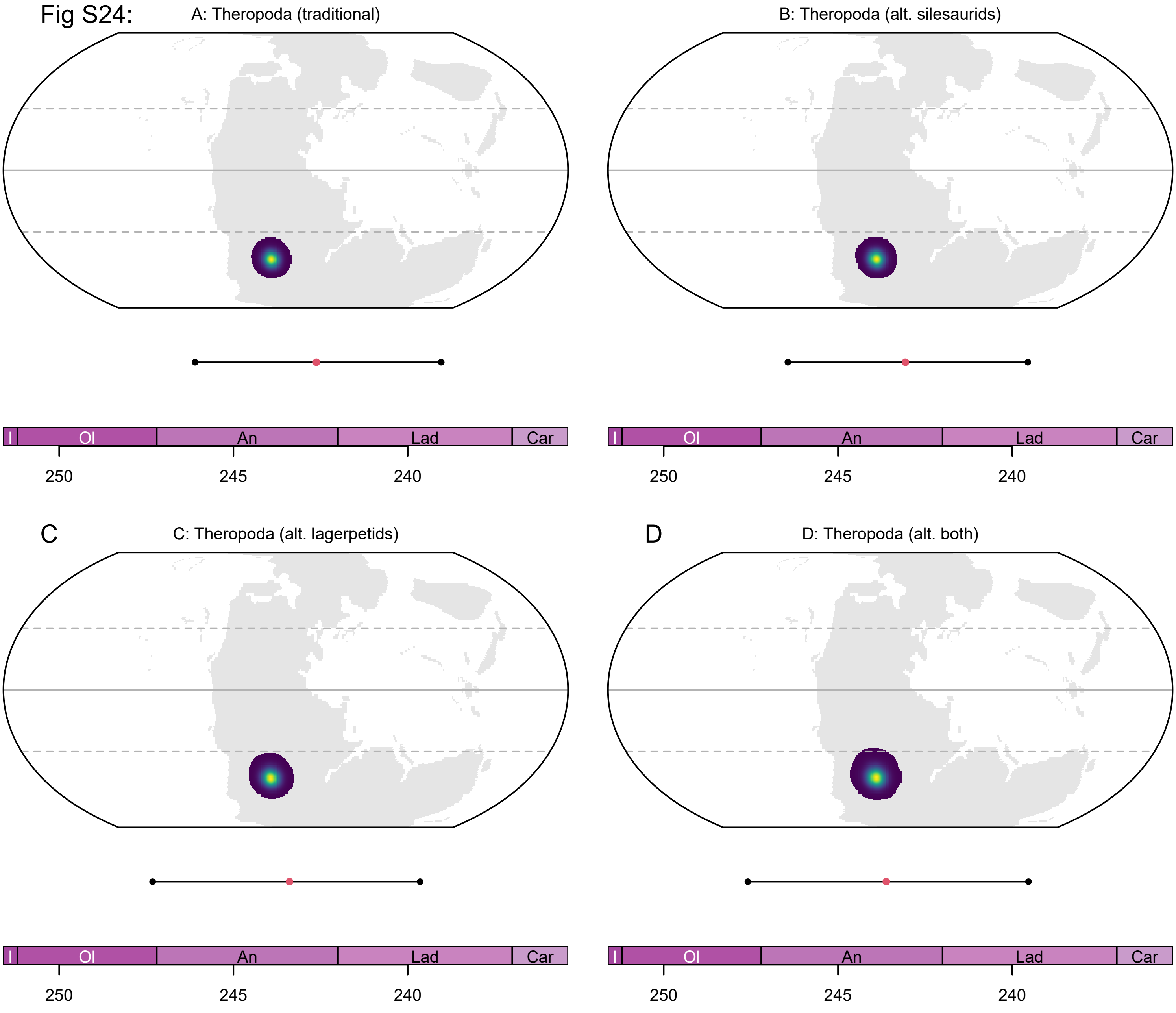

Fig S24:
A: Theropoda (traditional)
B: Theropoda (alt. silesaurids)
C
C: Theropoda (alt. lagerpetids)
D
D: Theropoda (alt. both)
I
Ol
An
Lad
Car
250
245
240

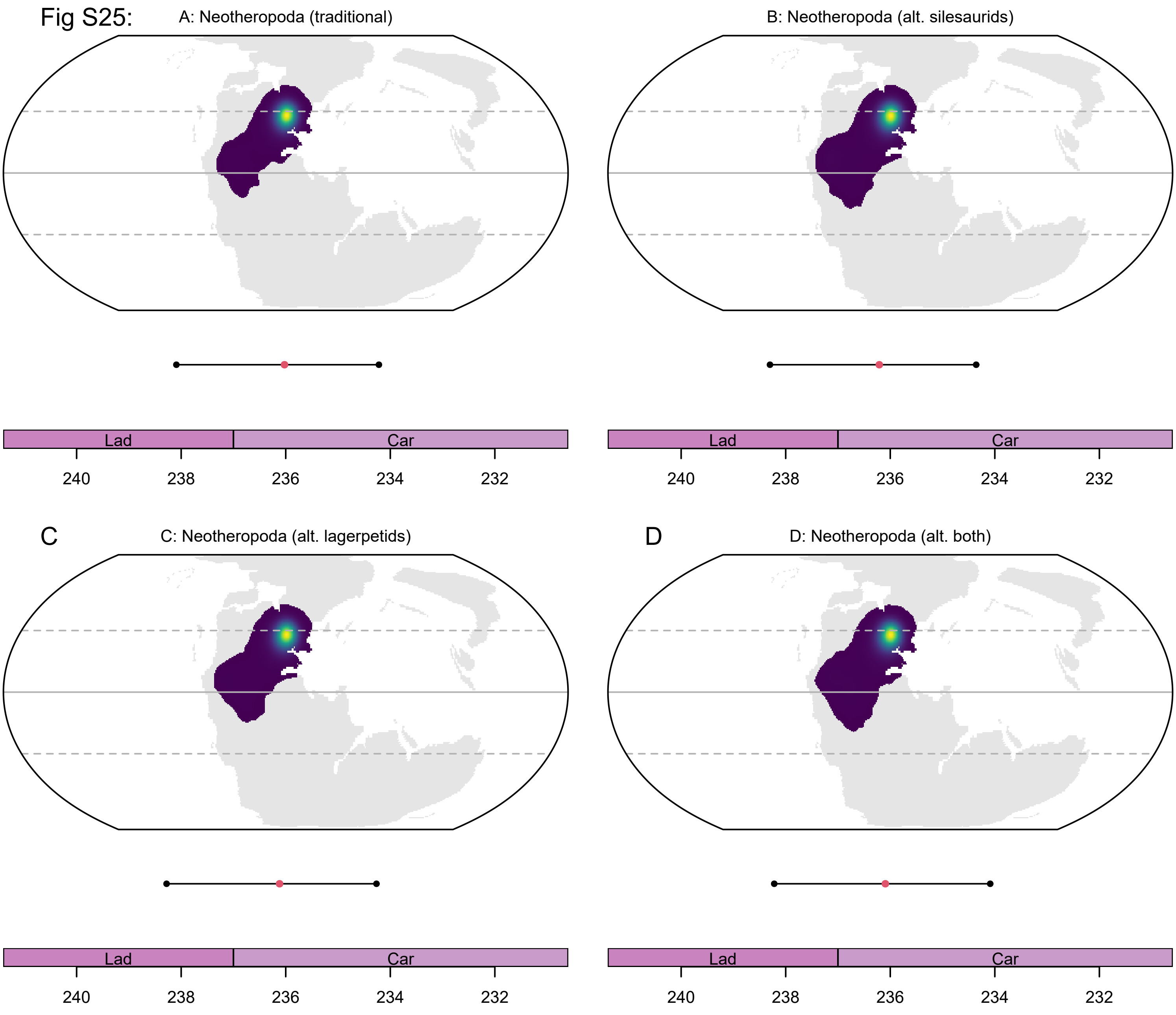
Fig S25:
A: Neotheropoda (traditional)
B: Neotheropoda (alt. silesaurids)
C
C: Neotheropoda (alt. lagerpetids)
D
D: Neotheropoda (alt. both)
Lad
Car
240
238
236
234
232

Fig S26:

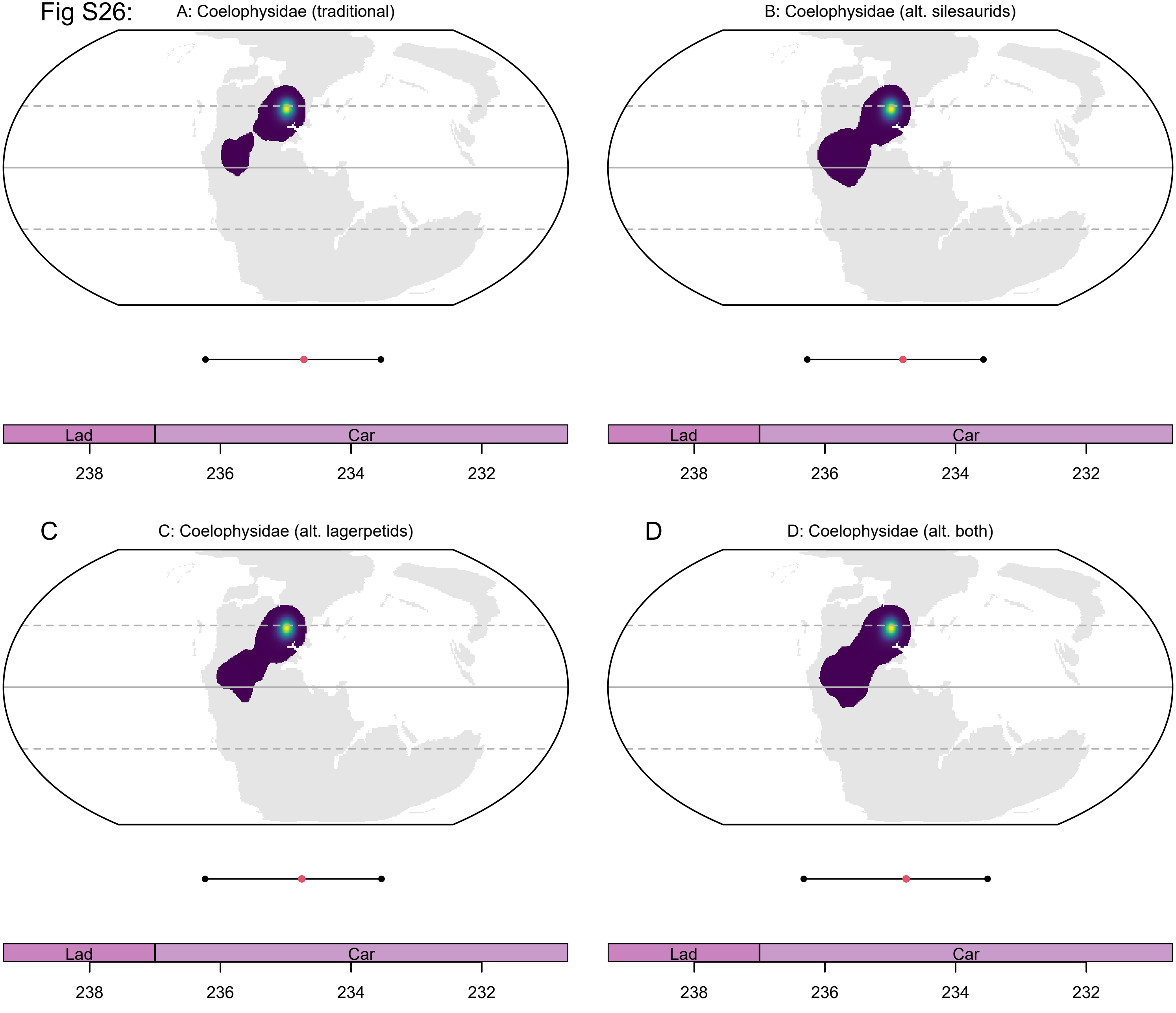

A: Coelophysidae (traditional)
Lad
Car
238
236
234
232
B: Coelophysidae (alt. silesaurids)
Lad
Car
238
236
234
232
C
C: Coelophysidae (alt. lagerpetids)
Lad
Car
238
236
234
232
D
D: Coelophysidae (alt. both)
Lad
Car
238
236
234
232

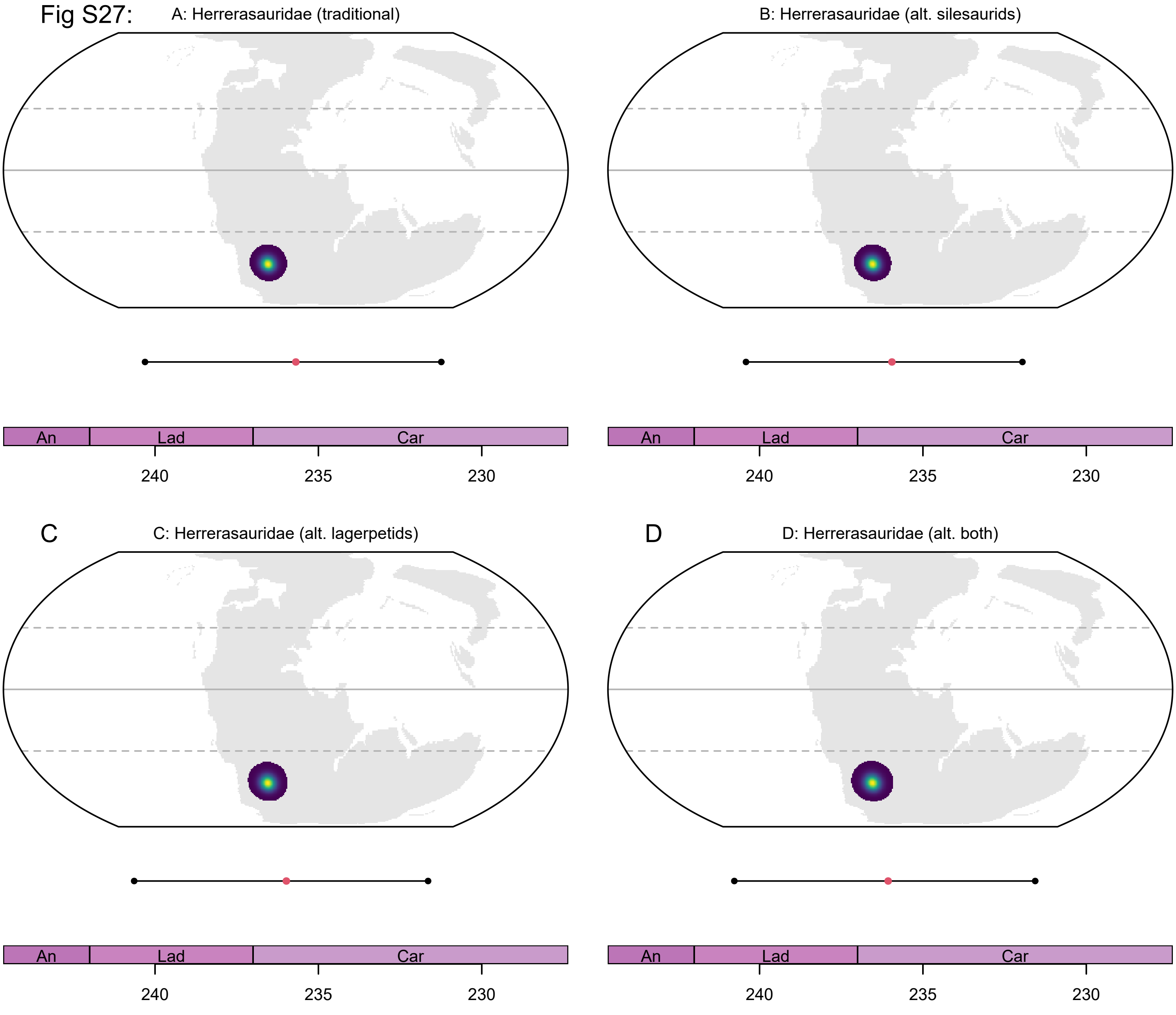
Fig S27:
A: Herrerasauridae (traditional)
B: Herrerasauridae (alt. silesaurids)
C
C: Herrerasauridae (alt. lagerpetids)
D
D: Herrerasauridae (alt. both)
An
Lad
Car
240
235
230

Fig S28:

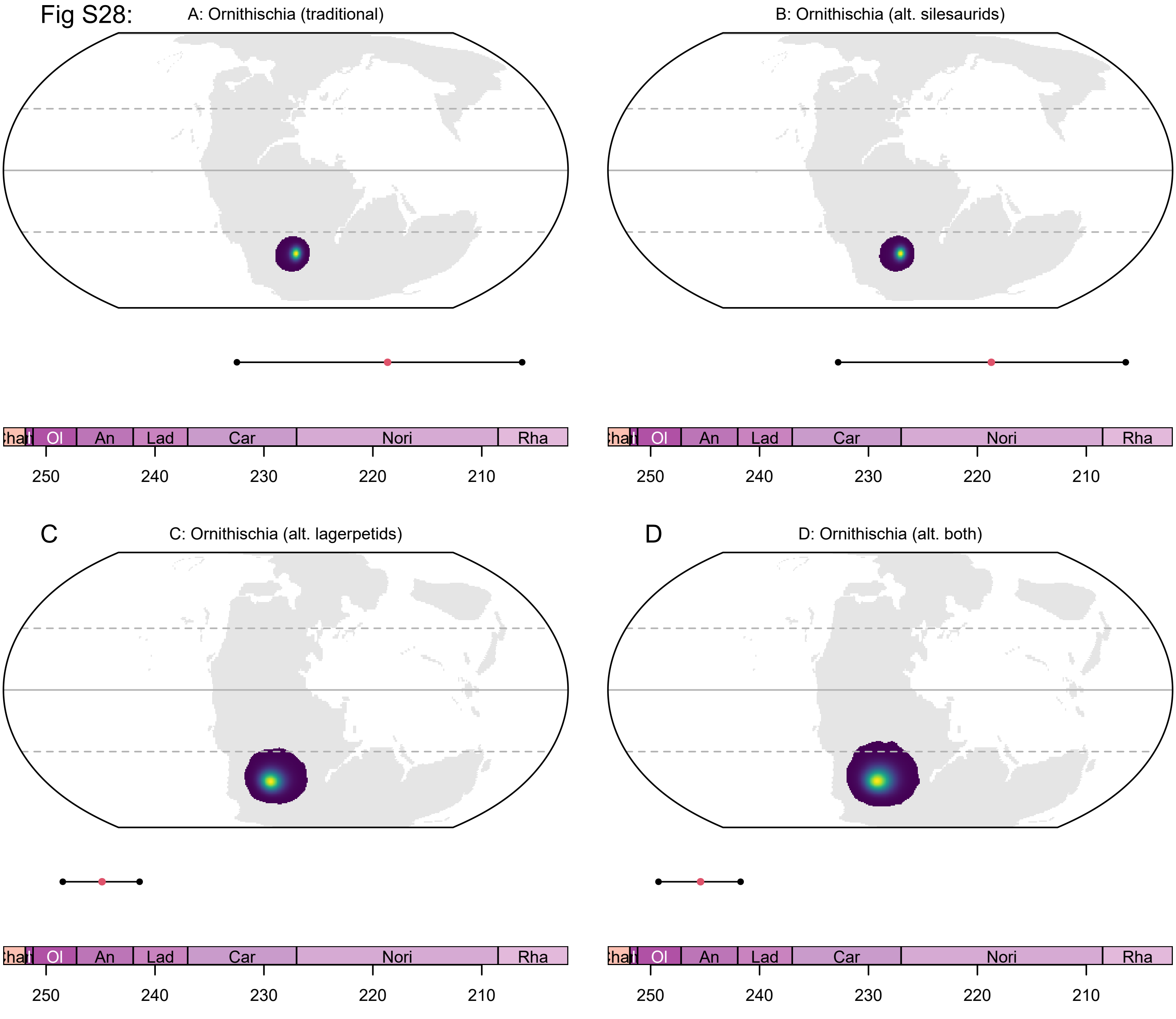
A: Ornithischia (traditional)
B: Ornithischia (alt. silesaurids)
C
C: Ornithischia (alt. lagerpetids)
D
D: Ornithischia (alt. both)
Ol
An
Lad
Car
Nori
Rha
250
240
230
220
210

Fig S29:

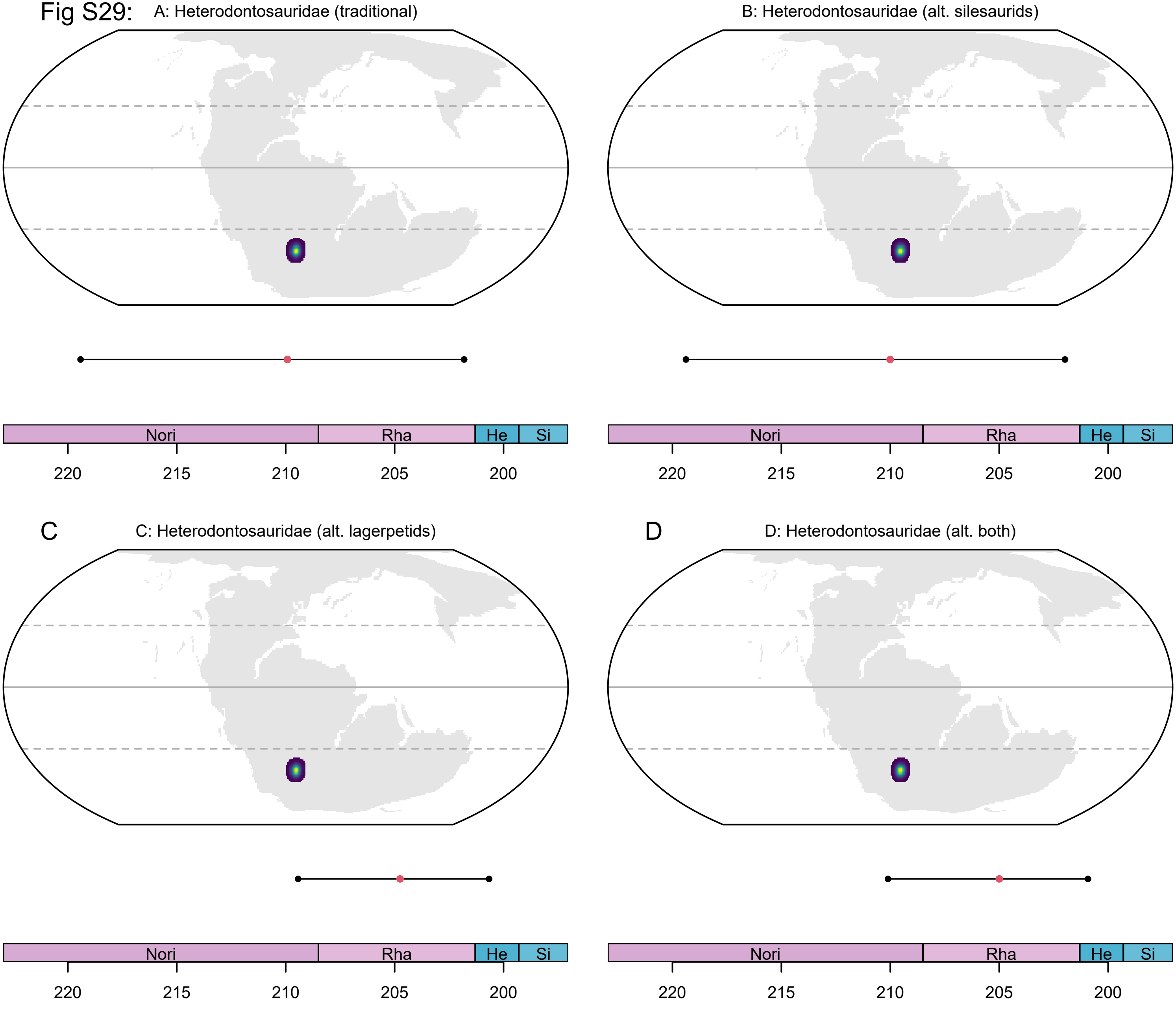
A: Heterodontosauridae (traditional)
B: Heterodontosauridae (alt. silesaurids)
C
C: Heterodontosauridae (alt. lagerpetids)
D
D: Heterodontosauridae (alt. both)
Nori
Rha
He
Si
220
215
210
205
200

Fig S30:

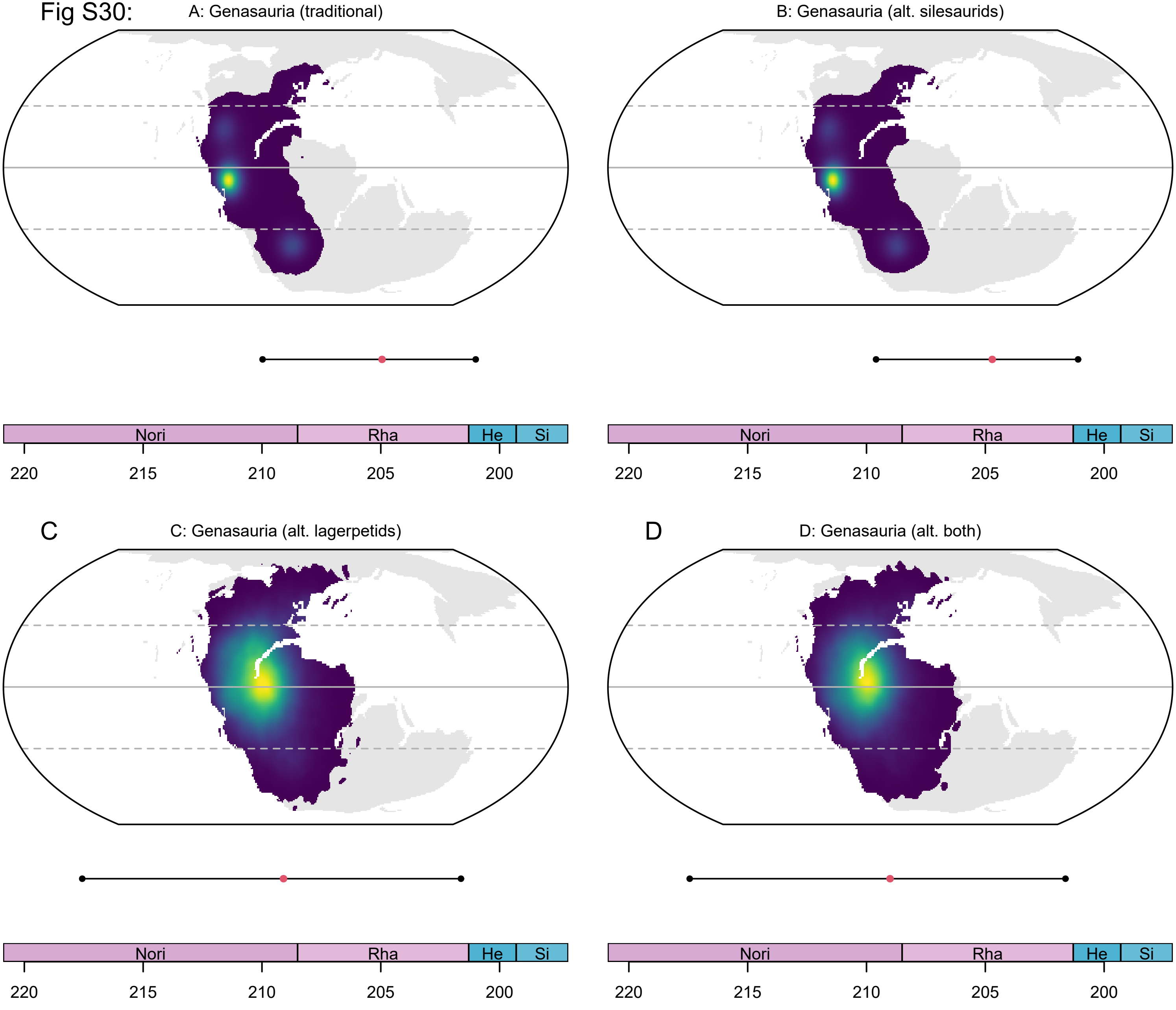
A: Genasauria (traditional)
B: Genasauria (alt. silesaurids)
C
C: Genasauria (alt. lagerpetids)
D
D: Genasauria (alt. both)
Nori
Rha
He
Si
220
215
210
205
200

Fig S31:

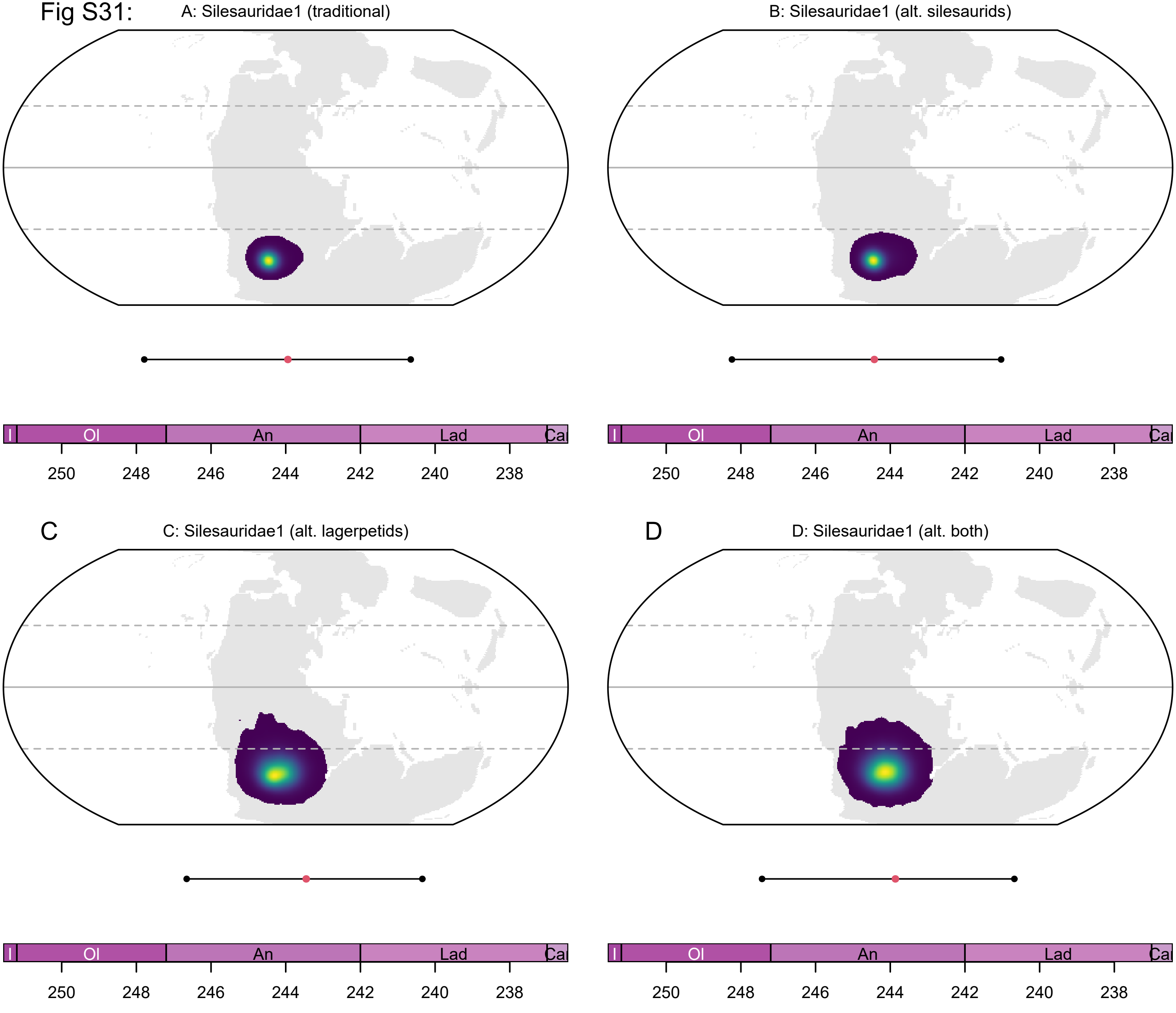
A: Silesauridae1 (traditional)
B: Silesauridae1 (alt. silesaurids)
C
C: Silesauridae1 (alt. lagerpetids)
D
D: Silesauridae1 (alt. both)
I
Ol
An
Lad
Cal
250
248
246
244
242
240
238

Fig S32:

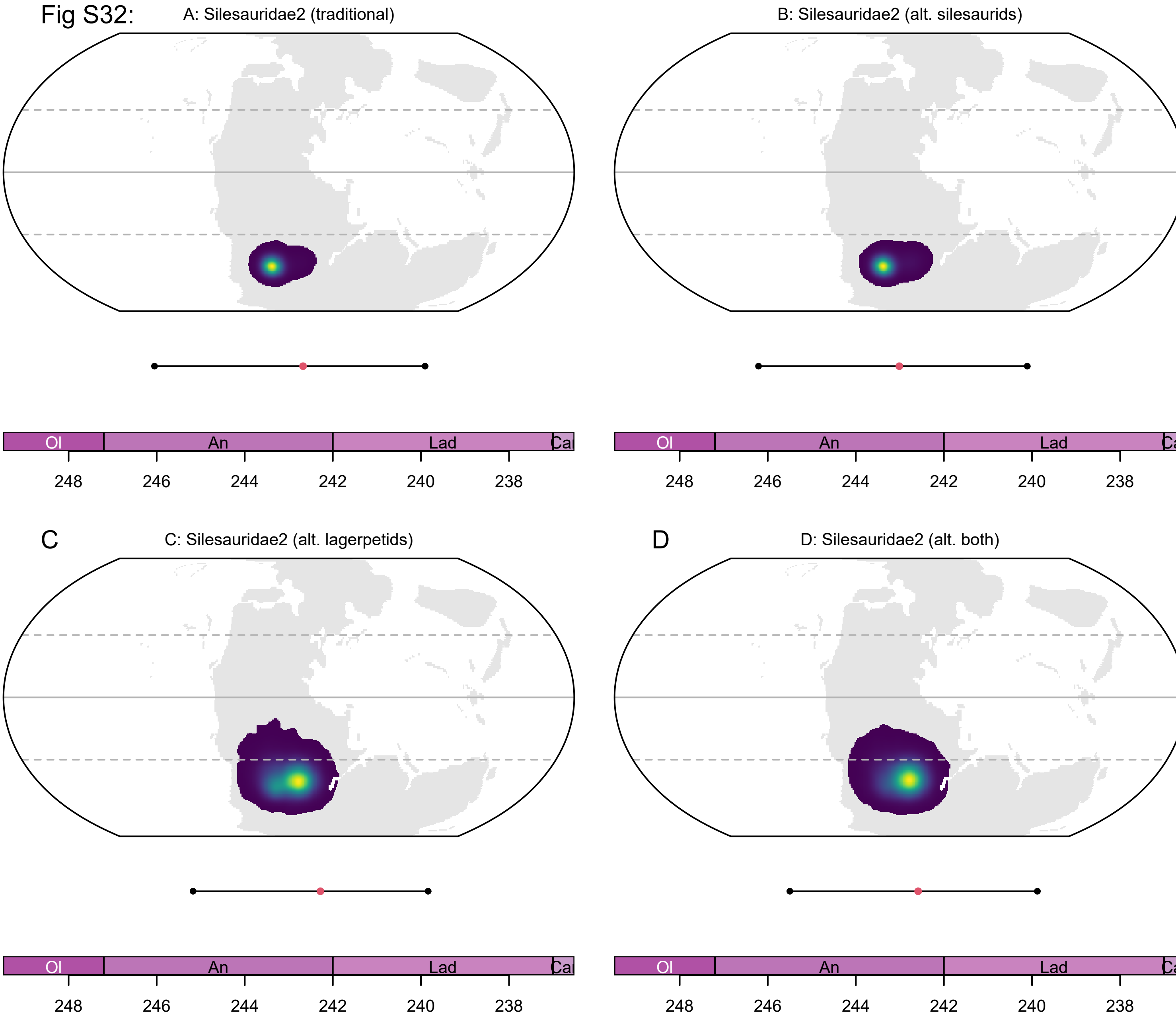
A: Silesauridae2 (traditional)
B: Silesauridae2 (alt. silesaurids)
C
C: Silesauridae2 (alt. lagerpetids)
D
D: Silesauridae2 (alt. both)
Ol
An
Lad
Car
248
246
244
242
240
238

Fig S33:

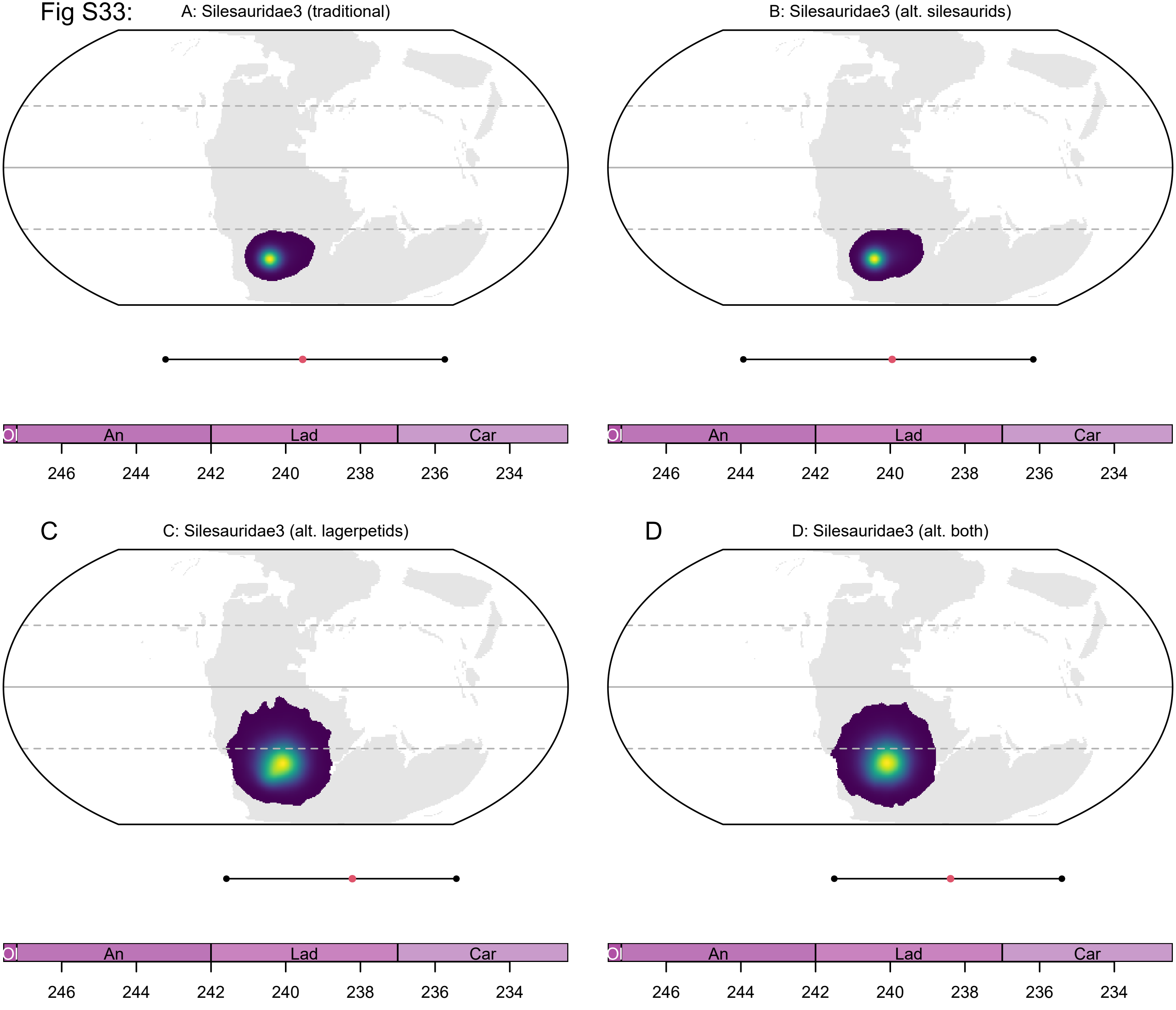
A: Silesauridae3 (traditional)
B: Silesauridae3 (alt. silesaurids)
C
C: Silesauridae3 (alt. lagerpetids)
D
D: Silesauridae3 (alt. both)
Ol
An
Lad
Car
246
244
242
240
238
236
234

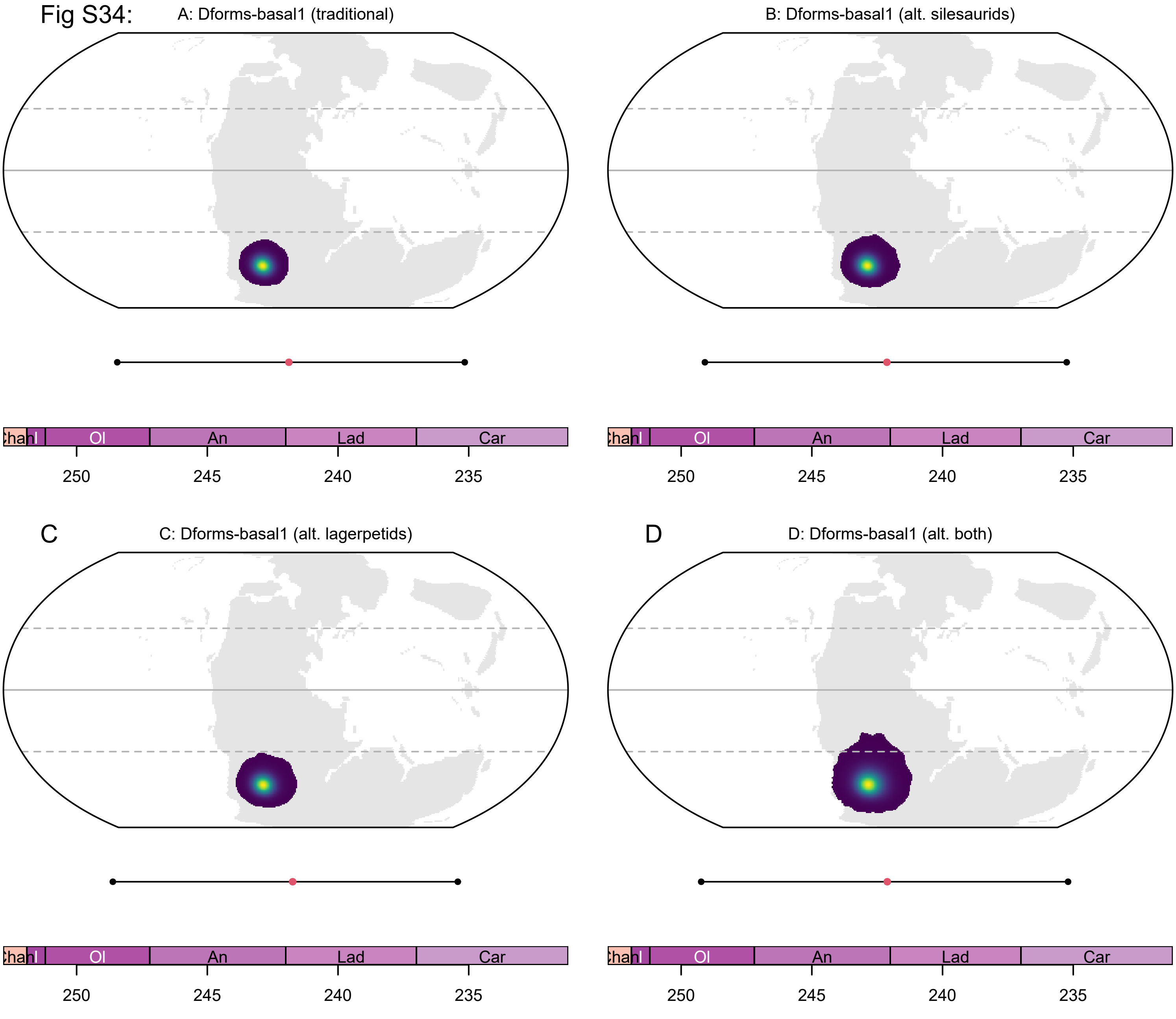

Fig S34:
A: Dforms-basal1 (traditional)
B: Dforms-basal1 (alt. silesaurids)
C
C: Dforms-basal1 (alt. lagerpetids)
D
D: Dforms-basal1 (alt. both)
Chan
I
Ol
An
Lad
Car
250
245
240
235

Fig S35:

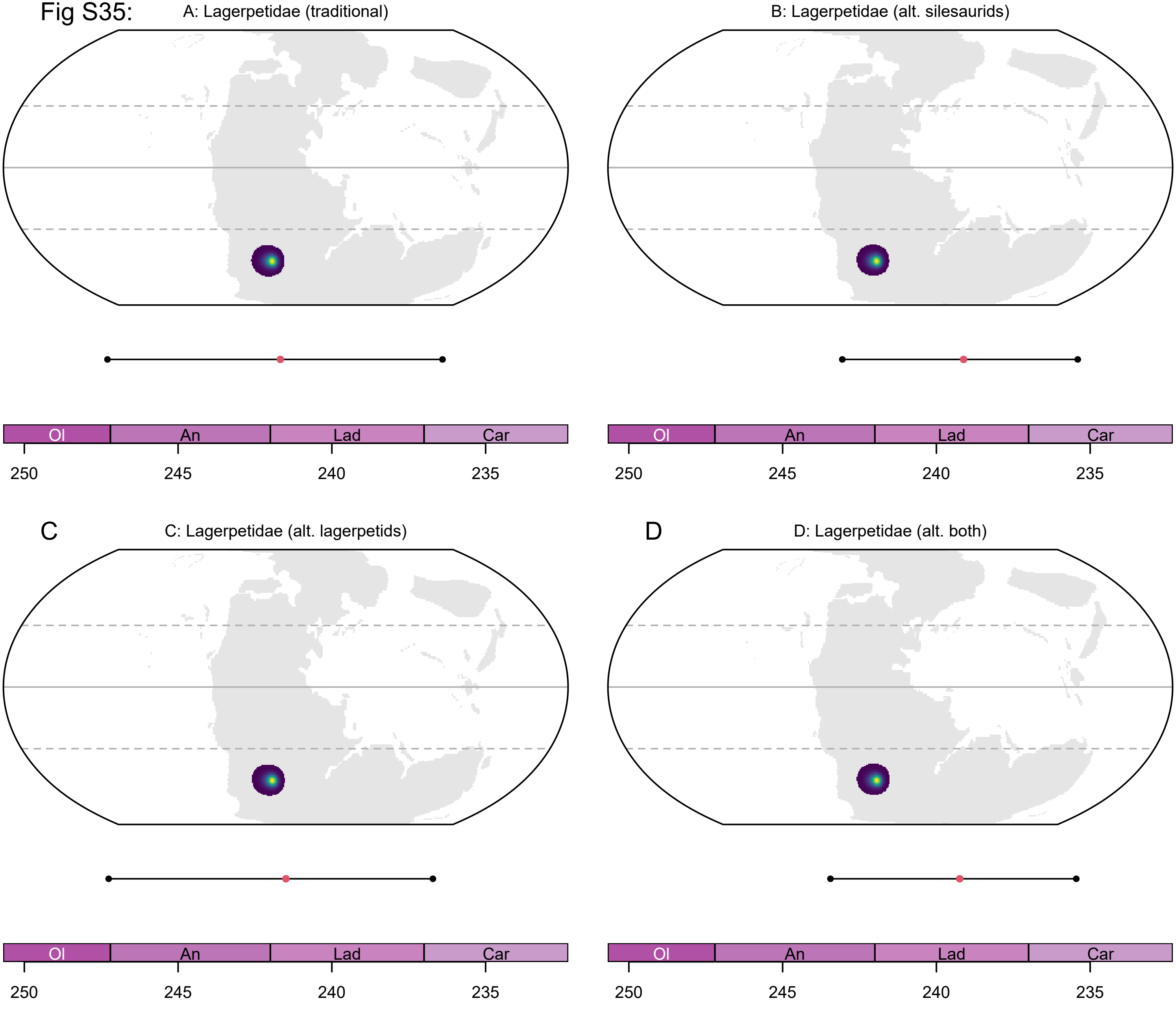

A: Lagerpetidae (traditional)
B: Lagerpetidae (alt. silesaurids)
C
C: Lagerpetidae (alt. lagerpetids)
D
D: Lagerpetidae (alt. both)
Ol
An
Lad
Car
250
245
240
235

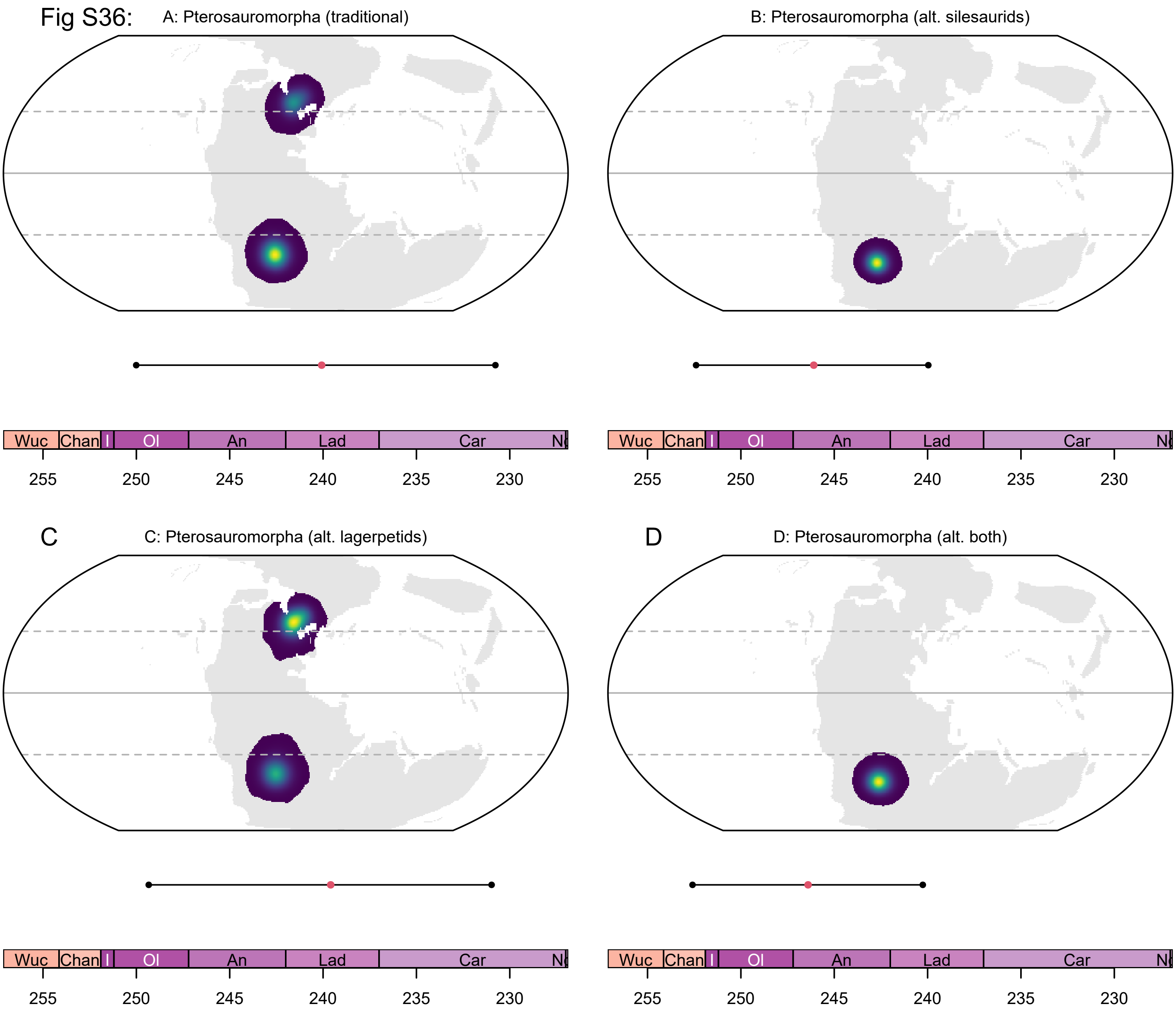

Fig S36:
A: Pterosauromorpha (traditional)
B: Pterosauromorpha (alt. silesaurids)
C
C: Pterosauromorpha (alt. lagerpetids)
D
D: Pterosauromorpha (alt. both)
Wuc
Chan
I
Ol
An
Lad
Car
255
250
245
240
235
230

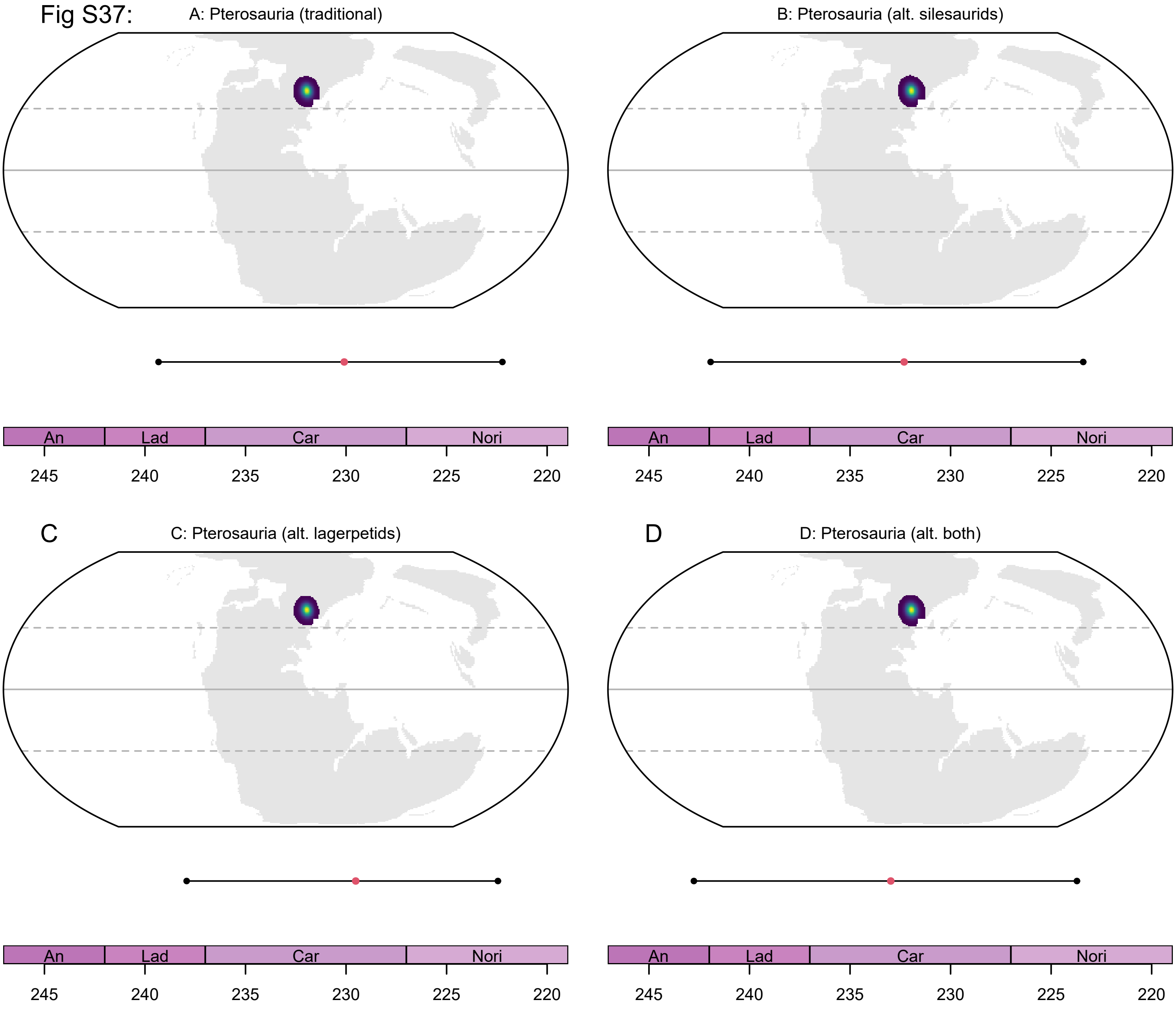

Fig S37:
A: Pterosauria (traditional)
B: Pterosauria (alt. silesaurids)
C
C: Pterosauria (alt. lagerpetids)
D
D: Pterosauria (alt. both)
An
Lad
Car
Nori
245
240
235
230
225
220

Fig S38:

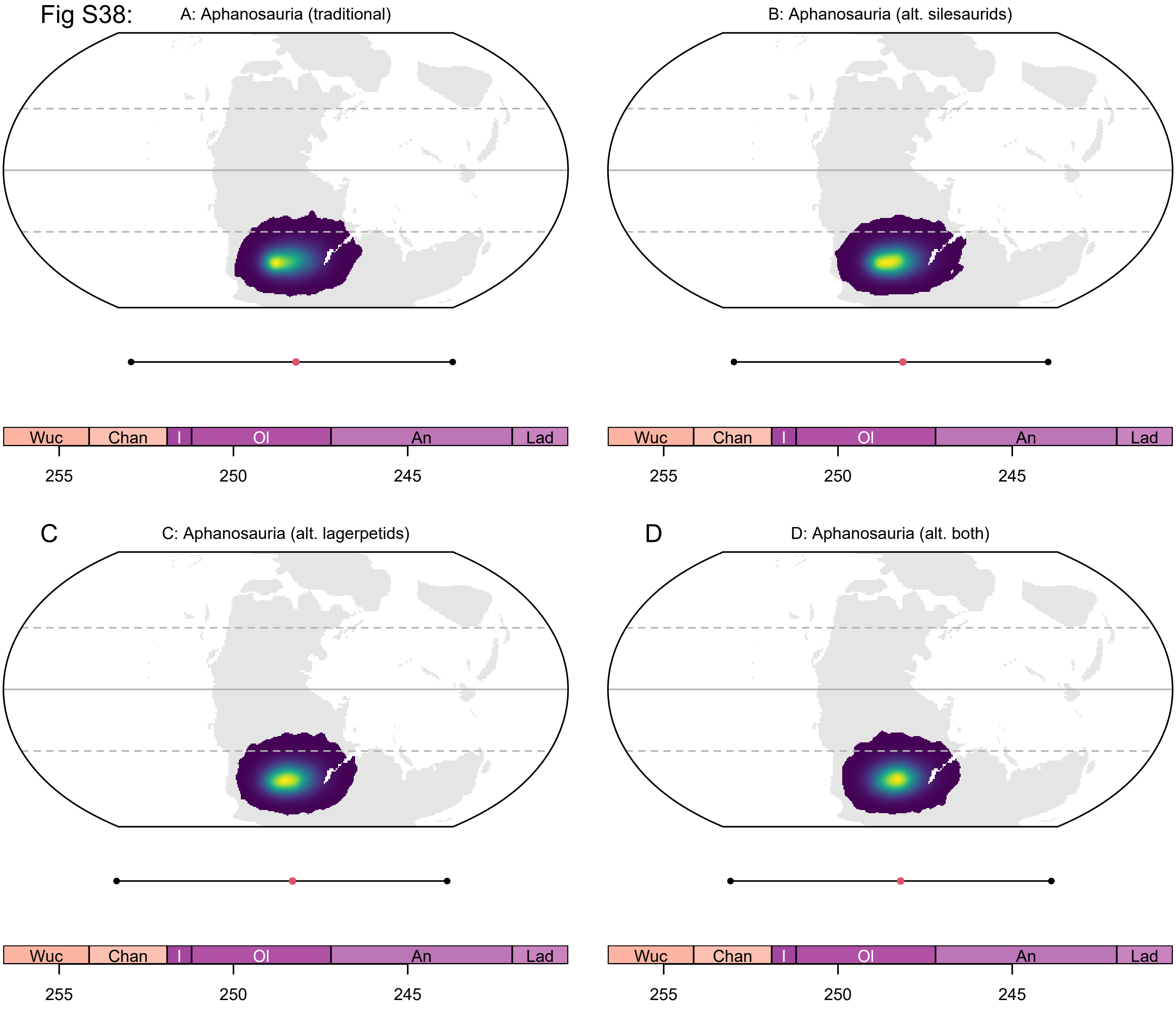

A: Aphanosauria (traditional)
B: Aphanosauria (alt. silesaurids)
C
C: Aphanosauria (alt. lagerpetids)
D
D: Aphanosauria (alt. both)
Wuc
Chan
I
Ol
An
Lad
255
250
245

Fig S39:

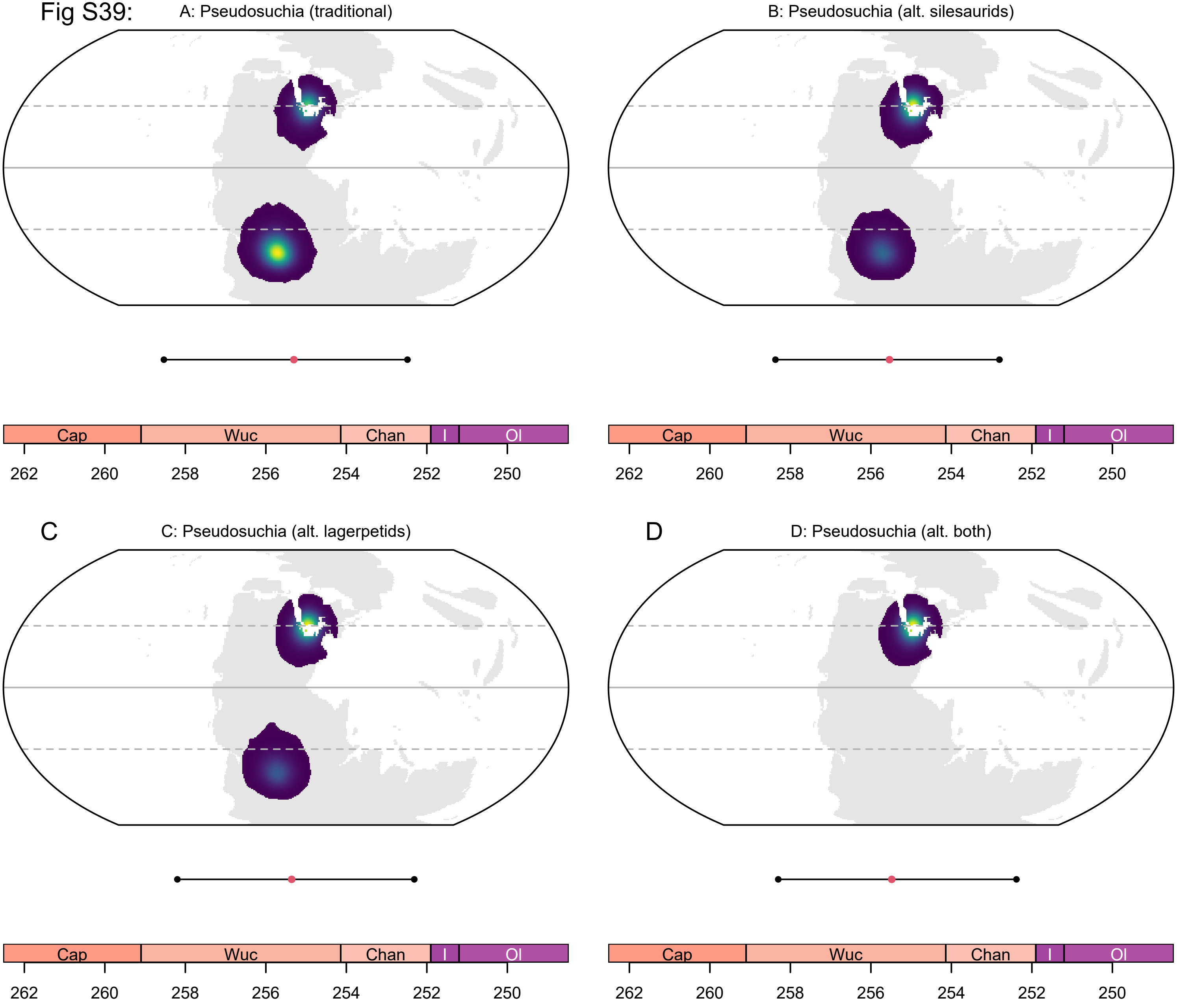
A: Pseudosuchia (traditional)
B: Pseudosuchia (alt. silesaurids)
C
C: Pseudosuchia (alt. lagerpetids)
D
D: Pseudosuchia (alt. both)
Cap
Wuc
Chan
I
Ol
262
260
258
256
254
252
250

Fig S40:

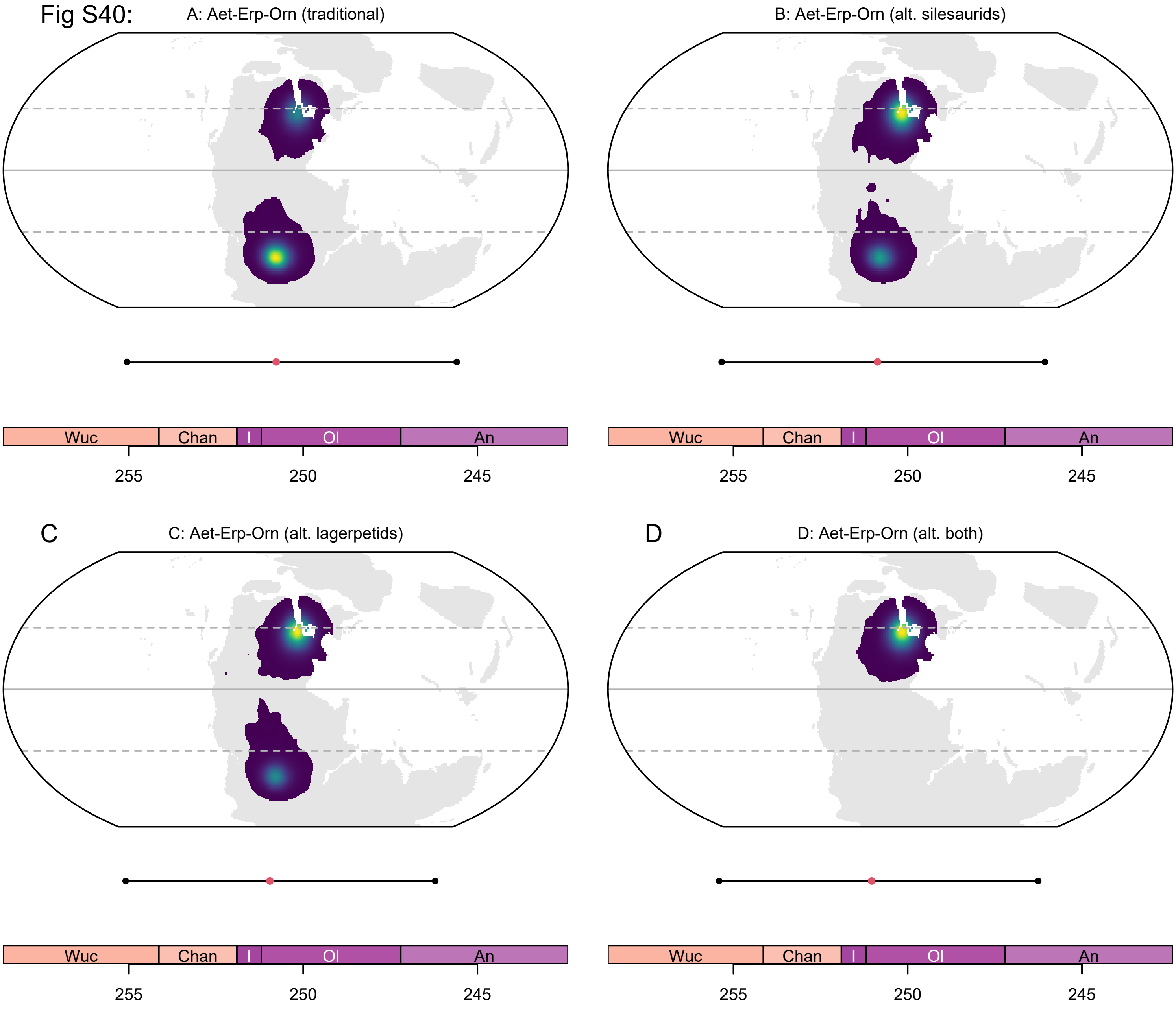
A: Aet-Erp-Orn (traditional)
B: Aet-Erp-Orn (alt. silesaurids)
C
C: Aet-Erp-Orn (alt. lagerpetids)
D
D: Aet-Erp-Orn (alt. both)
Wuc
Chan
I
Ol
An
255
250
245

Fig S41:

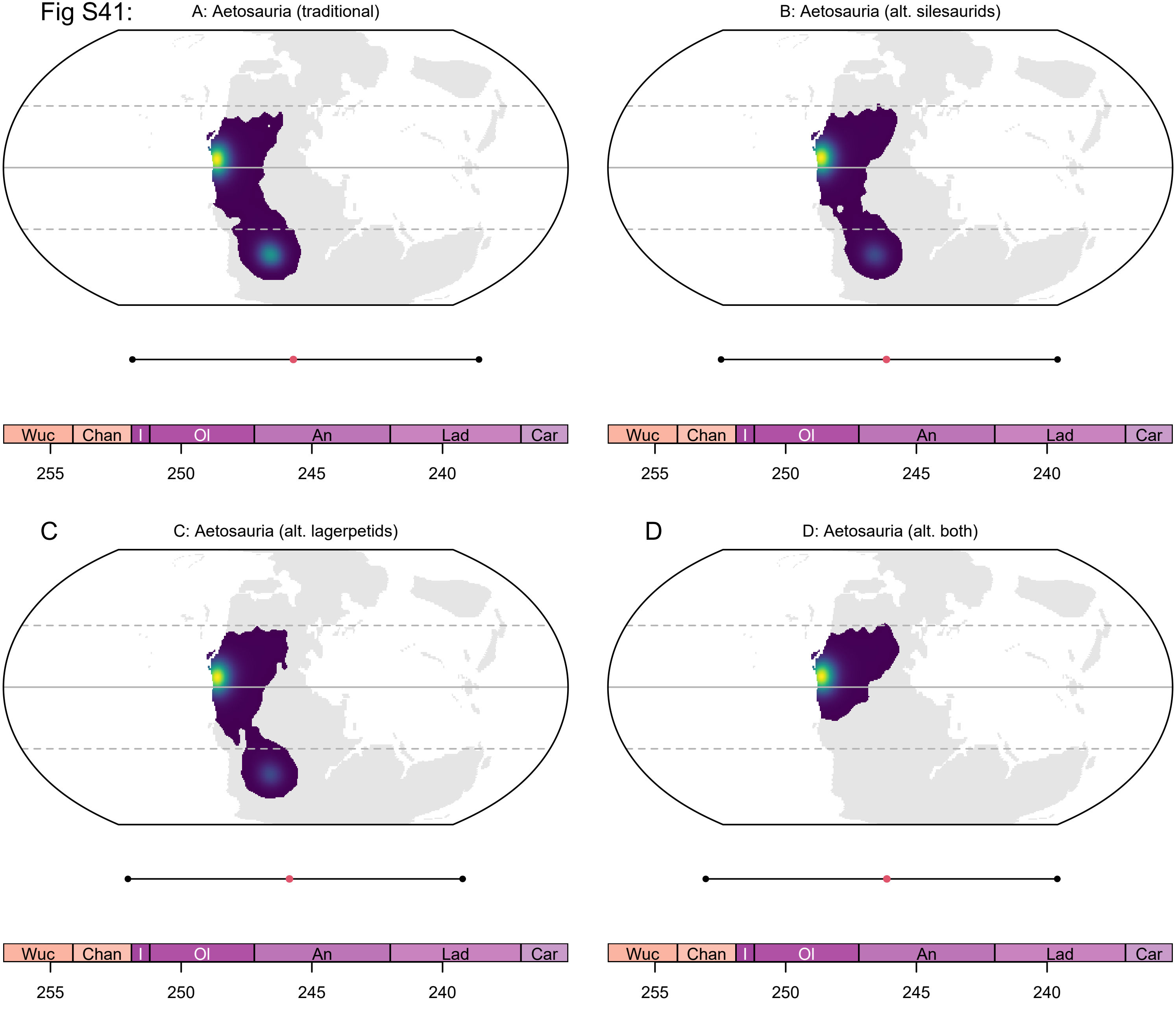
A: Aetosauria (traditional)
B: Aetosauria (alt. silesaurids)
C
C: Aetosauria (alt. lagerpetids)
D
D: Aetosauria (alt. both)
Wuc
Chan
I
Ol
An
Lad
Car
255
250
245
240

Fig S42:

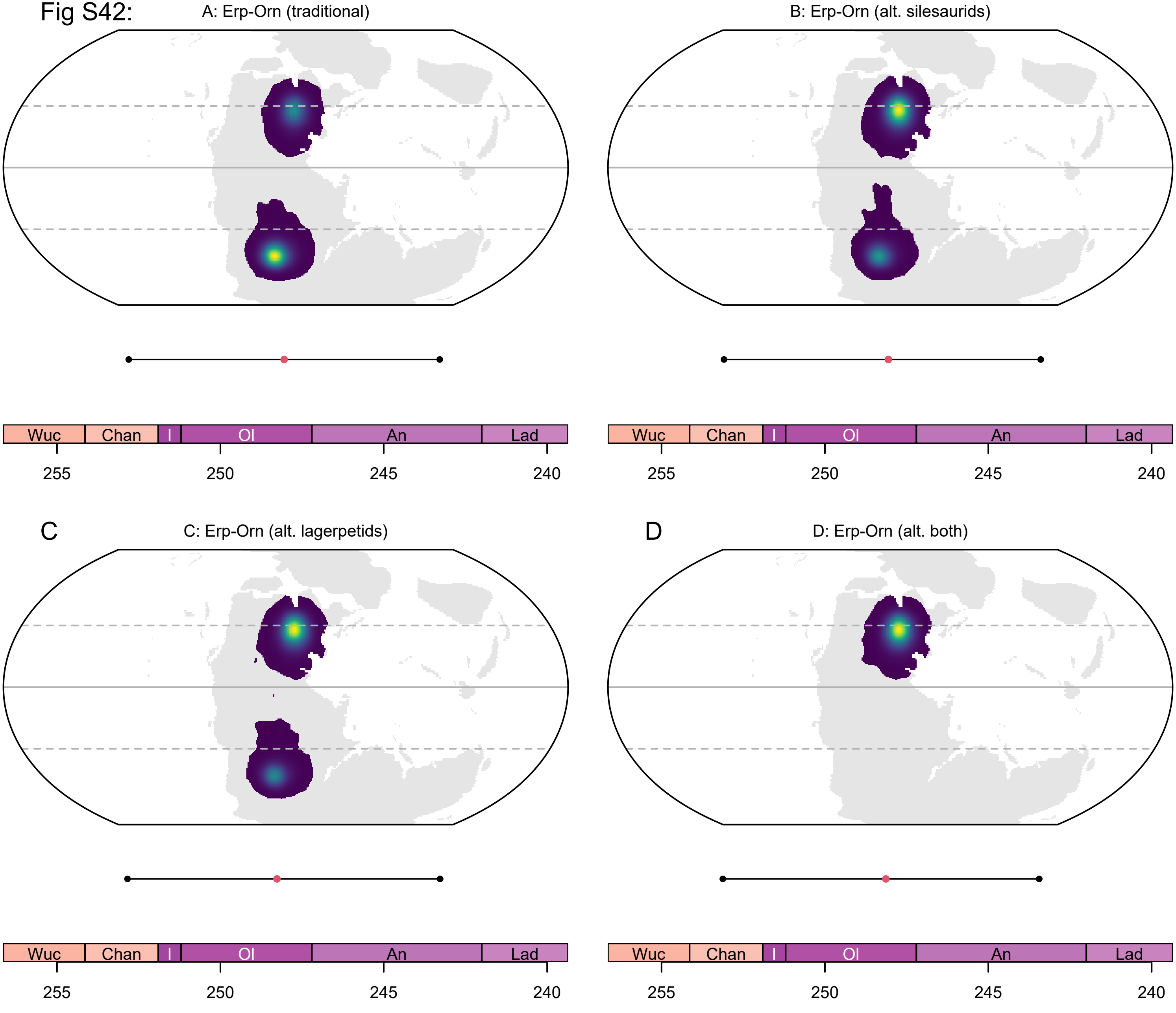
A: Erp-Orn (traditional)
B: Erp-Orn (alt. silesaurids)
C
C: Erp-Orn (alt. lagerpetids)
D
D: Erp-Orn (alt. both)
Wuc
Chan
I
Ol
An
Lad
255
250
245
240

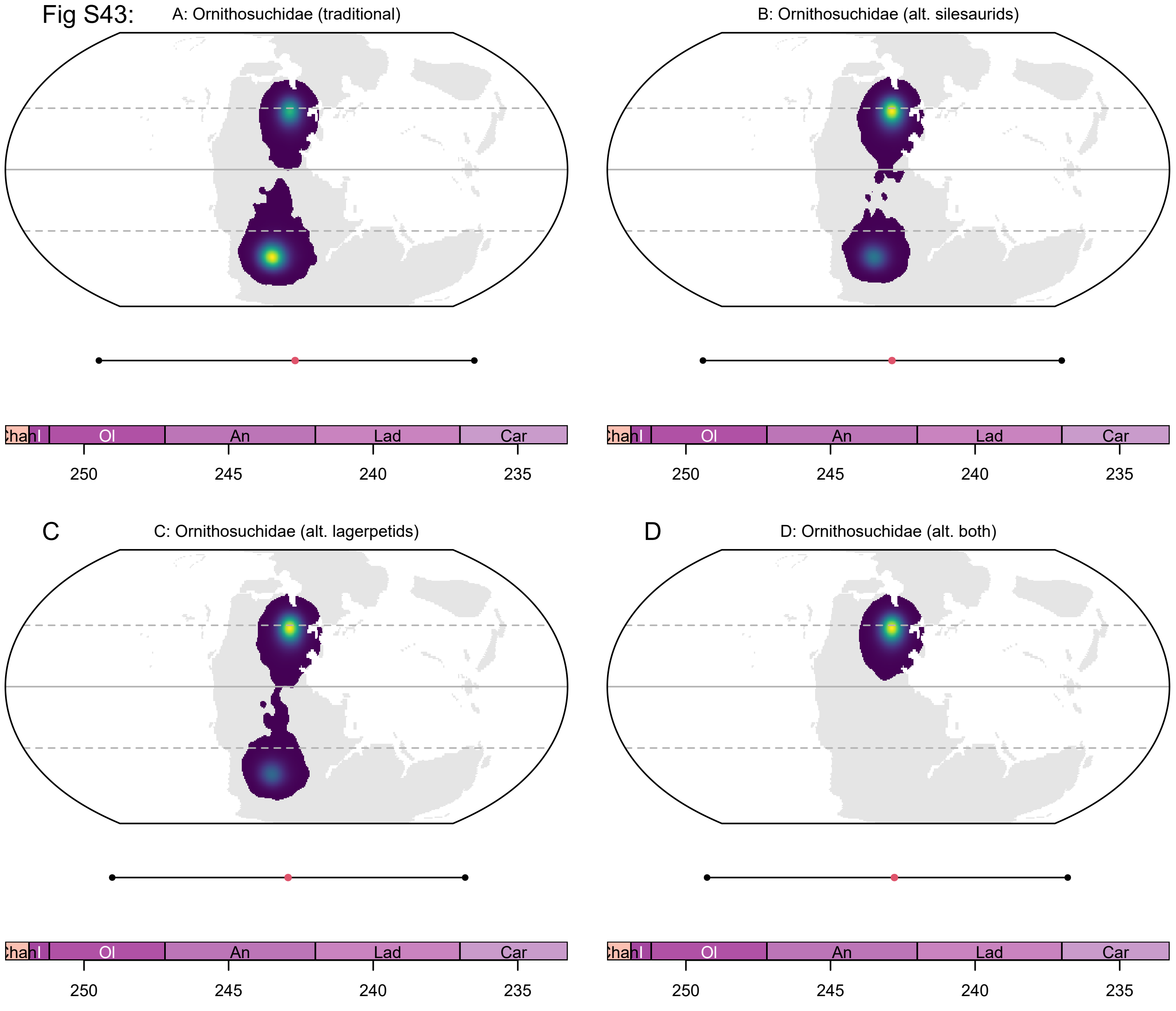
Fig S43:
A: Ornithosuchidae (traditional)
B: Ornithosuchidae (alt. silesaurids)
C
C: Ornithosuchidae (alt. lagerpetids)
D
D: Ornithosuchidae (alt. both)
Chan
I
Ol
An
Lad
Car
250
245
240
235

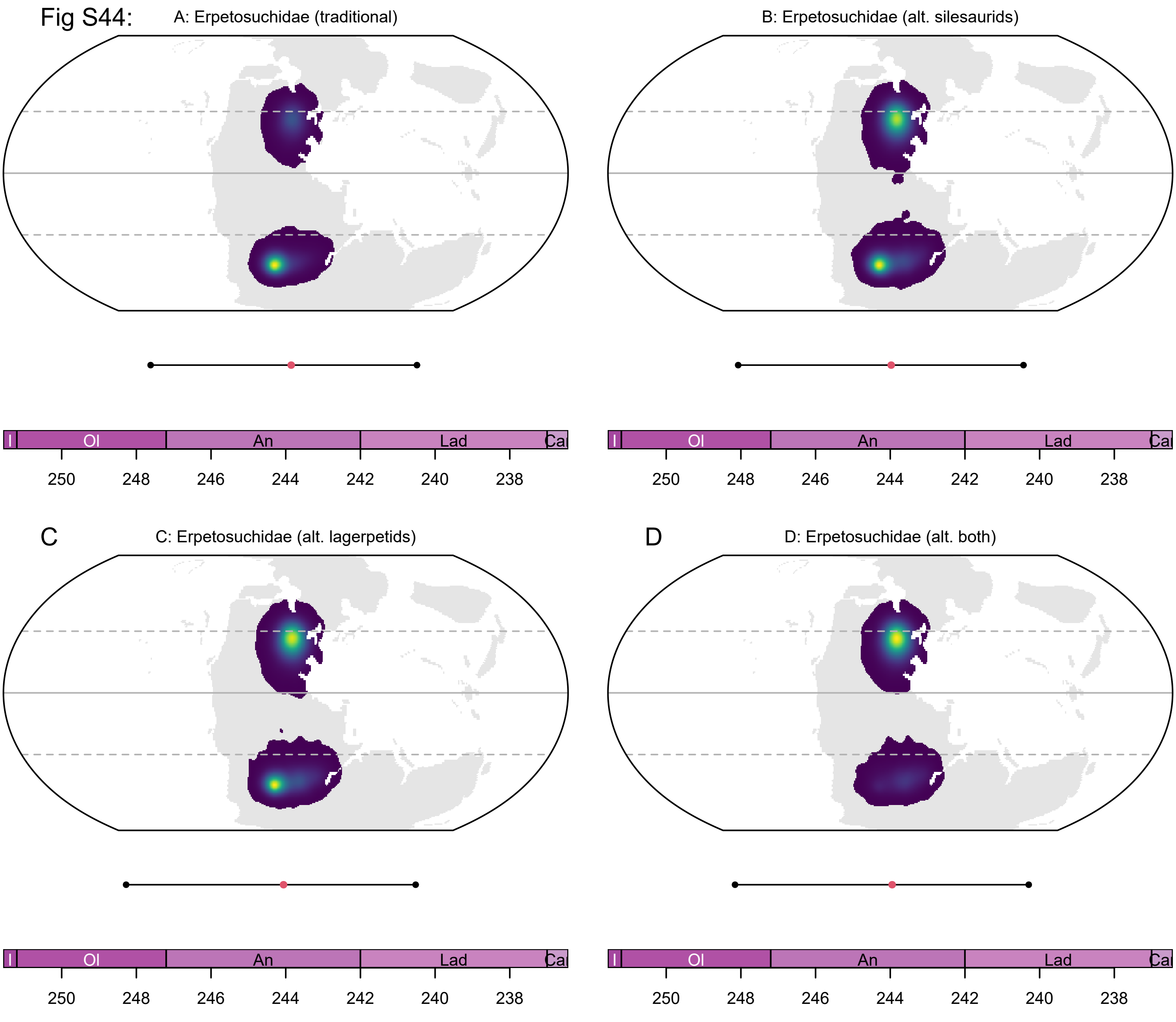
Fig S44:
A: Erpetosuchidae (traditional)
B: Erpetosuchidae (alt. silesaurids)
C
C: Erpetosuchidae (alt. lagerpetids)
D
D: Erpetosuchidae (alt. both)
I
Ol
An
Lad
Cal
250
248
246
244
242
240
238

Fig S45:

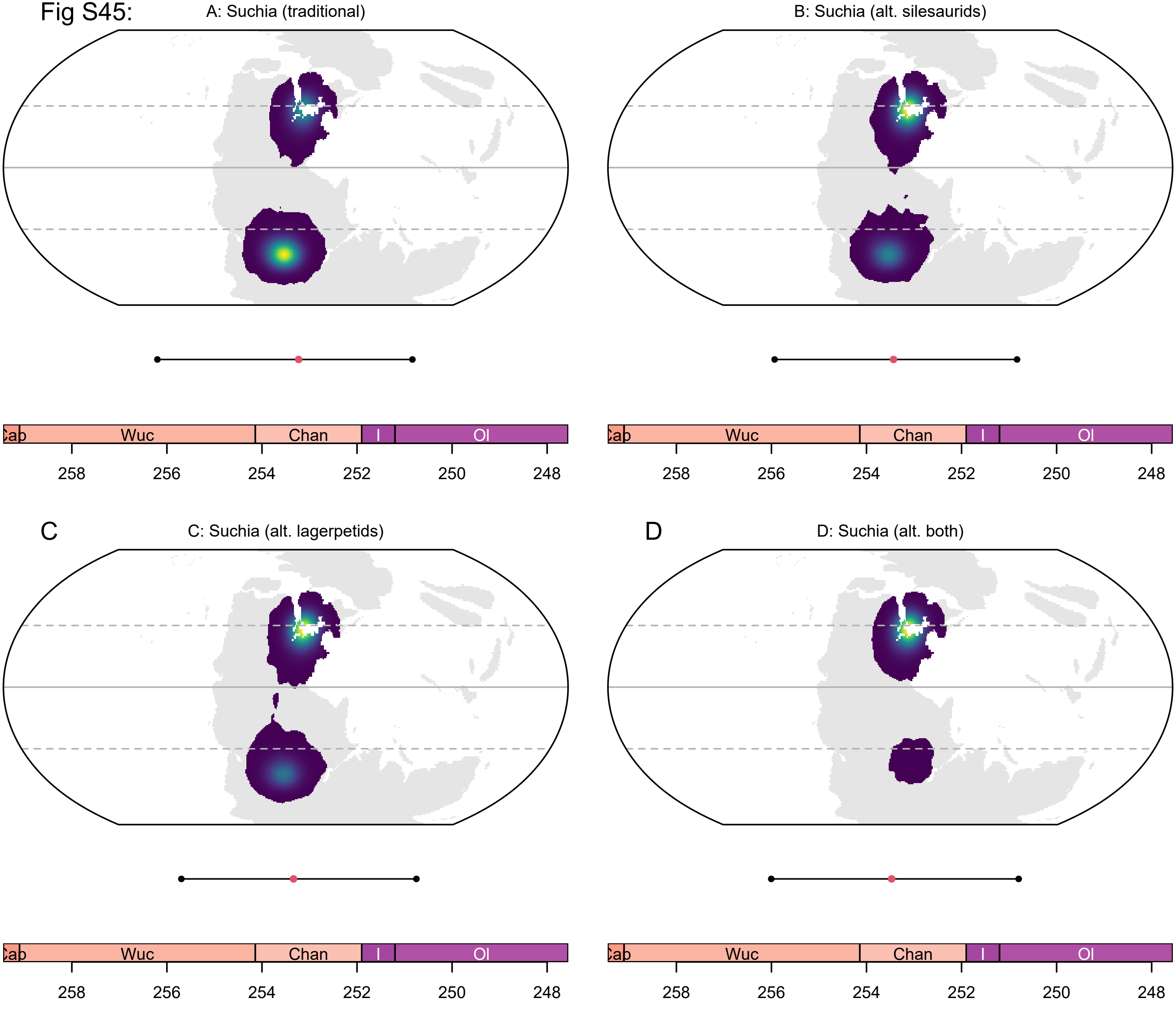
A: Suchia (traditional)
B: Suchia (alt. silesaurids)
C
C: Suchia (alt. lagerpetids)
D
D: Suchia (alt. both)
Cap
Wuc
Chan
I
Ol
258
256
254
252
250
248

Fig S46:

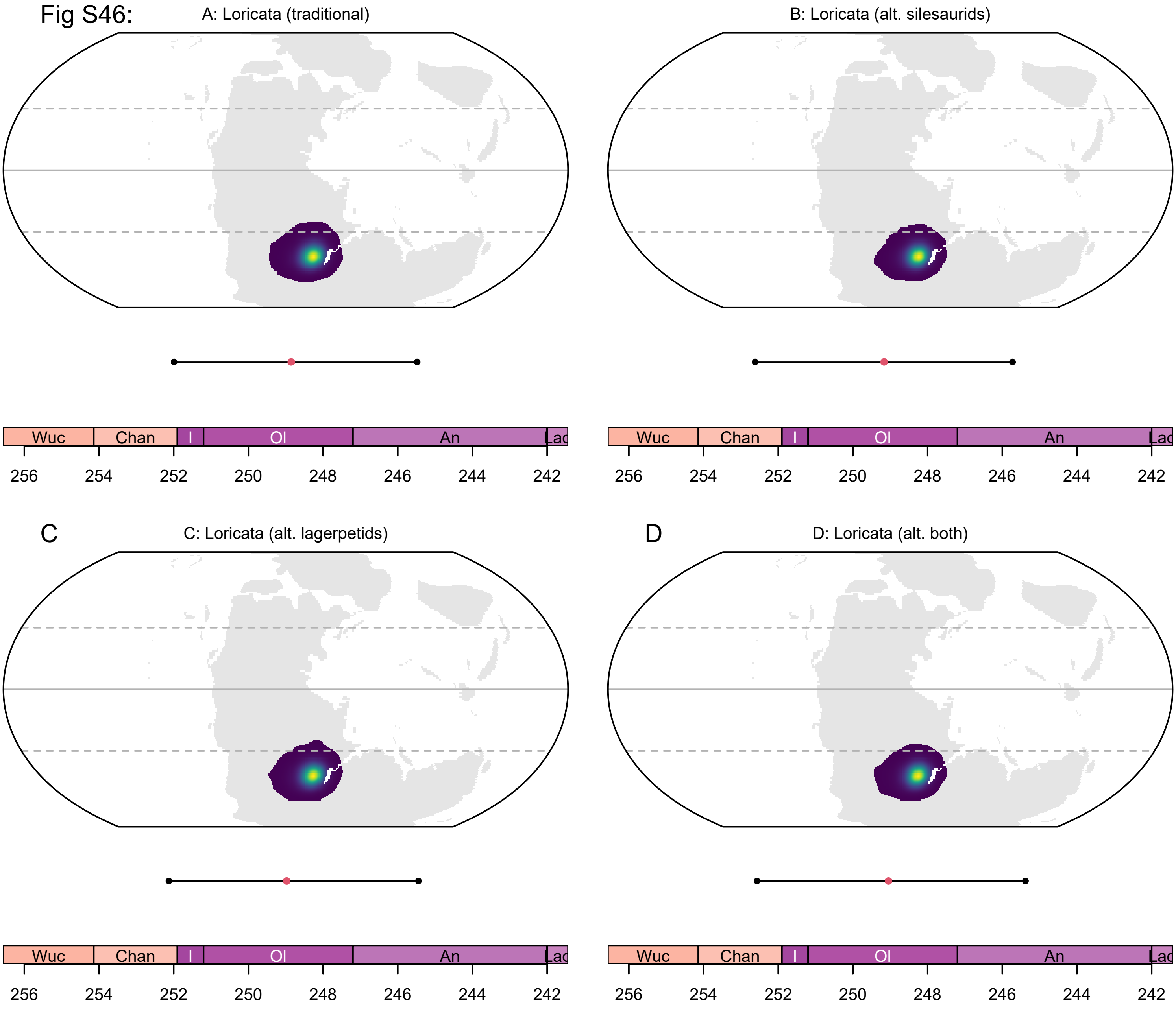

A: Loricata (traditional)
B: Loricata (alt. silesaurids)
C
C: Loricata (alt. lagerpetids)
D
D: Loricata (alt. both)
Wuc
Chan
I
Ol
An
Lad
256
254
252
250
248
246
244
242

Fig S47:

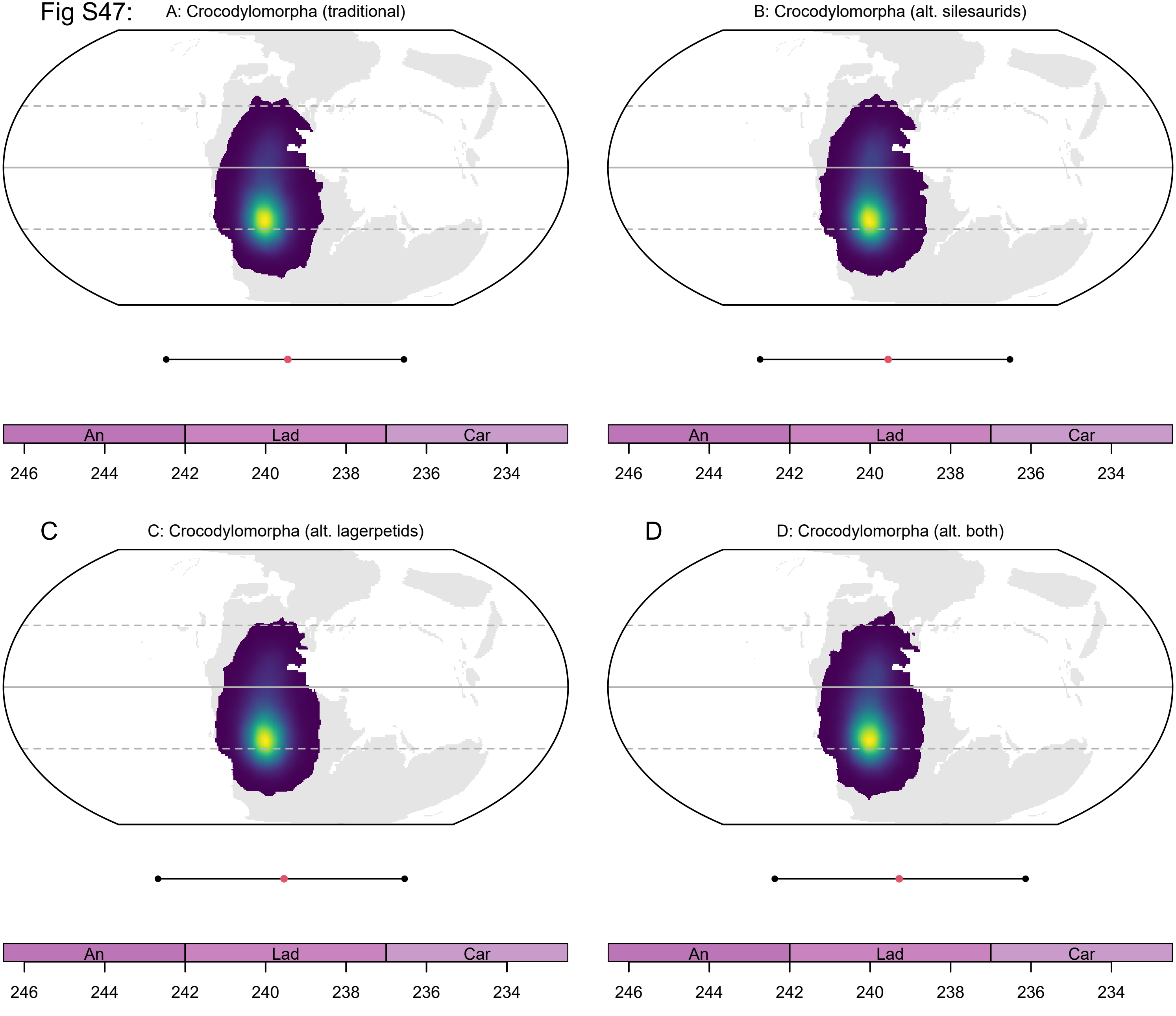
A: Crocodylomorpha (traditional)
B: Crocodylomorpha (alt. silesaurids)
C
C: Crocodylomorpha (alt. lagerpetids)
D
D: Crocodylomorpha (alt. both)
An
Lad
Car
246
244
242
240
238
236
234

Fig S48:

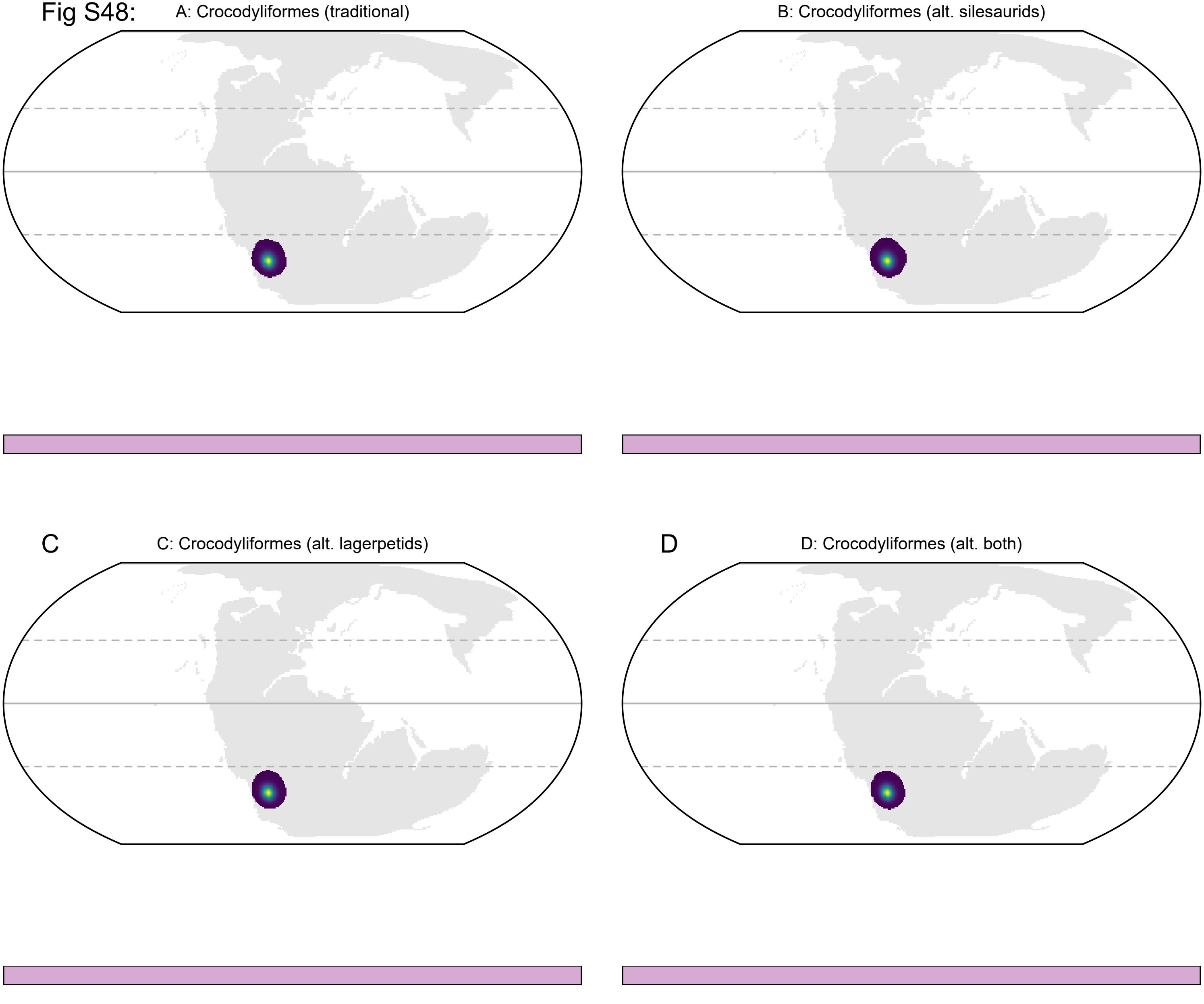

A: Crocodyliformes (traditional)
B: Crocodyliformes (alt. silesaurids)
C
C: Crocodyliformes (alt. lagerpetids)
D
D: Crocodyliformes (alt. both)

Fig S49:

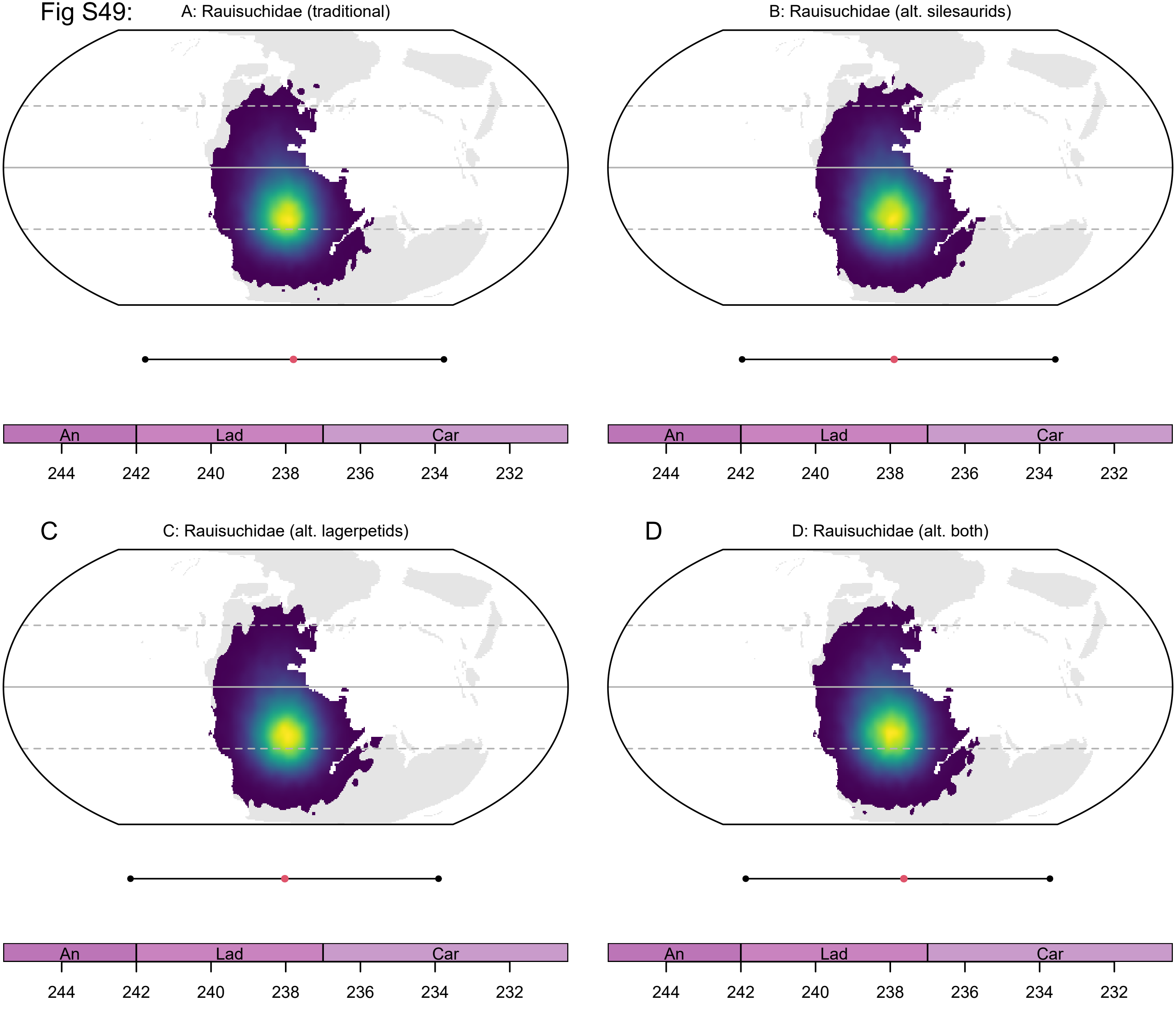
A: Raauisuchidae (traditional)
B: Rauisuchidae (alt. silesaurids)
C
C: Rauisuchidae (alt. lagerpetids)
D
D: Rauisuchidae (alt. both)
An
Lad
Car
244
242
240
238
236
234
232

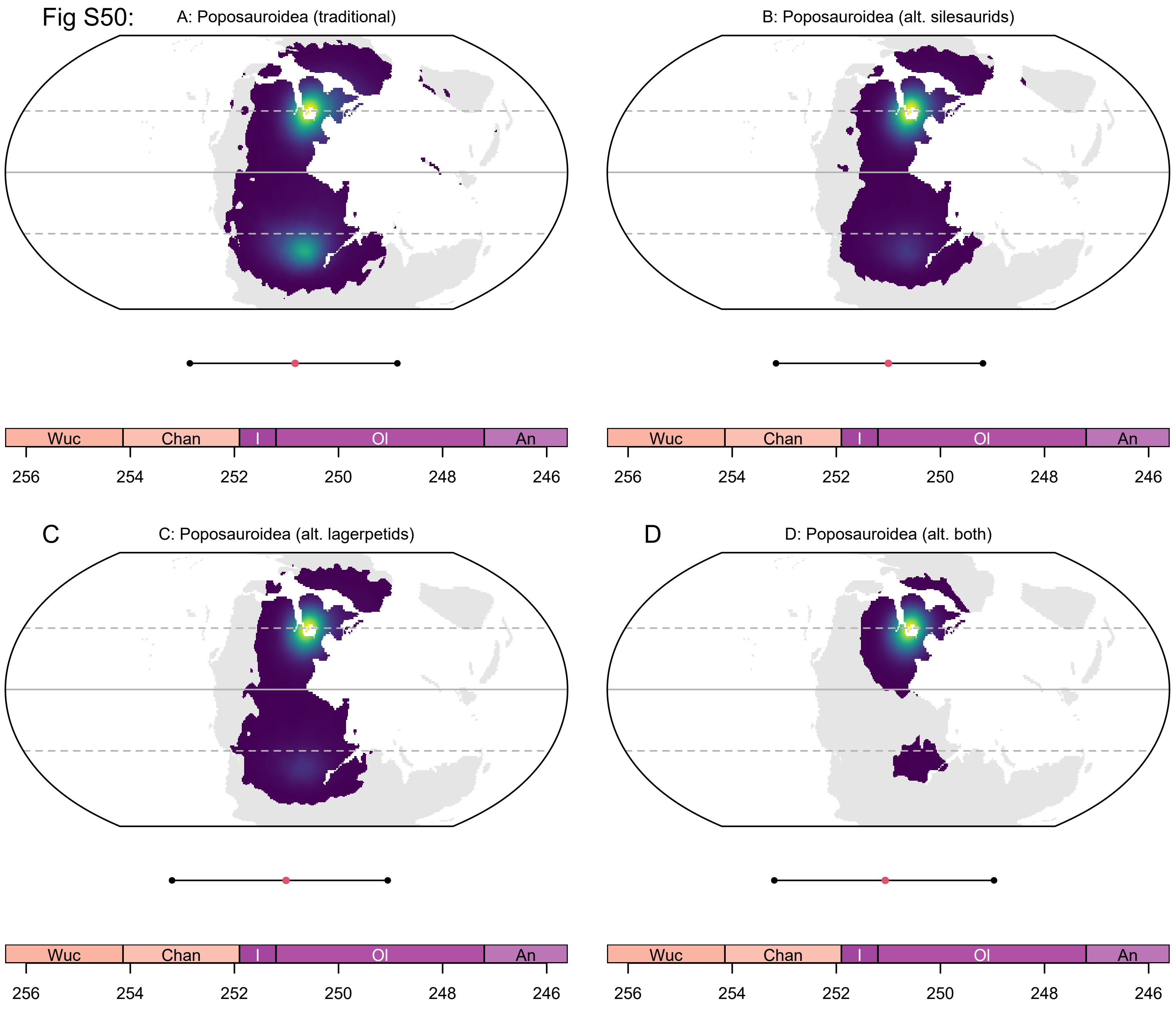
Fig S50:
A: Poposauroidea (traditional)
B: Poposauroidea (alt. silesaurids)
C
C: Poposauroidea (alt. lagerpetids)
D
D: Poposauroidea (alt. both)
Wuc
Chan
I
Ol
An
256
254
252
250
248
246

Fig S51:

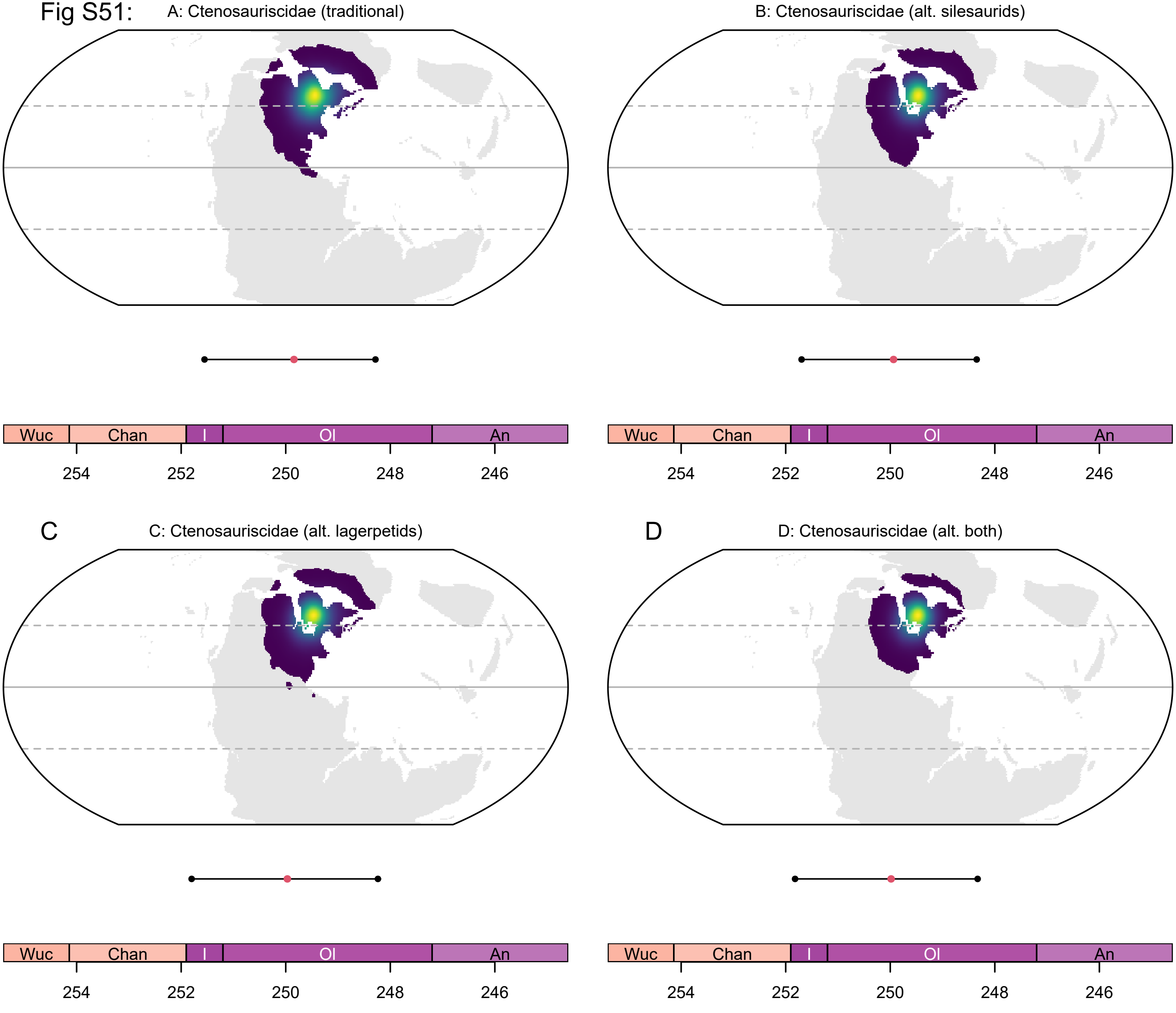

A: Ctenosauriscidae (traditional)
B: Ctenosauriscidae (alt. silesaurids)
C
C: Ctenosauriscidae (alt. lagerpetids)
D
D: Ctenosauriscidae (alt. both)
Wuc
Chan
I
Ol
An
254
252
250
248
246

Fig S52:

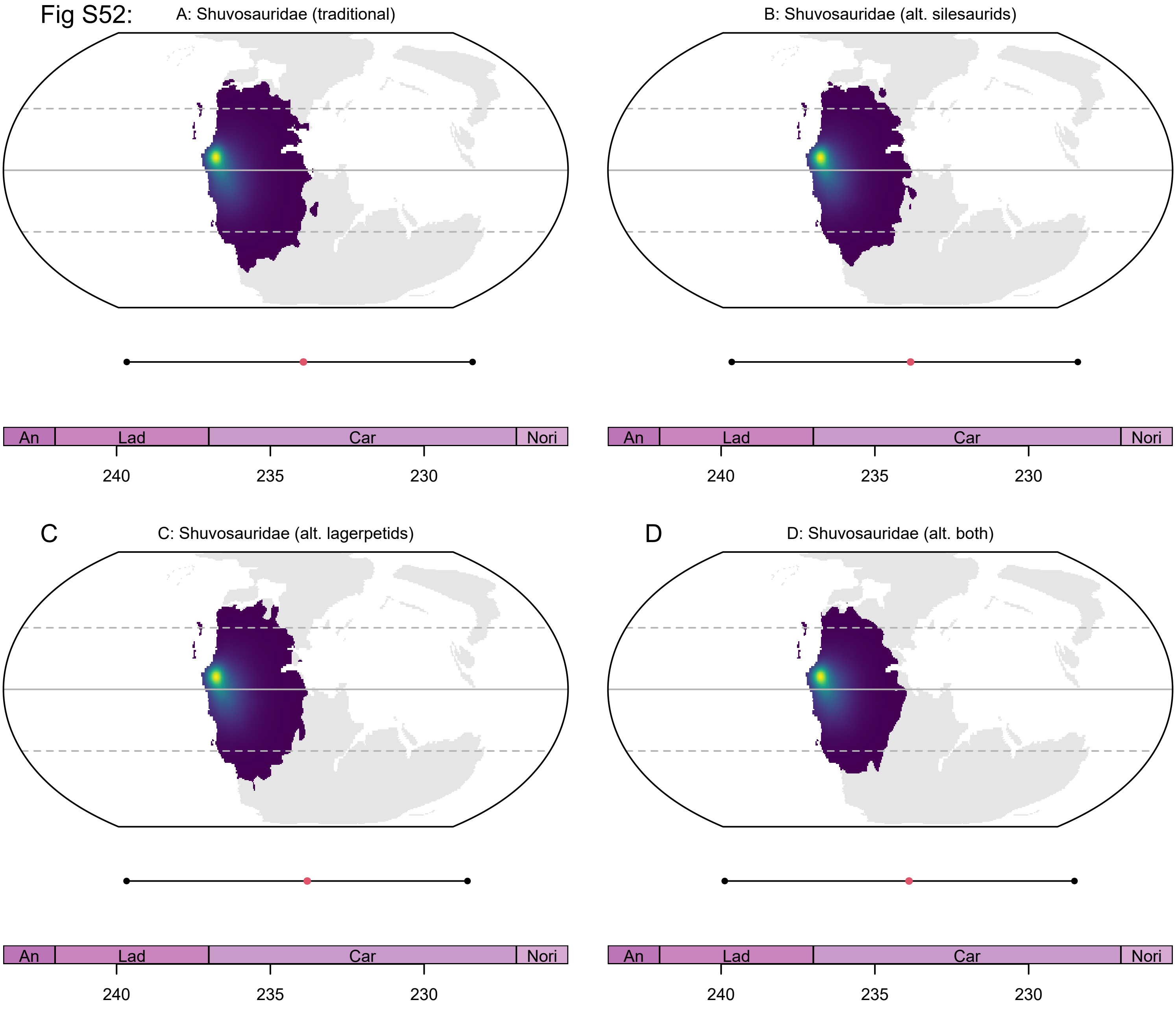
A: Shuvosauridae (traditional)
B: Shuvosauridae (alt. silesaurids)
C
C: Shuvosauridae (alt. lagerpetids)
D
D: Shuvosauridae (alt. both)
An
Lad
Car
Nori
240
235
230

Fig S53:

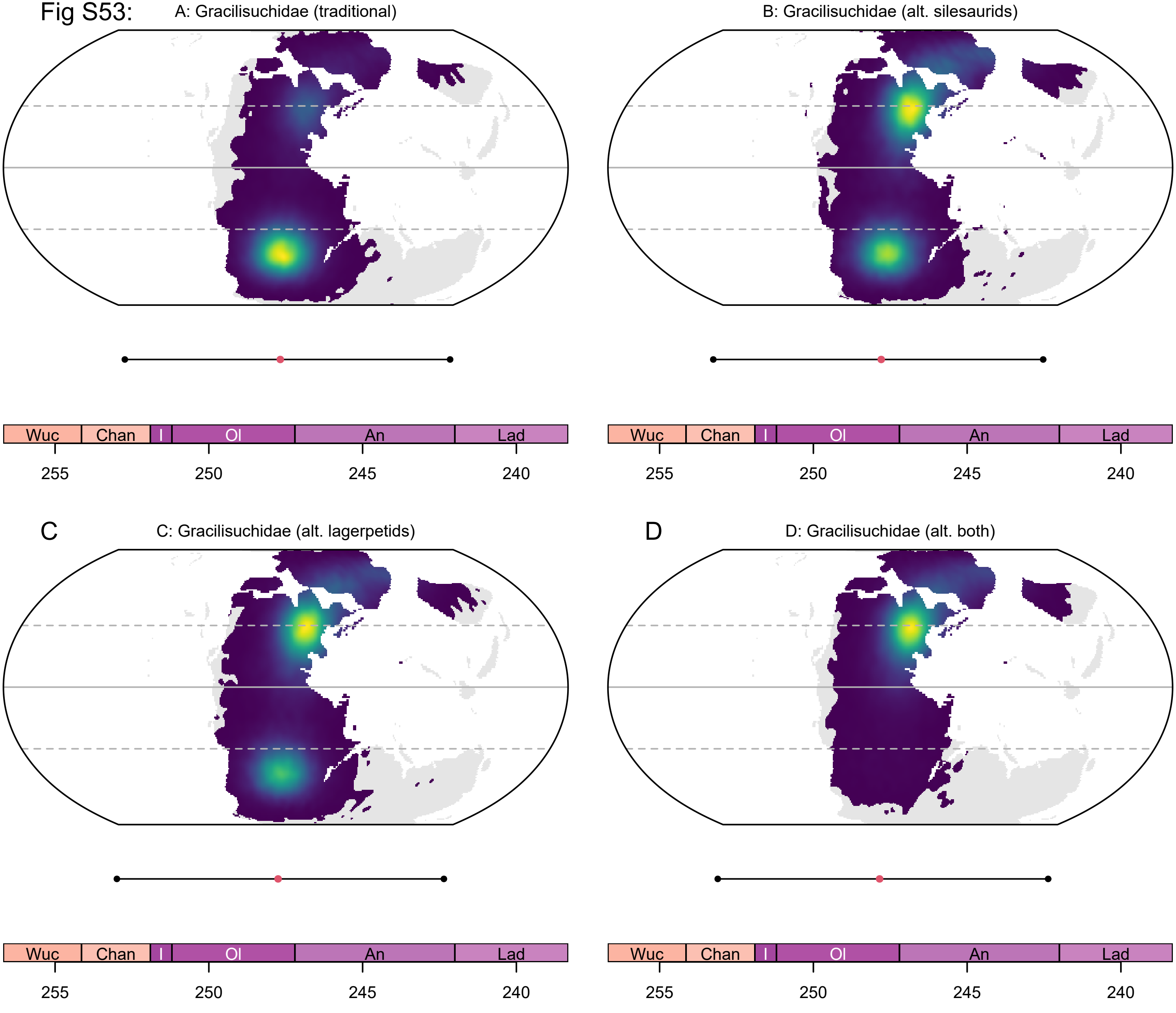
A: Gracilisuchidae (traditional)
B: Gracilisuchidae (alt. silesaurids)
C
C: Gracilisuchidae (alt. lagerpetids)
D
D: Gracilisuchidae (alt. both)
Wuc
Chan
I
Ol
An
Lad
255
250
245
240

Fig S54:

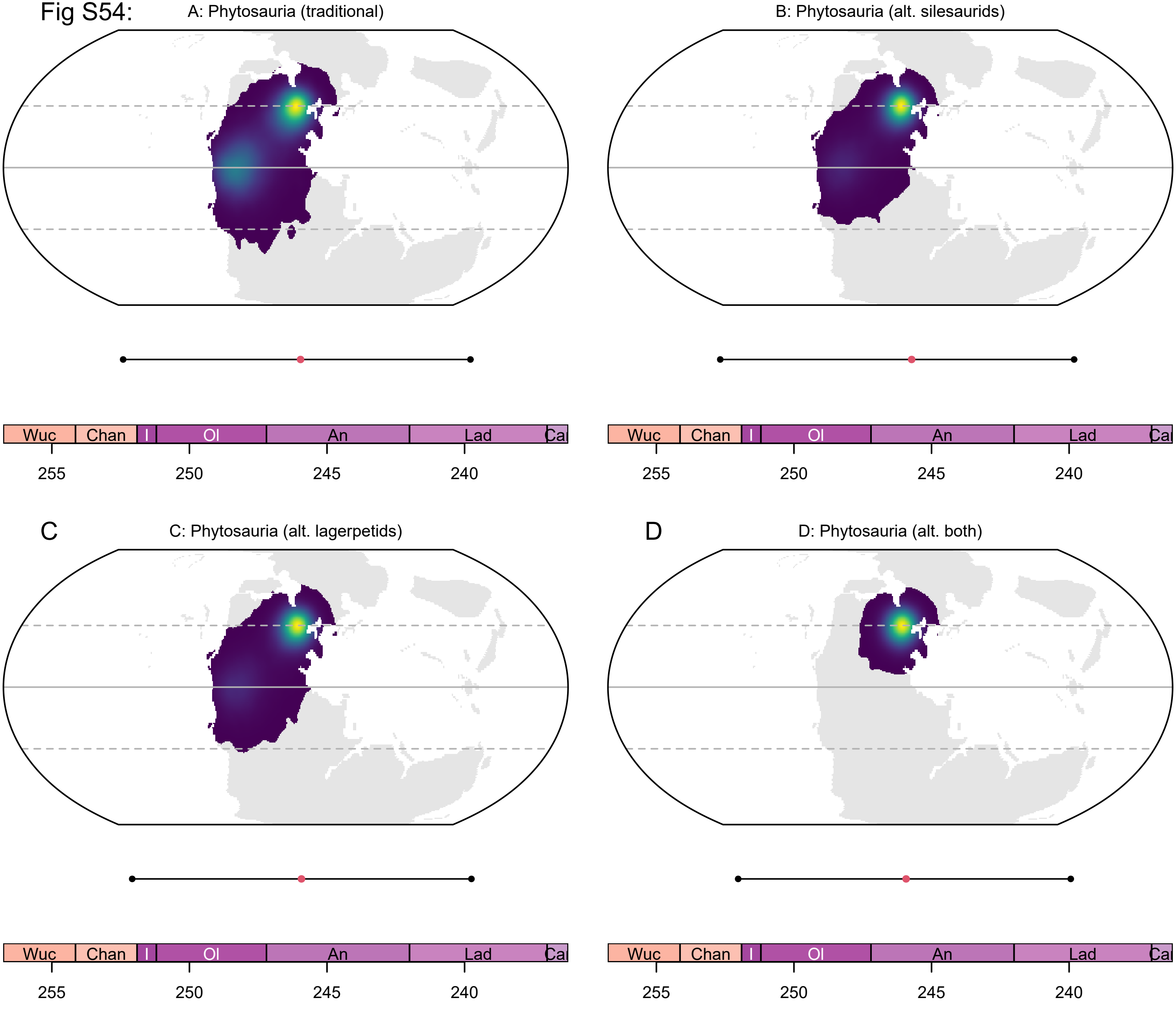

A: Phytosauria (traditional)
B: Phytosauria (alt. silesaurids)
C
C: Phytosauria (alt. lagerpetids)
D
D: Phytosauria (alt. both)
Wuc
Chan
I
Ol
An
Lad
Ca
255
250
245
240

Fig S55:

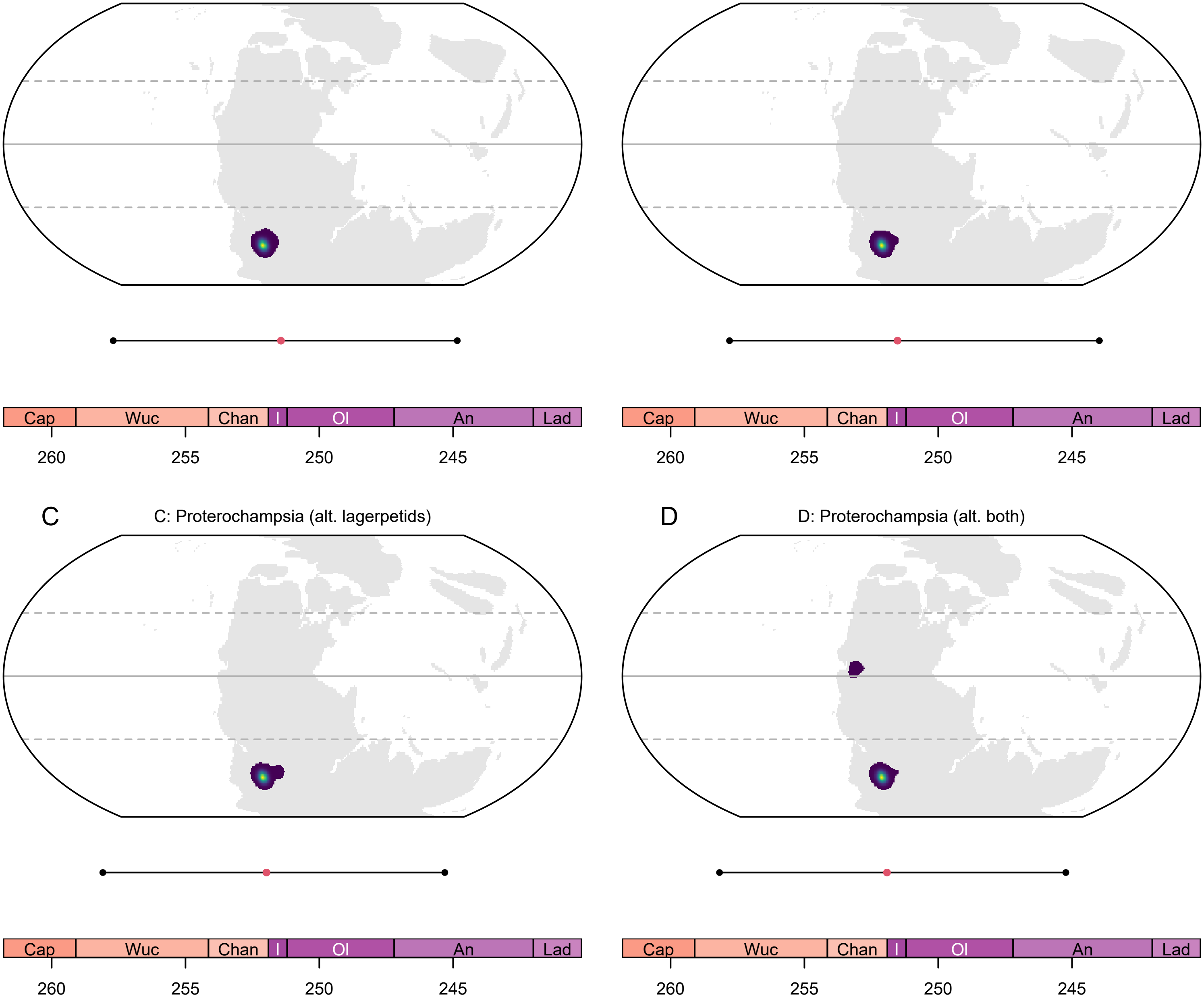
A: Proterochampsia (traditional)
B: Proterochampsia (alt. silesaurids)
C
C: Proterochampsia (alt. lagerpetids)
D
D: Proterochampsia (alt. both)
Cap
Wuc
Chan
I
Ol
An
Lad
260
255
250
245

Fig S56:

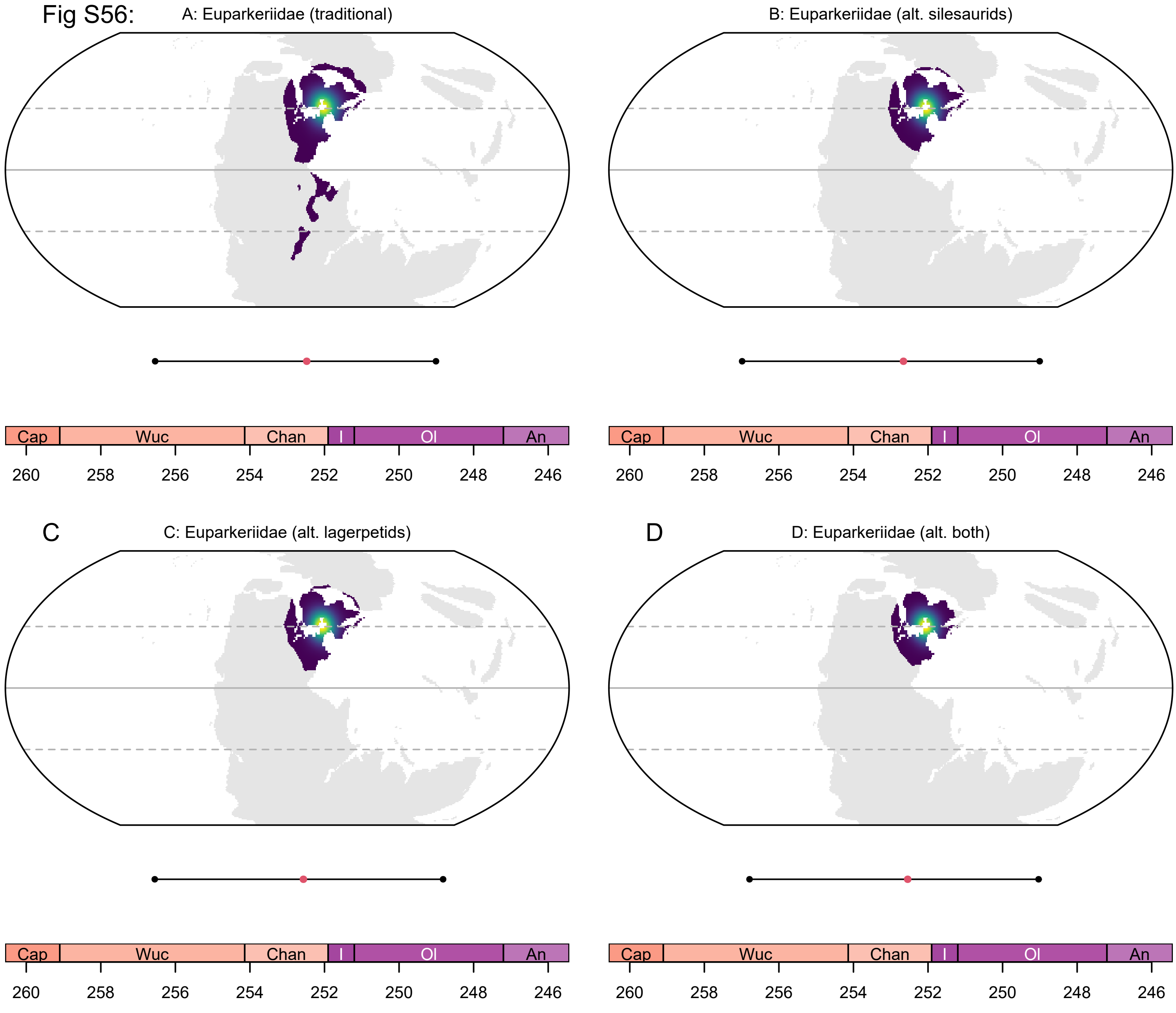
A: Euparkeriidae (traditional)
B: Euparkeriidae (alt. silesaurids)
C
C: Euparkeriidae (alt. lagerpetids)
D
D: Euparkeriidae (alt. both)
Cap
Wuc
Chan
I
Ol
An
260
258
256
254
252
250
248
246

Fig S57:

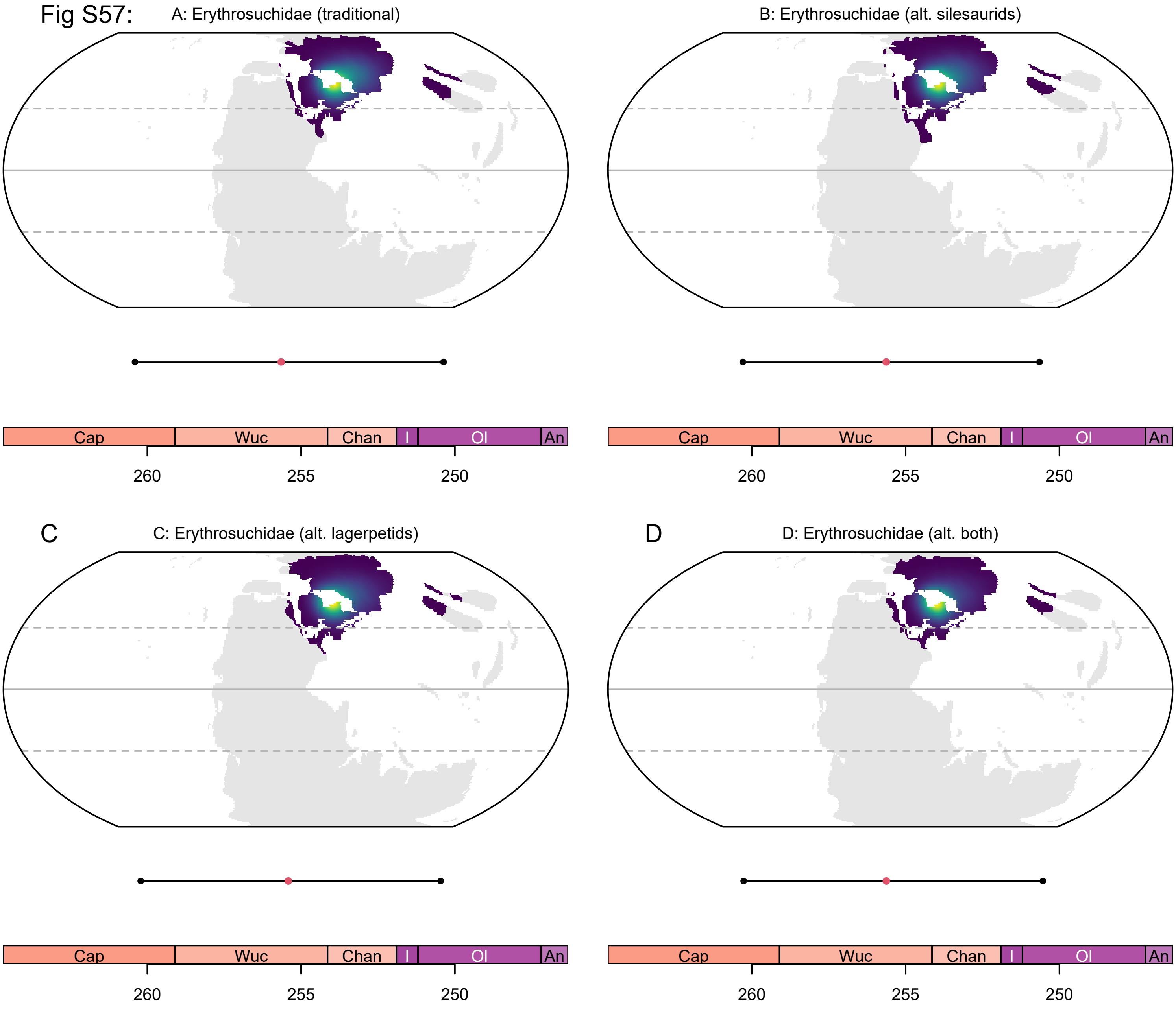
A: Erythrosuchidae (traditional)
B: Erythrosuchidae (alt. silesaurids)
C
C: Erythrosuchidae (alt. lagerpetids)
D
D: Erythrosuchidae (alt. both)
Cap
Wuc
Chan
I
Ol
An
260
255
250

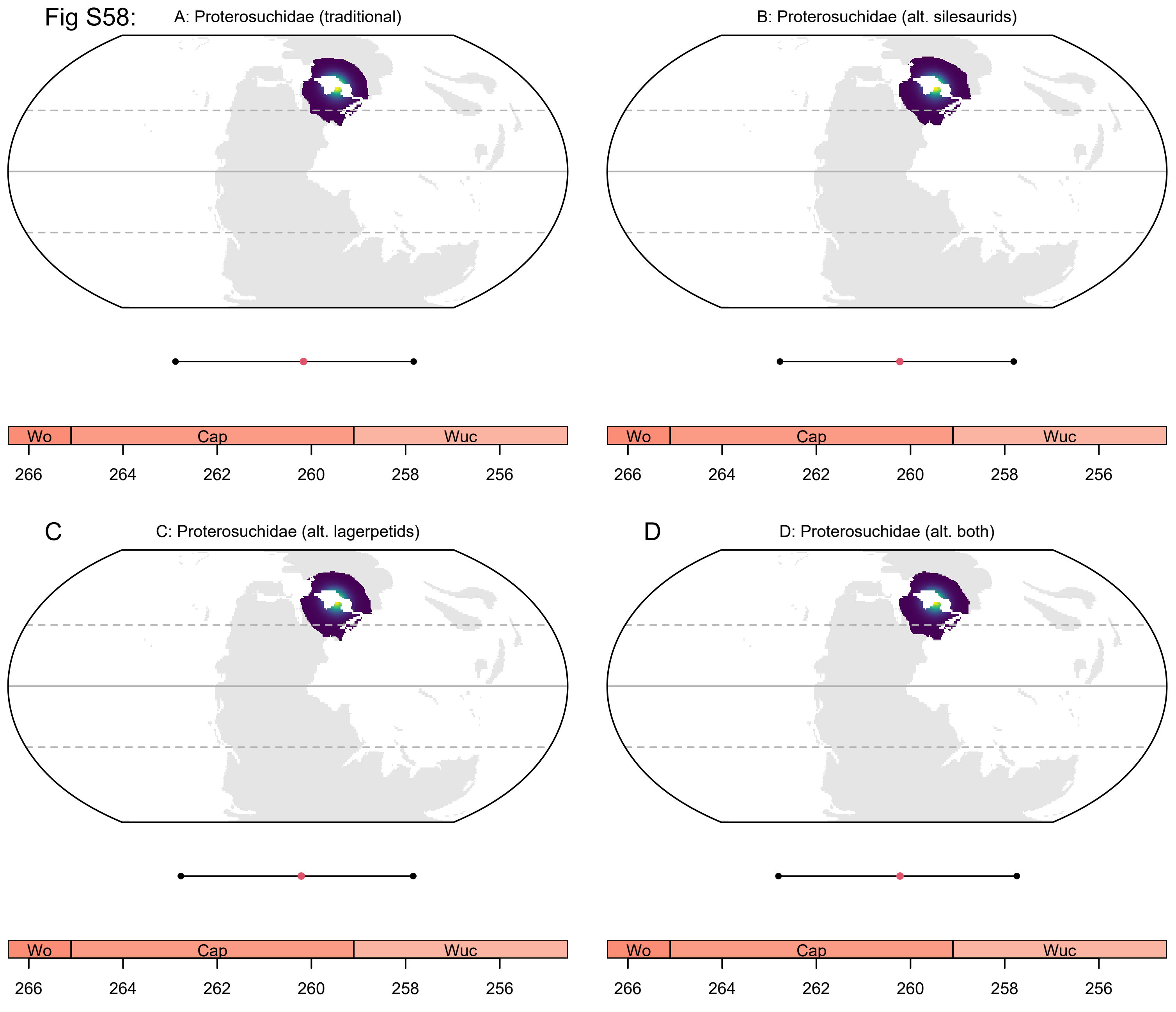

Fig S58:
A: Proterosuchidae (traditional)
B: Proterosuchidae (alt. silesaurids)
C
C: Proterosuchidae (alt. lagerpetids)
D
D: Proterosuchidae (alt. both)
Wo
Cap
Wuc
266
264
262
260
258
256

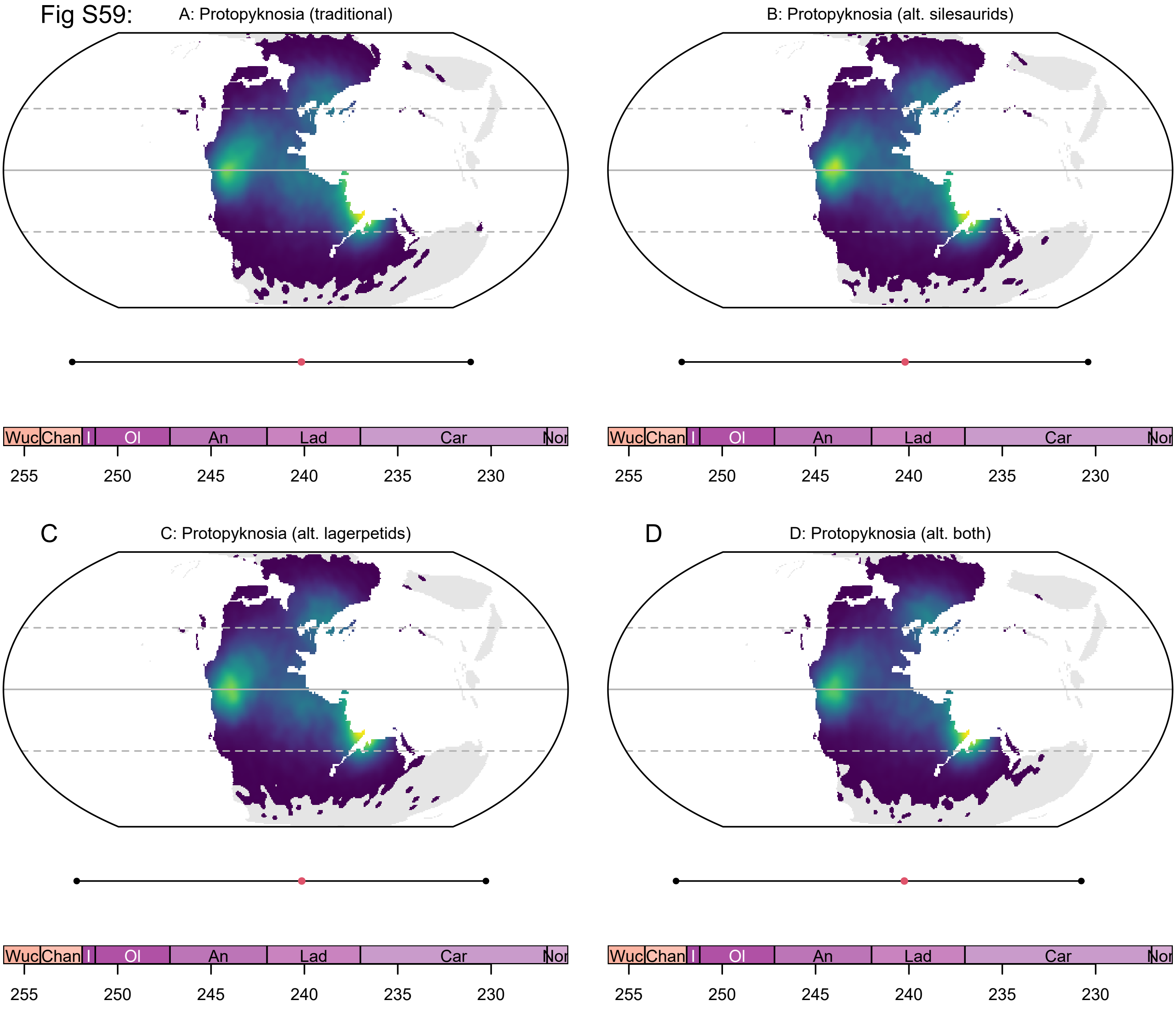
Fig S59:
A: Protopyknosia (traditional)
Wuc
Chan
I
Ol
An
Lad
Car
Nor
255
250
245
240
235
230
B: Protopyknosia (alt. silesaurids)
Wuc
Chan
I
Ol
An
Lad
Car
Nor
255
250
245
240
235
230
C
C: Protopyknosia (alt. lagerpetids)
Wuc
Chan
I
Ol
An
Lad
Car
Nor
255
250
245
240
235
230
D
D: Protopyknosia (alt. both)
Wuc
Chan
I
Ol
An
Lad
Car
Nor
255
250
245
240
235
230

Fig S60:

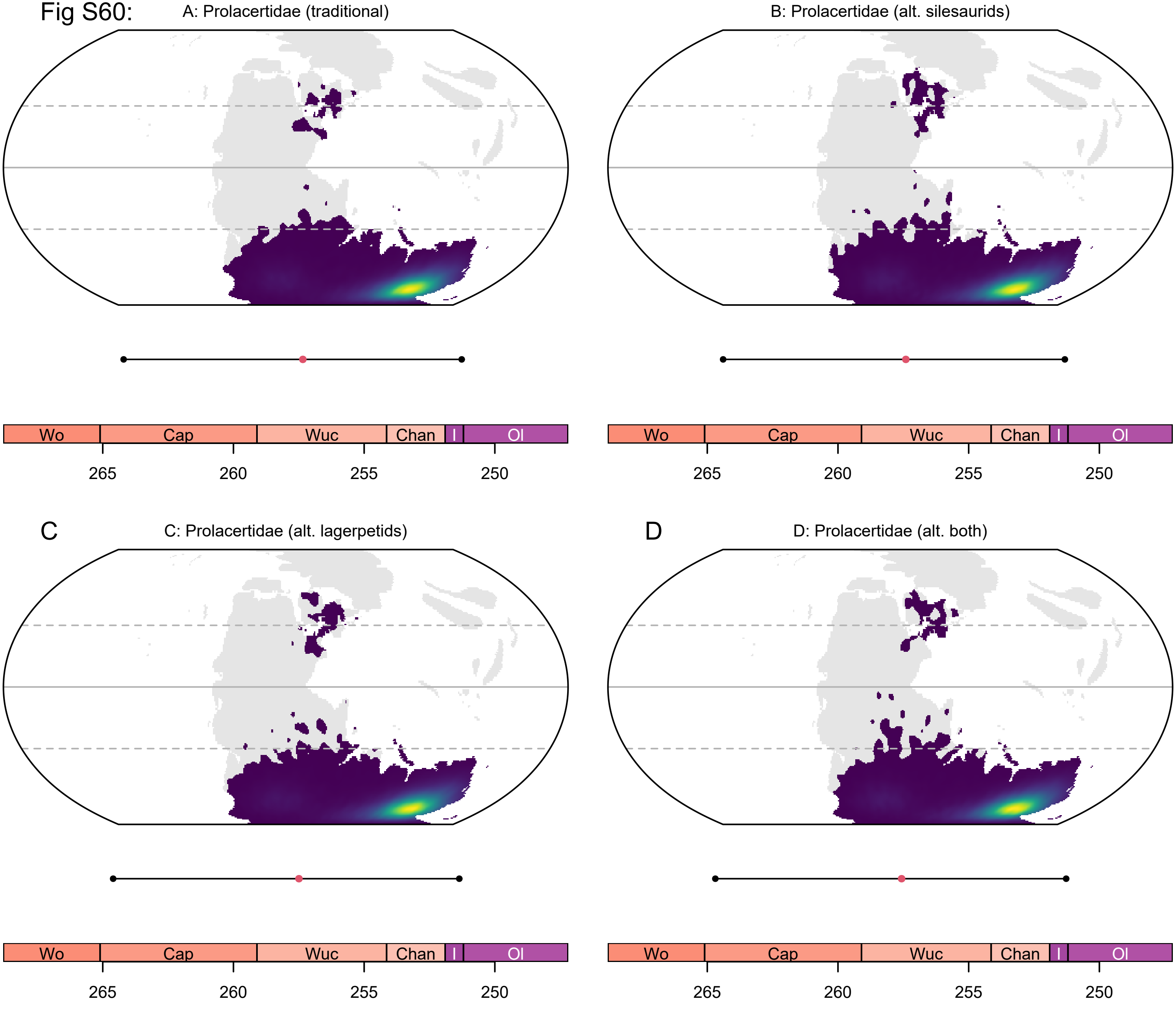
A: Prolacertidae (traditional)
B: Prolacertidae (alt. silesaurids)
C
C: Prolacertidae (alt. lagerpetids)
D
D: Prolacertidae (alt. both)
Wo
Cap
Wuc
Chan
I
Ol
265
260
255
250

Fig S61:

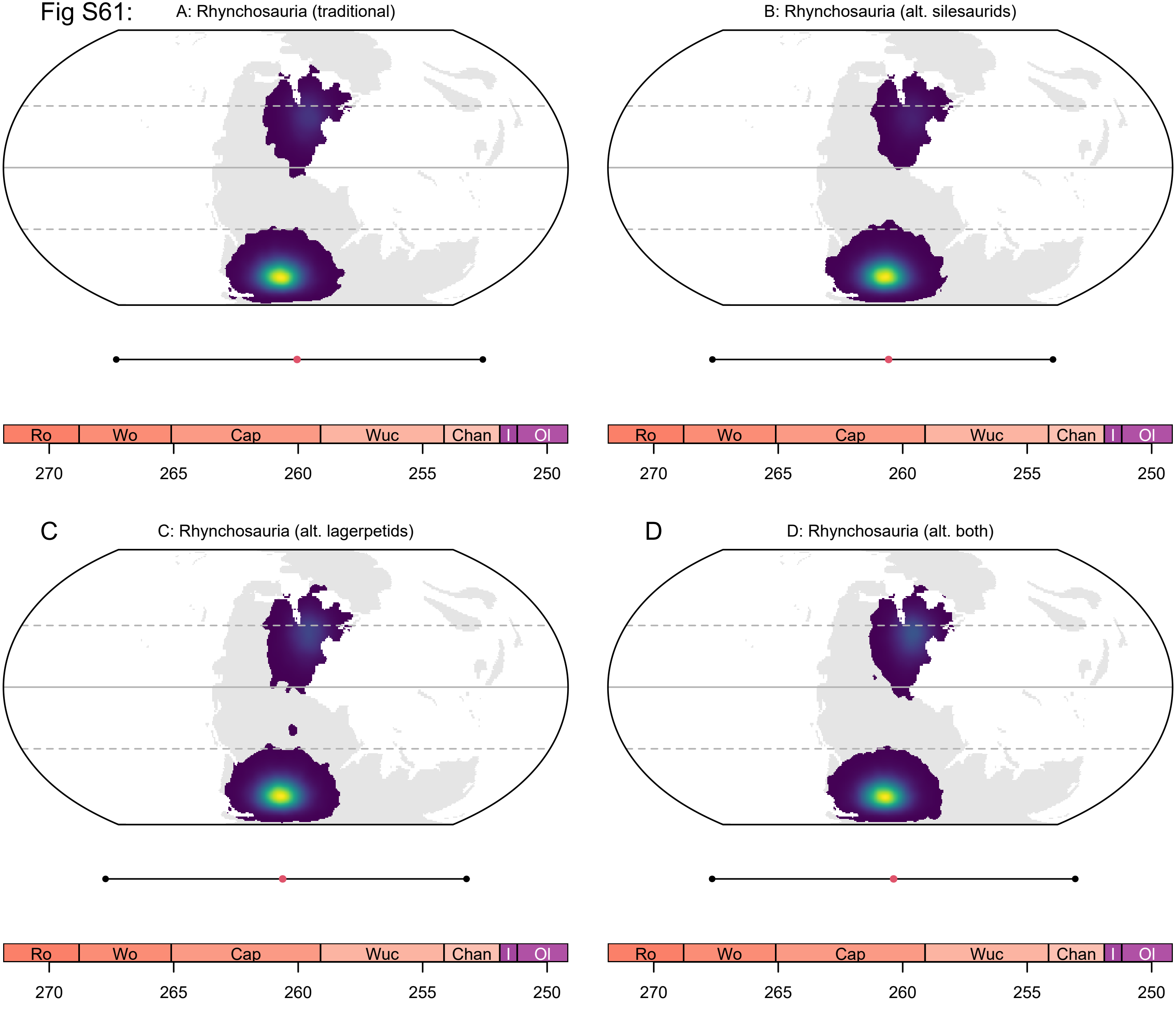

A: Rhynchosauria (traditional)
B: Rhynchosauria (alt. silesaurids)
C
C: Rhynchosauria (alt. lagerpetids)
D
D: Rhynchosauria (alt. both)
Ro
Wo
Cap
Wuc
Chan
I
Ol
270
265
260
255
250

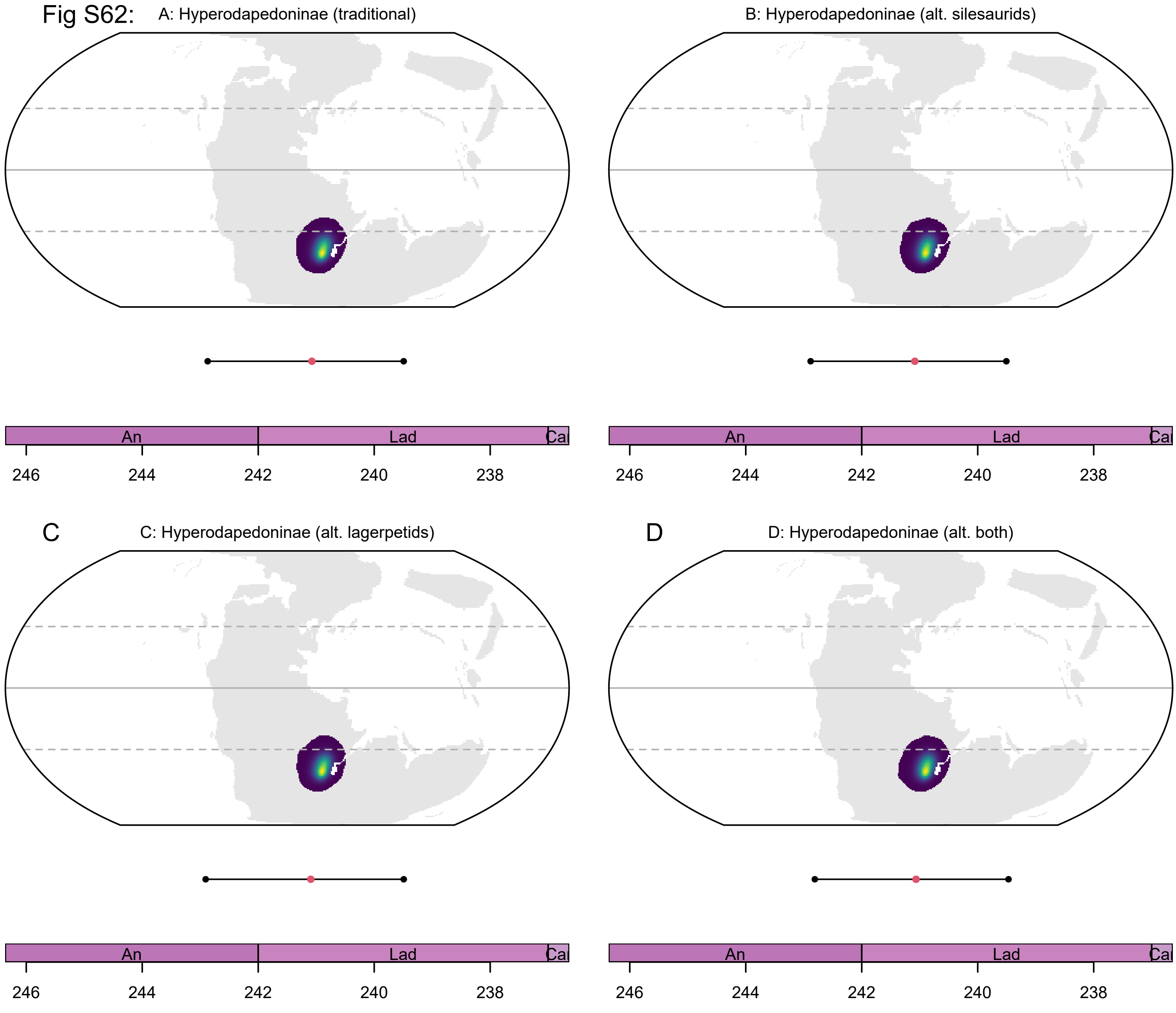
A: Hyperodapedoninae (traditional)
B: Hyperodapedoninae (alt. silesaurids)
C
C: Hyperodapedoninae (alt. lagerpetids)
D
D: Hyperodapedoninae (alt. both)
An
Lad
Car
246
244
242
240
238

Fig S62:

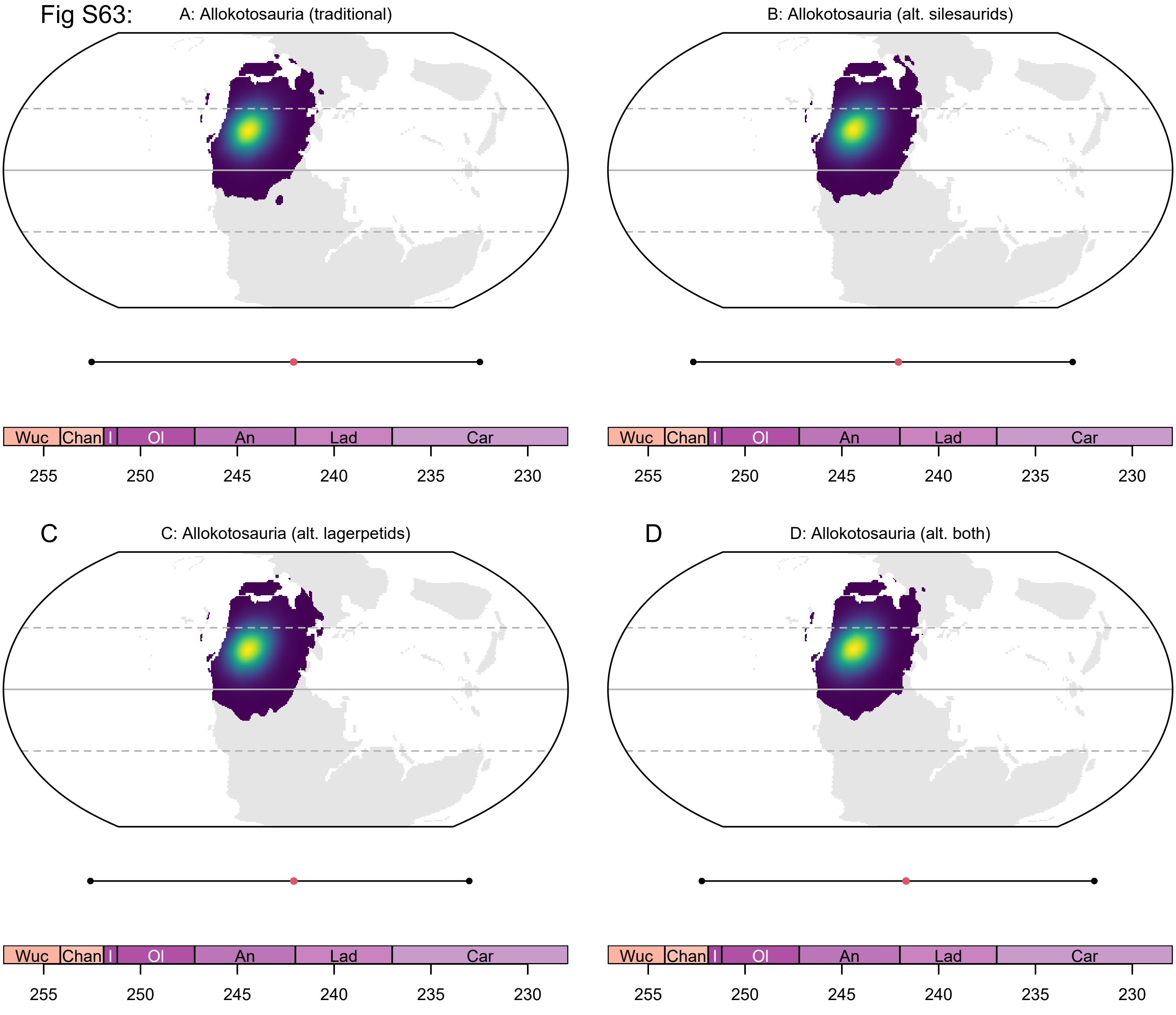
Fig S63:
A: Allokotosauria (traditional)
B: Allokotosauria (alt. silesaurids)
C
C: Allokotosauria (alt. lagerpetids)
D
D: Allokotosauria (alt. both)
Wuc
Chan
I
Ol
An
Lad
Car
255
250
245
240
235
230

Fig S64:

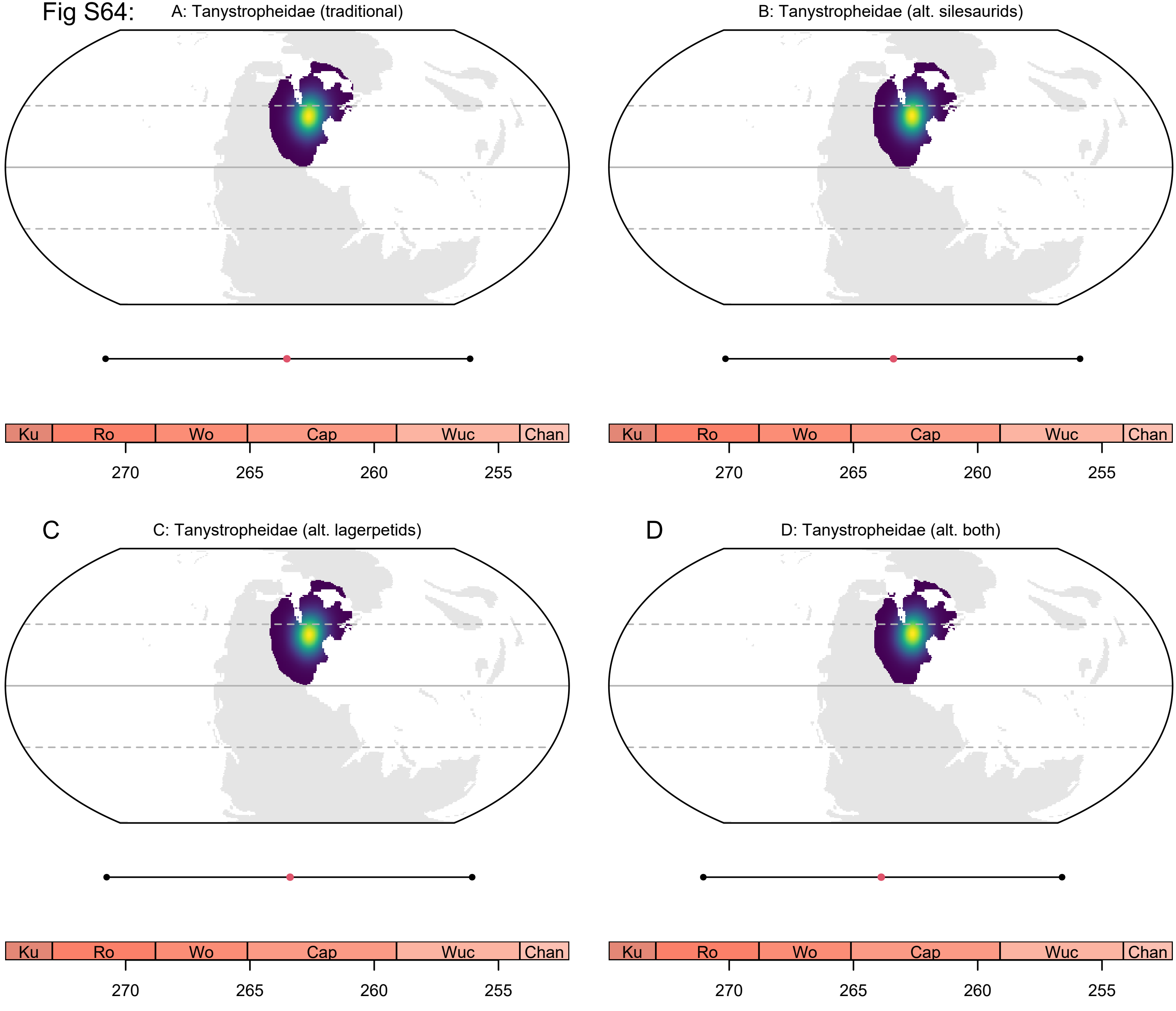
A: Tanystropheidae (traditional)
B: Tanystropheidae (alt. silesaurids)
C
C: Tanystropheidae (alt. lagerpetids)
D
D: Tanystropheidae (alt. both)
Ku
Ro
Wo
Cap
Wuc
Chan
270
265
260
255

Fig S65:

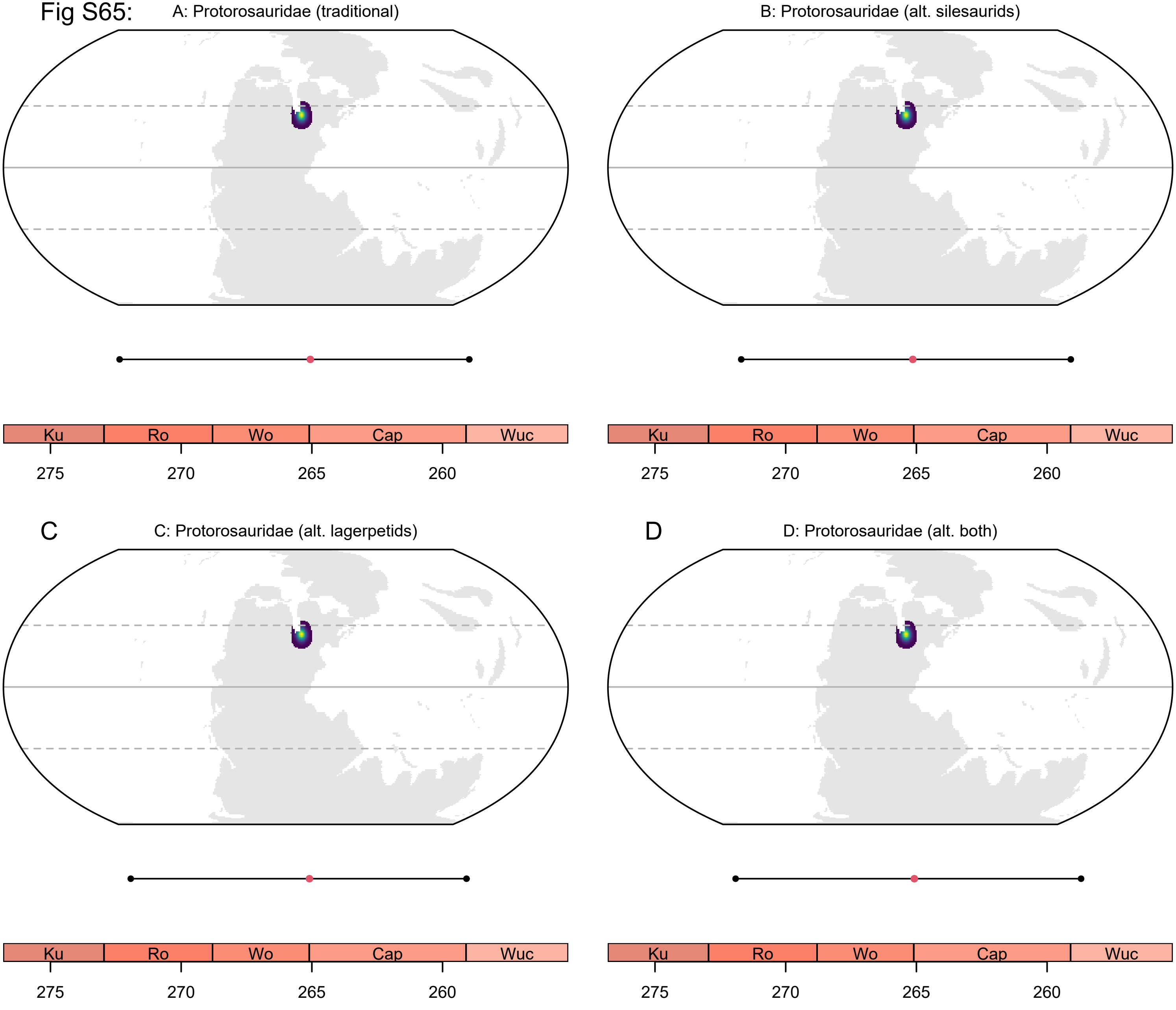

A: Protorosauridae (traditional)
B: Protorosauridae (alt. silesaurids)
C
C: Protorosauridae (alt. lagerpetids)
D
D: Protorosauridae (alt. both)
Ku
Ro
Wo
Cap
Wuc
275
270
265
260

Fig. S1: Kungurian

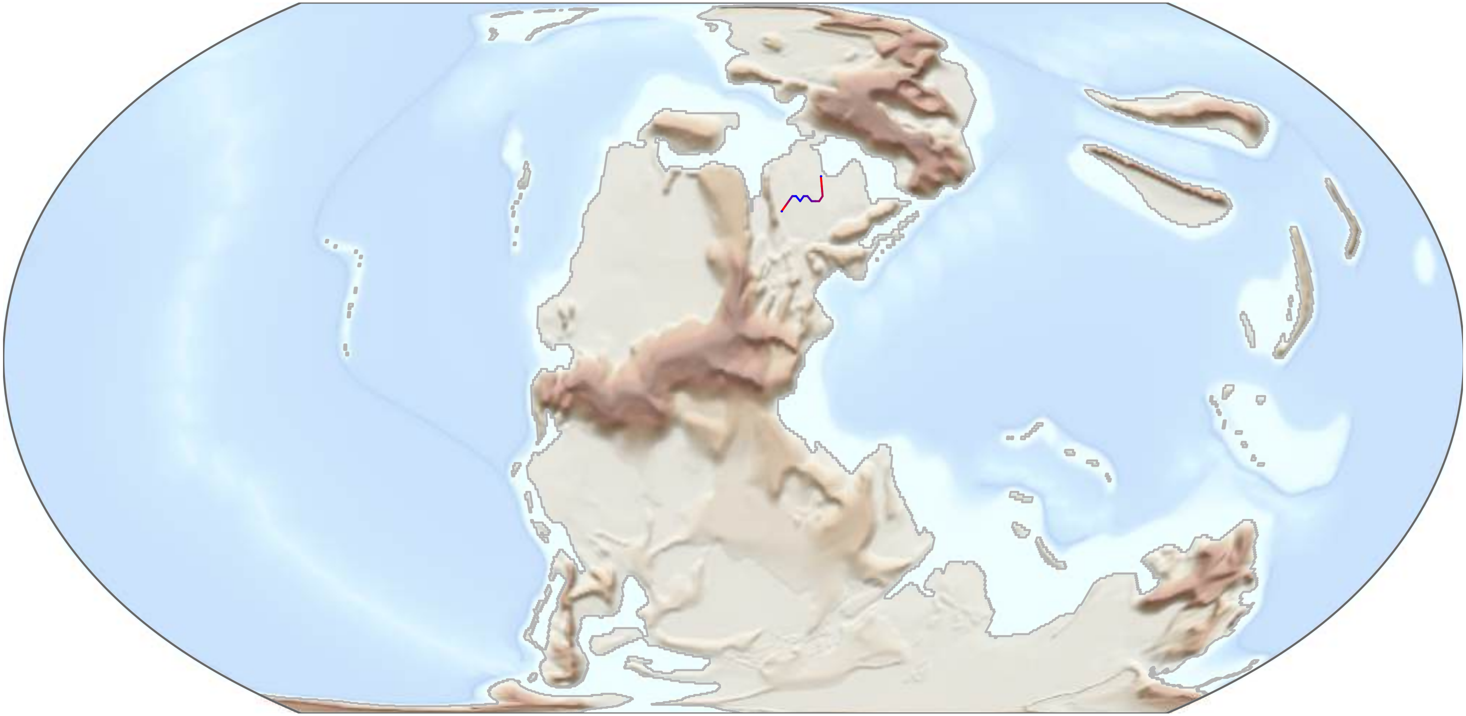

Phylogeographic dispersal paths run gradually from blue to red.

Paths may begin at the terminus of an ancestral path (immediate red to blue jump)

or share a common starting point (blue central region bounded by red ends

Fig. S2: Roadian

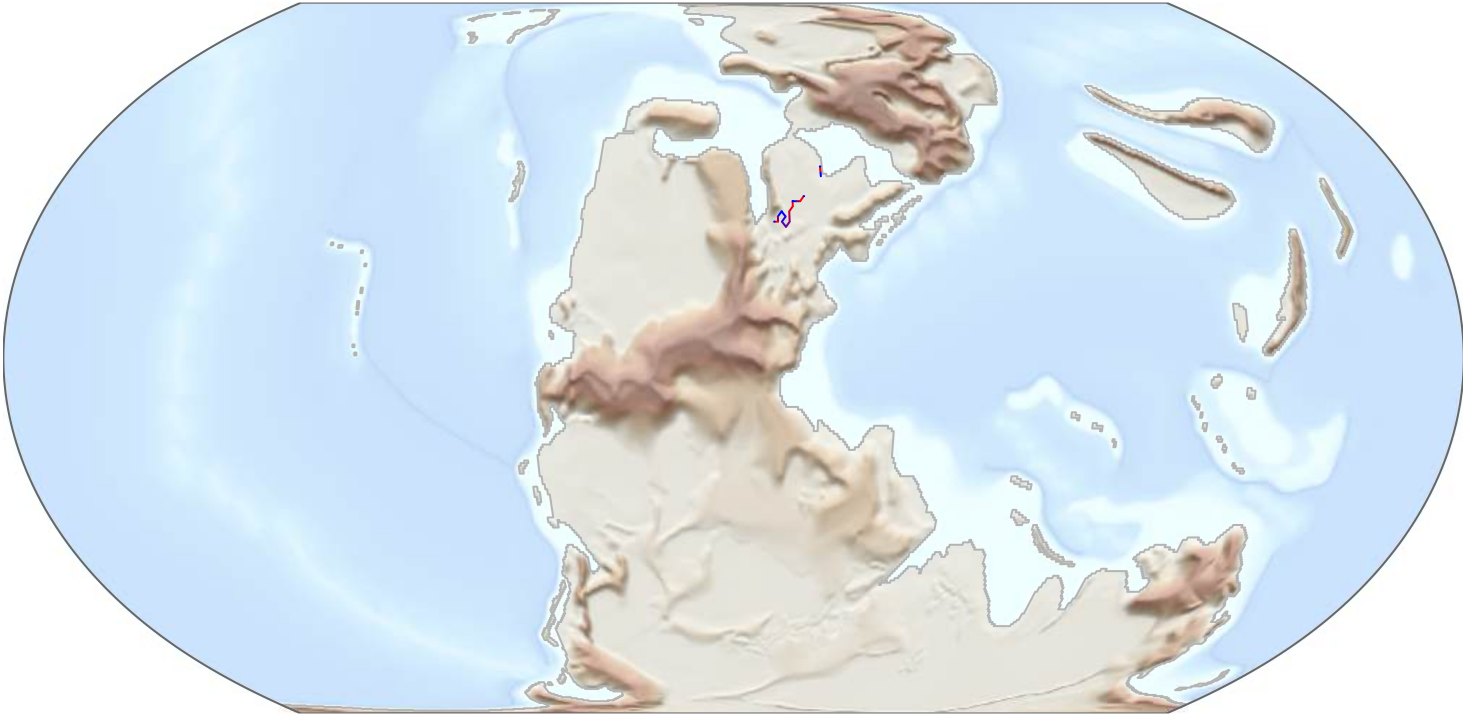

Phylogeographic dispersal paths run gradually from blue to red.

Paths may begin at the terminus of an ancestral path (immediate red to blue jump)

or share a common starting point (blue central region bounded by red ends

Fig. S3: Wordian

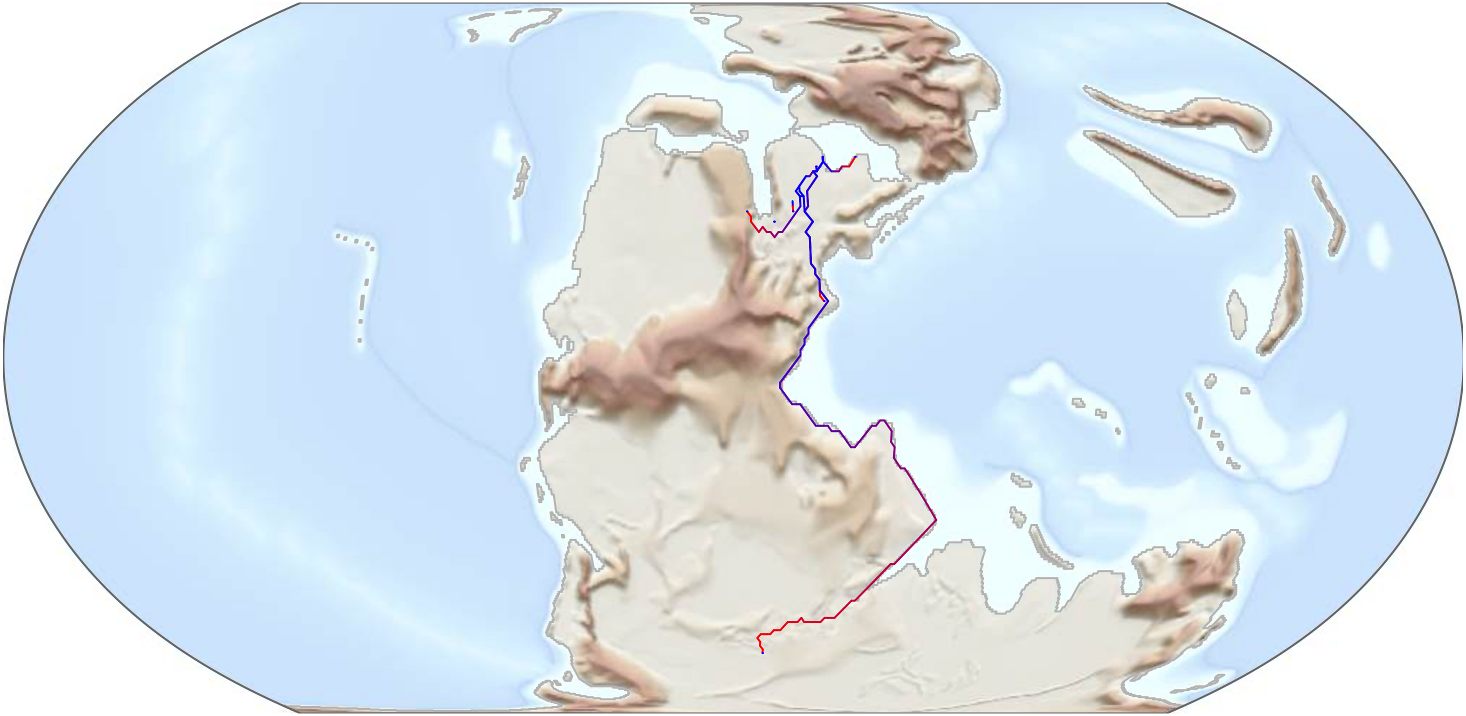

Phylogeographic dispersal paths run gradually from blue to red.

Paths may begin at the terminus of an ancestral path (immediate red to blue jump)

or share a common starting point (blue central region bounded by red ends

Fig. S4: Capitanian

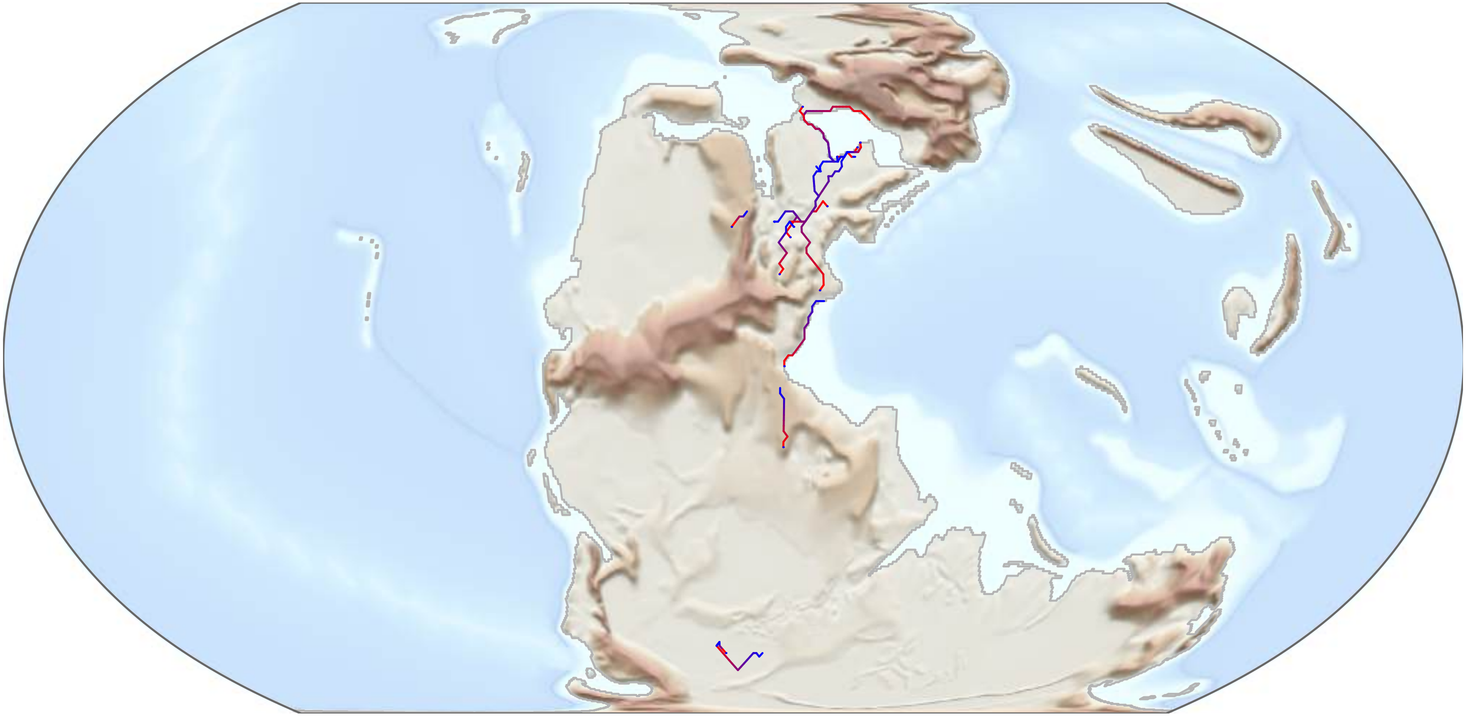

Phylogeographic dispersal paths run gradually from blue to red.

Paths may begin at the terminus of an ancestral path (immediate red to blue jump)

or share a common starting point (blue central region bounded by red ends

Fig. S5: Wuchiapingian

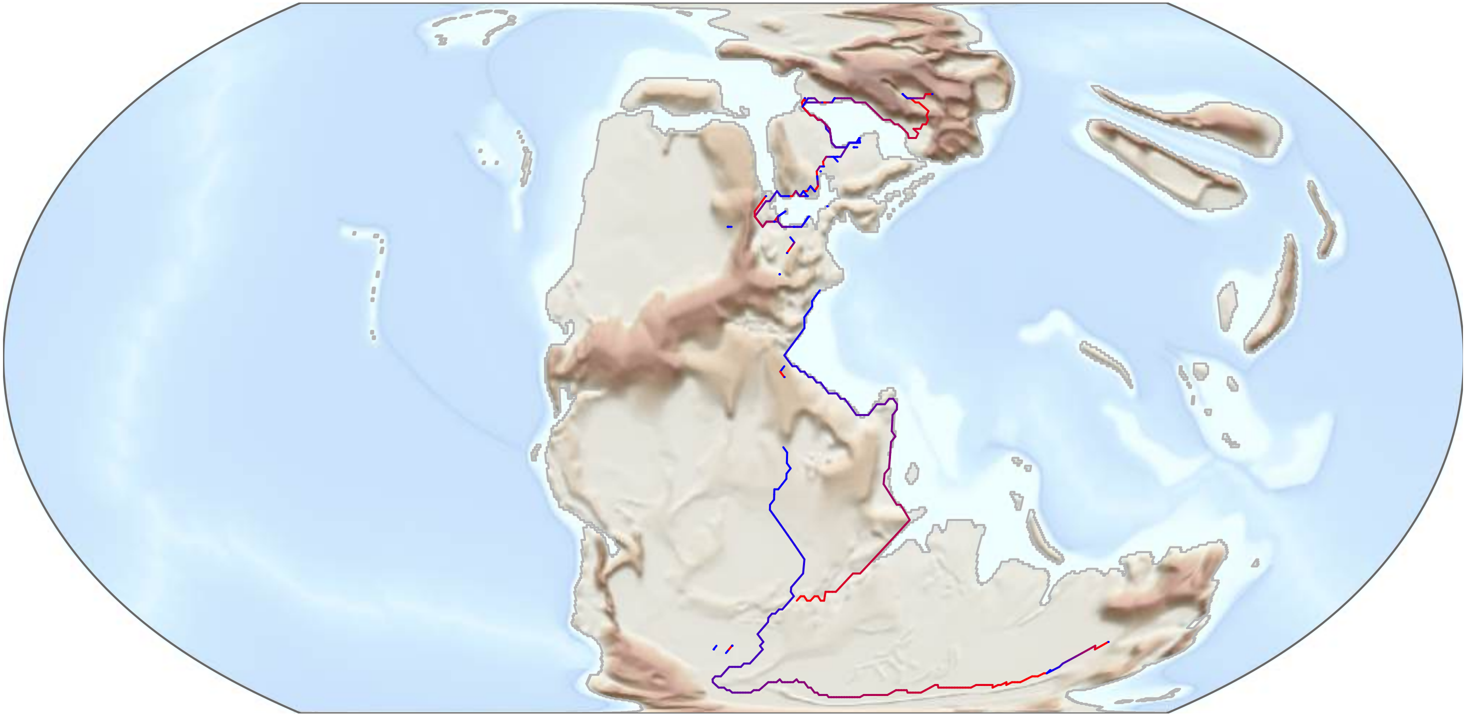

Phylogeographic dispersal paths run gradually from blue to red.

Paths may begin at the terminus of an ancestral path (immediate red to blue jump)

or share a common starting point (blue central region bounded by red ends

Fig. S6: Changhsingian

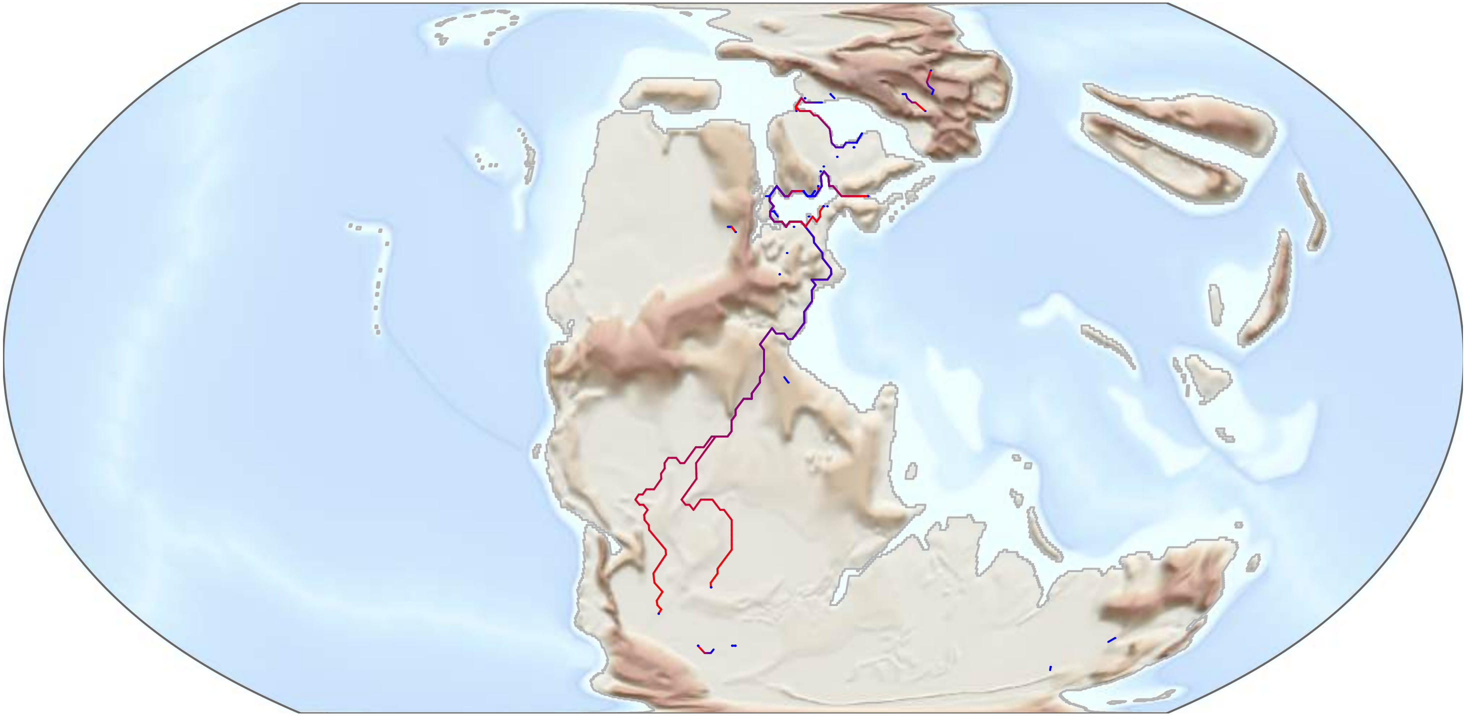

Phylogeographic dispersal paths run gradually from blue to red.

Paths may begin at the terminus of an ancestral path (immediate red to blue jump)

or share a common starting point (blue central region bounded by red ends

Fig. S7: Induan

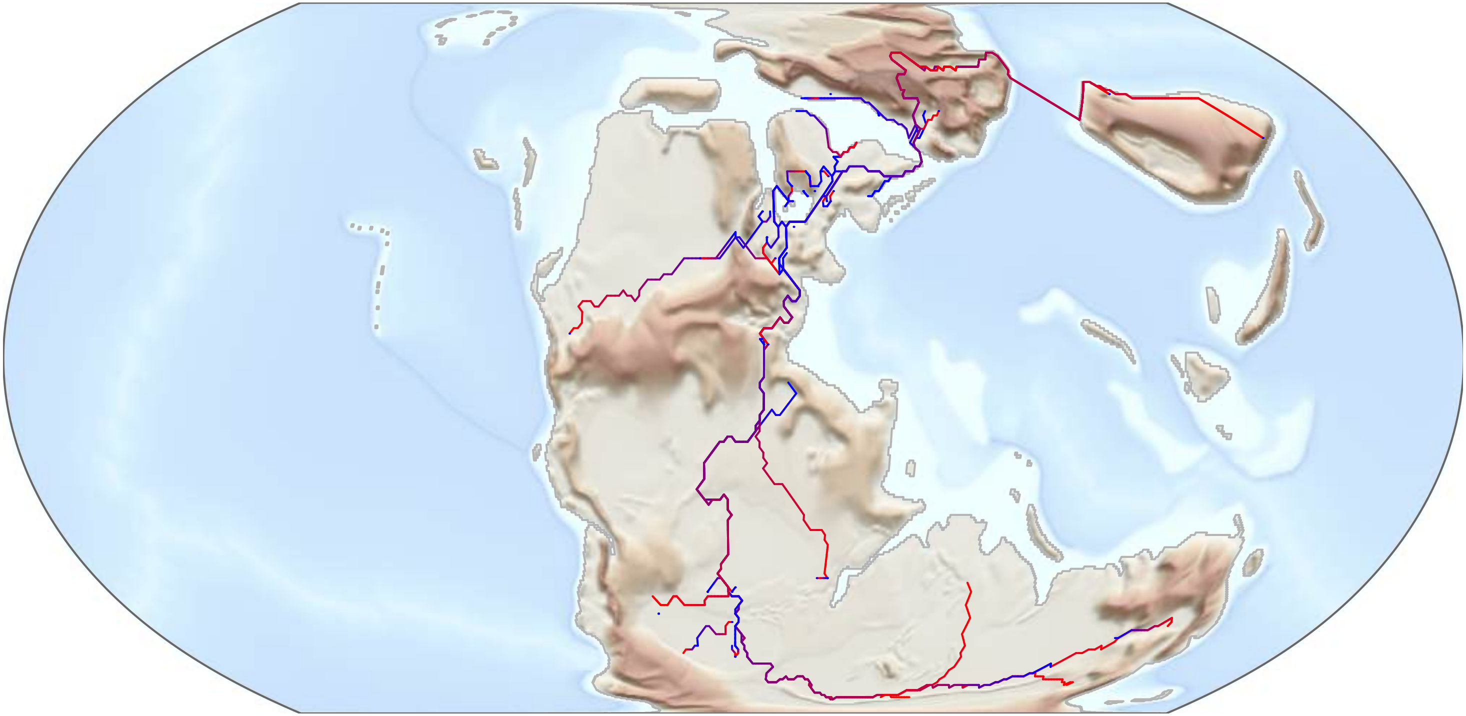

Phylogeographic dispersal paths run gradually from blue to red.

Paths may begin at the terminus of an ancestral path (immediate red to blue jump)

or share a common starting point (blue central region bounded by red ends

Fig. S8: Olenekian

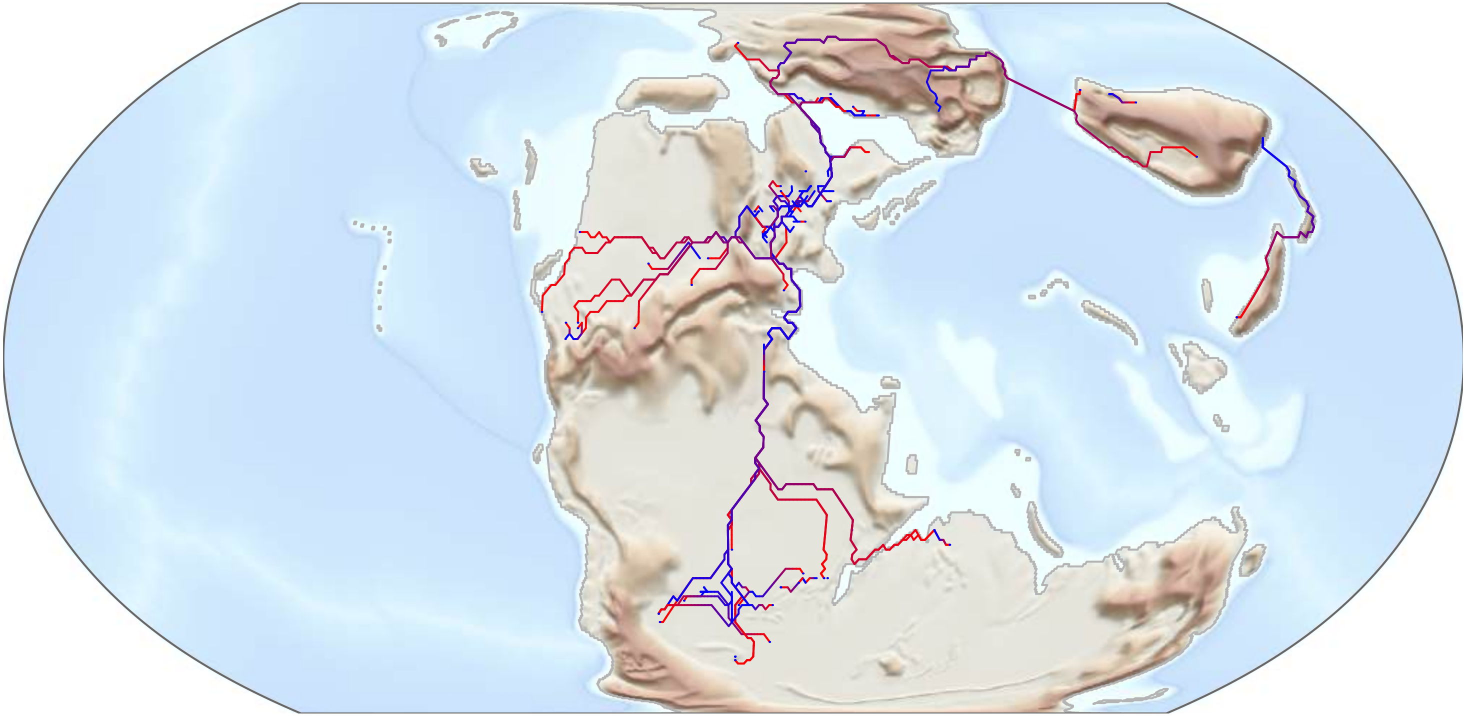

Phylogeographic dispersal paths run gradually from blue to red.

Paths may begin at the terminus of an ancestral path (immediate red to blue jump)

or share a common starting point (blue central region bounded by red ends

Fig. S9: Anisian

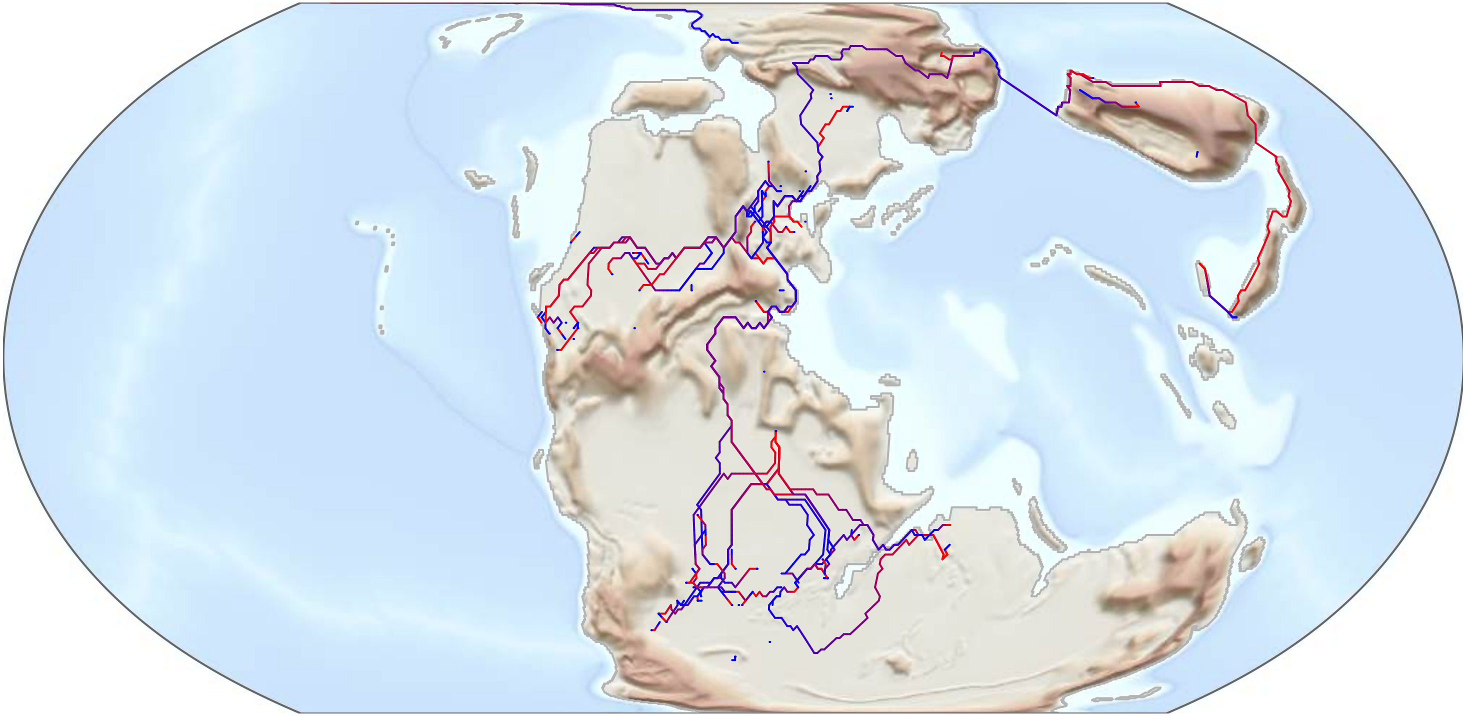

Phylogeographic dispersal paths run gradually from blue to red.

Paths may begin at the terminus of an ancestral path (immediate red to blue jump)

or share a common starting point (blue central region bounded by red ends

Fig. S10: Ladinian

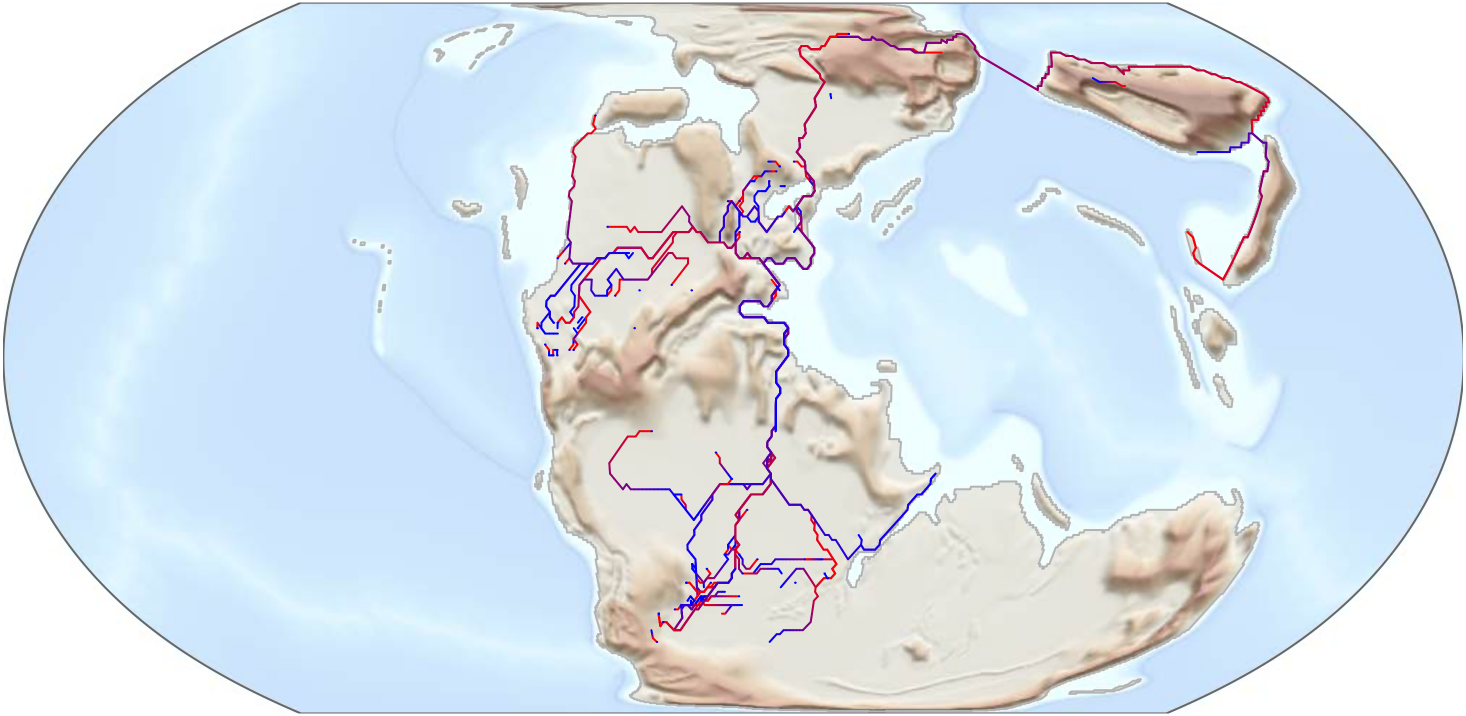

Phylogeographic dispersal paths run gradually from blue to red.

Paths may begin at the terminus of an ancestral path (immediate red to blue jump)

or share a common starting point (blue central region bounded by red ends

Fig. S11: Julian

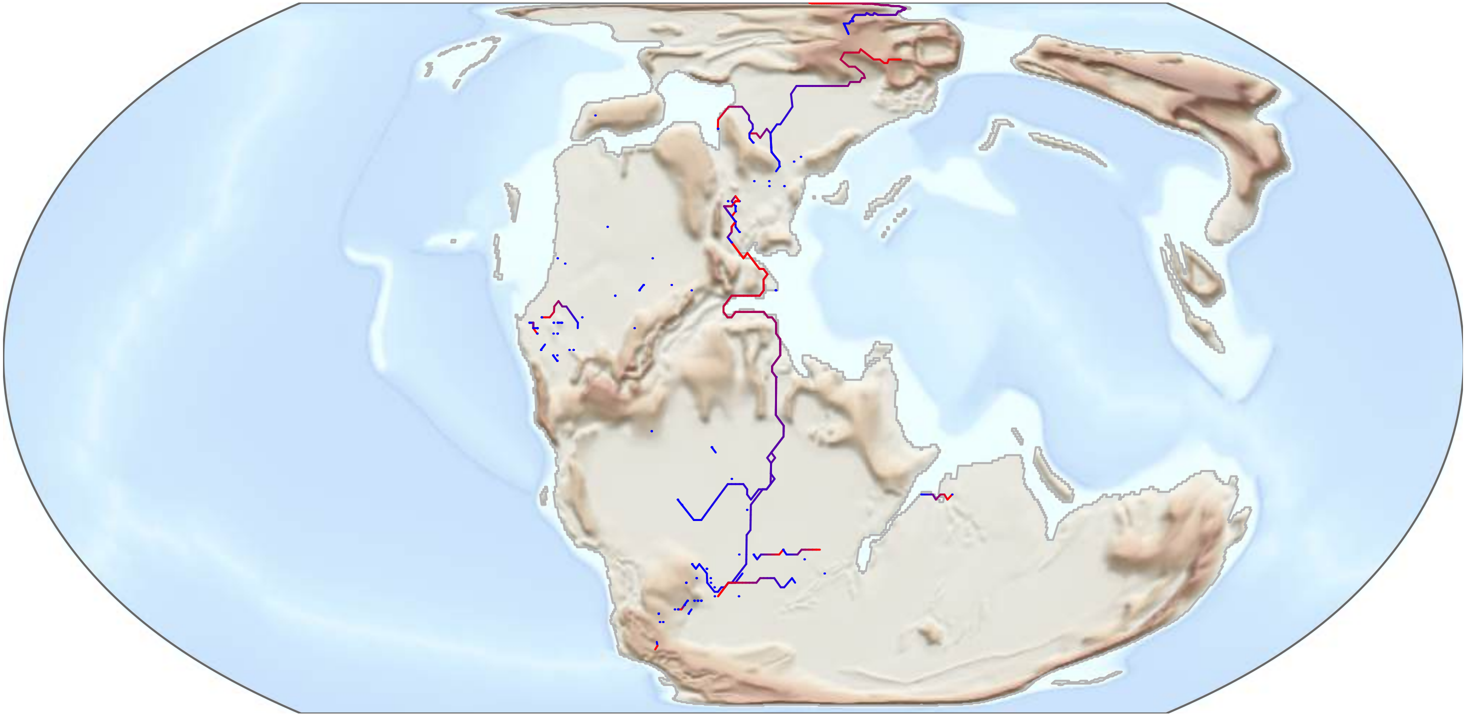

Phylogeographic dispersal paths run gradually from blue to red.

Paths may begin at the terminus of an ancestral path (immediate red to blue jump)

or share a common starting point (blue central region bounded by red ends

Fig. S12: Tuvalian

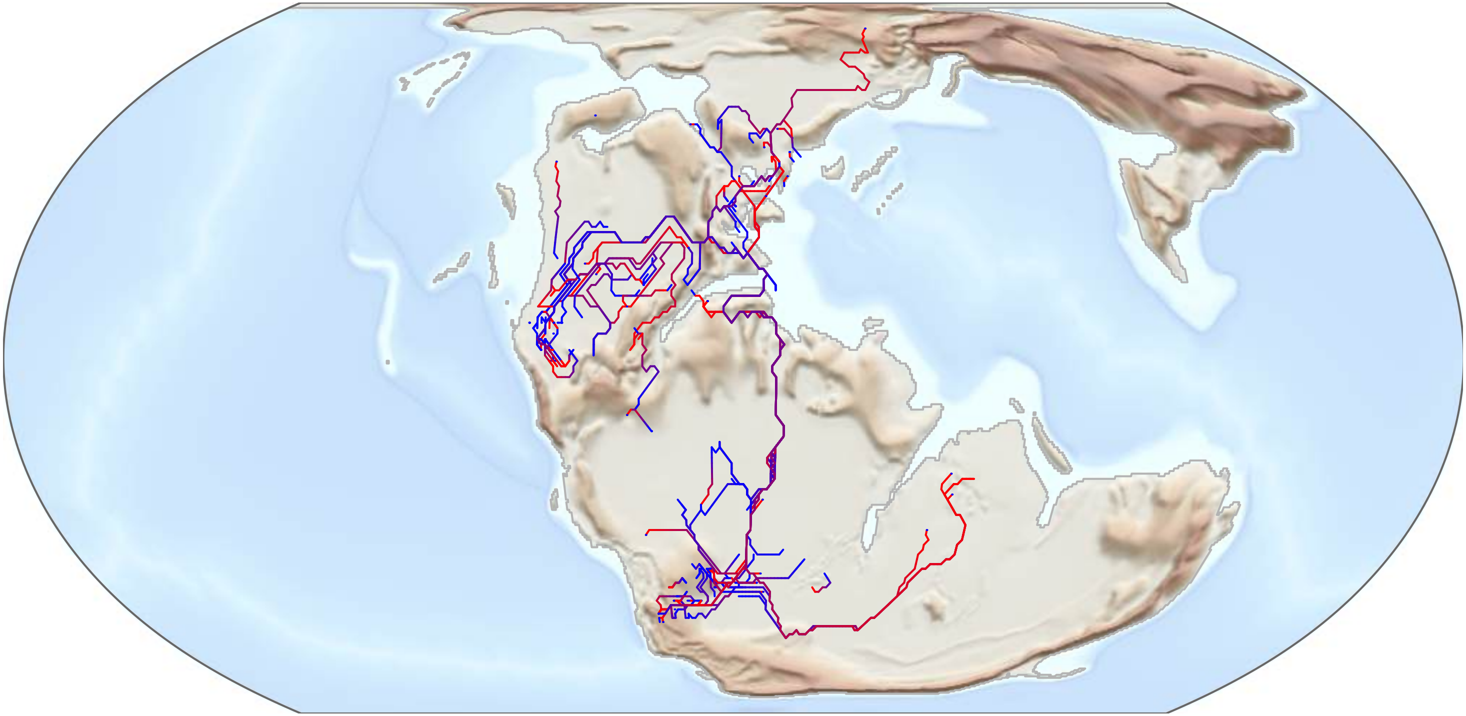

Phylogeographic dispersal paths run gradually from blue to red.

Paths may begin at the terminus of an ancestral path (immediate red to blue jump)

or share a common starting point (blue central region bounded by red ends

Fig. S13: Lacian

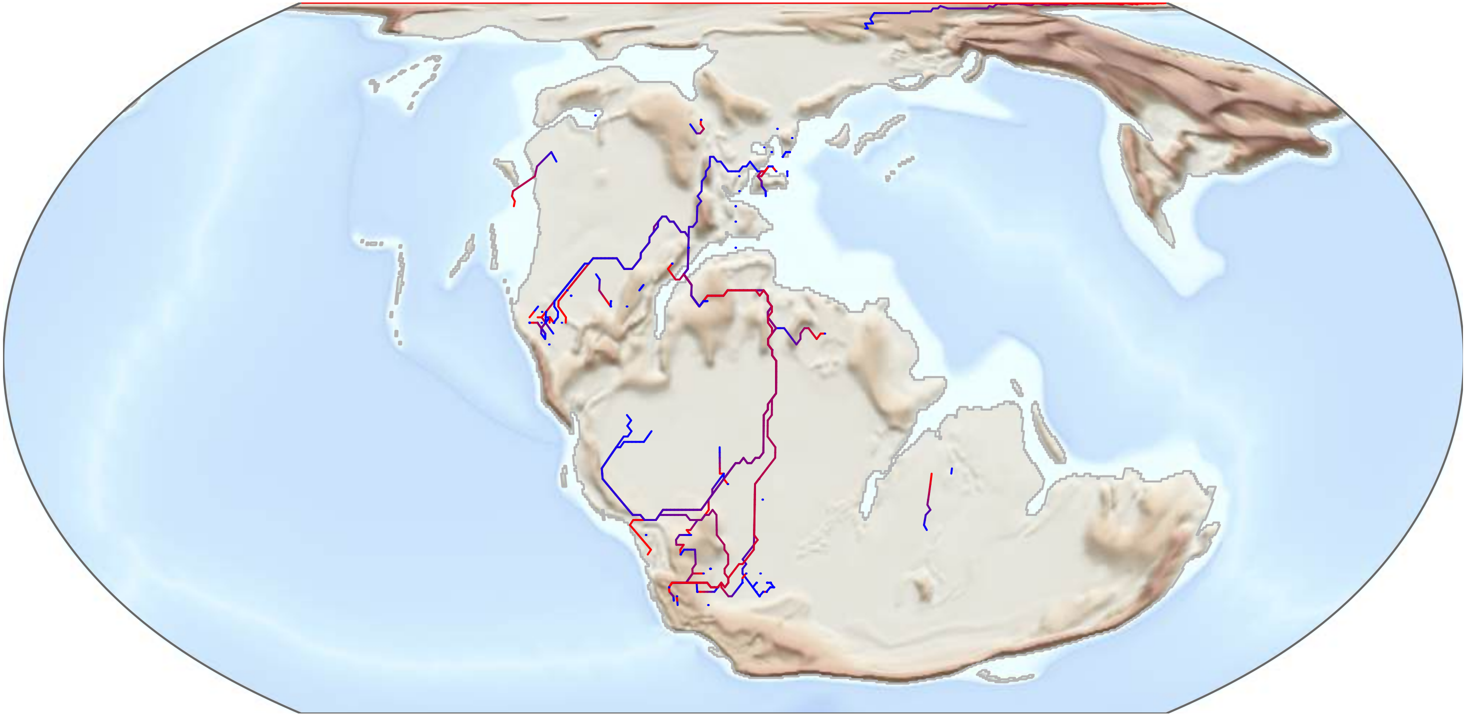

Phylogeographic dispersal paths run gradually from blue to red.

Paths may begin at the terminus of an ancestral path (immediate red to blue jump)

or share a common starting point (blue central region bounded by red ends

Fig. S14: Alaunian

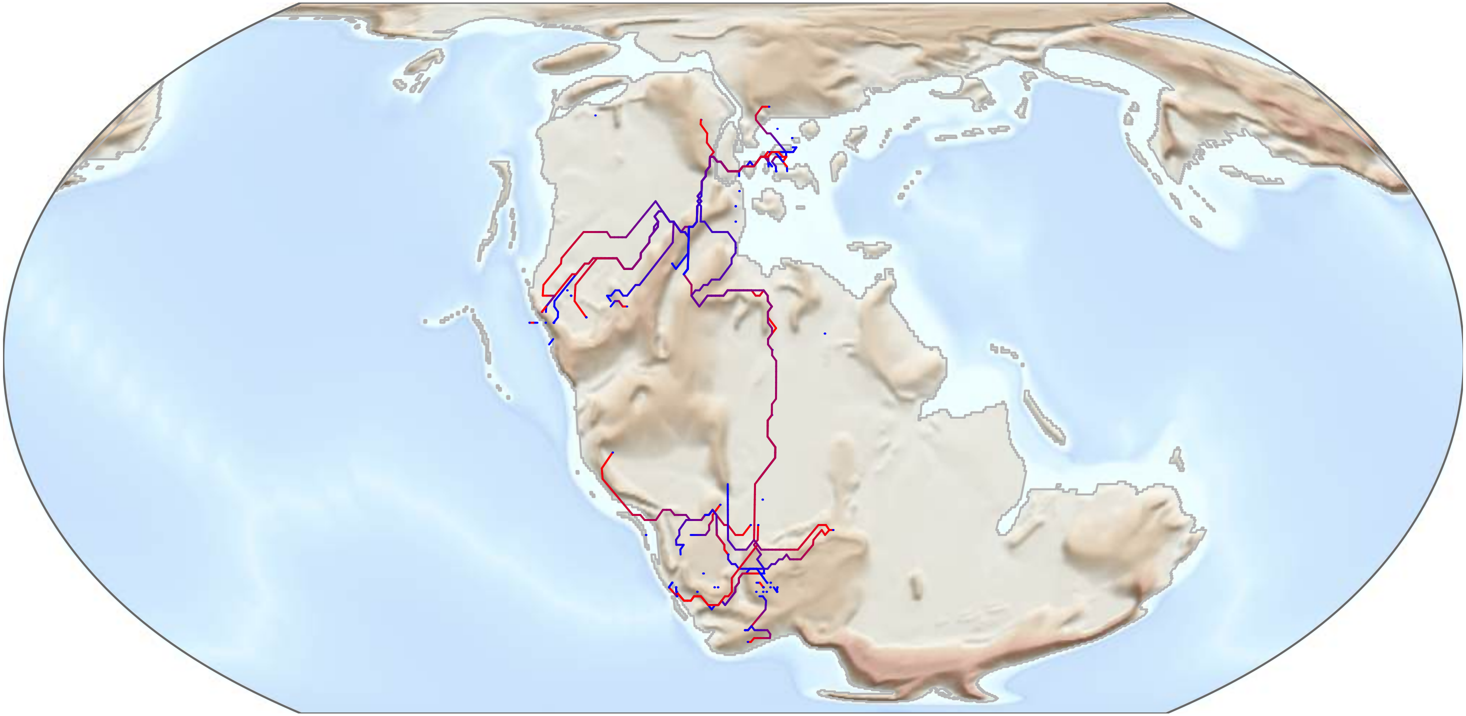

Phylogeographic dispersal paths run gradually from blue to red.

Paths may begin at the terminus of an ancestral path (immediate red to blue jump)

or share a common starting point (blue central region bounded by red ends

Fig. S15: Sevatian

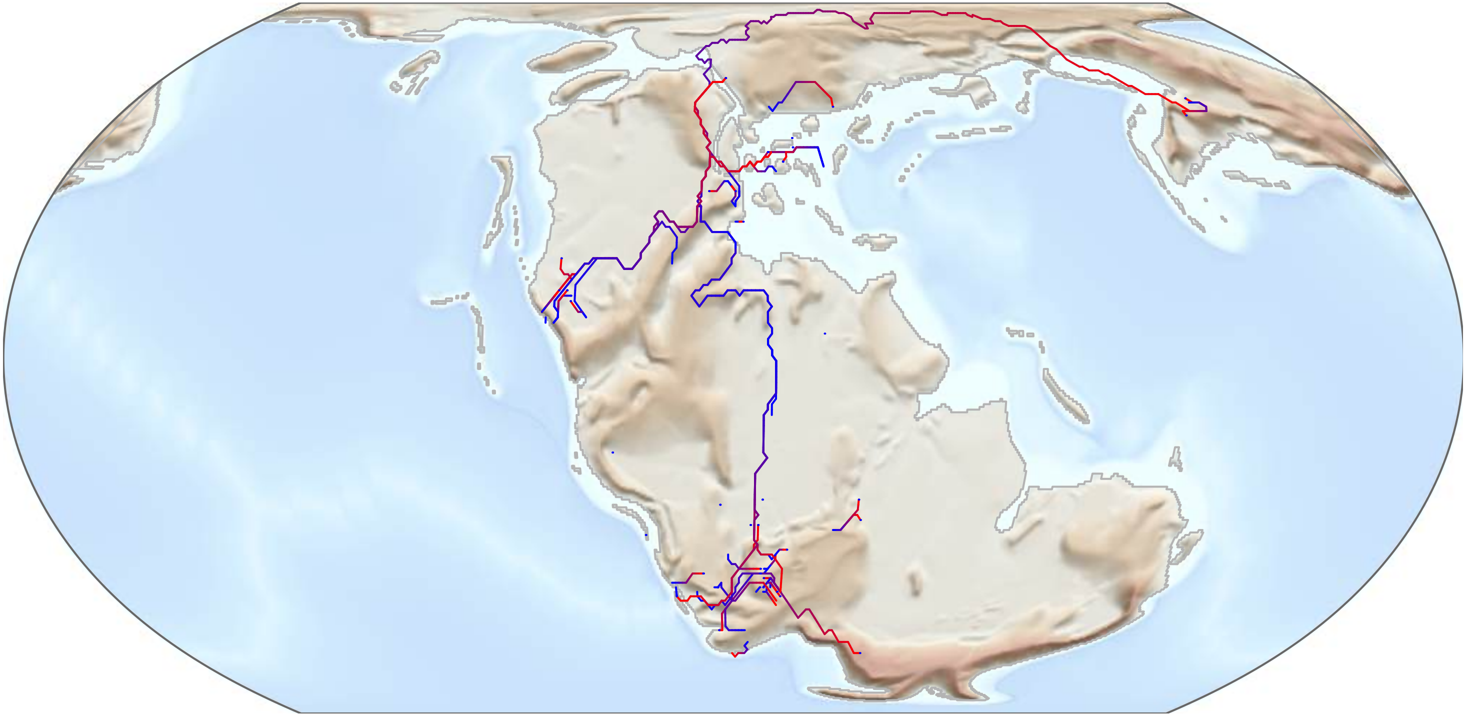

Phylogeographic dispersal paths run gradually from blue to red.

Paths may begin at the terminus of an ancestral path (immediate red to blue jump)

or share a common starting point (blue central region bounded by red ends

Fig. S16: Rhaetian

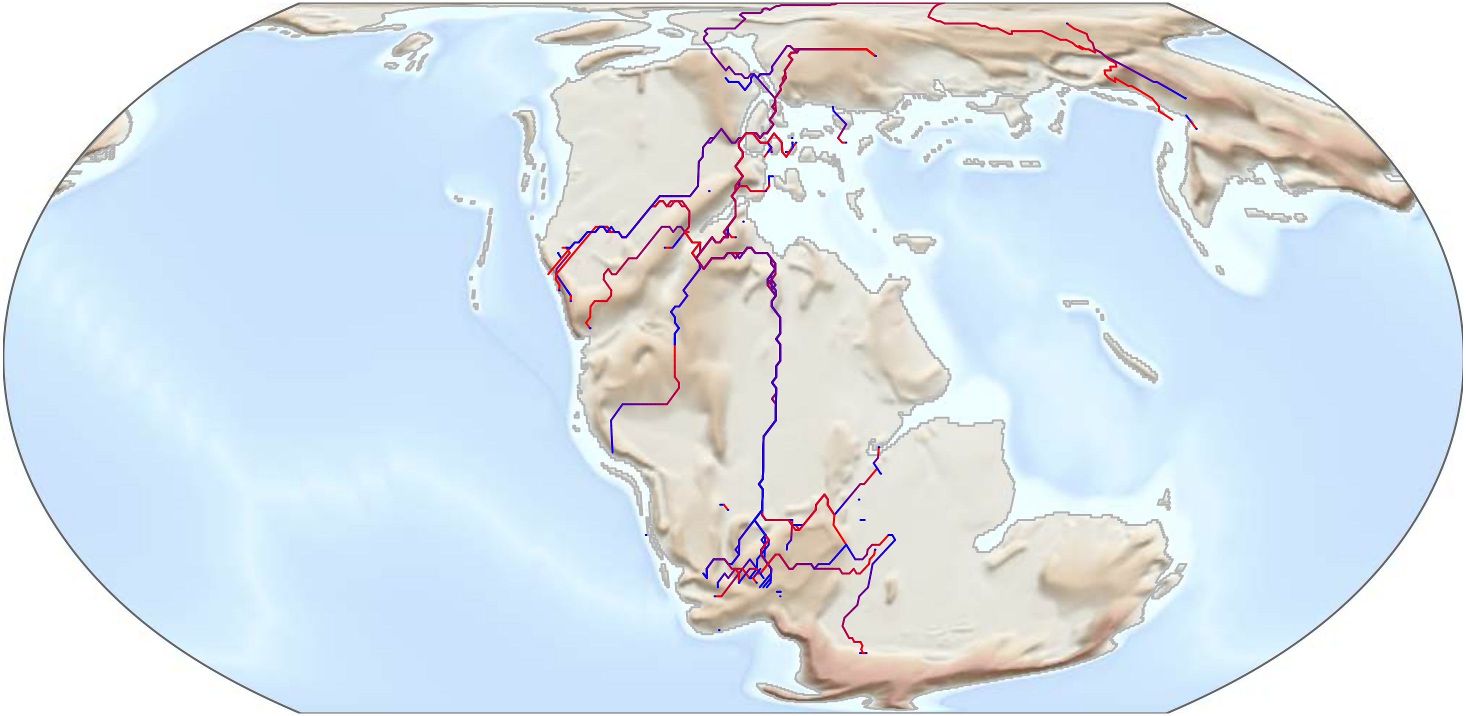

Phylogeographic dispersal paths run gradually from blue to red.

Paths may begin at the terminus of an ancestral path (immediate red to blue jump)

or share a common starting point (blue central region bounded by red ends

Fig. S1: Kungurian

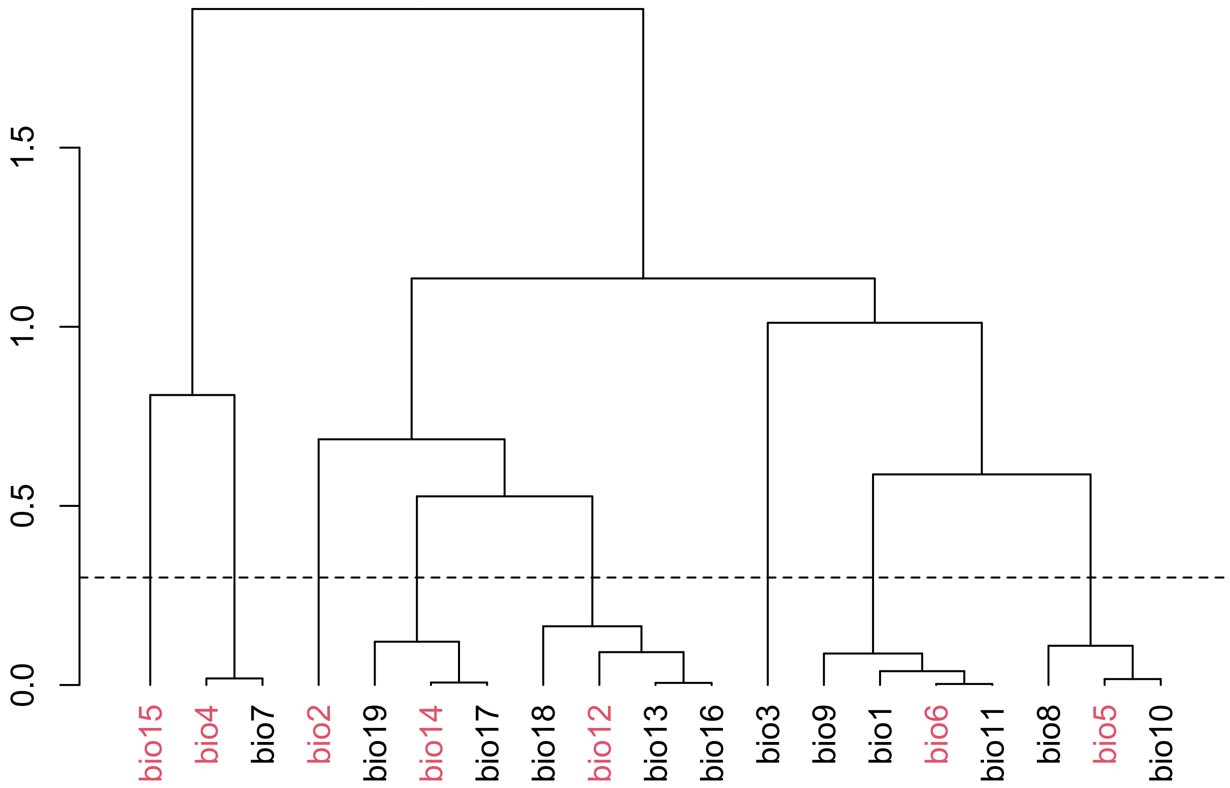

Correlation coefficients of the BIOCLIM variables for the Kungurian subtracted from 1 (i.e., perfect positive correlation = 0, perfect negative correlation = 2).

The dotted line represents a 0.7 correlation coefficient. Red labels were those chosen for each cluster based on the consistency of correlation groups across all dendrograms

Fig. S2: Roadian

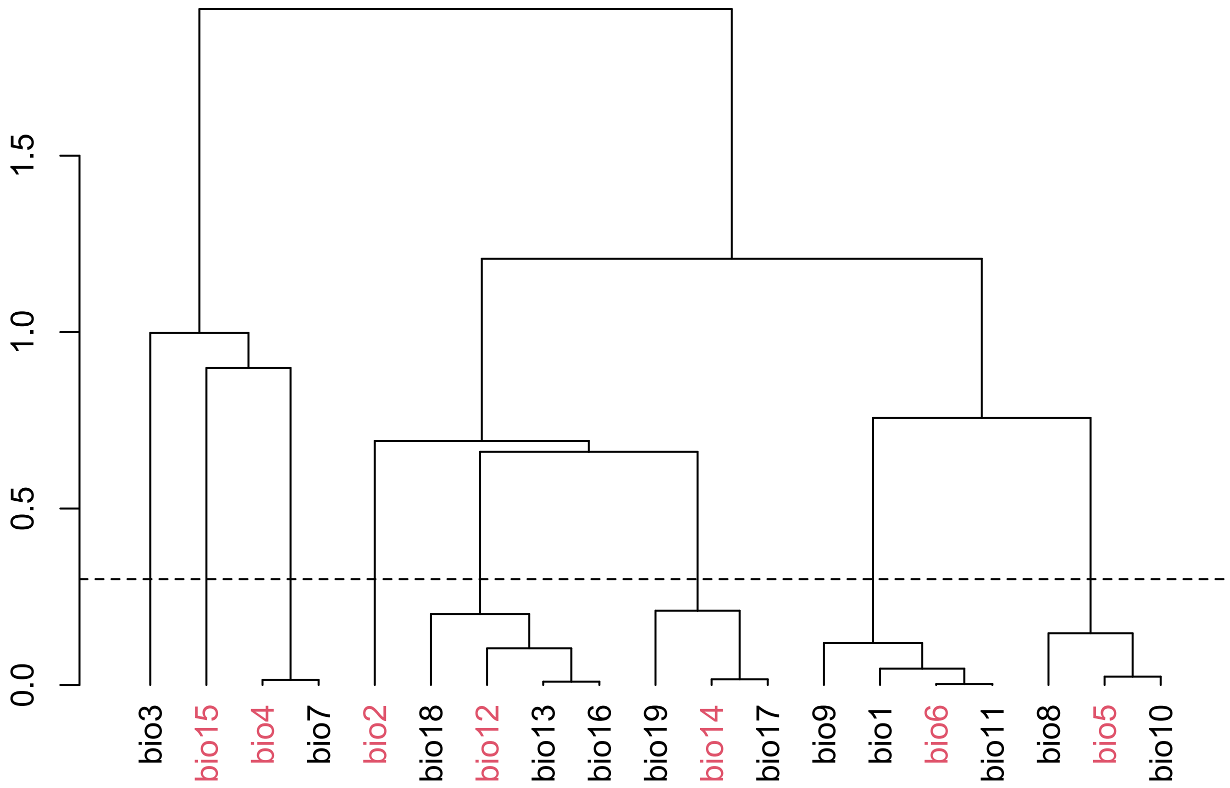

Correlation coefficients of the BIOCLIM variables for the Roadian subtracted from 1 (i.e., perfect positive correlation = 0, perfect negative correlation = 2).

The dotted line represents a 0.7 correlation coefficient. Red labels were those chosen for each cluster based on the consistency of correlation groups across all dendrograms

Fig. S3: Wordian

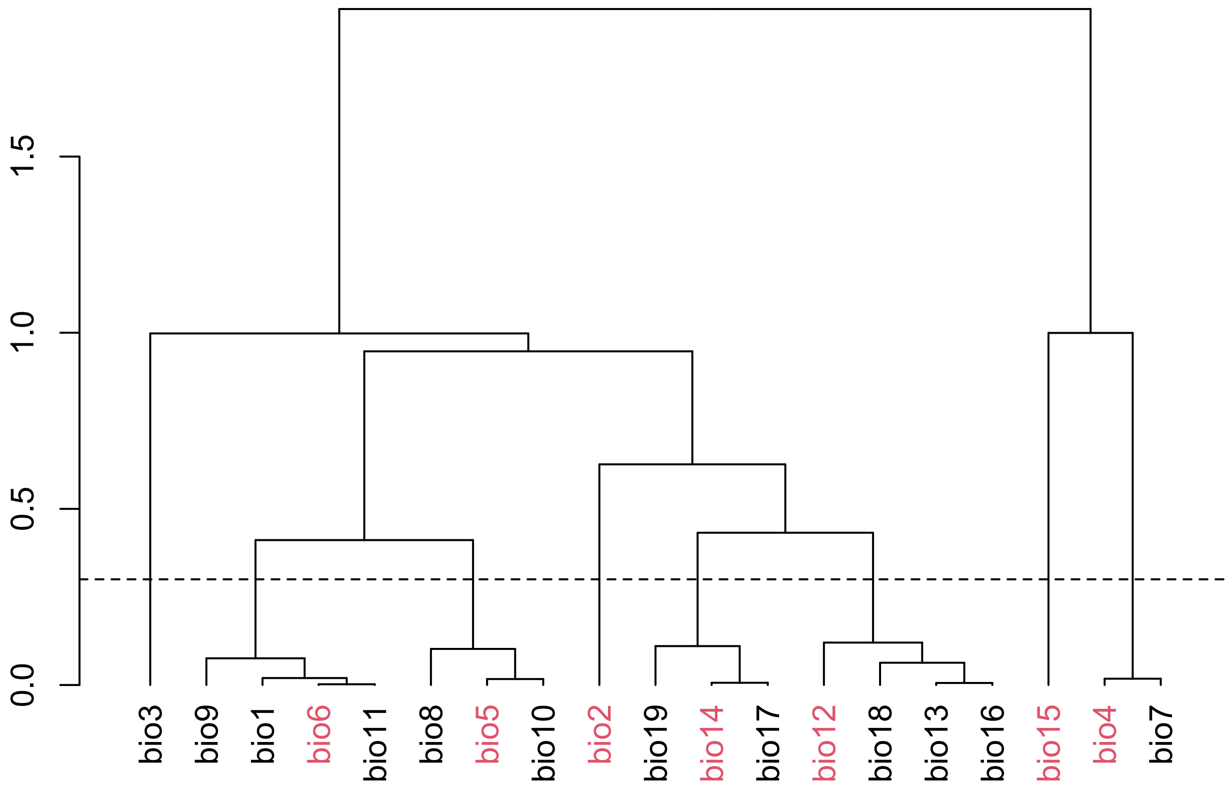

Correlation coefficients of the BIOCLIM variables for the Wordian subtracted from 1 (i.e., perfect positive correlation = 0, perfect negative correlation = 2).

The dotted line represents a 0.7 correlation coefficient. Red labels were those chosen for

each cluster based on the consistency of correlation groups across all dendrograms

Fig. S4: Capitanian

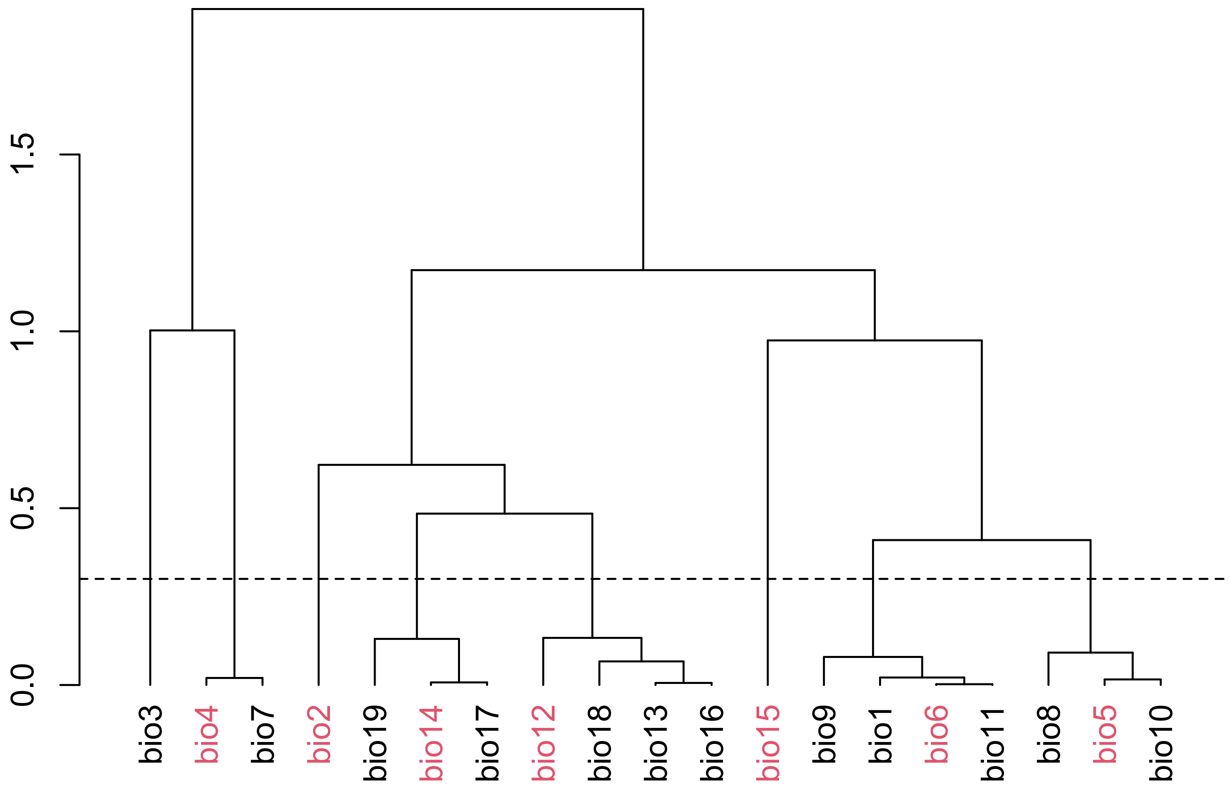

Correlation coefficients of the BIOCLIM variables for the Capitanian subtracted from 1 (i.e., perfect positive correlation = 0, perfect negative correlation = 2).

The dotted line represents a 0.7 correlation coefficient. Red labels were those chosen for each cluster based on the consistency of correlation groups across all dendrograms

Fig. S5: Wuchiapingian

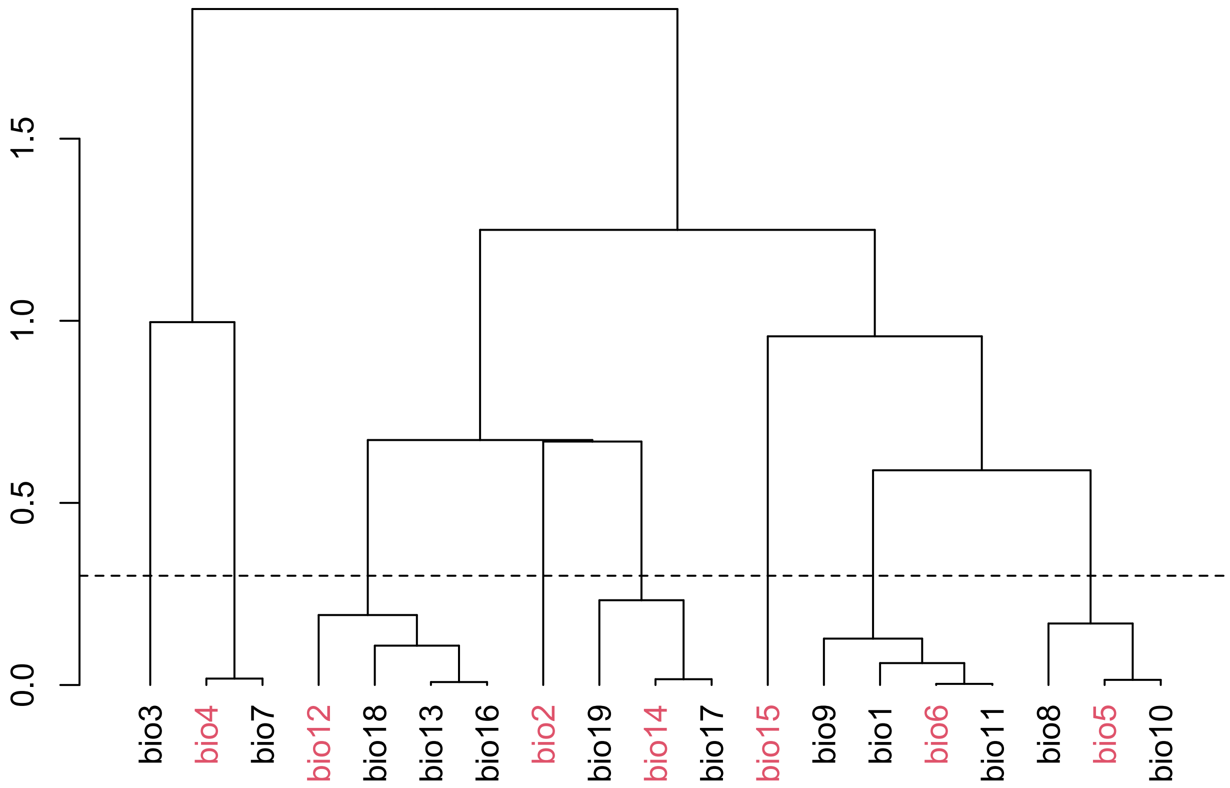

Correlation coefficients of the BIOCLIM variables for the Wuchiapingian subtracted from 1 (i.e., perfect positive correlation = 0, perfect negative correlation = 2).

The dotted line represents a 0.7 correlation coefficient. Red labels were those chosen for each cluster based on the consistency of correlation groups across all dendrograms

Fig. S6: Changhsingian

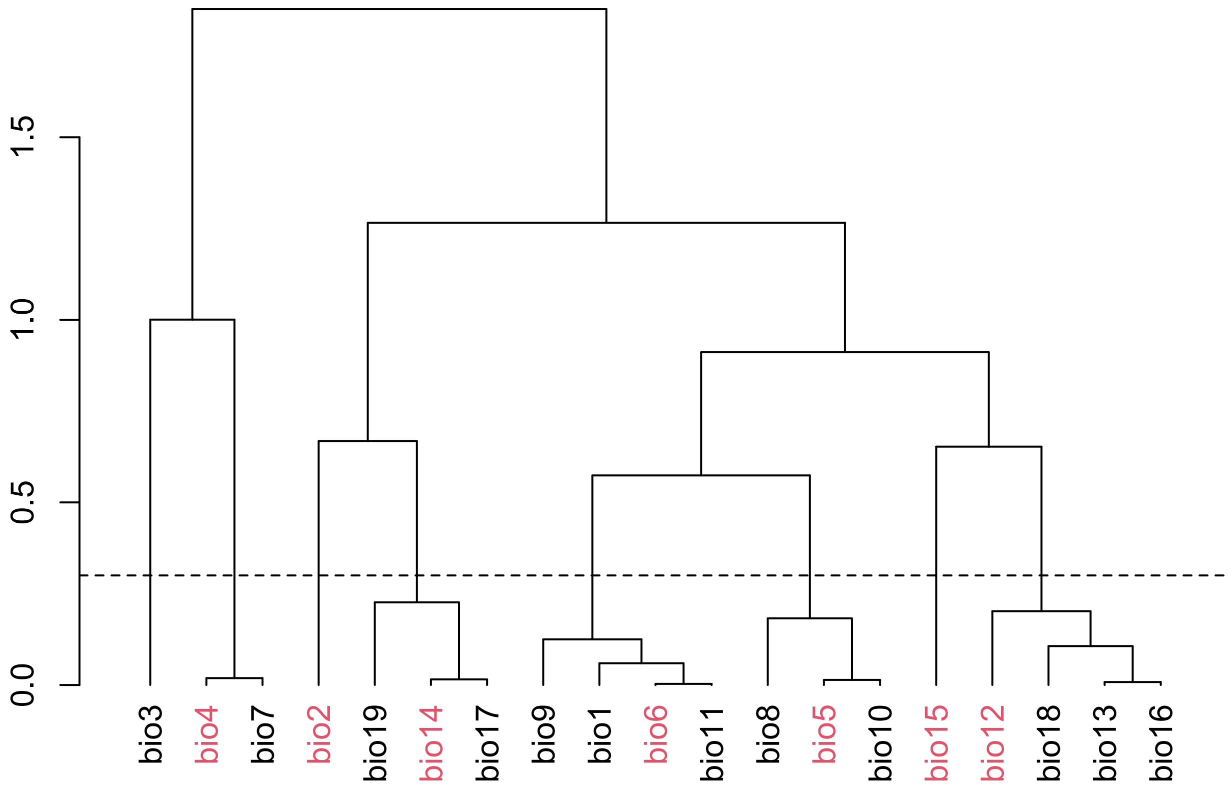

Correlation coefficients of the BIOCLIM variables for the Changhsingian subtracted from 1 (i.e., perfect positive correlation = 0, perfect negative correlation = 2).

The dotted line represents a 0.7 correlation coefficient. Red labels were those chosen for

each cluster based on the consistency of correlation groups across all dendrograms

Fig. S7: Induan

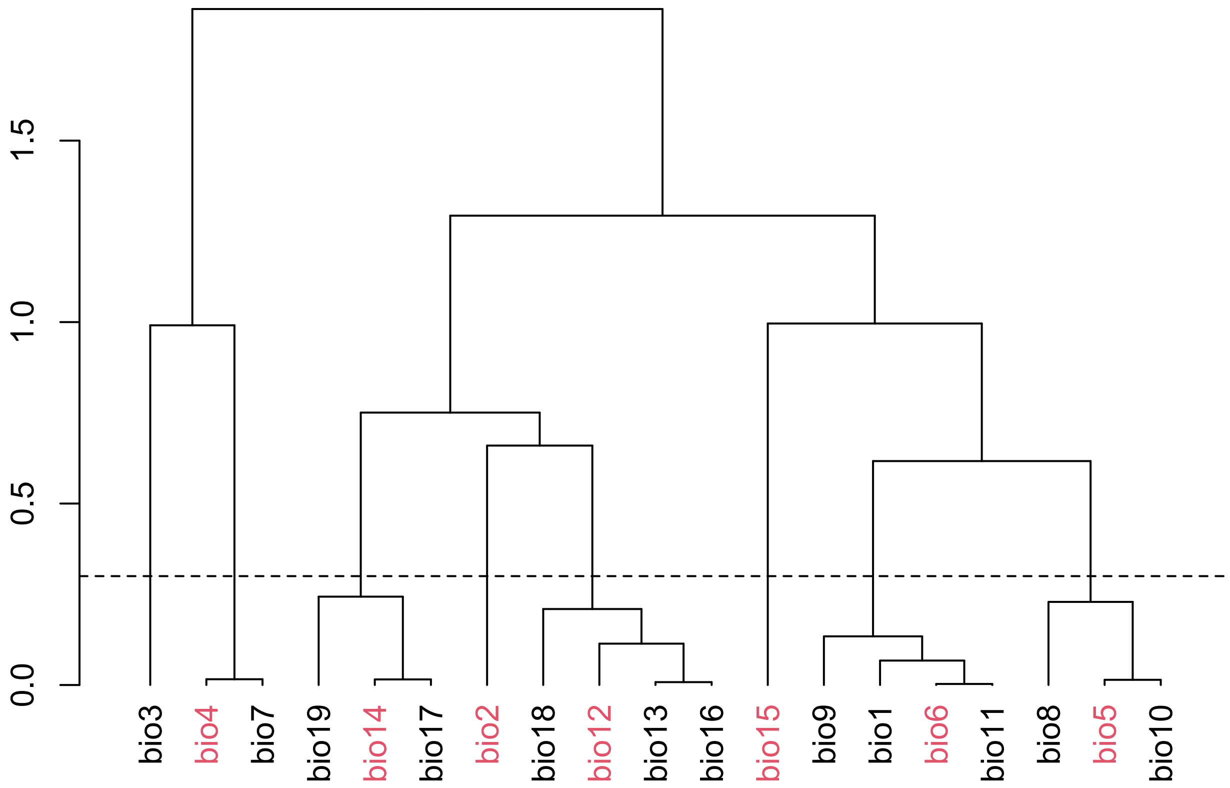

Correlation coefficients of the BIOCLIM variables for the Induan subtracted from 1 (i.e., perfect positive correlation = 0, perfect negative correlation = 2).

The dotted line represents a 0.7 correlation coefficient. Red labels were those chosen for

each cluster based on the consistency of correlation groups across all dendrograms

Fig. S8: Olenekian

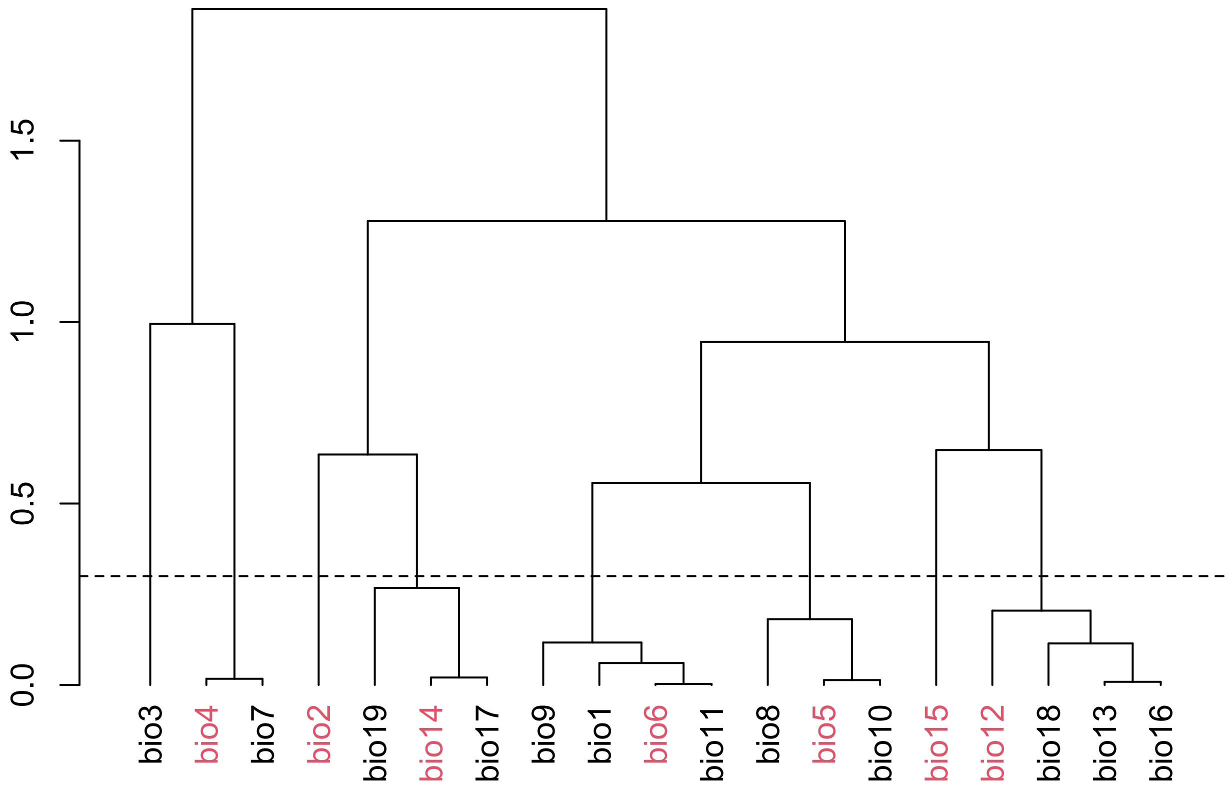

Correlation coefficients of the BIOCLIM variables for the Olenekian subtracted from 1 (i.e., perfect positive correlation = 0, perfect negative correlation = 2).

The dotted line represents a 0.7 correlation coefficient. Red labels were those chosen for each cluster based on the consistency of correlation groups across all dendrograms

Fig. S9: Anisian

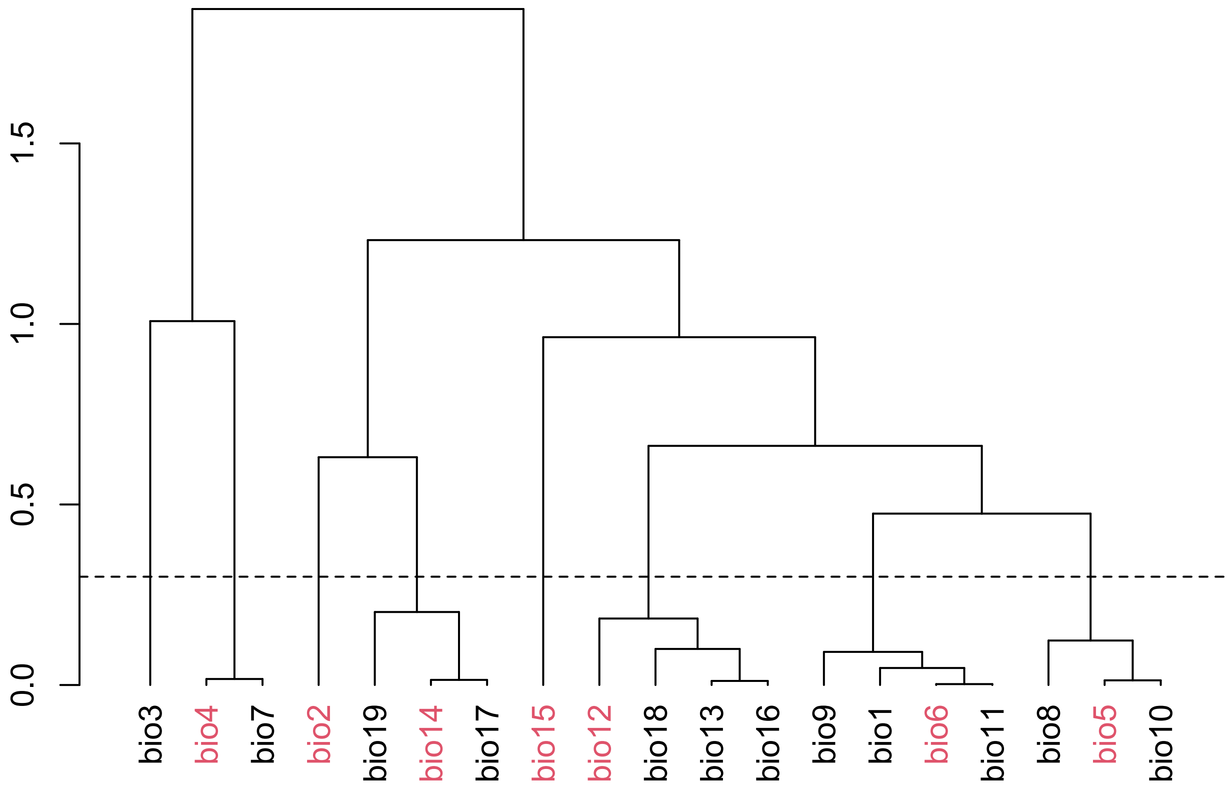

Correlation coefficients of the BIOCLIM variables for the Anisian subtracted from 1 (i.e., perfect positive correlation = 0, perfect negative correlation = 2).

The dotted line represents a 0.7 correlation coefficient. Red labels were those chosen for each cluster based on the consistency of correlation groups across all dendrograms

Fig. S10: Ladinian

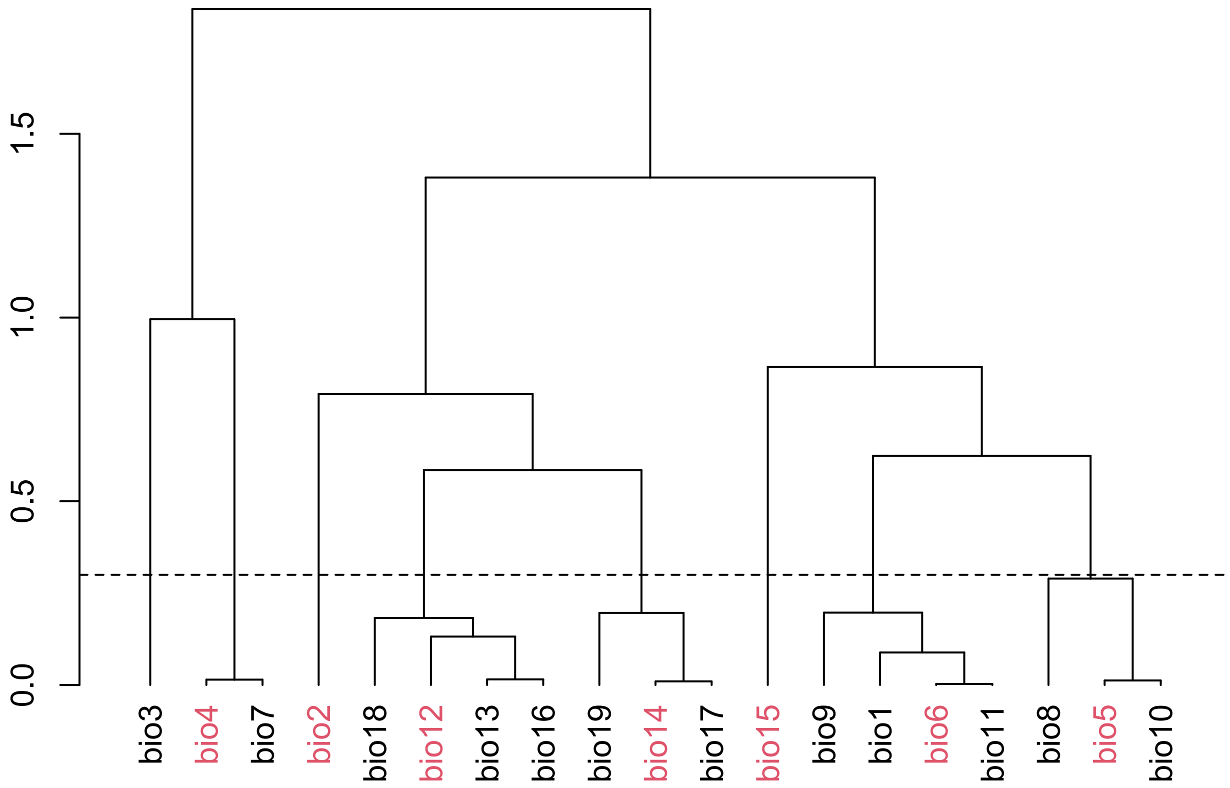

Correlation coefficients of the BIOCLIM variables for the Ladinian subtracted from 1 (i.e., perfect positive correlation = 0, perfect negative correlation = 2).

The dotted line represents a 0.7 correlation coefficient. Red labels were those chosen for

each cluster based on the consistency of correlation groups across all dendrograms

Fig. S11: Julian

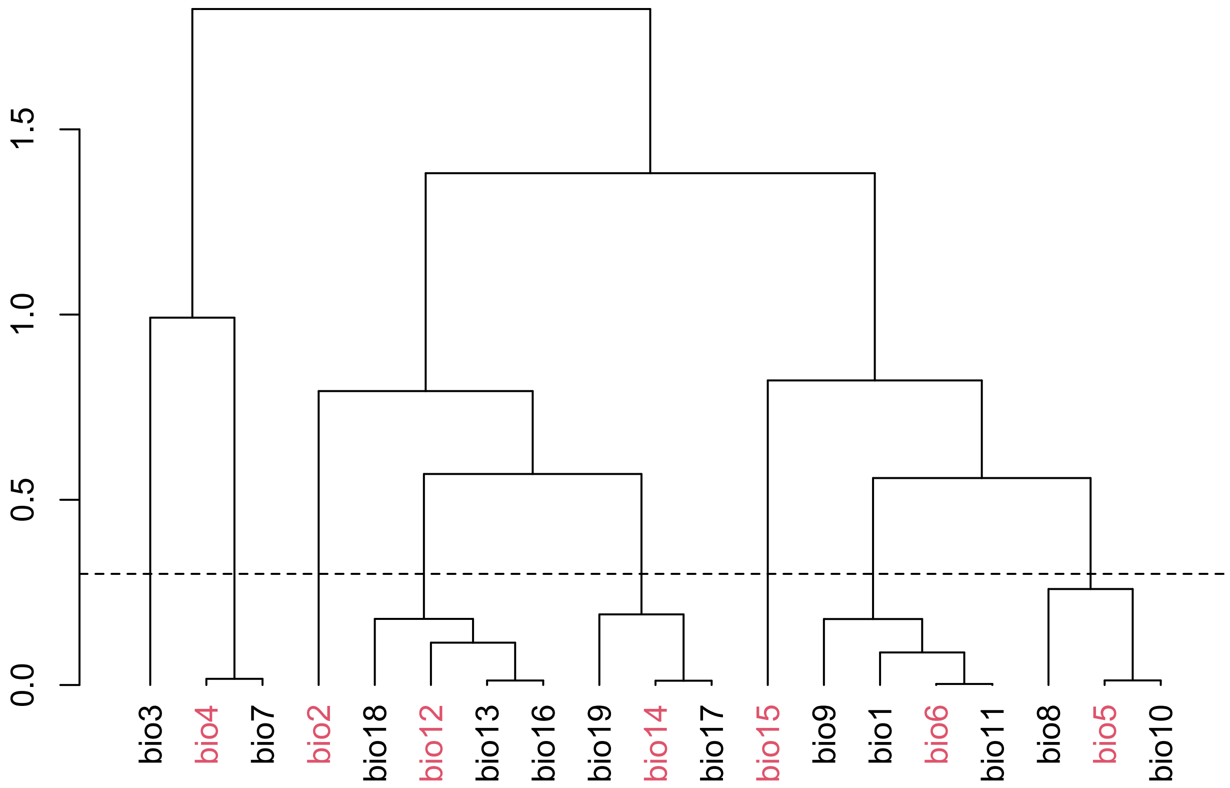

Correlation coefficients of the BIOCLIM variables for the Julian subtracted from 1 (i.e., perfect positive correlation = 0, perfect negative correlation = 2).

The dotted line represents a 0.7 correlation coefficient. Red labels were those chosen for each cluster based on the consistency of correlation groups across all dendrograms

Fig. S12: Tuvalian

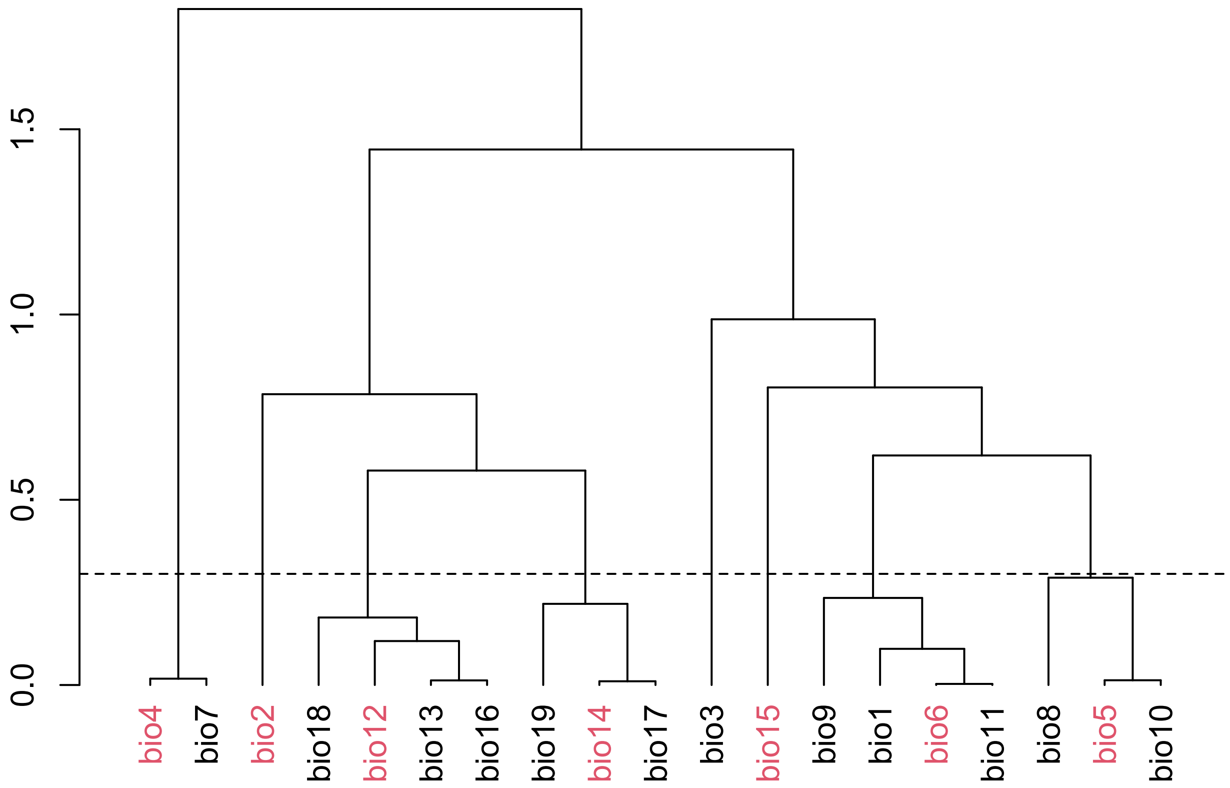

Correlation coefficients of the BIOCLIM variables for the Tuvalian subtracted from 1 (i.e., perfect positive correlation = 0, perfect negative correlation = 2).

The dotted line represents a 0.7 correlation coefficient. Red labels were those chosen for

each cluster based on the consistency of correlation groups across all dendrograms

Fig. S13: Lacian

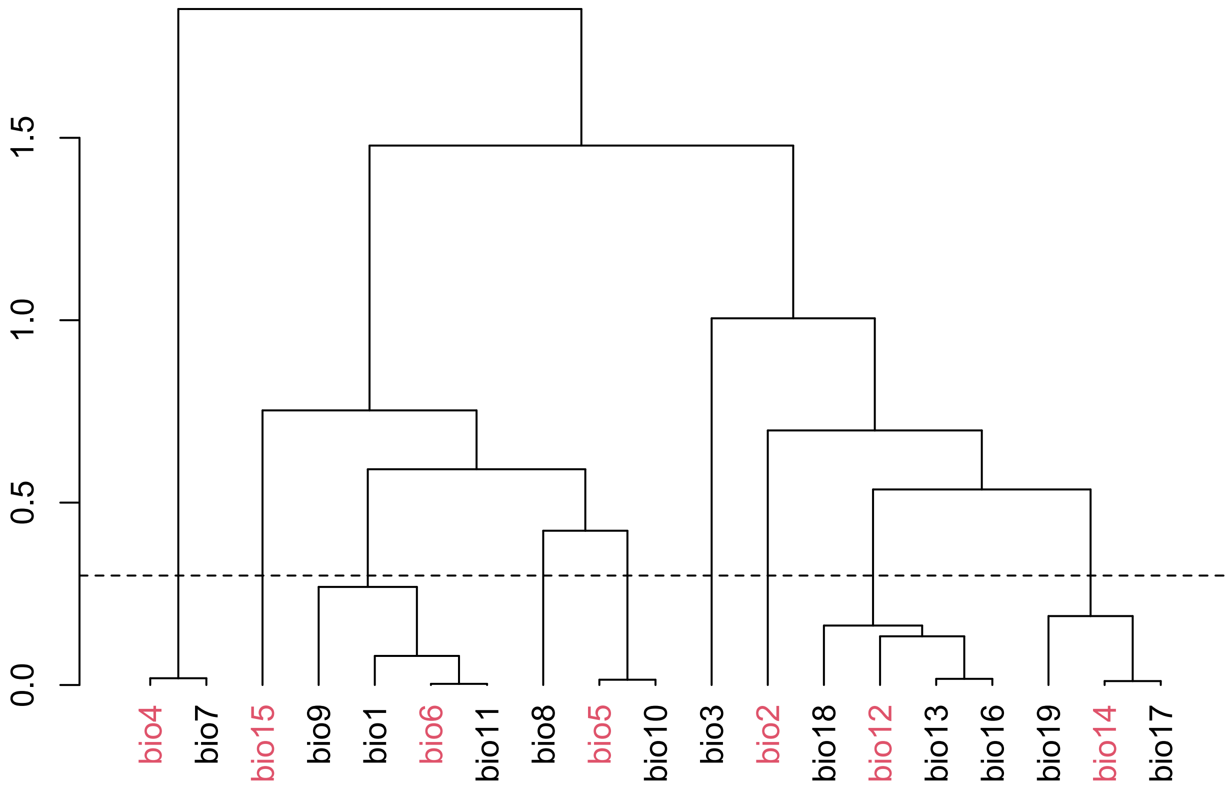

Correlation coefficients of the BIOCLIM variables for the Lacian subtracted from 1 (i.e., perfect positive correlation = 0, perfect negative correlation = 2).

The dotted line represents a 0.7 correlation coefficient. Red labels were those chosen for each cluster based on the consistency of correlation groups across all dendrograms

Fig. S14: Alaunian

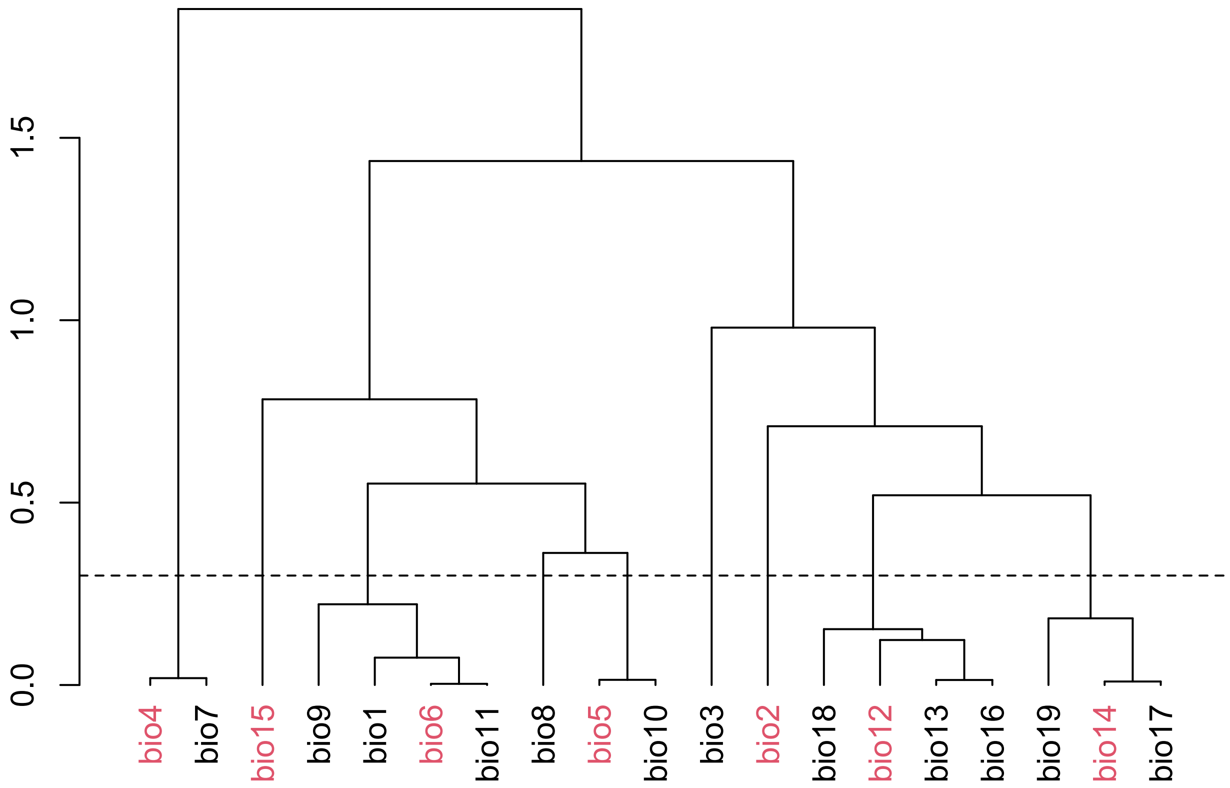

Correlation coefficients of the BIOCLIM variables for the Alaunian subtracted from 1 (i.e., perfect positive correlation = 0, perfect negative correlation = 2).

The dotted line represents a 0.7 correlation coefficient. Red labels were those chosen for each cluster based on the consistency of correlation groups across all dendrograms

Fig. S15: Sevatian

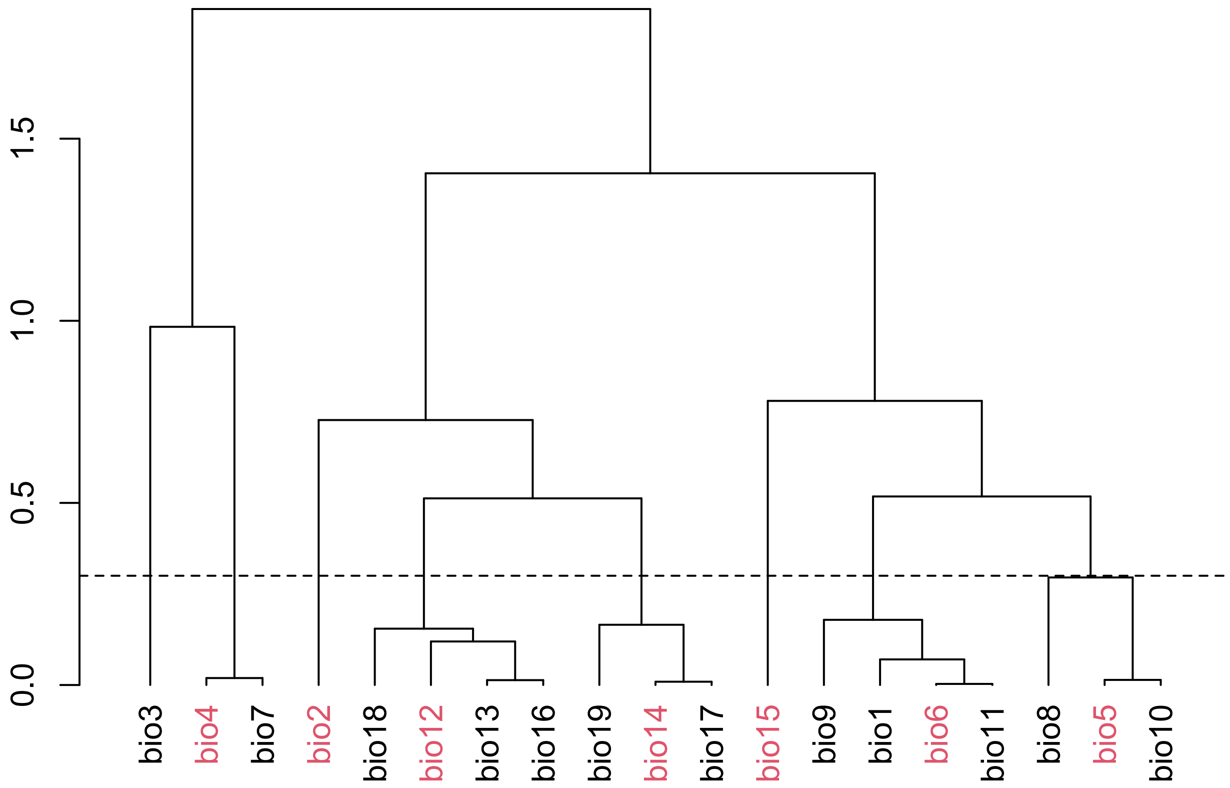

Correlation coefficients of the BIOCLIM variables for the Sevatian subtracted from 1 (i.e., perfect positive correlation = 0, perfect negative correlation = 2).

The dotted line represents a 0.7 correlation coefficient. Red labels were those chosen for

each cluster based on the consistency of correlation groups across all dendrograms

Fig. S16: Rhaetian

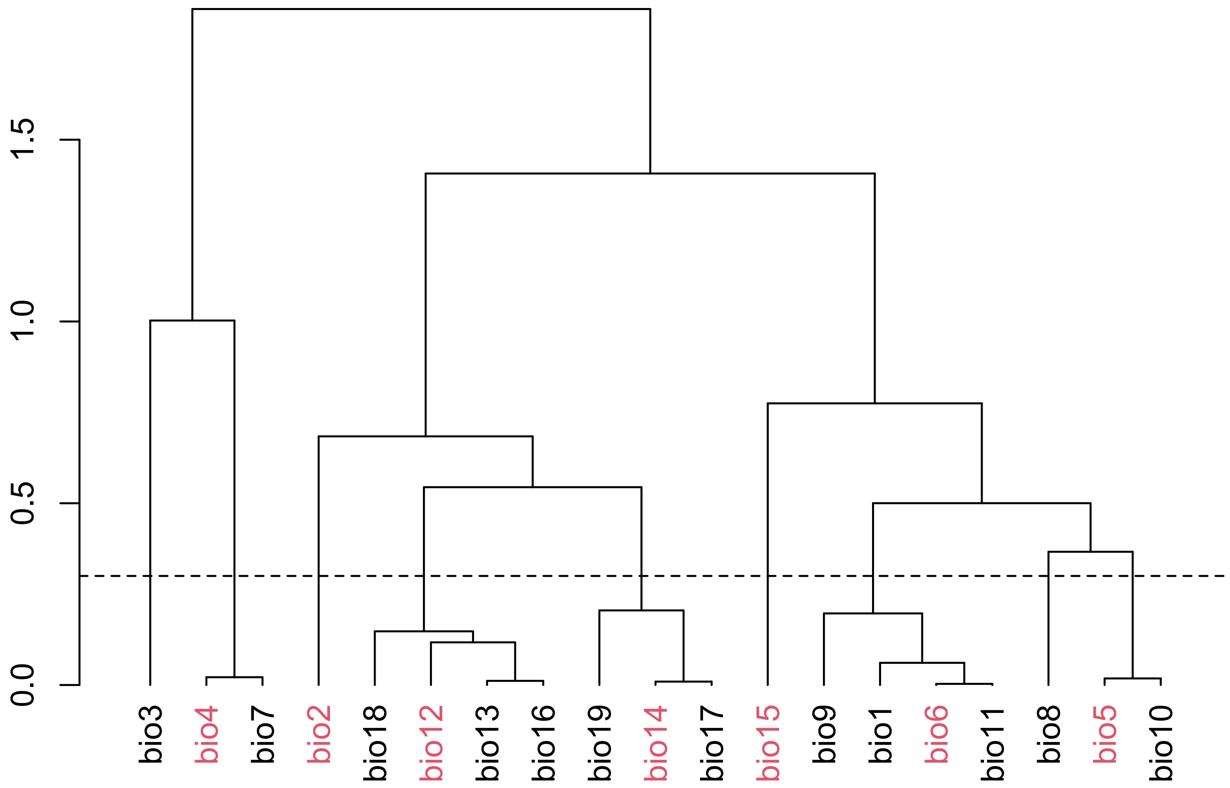

Correlation coefficients of the BIOCLIM variables for the Rhaetian subtracted from 1 (i.e., perfect positive correlation = 0, perfect negative correlation = 2).

The dotted line represents a 0.7 correlation coefficient. Red labels were those chosen for each cluster based on the consistency of correlation groups across all dendrograms

Table S1

trad	lag	sil	both	
bm	1	1	1	1
vr	0.890066	0.88543	0.890212	0.936592

Table S2: Potential scale reduction factors of ancestral state estimates across our alternative topologies under Brownian motion

trad_bm	trad_bm	lag_bm	lag_bm	sil_bm	sil_bm
param	psrf	param	psrf	param	psrf
Lh	0.99998	Lh	0.999977	Lh	0.999987
Scale	1	Scale	1.000004	Scale	1.000054
Node-00000 - Long	1.000016	Node-00000 - Long	1.000099	Node-00000 - Long	1.000016
Node-00000 - Lat	1.000073	Node-00000 - Lat	0.999989	Node-00000 - Lat	1.000028
Node-00001 - Long	1.000008	Node-00001 - Long	1.000054	Node-00001 - Long	1.000055
Node-00001 - Lat	1.000125	Node-00001 - Lat	1.000034	Node-00001 - Lat	1.00008
Node-00002 - Long	1.000003	Node-00002 - Long	1.000065	Node-00002 - Long	0.999999
Node-00002 - Lat	1.000082	Node-00002 - Lat	1.000085	Node-00002 - Lat	1.000009
Node-00003 - Long	0.999998	Node-00003 - Long	1.000041	Node-00003 - Long	1.000011
Node-00003 - Lat	1.000035	Node-00003 - Lat	1.000074	Node-00003 - Lat	1.000024
Node-00004 - Long	1.000002	Node-00004 - Long	1.000036	Node-00004 - Long	0.999996
Node-00004 - Lat	1.000026	Node-00004 - Lat	0.999992	Node-00004 - Lat	1.000009
Node-00005 - Long	1.000049	Node-00005 - Long	1.000076	Node-00005 - Long	1.000004
Node-00005 - Lat	1.000017	Node-00005 - Lat	0.999994	Node-00005 - Lat	1.000014
Node-00006 - Long	1.000011	Node-00006 - Long	1.000102	Node-00006 - Long	1.000015
Node-00006 - Lat	1.000034	Node-00006 - Lat	0.999997	Node-00006 - Lat	1.000032
Node-00007 - Long	1.000006	Node-00007 - Long	1.000083	Node-00007 - Long	1.00002
Node-00007 - Lat	0.99999	Node-00007 - Lat	1.000001	Node-00007 - Lat	1.000008
Node-00008 - Long	1.000004	Node-00008 - Long	1.000077	Node-00008 - Long	1.000018
Node-00008 - Lat	1.000017	Node-00008 - Lat	0.999978	Node-00008 - Lat	1.000019
Node-00009 - Long	0.99998	Node-00009 - Long	1.000071	Node-00009 - Long	0.999983
Node-00009 - Lat	1.000072	Node-00009 - Lat	0.999985	Node-00009 - Lat	1.000038
Node-00010 - Long	1.000035	Node-00010 - Long	1.000063	Node-00010 - Long	1.000012
Node-00010 - Lat	1.000008	Node-00010 - Lat	1.000047	Node-00010 - Lat	0.999979
Node-00011 - Long	0.999999	Node-00011 - Long	0.999982	Node-00011 - Long	1.000014
Node-00011 - Lat	1.000018	Node-00011 - Lat	1.000047	Node-00011 - Lat	1.000011
Node-00012 - Long	1.00006	Node-00012 - Long	1.000008	Node-00012 - Long	1.000047
Node-00012 - Lat	1.000048	Node-00012 - Lat	1.000065	Node-00012 - Lat	1.00001
Node-00013 - Long	0.999995	Node-00013 - Long	1.000077	Node-00013 - Long	1.000007
Node-00013 - Lat	1.000052	Node-00013 - Lat	1.000025	Node-00013 - Lat	1.000036
Node-00014 - Long	0.999984	Node-00014 - Long	1.000152	Node-00014 - Long	0.999988
Node-00014 - Lat	1.000015	Node-00014 - Lat	1.00007	Node-00014 - Lat	1.000028
Node-00015 - Long	1.000006	Node-00015 - Long	1.000115	Node-00015 - Long	1.000023
Node-00015 - Lat	1.000006	Node-00015 - Lat	1.000069	Node-00015 - Lat	1.000084
Node-00016 - Long	1.000031	Node-00016 - Long	1.000048	Node-00016 - Long	1.000029
Node-00016 - Lat	1.000023	Node-00016 - Lat	1.000093	Node-00016 - Lat	1.00005
Node-00017 - Long	1.000064	Node-00017 - Long	1.000124	Node-00017 - Long	1.000012
Node-00017 - Lat	1.000051	Node-00017 - Lat	1.000036	Node-00017 - Lat	0.999961
Node-00018 - Long	1.000037	Node-00018 - Long	1.000098	Node-00018 - Long	1.000008
Node-00018 - Lat	1.000018	Node-00018 - Lat	1.00003	Node-00018 - Lat	1.000006
Node-00019 - Long	1.000101	Node-00019 - Long	1.000075	Node-00019 - Long	1.00005
Node-00019 - Lat	1.000091	Node-00019 - Lat	1.000031	Node-00019 - Lat	1.000077
Node-00020 - Long	1.000168	Node-00020 - Long	1.00006	Node-00020 - Long	1.000013

Node-00020 - Lat	1.000189	Node-00020 - Lat	1.000085	Node-00020 - Lat	1.000035
Node-00021 - Long	1.000058	Node-00021 - Long	1.000099	Node-00021 - Long	1.000063
Node-00021 - Lat	1.000073	Node-00021 - Lat	1.000101	Node-00021 - Lat	1.00004
Node-00022 - Long	1.000034	Node-00022 - Long	1.000033	Node-00022 - Long	1.000026
Node-00022 - Lat	1.000082	Node-00022 - Lat	1.00001	Node-00022 - Lat	1
Node-00023 - Long	1.000014	Node-00023 - Long	1.000019	Node-00023 - Long	1.00003
Node-00023 - Lat	1.00001	Node-00023 - Lat	1.000006	Node-00023 - Lat	0.999991
Node-00024 - Long	0.999986	Node-00024 - Long	1.000014	Node-00024 - Long	1.000005
Node-00024 - Lat	0.99997	Node-00024 - Lat	0.999993	Node-00024 - Lat	1.000012
Node-00025 - Long	1.000013	Node-00025 - Long	0.999996	Node-00025 - Long	0.999992
Node-00025 - Lat	0.999963	Node-00025 - Lat	0.999993	Node-00025 - Lat	1.000026
Node-00026 - Long	0.999995	Node-00026 - Long	1.00001	Node-00026 - Long	1.000046
Node-00026 - Lat	0.99997	Node-00026 - Lat	1.000089	Node-00026 - Lat	1.000119
Node-00027 - Long	0.999975	Node-00027 - Long	1.000052	Node-00027 - Long	1.00015
Node-00027 - Lat	0.999978	Node-00027 - Lat	1.000123	Node-00027 - Lat	0.999987
Node-00028 - Long	0.999992	Node-00028 - Long	1.000068	Node-00028 - Long	1.000033
Node-00028 - Lat	1.000043	Node-00028 - Lat	1.000118	Node-00028 - Lat	1.000037
Node-00029 - Long	0.999991	Node-00029 - Long	1.000039	Node-00029 - Long	1.000057
Node-00029 - Lat	0.999971	Node-00029 - Lat	1.000076	Node-00029 - Lat	1.000182
Node-00030 - Long	0.999995	Node-00030 - Long	1.000071	Node-00030 - Long	0.999999
Node-00030 - Lat	1.000021	Node-00030 - Lat	0.999985	Node-00030 - Lat	1.000062
Node-00031 - Long	1.000002	Node-00031 - Long	1.000004	Node-00031 - Long	0.999993
Node-00031 - Lat	1.000009	Node-00031 - Lat	0.999992	Node-00031 - Lat	0.999998
Node-00032 - Long	1.000012	Node-00032 - Long	1.000029	Node-00032 - Long	0.999994
Node-00032 - Lat	1.000072	Node-00032 - Lat	1.000027	Node-00032 - Lat	0.999985
Node-00033 - Long	1.000033	Node-00033 - Long	1.000048	Node-00033 - Long	1
Node-00033 - Lat	1.000094	Node-00033 - Lat	1.000028	Node-00033 - Lat	1.000053
Node-00034 - Long	1.000121	Node-00034 - Long	1.000055	Node-00034 - Long	1.000027
Node-00034 - Lat	1.000061	Node-00034 - Lat	1.000048	Node-00034 - Lat	1.000004
Node-00035 - Long	1.000051	Node-00035 - Long	1.000056	Node-00035 - Long	1.000021
Node-00035 - Lat	1.000084	Node-00035 - Lat	1.000056	Node-00035 - Lat	1.000013
Node-00036 - Long	1.000032	Node-00036 - Long	1	Node-00036 - Long	1
Node-00036 - Lat	1.000079	Node-00036 - Lat	1.000046	Node-00036 - Lat	1.000054
Node-00037 - Long	1.000027	Node-00037 - Long	0.999994	Node-00037 - Long	1.000031
Node-00037 - Lat	1.000008	Node-00037 - Lat	1.000007	Node-00037 - Lat	1.00004
Node-00038 - Long	1.000009	Node-00038 - Long	0.999993	Node-00038 - Long	1.000016
Node-00038 - Lat	1.000008	Node-00038 - Lat	0.999979	Node-00038 - Lat	1.000045
Node-00039 - Long	1	Node-00039 - Long	0.999969	Node-00039 - Long	1.000069
Node-00039 - Lat	1.000012	Node-00039 - Lat	0.999981	Node-00039 - Lat	1.000056
Node-00040 - Long	0.999994	Node-00040 - Long	1.000004	Node-00040 - Long	1.000096
Node-00040 - Lat	1.00008	Node-00040 - Lat	1.000025	Node-00040 - Lat	1.000014
Node-00041 - Long	0.999981	Node-00041 - Long	0.99999	Node-00041 - Long	1.000082
Node-00041 - Lat	1.00003	Node-00041 - Lat	1.000049	Node-00041 - Lat	1.000038
Node-00042 - Long	0.999997	Node-00042 - Long	1.000027	Node-00042 - Long	1.000106
Node-00042 - Lat	0.999997	Node-00042 - Lat	1.00002	Node-00042 - Lat	1.000095
Node-00043 - Long	0.999973	Node-00043 - Long	1.000082	Node-00043 - Long	1.000109
Node-00043 - Lat	1.000026	Node-00043 - Lat	1.000013	Node-00043 - Lat	1.000114
Node-00044 - Long	0.999994	Node-00044 - Long	1.000067	Node-00044 - Long	1.000094

Node-00044 - Lat	1.000012	Node-00044 - Lat	1	Node-00044 - Lat	1.000066
Node-00045 - Long	0.99999	Node-00045 - Long	1.000042	Node-00045 - Long	1.000037
Node-00045 - Lat	0.999996	Node-00045 - Lat	1.000007	Node-00045 - Lat	1.000054
Node-00046 - Long	1.000015	Node-00046 - Long	0.999969	Node-00046 - Long	0.999995
Node-00046 - Lat	1.000013	Node-00046 - Lat	1.000015	Node-00046 - Lat	1.000026
Node-00047 - Long	1.000044	Node-00047 - Long	1.000044	Node-00047 - Long	1.000014
Node-00047 - Lat	1.00001	Node-00047 - Lat	0.999999	Node-00047 - Lat	1.000038
Node-00048 - Long	1.000054	Node-00048 - Long	NA	Node-00048 - Long	NA
Node-00048 - Lat	1.000031	Node-00048 - Lat	NA	Node-00048 - Lat	NA
Node-00049 - Long	NA	Node-00049 - Long	NA	Node-00049 - Long	NA
Node-00049 - Lat	NA	Node-00049 - Lat	NA	Node-00049 - Lat	NA
Node-00050 - Long	NA	Node-00050 - Long	NA	Node-00050 - Long	NA
Node-00050 - Lat	NA	Node-00050 - Lat	NA	Node-00050 - Lat	NA
Node-00051 - Long	NA	Node-00051 - Long	1.000074	Node-00051 - Long	1.000027
Node-00051 - Lat	NA	Node-00051 - Lat	1.000017	Node-00051 - Lat	1.000024
Node-00052 - Long	1.000031	Node-00052 - Long	1	Node-00052 - Long	1.000059
Node-00052 - Lat	1.000039	Node-00052 - Lat	1.000006	Node-00052 - Lat	1.000038
Node-00053 - Long	1.000034	Node-00053 - Long	0.99999	Node-00053 - Long	1.000057
Node-00053 - Lat	1.000057	Node-00053 - Lat	1.000009	Node-00053 - Lat	1.000009
Node-00054 - Long	1.000014	Node-00054 - Long	0.99998	Node-00054 - Long	1.000016
Node-00054 - Lat	1.000104	Node-00054 - Lat	0.999995	Node-00054 - Lat	1.000068
Node-00055 - Long	1.000073	Node-00055 - Long	0.999986	Node-00055 - Long	0.999986
Node-00055 - Lat	1.000115	Node-00055 - Lat	0.999996	Node-00055 - Lat	1.000073
Node-00056 - Long	1.000087	Node-00056 - Long	0.999961	Node-00056 - Long	0.999979
Node-00056 - Lat	1.000064	Node-00056 - Lat	1.000078	Node-00056 - Lat	1.000107
Node-00057 - Long	0.999995	Node-00057 - Long	1	Node-00057 - Long	1.000012
Node-00057 - Lat	1.000045	Node-00057 - Lat	1.000046	Node-00057 - Lat	0.999986
Node-00058 - Long	0.999982	Node-00058 - Long	NA	Node-00058 - Long	NA
Node-00058 - Lat	0.999984	Node-00058 - Lat	NA	Node-00058 - Lat	NA
Node-00059 - Long	NA	Node-00059 - Long	NA	Node-00059 - Long	NA
Node-00059 - Lat	NA	Node-00059 - Lat	NA	Node-00059 - Lat	NA
Node-00060 - Long	NA	Node-00060 - Long	NA	Node-00060 - Long	NA
Node-00060 - Lat	NA	Node-00060 - Lat	NA	Node-00060 - Lat	NA
Node-00061 - Long	NA	Node-00061 - Long	1.000016	Node-00061 - Long	0.999998
Node-00061 - Lat	NA	Node-00061 - Lat	1.000037	Node-00061 - Lat	1.000058
Node-00062 - Long	0.999999	Node-00062 - Long	1.000035	Node-00062 - Long	1.000013
Node-00062 - Lat	1.000039	Node-00062 - Lat	0.999998	Node-00062 - Lat	1.00005
Node-00063 - Long	0.999983	Node-00063 - Long	1.000042	Node-00063 - Long	0.999999
Node-00063 - Lat	1.000061	Node-00063 - Lat	1.000027	Node-00063 - Lat	1.000031
Node-00064 - Long	1.000056	Node-00064 - Long	1.000054	Node-00064 - Long	0.999984
Node-00064 - Lat	1.000038	Node-00064 - Lat	1.000066	Node-00064 - Lat	1.000009
Node-00065 - Long	0.999973	Node-00065 - Long	1.000035	Node-00065 - Long	1.000054
Node-00065 - Lat	1.000031	Node-00065 - Lat	1	Node-00065 - Lat	0.999987
Node-00066 - Long	0.999962	Node-00066 - Long	1.000038	Node-00066 - Long	1.000006
Node-00066 - Lat	1.000077	Node-00066 - Lat	1.000027	Node-00066 - Lat	1.000027
Node-00067 - Long	1.000061	Node-00067 - Long	NA	Node-00067 - Long	NA
Node-00067 - Lat	1.000079	Node-00067 - Lat	NA	Node-00067 - Lat	NA
Node-00068 - Long	NA	Node-00068 - Long	NA	Node-00068 - Long	NA

Node-00068 - Lat	NA	Node-00068 - Lat	NA	Node-00068 - Lat	NA
Node-00069 - Long	NA	Node-00069 - Long	NA	Node-00069 - Long	NA
Node-00069 - Lat	NA	Node-00069 - Lat	NA	Node-00069 - Lat	NA
Node-00070 - Long	NA	Node-00070 - Long	NA	Node-00070 - Long	NA
Node-00070 - Lat	NA	Node-00070 - Lat	NA	Node-00070 - Lat	NA
Node-00071 - Long	NA	Node-00071 - Long	NA	Node-00071 - Long	NA
Node-00071 - Lat	NA	Node-00071 - Lat	NA	Node-00071 - Lat	NA
Node-00072 - Long	NA	Node-00072 - Long	NA	Node-00072 - Long	NA
Node-00072 - Lat	NA	Node-00072 - Lat	NA	Node-00072 - Lat	NA
Node-00073 - Long	NA	Node-00073 - Long	1.000078	Node-00073 - Long	1.000043
Node-00073 - Lat	NA	Node-00073 - Lat	1.000042	Node-00073 - Lat	1.000062
Node-00074 - Long	1.00002	Node-00074 - Long	1.00007	Node-00074 - Long	0.99999
Node-00074 - Lat	1.00005	Node-00074 - Lat	0.999996	Node-00074 - Lat	1.000013
Node-00075 - Long	1	Node-00075 - Long	1.000136	Node-00075 - Long	1.000046
Node-00075 - Lat	1.00003	Node-00075 - Lat	1.000021	Node-00075 - Lat	0.999984
Node-00076 - Long	1.000147	Node-00076 - Long	NA	Node-00076 - Long	NA
Node-00076 - Lat	1.000046	Node-00076 - Lat	NA	Node-00076 - Lat	NA
Node-00077 - Long	NA	Node-00077 - Long	NA	Node-00077 - Long	NA
Node-00077 - Lat	NA	Node-00077 - Lat	NA	Node-00077 - Lat	NA
Node-00078 - Long	NA	Node-00078 - Long	NA	Node-00078 - Long	NA
Node-00078 - Lat	NA	Node-00078 - Lat	NA	Node-00078 - Lat	NA
Node-00079 - Long	NA	Node-00079 - Long	NA	Node-00079 - Long	NA
Node-00079 - Lat	NA	Node-00079 - Lat	NA	Node-00079 - Lat	NA
Node-00080 - Long	NA	Node-00080 - Long	NA	Node-00080 - Long	NA
Node-00080 - Lat	NA	Node-00080 - Lat	NA	Node-00080 - Lat	NA
Node-00081 - Long	NA	Node-00081 - Long	NA	Node-00081 - Long	NA
Node-00081 - Lat	NA	Node-00081 - Lat	NA	Node-00081 - Lat	NA
Node-00082 - Long	NA	Node-00082 - Long	1.000012	Node-00082 - Long	1.000025
Node-00082 - Lat	NA	Node-00082 - Lat	0.999983	Node-00082 - Lat	1.000002
Node-00083 - Long	0.999975	Node-00083 - Long	1.000004	Node-00083 - Long	1.000025
Node-00083 - Lat	1.000049	Node-00083 - Lat	1.00004	Node-00083 - Lat	1.000003
Node-00084 - Long	1.000028	Node-00084 - Long	NA	Node-00084 - Long	NA
Node-00084 - Lat	0.999975	Node-00084 - Lat	NA	Node-00084 - Lat	NA
Node-00085 - Long	NA	Node-00085 - Long	NA	Node-00085 - Long	NA
Node-00085 - Lat	NA	Node-00085 - Lat	NA	Node-00085 - Lat	NA
Node-00086 - Long	NA	Node-00086 - Long	1.000048	Node-00086 - Long	1.000034
Node-00086 - Lat	NA	Node-00086 - Lat	0.999982	Node-00086 - Lat	0.999969
Node-00087 - Long	1.000019	Node-00087 - Long	NA	Node-00087 - Long	NA
Node-00087 - Lat	1.000038	Node-00087 - Lat	NA	Node-00087 - Lat	NA
Node-00088 - Long	NA	Node-00088 - Long	NA	Node-00088 - Long	NA
Node-00088 - Lat	NA	Node-00088 - Lat	NA	Node-00088 - Lat	NA
Node-00089 - Long	NA	Node-00089 - Long	NA	Node-00089 - Long	NA
Node-00089 - Lat	NA	Node-00089 - Lat	NA	Node-00089 - Lat	NA
Node-00090 - Long	NA	Node-00090 - Long	1.000033	Node-00090 - Long	1.00003
Node-00090 - Lat	NA	Node-00090 - Lat	0.999983	Node-00090 - Lat	1.000033
Node-00091 - Long	1.000002	Node-00091 - Long	NA	Node-00091 - Long	NA
Node-00091 - Lat	1.000062	Node-00091 - Lat	NA	Node-00091 - Lat	NA
Node-00092 - Long	NA	Node-00092 - Long	1.000042	Node-00092 - Long	0.999992

Node-00092 - Lat	NA	Node-00092 - Lat	1.000015	Node-00092 - Lat	1.00003
Node-00093 - Long	0.99997	Node-00093 - Long	NA	Node-00093 - Long	NA
Node-00093 - Lat	1.000003	Node-00093 - Lat	NA	Node-00093 - Lat	NA
Node-00094 - Long	NA	Node-00094 - Long	NA	Node-00094 - Long	NA
Node-00094 - Lat	NA	Node-00094 - Lat	NA	Node-00094 - Lat	NA
Node-00095 - Long	NA	Node-00095 - Long	1.000001	Node-00095 - Long	0.999999
Node-00095 - Lat	NA	Node-00095 - Lat	1.000031	Node-00095 - Lat	0.999986
Node-00096 - Long	1.000027	Node-00096 - Long	NA	Node-00096 - Long	NA
Node-00096 - Lat	0.99998	Node-00096 - Lat	NA	Node-00096 - Lat	NA
Node-00097 - Long	NA	Node-00097 - Long	NA	Node-00097 - Long	NA
Node-00097 - Lat	NA	Node-00097 - Lat	NA	Node-00097 - Lat	NA
Node-00098 - Long	NA	Node-00098 - Long	NA	Node-00098 - Long	NA
Node-00098 - Lat	NA	Node-00098 - Lat	NA	Node-00098 - Lat	NA
Node-00099 - Long	NA	Node-00099 - Long	1.000103	Node-00099 - Long	1.000065
Node-00099 - Lat	NA	Node-00099 - Lat	0.999978	Node-00099 - Lat	1.000006
Node-00100 - Long	1.000219	Node-00100 - Long	1.000122	Node-00100 - Long	1.000095
Node-00100 - Lat	0.999993	Node-00100 - Lat	1.000005	Node-00100 - Lat	1.000007
Node-00101 - Long	1.0003	Node-00101 - Long	1.001014	Node-00101 - Long	1.001356
Node-00101 - Lat	1.000036	Node-00101 - Lat	0.999984	Node-00101 - Lat	0.999985
Node-00102 - Long	1.000719	Node-00102 - Long	NA	Node-00102 - Long	NA
Node-00102 - Lat	1.000015	Node-00102 - Lat	NA	Node-00102 - Lat	NA
Node-00103 - Long	NA	Node-00103 - Long	NA	Node-00103 - Long	NA
Node-00103 - Lat	NA	Node-00103 - Lat	NA	Node-00103 - Lat	NA
Node-00104 - Long	NA	Node-00104 - Long	NA	Node-00104 - Long	NA
Node-00104 - Lat	NA	Node-00104 - Lat	NA	Node-00104 - Lat	NA
Node-00105 - Long	NA	Node-00105 - Long	0.999997	Node-00105 - Long	0.999974
Node-00105 - Lat	NA	Node-00105 - Lat	1.000021	Node-00105 - Lat	1.000012
Node-00106 - Long	1.000146	Node-00106 - Long	0.99998	Node-00106 - Long	1.000057
Node-00106 - Lat	1.000072	Node-00106 - Lat	0.99998	Node-00106 - Lat	1.000057
Node-00107 - Long	1.000008	Node-00107 - Long	0.999973	Node-00107 - Long	0.999989
Node-00107 - Lat	1.000008	Node-00107 - Lat	1.000021	Node-00107 - Lat	1.000046
Node-00108 - Long	1.00002	Node-00108 - Long	0.999989	Node-00108 - Long	0.999964
Node-00108 - Lat	1.000003	Node-00108 - Lat	1.000031	Node-00108 - Lat	0.999994
Node-00109 - Long	1.000076	Node-00109 - Long	0.999977	Node-00109 - Long	0.999999
Node-00109 - Lat	0.999962	Node-00109 - Lat	1.000074	Node-00109 - Lat	0.999976
Node-00110 - Long	1.000062	Node-00110 - Long	NA	Node-00110 - Long	NA
Node-00110 - Lat	0.999991	Node-00110 - Lat	NA	Node-00110 - Lat	NA
Node-00111 - Long	NA	Node-00111 - Long	0.999965	Node-00111 - Long	0.999987
Node-00111 - Lat	NA	Node-00111 - Lat	1.000053	Node-00111 - Lat	0.999969
Node-00112 - Long	0.999972	Node-00112 - Long	NA	Node-00112 - Long	NA
Node-00112 - Lat	0.999969	Node-00112 - Lat	NA	Node-00112 - Lat	NA
Node-00113 - Long	NA	Node-00113 - Long	1.000052	Node-00113 - Long	1.000067
Node-00113 - Lat	NA	Node-00113 - Lat	1.000005	Node-00113 - Lat	0.99997
Node-00114 - Long	0.999984	Node-00114 - Long	1.000085	Node-00114 - Long	1.000051
Node-00114 - Lat	1.000074	Node-00114 - Lat	1.000002	Node-00114 - Lat	1.000029
Node-00115 - Long	1.000035	Node-00115 - Long	1.000007	Node-00115 - Long	0.99999
Node-00115 - Lat	1.000032	Node-00115 - Lat	0.999979	Node-00115 - Lat	1.000102
Node-00116 - Long	1.000002	Node-00116 - Long	NA	Node-00116 - Long	NA

Node-00116 - Lat	1.000054	Node-00116 - Lat	NA	Node-00116 - Lat	NA
Node-00117 - Long	NA	Node-00117 - Long	NA	Node-00117 - Long	NA
Node-00117 - Lat	NA	Node-00117 - Lat	NA	Node-00117 - Lat	NA
Node-00118 - Long	NA	Node-00118 - Long	1.000099	Node-00118 - Long	1.000015
Node-00118 - Lat	NA	Node-00118 - Lat	1.000029	Node-00118 - Lat	1.00007
Node-00119 - Long	0.999995	Node-00119 - Long	1.000059	Node-00119 - Long	0.999975
Node-00119 - Lat	1	Node-00119 - Lat	1.000098	Node-00119 - Lat	1.000043
Node-00120 - Long	1.000004	Node-00120 - Long	NA	Node-00120 - Long	NA
Node-00120 - Lat	1.000028	Node-00120 - Lat	NA	Node-00120 - Lat	NA
Node-00121 - Long	NA	Node-00121 - Long	NA	Node-00121 - Long	NA
Node-00121 - Lat	NA	Node-00121 - Lat	NA	Node-00121 - Lat	NA
Node-00122 - Long	NA	Node-00122 - Long	1.00002	Node-00122 - Long	1.000022
Node-00122 - Lat	NA	Node-00122 - Lat	1.000062	Node-00122 - Lat	0.999985
Node-00123 - Long	1.000023	Node-00123 - Long	NA	Node-00123 - Long	NA
Node-00123 - Lat	1.000022	Node-00123 - Lat	NA	Node-00123 - Lat	NA
Node-00124 - Long	NA	Node-00124 - Long	NA	Node-00124 - Long	NA
Node-00124 - Lat	NA	Node-00124 - Lat	NA	Node-00124 - Lat	NA
Node-00125 - Long	NA	Node-00125 - Long	1.000034	Node-00125 - Long	0.999991
Node-00125 - Lat	NA	Node-00125 - Lat	1.000086	Node-00125 - Lat	1.000051
Node-00126 - Long	1.000018	Node-00126 - Long	1.000061	Node-00126 - Long	0.999988
Node-00126 - Lat	0.999963	Node-00126 - Lat	1.000061	Node-00126 - Lat	0.999988
Node-00127 - Long	1.000007	Node-00127 - Long	NA	Node-00127 - Long	NA
Node-00127 - Lat	1.000007	Node-00127 - Lat	NA	Node-00127 - Lat	NA
Node-00128 - Long	NA	Node-00128 - Long	1.00012	Node-00128 - Long	1.000021
Node-00128 - Lat	NA	Node-00128 - Lat	1.000031	Node-00128 - Lat	1.000065
Node-00129 - Long	1.000032	Node-00129 - Long	1.000042	Node-00129 - Long	1.000012
Node-00129 - Lat	1.000033	Node-00129 - Lat	1.000042	Node-00129 - Lat	1.000012
Node-00130 - Long	0.999989	Node-00130 - Long	0.999999	Node-00130 - Long	0.999994
Node-00130 - Lat	0.999989	Node-00130 - Lat	1.000065	Node-00130 - Lat	1.000084
Node-00131 - Long	1.000078	Node-00131 - Long	NA	Node-00131 - Long	NA
Node-00131 - Lat	1.00004	Node-00131 - Lat	NA	Node-00131 - Lat	NA
Node-00132 - Long	NA	Node-00132 - Long	NA	Node-00132 - Long	NA
Node-00132 - Lat	NA	Node-00132 - Lat	NA	Node-00132 - Lat	NA
Node-00133 - Long	NA	Node-00133 - Long	1.000124	Node-00133 - Long	0.999982
Node-00133 - Lat	NA	Node-00133 - Lat	0.999994	Node-00133 - Lat	1.000026
Node-00134 - Long	0.999976	Node-00134 - Long	NA	Node-00134 - Long	NA
Node-00134 - Lat	1.000034	Node-00134 - Lat	NA	Node-00134 - Lat	NA
Node-00135 - Long	NA	Node-00135 - Long	1.000069	Node-00135 - Long	1.00001
Node-00135 - Lat	NA	Node-00135 - Lat	1.000025	Node-00135 - Lat	0.999989
Node-00136 - Long	1.000008	Node-00136 - Long	NA	Node-00136 - Long	NA
Node-00136 - Lat	1.000042	Node-00136 - Lat	NA	Node-00136 - Lat	NA
Node-00137 - Long	NA	Node-00137 - Long	NA	Node-00137 - Long	NA
Node-00137 - Lat	NA	Node-00137 - Lat	NA	Node-00137 - Lat	NA
Node-00138 - Long	NA	Node-00138 - Long	1.000024	Node-00138 - Long	1.000011
Node-00138 - Lat	NA	Node-00138 - Lat	1.000016	Node-00138 - Lat	0.999991
Node-00139 - Long	0.999992	Node-00139 - Long	NA	Node-00139 - Long	NA
Node-00139 - Lat	1.00002	Node-00139 - Lat	NA	Node-00139 - Lat	NA
Node-00140 - Long	NA	Node-00140 - Long	NA	Node-00140 - Long	NA

Node-00140 - Lat	NA	Node-00140 - Lat	NA	Node-00140 - Lat	NA
Node-00141 - Long	NA	Node-00141 - Long	1.000103	Node-00141 - Long	0.99998
Node-00141 - Lat	NA	Node-00141 - Lat	1.000076	Node-00141 - Lat	1.000053
Node-00142 - Long	0.999989	Node-00142 - Long	NA	Node-00142 - Long	NA
Node-00142 - Lat	0.999995	Node-00142 - Lat	NA	Node-00142 - Lat	NA
Node-00143 - Long	NA	Node-00143 - Long	NA	Node-00143 - Long	NA
Node-00143 - Lat	NA	Node-00143 - Lat	NA	Node-00143 - Lat	NA
Node-00144 - Long	NA	Node-00144 - Long	1.000019	Node-00144 - Long	1.000031
Node-00144 - Lat	NA	Node-00144 - Lat	0.999984	Node-00144 - Lat	1.000002
Node-00145 - Long	1.000084	Node-00145 - Long	NA	Node-00145 - Long	NA
Node-00145 - Lat	1.000113	Node-00145 - Lat	NA	Node-00145 - Lat	NA
Node-00146 - Long	NA	Node-00146 - Long	NA	Node-00146 - Long	NA
Node-00146 - Lat	NA	Node-00146 - Lat	NA	Node-00146 - Lat	NA
Node-00147 - Long	NA	Node-00147 - Long	0.999986	Node-00147 - Long	1.000015
Node-00147 - Lat	NA	Node-00147 - Lat	1.000022	Node-00147 - Lat	1.000016
Node-00148 - Long	0.999978	Node-00148 - Long	0.999993	Node-00148 - Long	1.00007
Node-00148 - Lat	0.999973	Node-00148 - Lat	1.000055	Node-00148 - Lat	1.000016
Node-00149 - Long	1.000075	Node-00149 - Long	1.000028	Node-00149 - Long	1.000118
Node-00149 - Lat	0.999968	Node-00149 - Lat	1.000024	Node-00149 - Lat	1.000091
Node-00150 - Long	1.000029	Node-00150 - Long	NA	Node-00150 - Long	NA
Node-00150 - Lat	0.999983	Node-00150 - Lat	NA	Node-00150 - Lat	NA
Node-00151 - Long	NA	Node-00151 - Long	NA	Node-00151 - Long	NA
Node-00151 - Lat	NA	Node-00151 - Lat	NA	Node-00151 - Lat	NA
Node-00152 - Long	NA	Node-00152 - Long	NA	Node-00152 - Long	NA
Node-00152 - Lat	NA	Node-00152 - Lat	NA	Node-00152 - Lat	NA
Node-00153 - Long	NA	Node-00153 - Long	NA	Node-00153 - Long	NA
Node-00153 - Lat	NA	Node-00153 - Lat	NA	Node-00153 - Lat	NA
Node-00154 - Long	NA	Node-00154 - Long	NA	Node-00154 - Long	NA
Node-00154 - Lat	NA	Node-00154 - Lat	NA	Node-00154 - Lat	NA
Node-00155 - Long	NA	Node-00155 - Long	1.000032	Node-00155 - Long	1.000036
Node-00155 - Lat	NA	Node-00155 - Lat	1.000026	Node-00155 - Lat	0.999989
Node-00156 - Long	1.000064	Node-00156 - Long	NA	Node-00156 - Long	NA
Node-00156 - Lat	0.999996	Node-00156 - Lat	NA	Node-00156 - Lat	NA
Node-00157 - Long	NA	Node-00157 - Long	NA	Node-00157 - Long	NA
Node-00157 - Lat	NA	Node-00157 - Lat	NA	Node-00157 - Lat	NA
Node-00158 - Long	NA	Node-00158 - Long	1.000022	Node-00158 - Long	1.000067
Node-00158 - Lat	NA	Node-00158 - Lat	0.99996	Node-00158 - Lat	0.999985
Node-00159 - Long	0.99999	Node-00159 - Long	1.000005	Node-00159 - Long	0.999974
Node-00159 - Lat	0.999999	Node-00159 - Lat	1.000004	Node-00159 - Lat	1.000013
Node-00160 - Long	1.000036	Node-00160 - Long	NA	Node-00160 - Long	NA
Node-00160 - Lat	1.000014	Node-00160 - Lat	NA	Node-00160 - Lat	NA
Node-00161 - Long	NA	Node-00161 - Long	1.000022	Node-00161 - Long	0.999979
Node-00161 - Lat	NA	Node-00161 - Lat	0.999991	Node-00161 - Lat	1.000039
Node-00162 - Long	1.000005	Node-00162 - Long	1.000004	Node-00162 - Long	1.000012
Node-00162 - Lat	1.000006	Node-00162 - Lat	1.000021	Node-00162 - Lat	1.000024
Node-00163 - Long	1.000019	Node-00163 - Long	1.000031	Node-00163 - Long	1.000032
Node-00163 - Lat	0.999988	Node-00163 - Lat	1.000003	Node-00163 - Lat	1.000024
Node-00164 - Long	0.999986	Node-00164 - Long	1.000023	Node-00164 - Long	1.000021

Node-00164 - Lat	1	Node-00164 - Lat	0.999989	Node-00164 - Lat	1.000043
Node-00165 - Long	1.000064	Node-00165 - Long	0.999995	Node-00165 - Long	1.000032
Node-00165 - Lat	1.000038	Node-00165 - Lat	0.999967	Node-00165 - Lat	0.999987
Node-00166 - Long	1.000124	Node-00166 - Long	1.000035	Node-00166 - Long	1.000079
Node-00166 - Lat	1.000009	Node-00166 - Lat	0.999982	Node-00166 - Lat	0.999996
Node-00167 - Long	1.000051	Node-00167 - Long	1.000002	Node-00167 - Long	1.000054
Node-00167 - Lat	0.999987	Node-00167 - Lat	0.999993	Node-00167 - Lat	0.999991
Node-00168 - Long	1.000045	Node-00168 - Long	1.00004	Node-00168 - Long	1.000054
Node-00168 - Lat	0.999984	Node-00168 - Lat	1.000047	Node-00168 - Lat	1.000043
Node-00169 - Long	1.000024	Node-00169 - Long	1.00001	Node-00169 - Long	1.000065
Node-00169 - Lat	0.999966	Node-00169 - Lat	1.000105	Node-00169 - Lat	1.000016
Node-00170 - Long	1.000072	Node-00170 - Long	1.000047	Node-00170 - Long	1.000054
Node-00170 - Lat	0.999972	Node-00170 - Lat	1.000083	Node-00170 - Lat	1.000011
Node-00171 - Long	0.999998	Node-00171 - Long	0.999993	Node-00171 - Long	1.000047
Node-00171 - Lat	1.000031	Node-00171 - Lat	1.000019	Node-00171 - Lat	1.000014
Node-00172 - Long	0.999993	Node-00172 - Long	1.000015	Node-00172 - Long	1.000031
Node-00172 - Lat	1.000001	Node-00172 - Lat	1.000035	Node-00172 - Lat	1.000092
Node-00173 - Long	1	Node-00173 - Long	1.000019	Node-00173 - Long	1.000047
Node-00173 - Lat	0.999989	Node-00173 - Lat	1.000096	Node-00173 - Lat	1.000105
Node-00174 - Long	1.000043	Node-00174 - Long	0.999985	Node-00174 - Long	1.000006
Node-00174 - Lat	1.000016	Node-00174 - Lat	1.00004	Node-00174 - Lat	1.000061
Node-00175 - Long	1.000062	Node-00175 - Long	1.000006	Node-00175 - Long	1.000014
Node-00175 - Lat	1.000023	Node-00175 - Lat	1.000031	Node-00175 - Lat	1.000057
Node-00176 - Long	1.000028	Node-00176 - Long	NA	Node-00176 - Long	NA
Node-00176 - Lat	1.000011	Node-00176 - Lat	NA	Node-00176 - Lat	NA
Node-00177 - Long	NA	Node-00177 - Long	0.999995	Node-00177 - Long	1.000042
Node-00177 - Lat	NA	Node-00177 - Lat	1.00001	Node-00177 - Lat	1.00001
Node-00178 - Long	0.999999	Node-00178 - Long	NA	Node-00178 - Long	NA
Node-00178 - Lat	1.000024	Node-00178 - Lat	NA	Node-00178 - Lat	NA
Node-00179 - Long	NA	Node-00179 - Long	NA	Node-00179 - Long	NA
Node-00179 - Lat	NA	Node-00179 - Lat	NA	Node-00179 - Lat	NA
Node-00180 - Long	NA	Node-00180 - Long	1.00001	Node-00180 - Long	0.99999
Node-00180 - Lat	NA	Node-00180 - Lat	0.999974	Node-00180 - Lat	1.000089
Node-00181 - Long	0.999958	Node-00181 - Long	1.000185	Node-00181 - Long	0.999972
Node-00181 - Lat	1.00002	Node-00181 - Lat	1.000087	Node-00181 - Lat	0.999966
Node-00182 - Long	1.000113	Node-00182 - Long	NA	Node-00182 - Long	NA
Node-00182 - Lat	1.00006	Node-00182 - Lat	NA	Node-00182 - Lat	NA
Node-00183 - Long	NA	Node-00183 - Long	NA	Node-00183 - Long	NA
Node-00183 - Lat	NA	Node-00183 - Lat	NA	Node-00183 - Lat	NA
Node-00184 - Long	NA	Node-00184 - Long	NA	Node-00184 - Long	NA
Node-00184 - Lat	NA	Node-00184 - Lat	NA	Node-00184 - Lat	NA
Node-00185 - Long	NA	Node-00185 - Long	NA	Node-00185 - Long	NA
Node-00185 - Lat	NA	Node-00185 - Lat	NA	Node-00185 - Lat	NA
Node-00186 - Long	NA	Node-00186 - Long	NA	Node-00186 - Long	NA
Node-00186 - Lat	NA	Node-00186 - Lat	NA	Node-00186 - Lat	NA
Node-00187 - Long	NA	Node-00187 - Long	0.999982	Node-00187 - Long	0.999969
Node-00187 - Lat	NA	Node-00187 - Lat	1.000047	Node-00187 - Lat	1.000015
Node-00188 - Long	0.999972	Node-00188 - Long	NA	Node-00188 - Long	NA

Node-00188 - Lat	1.000018	Node-00188 - Lat	NA	Node-00188 - Lat	NA
Node-00189 - Long	NA	Node-00189 - Long	1.000034	Node-00189 - Long	1.000034
Node-00189 - Lat	NA	Node-00189 - Lat	1.000049	Node-00189 - Lat	1.000029
Node-00190 - Long	0.999993	Node-00190 - Long	0.999988	Node-00190 - Long	1.000019
Node-00190 - Lat	1.000008	Node-00190 - Lat	1.00002	Node-00190 - Lat	1.000007
Node-00191 - Long	1.000074	Node-00191 - Long	NA	Node-00191 - Long	NA
Node-00191 - Lat	1.000058	Node-00191 - Lat	NA	Node-00191 - Lat	NA
Node-00192 - Long	NA	Node-00192 - Long	NA	Node-00192 - Long	NA
Node-00192 - Lat	NA	Node-00192 - Lat	NA	Node-00192 - Lat	NA
Node-00193 - Long	NA	Node-00193 - Long	1.000073	Node-00193 - Long	0.999998
Node-00193 - Lat	NA	Node-00193 - Lat	0.999986	Node-00193 - Lat	0.999991
Node-00194 - Long	0.999981	Node-00194 - Long	0.999977	Node-00194 - Long	1.000006
Node-00194 - Lat	1.000017	Node-00194 - Lat	1.000024	Node-00194 - Lat	1.000013
Node-00195 - Long	0.999974	Node-00195 - Long	1.000009	Node-00195 - Long	1.000067
Node-00195 - Lat	0.999991	Node-00195 - Lat	0.999993	Node-00195 - Lat	0.999994
Node-00196 - Long	1.000033	Node-00196 - Long	1.000082	Node-00196 - Long	0.999997
Node-00196 - Lat	1.000036	Node-00196 - Lat	1.000096	Node-00196 - Lat	1.000072
Node-00197 - Long	1.000062	Node-00197 - Long	1.000024	Node-00197 - Long	1.000018
Node-00197 - Lat	0.999989	Node-00197 - Lat	1.000079	Node-00197 - Lat	1.00004
Node-00198 - Long	1.000038	Node-00198 - Long	NA	Node-00198 - Long	NA
Node-00198 - Lat	0.999999	Node-00198 - Lat	NA	Node-00198 - Lat	NA
Node-00199 - Long	NA	Node-00199 - Long	1.001567	Node-00199 - Long	1.005639
Node-00199 - Lat	NA	Node-00199 - Lat	1.001501	Node-00199 - Lat	1.005823
Node-00200 - Long	1.000459	Node-00200 - Long	NA	Node-00200 - Long	NA
Node-00200 - Lat	1.00039	Node-00200 - Lat	NA	Node-00200 - Lat	NA
Node-00201 - Long	NA	Node-00201 - Long	NA	Node-00201 - Long	NA
Node-00201 - Lat	NA	Node-00201 - Lat	NA	Node-00201 - Lat	NA
Node-00202 - Long	NA	Node-00202 - Long	1.000002	Node-00202 - Long	1.000044
Node-00202 - Lat	NA	Node-00202 - Lat	1.000078	Node-00202 - Lat	1.000019
Node-00203 - Long	1.000043	Node-00203 - Long	1.000026	Node-00203 - Long	1.000043
Node-00203 - Lat	1.000065	Node-00203 - Lat	1.000026	Node-00203 - Lat	1.000043
Node-00204 - Long	1.000004	Node-00204 - Long	NA	Node-00204 - Long	NA
Node-00204 - Lat	1.000004	Node-00204 - Lat	NA	Node-00204 - Lat	NA
Node-00205 - Long	NA	Node-00205 - Long	NA	Node-00205 - Long	NA
Node-00205 - Lat	NA	Node-00205 - Lat	NA	Node-00205 - Lat	NA
Node-00206 - Long	NA	Node-00206 - Long	1.00002	Node-00206 - Long	1.00006
Node-00206 - Lat	NA	Node-00206 - Lat	1.00002	Node-00206 - Lat	1.00006
Node-00207 - Long	0.999965	Node-00207 - Long	NA	Node-00207 - Long	NA
Node-00207 - Lat	0.999965	Node-00207 - Lat	NA	Node-00207 - Lat	NA
Node-00208 - Long	NA	Node-00208 - Long	NA	Node-00208 - Long	NA
Node-00208 - Lat	NA	Node-00208 - Lat	NA	Node-00208 - Lat	NA
Node-00209 - Long	NA	Node-00209 - Long	NA	Node-00209 - Long	NA
Node-00209 - Lat	NA	Node-00209 - Lat	NA	Node-00209 - Lat	NA
Node-00210 - Long	NA	Node-00210 - Long	NA	Node-00210 - Long	NA
Node-00210 - Lat	NA	Node-00210 - Lat	NA	Node-00210 - Lat	NA
Node-00211 - Long	NA	Node-00211 - Long	0.999996	Node-00211 - Long	1.000086
Node-00211 - Lat	NA	Node-00211 - Lat	0.999996	Node-00211 - Lat	1.000086
Node-00212 - Long	1.000029	Node-00212 - Long	NA	Node-00212 - Long	NA

Node-00212 - Lat	1.000029	Node-00212 - Lat	NA	Node-00212 - Lat	NA
Node-00213 - Long	NA	Node-00213 - Long	1.000061	Node-00213 - Long	0.999999
Node-00213 - Lat	NA	Node-00213 - Lat	1.000022	Node-00213 - Lat	1.000011
Node-00214 - Long	0.999987	Node-00214 - Long	1.000039	Node-00214 - Long	1.000017
Node-00214 - Lat	1.000001	Node-00214 - Lat	1.000026	Node-00214 - Lat	1.000005
Node-00215 - Long	0.999976	Node-00215 - Long	1.000002	Node-00215 - Long	1.000002
Node-00215 - Lat	0.999976	Node-00215 - Lat	1.000091	Node-00215 - Lat	0.99999
Node-00216 - Long	0.999991	Node-00216 - Long	0.999998	Node-00216 - Long	1.00004
Node-00216 - Lat	1.00004	Node-00216 - Lat	1.000017	Node-00216 - Lat	1.000075
Node-00217 - Long	0.999969	Node-00217 - Long	NA	Node-00217 - Long	NA
Node-00217 - Lat	1.000114	Node-00217 - Lat	NA	Node-00217 - Lat	NA
Node-00218 - Long	NA	Node-00218 - Long	NA	Node-00218 - Long	NA
Node-00218 - Lat	NA	Node-00218 - Lat	NA	Node-00218 - Lat	NA
Node-00219 - Long	NA	Node-00219 - Long	0.999989	Node-00219 - Long	1.000002
Node-00219 - Lat	NA	Node-00219 - Lat	1.000026	Node-00219 - Lat	1.000009
Node-00220 - Long	1.000025	Node-00220 - Long	NA	Node-00220 - Long	NA
Node-00220 - Lat	0.999993	Node-00220 - Lat	NA	Node-00220 - Lat	NA
Node-00221 - Long	NA	Node-00221 - Long	0.999969	Node-00221 - Long	0.999965
Node-00221 - Lat	NA	Node-00221 - Lat	0.999969	Node-00221 - Lat	0.999965
Node-00222 - Long	1.000046	Node-00222 - Long	NA	Node-00222 - Long	NA
Node-00222 - Lat	1.000046	Node-00222 - Lat	NA	Node-00222 - Lat	NA
Node-00223 - Long	NA	Node-00223 - Long	NA	Node-00223 - Long	NA
Node-00223 - Lat	NA	Node-00223 - Lat	NA	Node-00223 - Lat	NA
Node-00224 - Long	NA	Node-00224 - Long	NA	Node-00224 - Long	NA
Node-00224 - Lat	NA	Node-00224 - Lat	NA	Node-00224 - Lat	NA
Node-00225 - Long	NA	Node-00225 - Long	NA	Node-00225 - Long	NA
Node-00225 - Lat	NA	Node-00225 - Lat	NA	Node-00225 - Lat	NA
Node-00226 - Long	NA	Node-00226 - Long	1.000057	Node-00226 - Long	1.000062
Node-00226 - Lat	NA	Node-00226 - Lat	1.000026	Node-00226 - Lat	1.000014
Node-00227 - Long	1.000091	Node-00227 - Long	0.99999	Node-00227 - Long	1.000002
Node-00227 - Lat	1.000043	Node-00227 - Lat	1.000038	Node-00227 - Lat	1.000012
Node-00228 - Long	1.000001	Node-00228 - Long	1.000015	Node-00228 - Long	0.999968
Node-00228 - Lat	1.000017	Node-00228 - Lat	1.000047	Node-00228 - Lat	0.999986
Node-00229 - Long	0.999983	Node-00229 - Long	0.999997	Node-00229 - Long	1.000067
Node-00229 - Lat	1.000016	Node-00229 - Lat	1.000048	Node-00229 - Lat	1.000028
Node-00230 - Long	1.000057	Node-00230 - Long	NA	Node-00230 - Long	0.999989
Node-00230 - Lat	0.999995	Node-00230 - Lat	NA	Node-00230 - Lat	1.000064
Node-00231 - Long	NA	Node-00231 - Long	NA	Node-00231 - Long	0.999978
Node-00231 - Lat	NA	Node-00231 - Lat	NA	Node-00231 - Lat	1.000033
Node-00232 - Long	NA	Node-00232 - Long	NA	Node-00232 - Long	0.999994
Node-00232 - Lat	NA	Node-00232 - Lat	NA	Node-00232 - Lat	1.000031
Node-00233 - Long	NA	Node-00233 - Long	1.000001	Node-00233 - Long	1.000002
Node-00233 - Lat	NA	Node-00233 - Lat	1.000085	Node-00233 - Lat	1.000027
Node-00234 - Long	0.999996	Node-00234 - Long	NA	Node-00234 - Long	1.000112
Node-00234 - Lat	1.00001	Node-00234 - Lat	NA	Node-00234 - Lat	1.000061
Node-00235 - Long	NA	Node-00235 - Long	NA	Node-00235 - Long	1.000013
Node-00235 - Lat	NA	Node-00235 - Lat	NA	Node-00235 - Lat	1.000026
Node-00236 - Long	NA	Node-00236 - Long	1.000084	Node-00236 - Long	1.000097

Node-00236 - Lat	NA	Node-00236 - Lat	0.99997	Node-00236 - Lat	1.000014
Node-00237 - Long	1.000006	Node-00237 - Long	1.000025	Node-00237 - Long	1.000039
Node-00237 - Lat	0.999984	Node-00237 - Lat	1.000049	Node-00237 - Lat	0.999959
Node-00238 - Long	1.000051	Node-00238 - Long	1.000011	Node-00238 - Long	1.000011
Node-00238 - Lat	1.000036	Node-00238 - Lat	0.999983	Node-00238 - Lat	0.999968
Node-00239 - Long	1.000008	Node-00239 - Long	1.000038	Node-00239 - Long	1.000079
Node-00239 - Lat	1.000028	Node-00239 - Lat	1.000049	Node-00239 - Lat	1.000016
Node-00240 - Long	0.999991	Node-00240 - Long	NA	Node-00240 - Long	0.999983
Node-00240 - Lat	0.999977	Node-00240 - Lat	NA	Node-00240 - Lat	1.00003
Node-00241 - Long	NA	Node-00241 - Long	1.000028	Node-00241 - Long	0.999994
Node-00241 - Lat	NA	Node-00241 - Lat	1.000028	Node-00241 - Lat	0.999972
Node-00242 - Long	1.000027	Node-00242 - Long	0.999985	Node-00242 - Long	NA
Node-00242 - Lat	1.000027	Node-00242 - Lat	1.000035	Node-00242 - Lat	NA
Node-00243 - Long	1.000015	Node-00243 - Long	NA	Node-00243 - Long	NA
Node-00243 - Lat	1.00001	Node-00243 - Lat	NA	Node-00243 - Lat	NA
Node-00244 - Long	NA	Node-00244 - Long	1.000006	Node-00244 - Long	NA
Node-00244 - Lat	NA	Node-00244 - Lat	1.000037	Node-00244 - Lat	NA
Node-00245 - Long	1.000015	Node-00245 - Long	1.000015	Node-00245 - Long	1.000037
Node-00245 - Lat	1.000021	Node-00245 - Lat	1.000015	Node-00245 - Lat	0.999979
Node-00246 - Long	1.00001	Node-00246 - Long	NA	Node-00246 - Long	NA
Node-00246 - Lat	1.00001	Node-00246 - Lat	NA	Node-00246 - Lat	NA
Node-00247 - Long	NA	Node-00247 - Long	NA	Node-00247 - Long	NA
Node-00247 - Lat	NA	Node-00247 - Lat	NA	Node-00247 - Lat	NA
Node-00248 - Long	NA	Node-00248 - Long	1.000012	Node-00248 - Long	0.999982
Node-00248 - Lat	NA	Node-00248 - Lat	0.999979	Node-00248 - Lat	0.999982
Node-00249 - Long	1	Node-00249 - Long	1.000011	Node-00249 - Long	NA
Node-00249 - Lat	1.000025	Node-00249 - Lat	0.999963	Node-00249 - Lat	NA
Node-00250 - Long	1.000038	Node-00250 - Long	1.000019	Node-00250 - Long	0.999995
Node-00250 - Lat	1.000034	Node-00250 - Lat	0.999991	Node-00250 - Lat	0.999971
Node-00251 - Long	1.000045	Node-00251 - Long	NA	Node-00251 - Long	1.000011
Node-00251 - Lat	1.000063	Node-00251 - Lat	NA	Node-00251 - Lat	0.999996
Node-00252 - Long	NA	Node-00252 - Long	0.999982	Node-00252 - Long	NA
Node-00252 - Lat	NA	Node-00252 - Lat	0.999988	Node-00252 - Lat	NA
Node-00253 - Long	1.000012	Node-00253 - Long	0.999995	Node-00253 - Long	1.00003
Node-00253 - Lat	1.000054	Node-00253 - Lat	1.000023	Node-00253 - Lat	1.00003
Node-00254 - Long	0.99998	Node-00254 - Long	1.000072	Node-00254 - Long	1.000035
Node-00254 - Lat	1.00003	Node-00254 - Lat	1.000042	Node-00254 - Lat	1.000048
Node-00255 - Long	0.999969	Node-00255 - Long	NA	Node-00255 - Long	NA
Node-00255 - Lat	1.000017	Node-00255 - Lat	NA	Node-00255 - Lat	NA
Node-00256 - Long	NA	Node-00256 - Long	NA	Node-00256 - Long	1.000086
Node-00256 - Lat	NA	Node-00256 - Lat	NA	Node-00256 - Lat	0.999981
Node-00257 - Long	NA	Node-00257 - Long	0.999981	Node-00257 - Long	NA
Node-00257 - Lat	NA	Node-00257 - Lat	0.999987	Node-00257 - Lat	NA
Node-00258 - Long	1.000009	Node-00258 - Long	1.000068	Node-00258 - Long	0.999977
Node-00258 - Lat	1.000046	Node-00258 - Lat	0.99998	Node-00258 - Lat	0.999977
Node-00259 - Long	1.000073	Node-00259 - Long	1.000006	Node-00259 - Long	1.000007
Node-00259 - Lat	0.999988	Node-00259 - Lat	1.000016	Node-00259 - Lat	0.999995
Node-00260 - Long	1.000074	Node-00260 - Long	0.999994	Node-00260 - Long	NA

Node-00260 - Lat	1.00013	Node-00260 - Lat	0.999994	Node-00260 - Lat	NA
Node-00261 - Long	0.999982	Node-00261 - Long	NA	Node-00261 - Long	NA
Node-00261 - Lat	0.999982	Node-00261 - Lat	NA	Node-00261 - Lat	NA
Node-00262 - Long	NA	Node-00262 - Long	NA	Node-00262 - Long	NA
Node-00262 - Lat	NA	Node-00262 - Lat	NA	Node-00262 - Lat	NA
Node-00263 - Long	NA	Node-00263 - Long	NA	Node-00263 - Long	1.000046
Node-00263 - Lat	NA	Node-00263 - Lat	NA	Node-00263 - Lat	1.00001
Node-00264 - Long	NA	Node-00264 - Long	1.000015	Node-00264 - Long	NA
Node-00264 - Lat	NA	Node-00264 - Lat	1.000134	Node-00264 - Lat	NA
Node-00265 - Long	1	Node-00265 - Long	NA	Node-00265 - Long	NA
Node-00265 - Lat	1.000022	Node-00265 - Lat	NA	Node-00265 - Lat	NA
Node-00266 - Long	NA	Node-00266 - Long	NA	Node-00266 - Long	NA
Node-00266 - Lat	NA	Node-00266 - Lat	NA	Node-00266 - Lat	NA
Node-00267 - Long	NA	Node-00267 - Long	NA	Node-00267 - Long	NA
Node-00267 - Lat	NA	Node-00267 - Lat	NA	Node-00267 - Lat	NA
Node-00268 - Long	NA	Node-00268 - Long	NA	Node-00268 - Long	NA
Node-00268 - Lat	NA	Node-00268 - Lat	NA	Node-00268 - Lat	NA
Node-00269 - Long	NA	Node-00269 - Long	NA	Node-00269 - Long	NA
Node-00269 - Lat	NA	Node-00269 - Lat	NA	Node-00269 - Lat	NA
Node-00270 - Long	NA	Node-00270 - Long	1.00002	Node-00270 - Long	NA
Node-00270 - Lat	NA	Node-00270 - Lat	1.000053	Node-00270 - Lat	NA
Node-00271 - Long	0.999999	Node-00271 - Long	NA	Node-00271 - Long	NA
Node-00271 - Lat	0.999982	Node-00271 - Lat	NA	Node-00271 - Lat	NA
Node-00272 - Long	NA	Node-00272 - Long	NA	Node-00272 - Long	1.000006
Node-00272 - Lat	NA	Node-00272 - Lat	NA	Node-00272 - Lat	1.000013
Node-00273 - Long	NA	Node-00273 - Long	1.000037	Node-00273 - Long	NA
Node-00273 - Lat	NA	Node-00273 - Lat	0.999959	Node-00273 - Lat	NA
Node-00274 - Long	1.000053	Node-00274 - Long	1.000048	Node-00274 - Long	NA
Node-00274 - Lat	1.000007	Node-00274 - Lat	0.999979	Node-00274 - Lat	NA
Node-00275 - Long	1.000071	Node-00275 - Long	1.000029	Node-00275 - Long	1.00002
Node-00275 - Lat	0.999994	Node-00275 - Lat	1.000004	Node-00275 - Lat	1.000039
Node-00276 - Long	1.000125	Node-00276 - Long	1.000064	Node-00276 - Long	1.000031
Node-00276 - Lat	1.000106	Node-00276 - Lat	1.000009	Node-00276 - Lat	0.999999
Node-00277 - Long	0.999991	Node-00277 - Long	0.999989	Node-00277 - Long	1.000015
Node-00277 - Lat	1.000093	Node-00277 - Lat	0.999986	Node-00277 - Lat	1.000007
Node-00278 - Long	1.000035	Node-00278 - Long	1.000083	Node-00278 - Long	1.000046
Node-00278 - Lat	1	Node-00278 - Lat	1.000049	Node-00278 - Lat	1.000031
Node-00279 - Long	1.000161	Node-00279 - Long	NA	Node-00279 - Long	1.000008
Node-00279 - Lat	1.000122	Node-00279 - Lat	NA	Node-00279 - Lat	1.000014
Node-00280 - Long	NA	Node-00280 - Long	1.000005	Node-00280 - Long	1.000268
Node-00280 - Lat	NA	Node-00280 - Lat	1.000014	Node-00280 - Lat	1.000274
Node-00281 - Long	NA	Node-00281 - Long	1.000033	Node-00281 - Long	NA
Node-00281 - Lat	NA	Node-00281 - Lat	NA	Node-00281 - Lat	NA
Node-00282 - Long	NA	Node-00282 - Long	1.000049	Node-00282 - Long	NA
Node-00282 - Lat	NA	Node-00282 - Lat	0.999987	Node-00282 - Lat	NA
Node-00283 - Long	1.000042	Node-00283 - Long	NA	Node-00283 - Long	NA
Node-00283 - Lat	0.999981	Node-00283 - Lat	NA	Node-00283 - Lat	NA
Node-00284 - Long	NA	Node-00284 - Long	0.999963	Node-00284 - Long	1.000026

Node-00284 - Lat	NA	Node-00284 - Lat	1.000007	Node-00284 - Lat	1.00001
Node-00285 - Long	NA	Node-00285 - Long	0.999966	Node-00285 - Long	NA
Node-00285 - Lat	NA	Node-00285 - Lat	0.999964	Node-00285 - Lat	NA
Node-00286 - Long	NA	Node-00286 - Long	NA	Node-00286 - Long	NA
Node-00286 - Lat	NA	Node-00286 - Lat	NA	Node-00286 - Lat	NA
Node-00287 - Long	0.999974	Node-00287 - Long	1.000063	Node-00287 - Long	NA
Node-00287 - Lat	1.000019	Node-00287 - Lat	1.000017	Node-00287 - Lat	NA
Node-00288 - Long	1.000005	Node-00288 - Long	1.000075	Node-00288 - Long	1.000038
Node-00288 - Lat	1.000055	Node-00288 - Lat	1.000017	Node-00288 - Lat	1.000034
Node-00289 - Long	1.000031	Node-00289 - Long	1.000012	Node-00289 - Long	1.000058
Node-00289 - Lat	0.999984	Node-00289 - Lat	0.999983	Node-00289 - Lat	0.999994
Node-00290 - Long	1.000036	Node-00290 - Long	0.999993	Node-00290 - Long	1.000043
Node-00290 - Lat	0.999962	Node-00290 - Lat	1.000055	Node-00290 - Lat	1.000073
Node-00291 - Long	1.00003	Node-00291 - Long	NA	Node-00291 - Long	1.000078
Node-00291 - Lat	0.999996	Node-00291 - Lat	NA	Node-00291 - Lat	1.000025
Node-00292 - Long	NA	Node-00292 - Long	0.999971	Node-00292 - Long	1.000021
Node-00292 - Lat	NA	Node-00292 - Lat	1.000089	Node-00292 - Lat	0.999991
Node-00293 - Long	0.999983	Node-00293 - Long	NA	Node-00293 - Long	NA
Node-00293 - Lat	0.999985	Node-00293 - Lat	NA	Node-00293 - Lat	NA
Node-00294 - Long	1.000021	Node-00294 - Long	0.999983	Node-00294 - Long	1.000031
Node-00294 - Lat	NA	Node-00294 - Lat	1.000001	Node-00294 - Lat	1.000008
Node-00295 - Long	1.000032	Node-00295 - Long	0.999977	Node-00295 - Long	0.999984
Node-00295 - Lat	1.000013	Node-00295 - Lat	1.000013	Node-00295 - Lat	NA
Node-00296 - Long	NA	Node-00296 - Long	0.999994	Node-00296 - Long	1.000074
Node-00296 - Lat	NA	Node-00296 - Lat	0.999994	Node-00296 - Lat	1.000033
Node-00297 - Long	1.000112	Node-00297 - Long	1.000012	Node-00297 - Long	NA
Node-00297 - Lat	1.000128	Node-00297 - Lat	0.999995	Node-00297 - Lat	NA
Node-00298 - Long	1.000139	Node-00298 - Long	NA	Node-00298 - Long	1.000073
Node-00298 - Lat	1.000146	Node-00298 - Lat	NA	Node-00298 - Lat	1.000139
Node-00299 - Long	NA	Node-00299 - Long	1.000025	Node-00299 - Long	1.000237
Node-00299 - Lat	NA	Node-00299 - Lat	0.999998	Node-00299 - Lat	1.000229
Node-00300 - Long	1.000022	Node-00300 - Long	1.000062	Node-00300 - Long	NA
Node-00300 - Lat	1.000054	Node-00300 - Lat	0.999988	Node-00300 - Lat	NA
Node-00301 - Long	0.999997	Node-00301 - Long	NA	Node-00301 - Long	1.000053
Node-00301 - Lat	0.999977	Node-00301 - Lat	NA	Node-00301 - Lat	1.000012
Node-00302 - Long	1.000032	Node-00302 - Long	1.000029	Node-00302 - Long	1.00004
Node-00302 - Lat	1.000001	Node-00302 - Lat	1.000004	Node-00302 - Lat	1.000083
Node-00303 - Long	1.000061	Node-00303 - Long	NA	Node-00303 - Long	1.000004
Node-00303 - Lat	1.000008	Node-00303 - Lat	NA	Node-00303 - Lat	1.000041
Node-00304 - Long	NA	Node-00304 - Long	NA	Node-00304 - Long	1.000003
Node-00304 - Lat	NA	Node-00304 - Lat	NA	Node-00304 - Lat	1.000002
Node-00305 - Long	1.000015	Node-00305 - Long	NA	Node-00305 - Long	NA
Node-00305 - Lat	1.000029	Node-00305 - Lat	NA	Node-00305 - Lat	NA
Node-00306 - Long	NA	Node-00306 - Long	NA	Node-00306 - Long	1.000002
Node-00306 - Lat	NA	Node-00306 - Lat	NA	Node-00306 - Lat	1.000001
Node-00307 - Long	0.999989	Node-00307 - Long	1.000056	Node-00307 - Long	NA
Node-00307 - Lat	0.999989	Node-00307 - Lat	1.000142	Node-00307 - Lat	NA
Node-00308 - Long	0.999989	Node-00308 - Long	0.999986	Node-00308 - Long	1.000029

Node-00308 - Lat	1.000068	Node-00308 - Lat	0.999986	Node-00308 - Lat	1.000018
Node-00309 - Long	1.00004	Node-00309 - Long	NA	Node-00309 - Long	1.000039
Node-00309 - Lat	1.00004	Node-00309 - Lat	NA	Node-00309 - Lat	1.000017
Node-00310 - Long	1.000037	Node-00310 - Long	1.000054	Node-00310 - Long	1.000039
Node-00310 - Lat	1.000001	Node-00310 - Lat	0.999995	Node-00310 - Lat	1.000039
Node-00311 - Long	NA	Node-00311 - Long	0.999972	Node-00311 - Long	1.00002
Node-00311 - Lat	NA	Node-00311 - Lat	0.999975	Node-00311 - Lat	1.000017
Node-00312 - Long	1.000099	Node-00312 - Long	1.000053	Node-00312 - Long	NA
Node-00312 - Lat	1.000009	Node-00312 - Lat	0.999986	Node-00312 - Lat	NA
Node-00313 - Long	1.000067	Node-00313 - Long	0.999975	Node-00313 - Long	1.000066
Node-00313 - Lat	1.00005	Node-00313 - Lat	1.000014	Node-00313 - Lat	1
Node-00314 - Long	NA	Node-00314 - Long	0.999982	Node-00314 - Long	1.000033
Node-00314 - Lat	NA	Node-00314 - Lat	1.00001	Node-00314 - Lat	1.000044
Node-00315 - Long	1.00002	Node-00315 - Long	1.001031	Node-00315 - Long	NA
Node-00315 - Lat	1.000055	Node-00315 - Lat	1.000777	Node-00315 - Lat	NA
Node-00316 - Long	NA	Node-00316 - Long	NA	Node-00316 - Long	1.000001
Node-00316 - Lat	NA	Node-00316 - Lat	NA	Node-00316 - Lat	1.000099
Node-00317 - Long	NA	Node-00317 - Long	NA	Node-00317 - Long	NA
Node-00317 - Lat	NA	Node-00317 - Lat	NA	Node-00317 - Lat	NA
Node-00318 - Long	NA	Node-00318 - Long	NA	Node-00318 - Long	NA
Node-00318 - Lat	NA	Node-00318 - Lat	NA	Node-00318 - Lat	NA
Node-00319 - Long	NA	Node-00319 - Long	1.000024	Node-00319 - Long	NA
Node-00319 - Lat	NA	Node-00319 - Lat	1.000006	Node-00319 - Lat	NA
Node-00320 - Long	0.999994	Node-00320 - Long	NA	Node-00320 - Long	NA
Node-00320 - Lat	1.000021	Node-00320 - Lat	NA	Node-00320 - Lat	NA
Node-00321 - Long	0.999973	Node-00321 - Long	NA	Node-00321 - Long	0.999965
Node-00321 - Lat	0.999973	Node-00321 - Lat	NA	Node-00321 - Lat	1.000076
Node-00322 - Long	NA	Node-00322 - Long	NA	Node-00322 - Long	1.000004
Node-00322 - Lat	NA	Node-00322 - Lat	NA	Node-00322 - Lat	1.000004
Node-00323 - Long	NA	Node-00323 - Long	1.000065	Node-00323 - Long	NA
Node-00323 - Lat	NA	Node-00323 - Lat	0.999994	Node-00323 - Lat	NA
Node-00324 - Long	NA	Node-00324 - Long	NA	Node-00324 - Long	NA
Node-00324 - Lat	NA	Node-00324 - Lat	NA	Node-00324 - Lat	NA
Node-00325 - Long	0.99997	Node-00325 - Long	NA	Node-00325 - Long	NA
Node-00325 - Lat	0.999977	Node-00325 - Lat	NA	Node-00325 - Lat	NA
Node-00326 - Long	1.000018	Node-00326 - Long	0.999984	Node-00326 - Long	0.999993
Node-00326 - Lat	0.99999	Node-00326 - Lat	1.00007	Node-00326 - Lat	1.000029
Node-00327 - Long	1.000001	Node-00327 - Long	0.999999	Node-00327 - Long	0.999995
Node-00327 - Lat	1.000018	Node-00327 - Lat	1.000059	Node-00327 - Lat	1.000044
Node-00328 - Long	NA	Node-00328 - Long	1.000024	Node-00328 - Long	1.000028
Node-00328 - Lat	NA	Node-00328 - Lat	1.000035	Node-00328 - Lat	1.000022
Node-00329 - Long	NA	Node-00329 - Long	NA	Node-00329 - Long	NA
Node-00329 - Lat	NA	Node-00329 - Lat	NA	Node-00329 - Lat	NA
Node-00330 - Long	NA	Node-00330 - Long	NA	Node-00330 - Long	NA
Node-00330 - Lat	NA	Node-00330 - Lat	NA	Node-00330 - Lat	NA
Node-00331 - Long	NA	Node-00331 - Long	NA	Node-00331 - Long	NA
Node-00331 - Lat	NA	Node-00331 - Lat	NA	Node-00331 - Lat	NA
Node-00332 - Long	NA	Node-00332 - Long	NA	Node-00332 - Long	NA

Node-00332 - Lat	NA	Node-00332 - Lat	NA	Node-00332 - Lat	NA
Node-00333 - Long	0.999975	Node-00333 - Long	NA	Node-00333 - Long	NA
Node-00333 - Lat	1.000103	Node-00333 - Lat	NA	Node-00333 - Lat	NA
Node-00334 - Long	NA	Node-00334 - Long	1.000021	Node-00334 - Long	1.00007
Node-00334 - Lat	NA	Node-00334 - Lat	1.000066	Node-00334 - Lat	0.999989
Node-00335 - Long	1.00005	Node-00335 - Long	NA	Node-00335 - Long	NA
Node-00335 - Lat	0.999995	Node-00335 - Lat	NA	Node-00335 - Lat	NA
Node-00336 - Long	NA	Node-00336 - Long	1.000002	Node-00336 - Long	1.000014
Node-00336 - Lat	NA	Node-00336 - Lat	0.999976	Node-00336 - Lat	0.99998
Node-00337 - Long	NA	Node-00337 - Long	NA	Node-00337 - Long	NA
Node-00337 - Lat	NA	Node-00337 - Lat	NA	Node-00337 - Lat	NA
Node-00338 - Long	1	Node-00338 - Long	NA	Node-00338 - Long	NA
Node-00338 - Lat	1.000062	Node-00338 - Lat	NA	Node-00338 - Lat	NA
Node-00339 - Long	1.000046	Node-00339 - Long	1.00005	Node-00339 - Long	0.999986
Node-00339 - Lat	0.999999	Node-00339 - Lat	0.999998	Node-00339 - Lat	1.000051
Node-00340 - Long	1.000018	Node-00340 - Long	1.000064	Node-00340 - Long	0.999984
Node-00340 - Lat	0.999986	Node-00340 - Lat	1	Node-00340 - Lat	1.000053
Node-00341 - Long	0.999985	Node-00341 - Long	1.000097	Node-00341 - Long	1.000018
Node-00341 - Lat	1.000004	Node-00341 - Lat	0.999975	Node-00341 - Lat	1.000071
Node-00342 - Long	1	Node-00342 - Long	1.000024	Node-00342 - Long	0.999984
Node-00342 - Lat	0.999989	Node-00342 - Lat	1.000007	Node-00342 - Lat	1.000059
Node-00343 - Long	1.000008	Node-00343 - Long	1.000057	Node-00343 - Long	1.000005
Node-00343 - Lat	1.00002	Node-00343 - Lat	1.000022	Node-00343 - Lat	1.00002
Node-00344 - Long	1.000049	Node-00344 - Long	0.999992	Node-00344 - Long	1.00009
Node-00344 - Lat	1.000025	Node-00344 - Lat	1.000015	Node-00344 - Lat	0.999988
Node-00345 - Long	1.000044	Node-00345 - Long	1.000012	Node-00345 - Long	1.000058
Node-00345 - Lat	1.000049	Node-00345 - Lat	1.000024	Node-00345 - Lat	0.999969
Node-00346 - Long	1.00007	Node-00346 - Long	1.000037	Node-00346 - Long	1.000081
Node-00346 - Lat	1.000036	Node-00346 - Lat	1.000001	Node-00346 - Lat	1.00002
Node-00347 - Long	0.999988	Node-00347 - Long	0.999999	Node-00347 - Long	1.000065
Node-00347 - Lat	0.999995	Node-00347 - Lat	1.000023	Node-00347 - Lat	1.000047
Node-00348 - Long	0.999977	Node-00348 - Long	1.000008	Node-00348 - Long	1.000033
Node-00348 - Lat	0.999973	Node-00348 - Lat	1.000045	Node-00348 - Lat	1.000119
Node-00349 - Long	0.999996	Node-00349 - Long	1.000067	Node-00349 - Long	1.000047
Node-00349 - Lat	0.99999	Node-00349 - Lat	1.000074	Node-00349 - Lat	1.000049
Node-00350 - Long	1.000039	Node-00350 - Long	1.000039	Node-00350 - Long	0.999976
Node-00350 - Lat	1.000016	Node-00350 - Lat	1.000101	Node-00350 - Lat	1.000009
Node-00351 - Long	1.000048	Node-00351 - Long	0.999976	Node-00351 - Long	1.000056
Node-00351 - Lat	1.000036	Node-00351 - Lat	1.000105	Node-00351 - Lat	0.999999
Node-00352 - Long	NA	Node-00352 - Long	1.000018	Node-00352 - Long	1.000024
Node-00352 - Lat	NA	Node-00352 - Lat	1.000066	Node-00352 - Lat	1.000013
Node-00353 - Long	1.000071	Node-00353 - Long	NA	Node-00353 - Long	NA
Node-00353 - Lat	0.999997	Node-00353 - Lat	NA	Node-00353 - Lat	NA
Node-00354 - Long	1.000025	Node-00354 - Long	1.000014	Node-00354 - Long	1.000034
Node-00354 - Lat	1.00004	Node-00354 - Lat	0.999992	Node-00354 - Lat	1.000033
Node-00355 - Long	1.000001	Node-00355 - Long	1.000027	Node-00355 - Long	1.000019
Node-00355 - Lat	1.000039	Node-00355 - Lat	1.000015	Node-00355 - Lat	1.000052
Node-00356 - Long	1.000071	Node-00356 - Long	1	Node-00356 - Long	0.999997

Node-00356 - Lat	1.000071	Node-00356 - Lat	1.000055	Node-00356 - Lat	1.000104
Node-00357 - Long	NA	Node-00357 - Long	1.000035	Node-00357 - Long	1.000059
Node-00357 - Lat	NA	Node-00357 - Lat	1.000035	Node-00357 - Lat	1.000059
Node-00358 - Long	0.999988	Node-00358 - Long	NA	Node-00358 - Long	NA
Node-00358 - Lat	0.999988	Node-00358 - Lat	NA	Node-00358 - Lat	NA
Node-00359 - Long	NA	Node-00359 - Long	1.000045	Node-00359 - Long	0.999994
Node-00359 - Lat	NA	Node-00359 - Lat	1.000045	Node-00359 - Lat	0.999994
Node-00360 - Long	0.99997	Node-00360 - Long	NA	Node-00360 - Long	NA
Node-00360 - Lat	1.00003	Node-00360 - Lat	NA	Node-00360 - Lat	NA
Node-00361 - Long	1.000047	Node-00361 - Long	0.999998	Node-00361 - Long	1.000027
Node-00361 - Lat	1	Node-00361 - Lat	1.000017	Node-00361 - Lat	1.000072
Node-00362 - Long	NA	Node-00362 - Long	1.000002	Node-00362 - Long	1.00005
Node-00362 - Lat	NA	Node-00362 - Lat	1.00005	Node-00362 - Lat	1.000008
Node-00363 - Long	1.000091	Node-00363 - Long	NA	Node-00363 - Long	NA
Node-00363 - Lat	1.000091	Node-00363 - Lat	NA	Node-00363 - Lat	NA
Node-00364 - Long	1.00001	Node-00364 - Long	1.00001	Node-00364 - Long	0.999986
Node-00364 - Lat	1.000134	Node-00364 - Lat	1.00001	Node-00364 - Lat	0.999986
Node-00365 - Long	NA	Node-00365 - Long	1.000059	Node-00365 - Long	1.00004
Node-00365 - Lat	NA	Node-00365 - Lat	1.000002	Node-00365 - Lat	1.000004
Node-00366 - Long	1.000059	Node-00366 - Long	NA	Node-00366 - Long	NA
Node-00366 - Lat	1.000007	Node-00366 - Lat	NA	Node-00366 - Lat	NA
Node-00367 - Long	NA	Node-00367 - Long	1.000064	Node-00367 - Long	1.000062
Node-00367 - Lat	NA	Node-00367 - Lat	1.000017	Node-00367 - Lat	0.999998
Node-00368 - Long	1.000331	Node-00368 - Long	NA	Node-00368 - Long	NA
Node-00368 - Lat	1.000237	Node-00368 - Lat	NA	Node-00368 - Lat	NA
Node-00369 - Long	NA	Node-00369 - Long	1.000339	Node-00369 - Long	1.000009
Node-00369 - Lat	NA	Node-00369 - Lat	1.000199	Node-00369 - Lat	0.999995
Node-00370 - Long	1.000058	Node-00370 - Long	NA	Node-00370 - Long	NA
Node-00370 - Lat	1.000093	Node-00370 - Lat	NA	Node-00370 - Lat	NA
Node-00371 - Long	1.000048	Node-00371 - Long	0.999994	Node-00371 - Long	0.999997
Node-00371 - Lat	0.99999	Node-00371 - Lat	1.000081	Node-00371 - Lat	0.999982
Node-00372 - Long	1.000017	Node-00372 - Long	1.000053	Node-00372 - Long	0.999996
Node-00372 - Lat	1.000006	Node-00372 - Lat	1.000028	Node-00372 - Lat	1.000081
Node-00373 - Long	1.000003	Node-00373 - Long	1.000046	Node-00373 - Long	0.999985
Node-00373 - Lat	1.000004	Node-00373 - Lat	1.000093	Node-00373 - Lat	1.000045
Node-00374 - Long	1.000043	Node-00374 - Long	1.000021	Node-00374 - Long	1.000024
Node-00374 - Lat	1.000048	Node-00374 - Lat	1.00002	Node-00374 - Lat	1.000021
Node-00375 - Long	0.999979	Node-00375 - Long	1.000016	Node-00375 - Long	1.000025
Node-00375 - Lat	0.999997	Node-00375 - Lat	1.000021	Node-00375 - Lat	0.999993
Node-00376 - Long	NA	Node-00376 - Long	1.000049	Node-00376 - Long	1.000025
Node-00376 - Lat	NA	Node-00376 - Lat	1.000011	Node-00376 - Lat	0.999992
Node-00377 - Long	1.000012	Node-00377 - Long	NA	Node-00377 - Long	NA
Node-00377 - Lat	1.000029	Node-00377 - Lat	NA	Node-00377 - Lat	NA
Node-00378 - Long	1.00002	Node-00378 - Long	1.000068	Node-00378 - Long	1.000078
Node-00378 - Lat	0.999976	Node-00378 - Lat	1.000036	Node-00378 - Lat	1.000067
Node-00379 - Long	1.000024	Node-00379 - Long	1.000051	Node-00379 - Long	1.000011
Node-00379 - Lat	1.000002	Node-00379 - Lat	1.000003	Node-00379 - Lat	1.000057
Node-00380 - Long	NA	Node-00380 - Long	1.000097	Node-00380 - Long	1.000039

Node-00380 - Lat	NA	Node-00380 - Lat	1.000008	Node-00380 - Lat	0.999987
Node-00381 - Long	1.00003	Node-00381 - Long	NA	Node-00381 - Long	NA
Node-00381 - Lat	0.999987	Node-00381 - Lat	NA	Node-00381 - Lat	NA
Node-00382 - Long	NA	Node-00382 - Long	1.000002	Node-00382 - Long	1.000023
Node-00382 - Lat	NA	Node-00382 - Lat	0.99999	Node-00382 - Lat	0.999986
Node-00383 - Long	1.000057	Node-00383 - Long	NA	Node-00383 - Long	NA
Node-00383 - Lat	1.000038	Node-00383 - Lat	NA	Node-00383 - Lat	NA
Node-00384 - Long	1.000021	Node-00384 - Long	1.000009	Node-00384 - Long	1.000058
Node-00384 - Lat	1.000041	Node-00384 - Lat	1.000072	Node-00384 - Lat	1.000049
Node-00385 - Long	1.002507	Node-00385 - Long	0.999973	Node-00385 - Long	0.99998
Node-00385 - Lat	1.003262	Node-00385 - Lat	1.000061	Node-00385 - Lat	1.000009
Node-00386 - Long	1.000012	Node-00386 - Long	1.003684	Node-00386 - Long	1.000686
Node-00386 - Lat	1.000099	Node-00386 - Lat	1.004972	Node-00386 - Lat	1.00132
Node-00387 - Long	0.999968	Node-00387 - Long	1.000103	Node-00387 - Long	0.999994
Node-00387 - Lat	1.00003	Node-00387 - Lat	1.000162	Node-00387 - Lat	0.999999
Node-00388 - Long	1.000042	Node-00388 - Long	1.000071	Node-00388 - Long	0.999994
Node-00388 - Lat	1.000058	Node-00388 - Lat	0.999962	Node-00388 - Lat	0.999979
Node-00389 - Long	NA	Node-00389 - Long	0.999984	Node-00389 - Long	0.999986
Node-00389 - Lat	NA	Node-00389 - Lat	0.999978	Node-00389 - Lat	0.999978
Node-00390 - Long	1.000024	Node-00390 - Long	NA	Node-00390 - Long	NA
Node-00390 - Lat	1.000021	Node-00390 - Lat	NA	Node-00390 - Lat	NA
Node-00391 - Long	1.000024	Node-00391 - Long	1.000039	Node-00391 - Long	1.000053
Node-00391 - Lat	0.999989	Node-00391 - Lat	1.000003	Node-00391 - Lat	1.000047
Node-00392 - Long	0.999977	Node-00392 - Long	0.999985	Node-00392 - Long	1.000041
Node-00392 - Lat	1.000025	Node-00392 - Lat	1.000021	Node-00392 - Lat	1.000011
Node-00393 - Long	0.999972	Node-00393 - Long	1.000034	Node-00393 - Long	0.999996
Node-00393 - Lat	1.000052	Node-00393 - Lat	0.999968	Node-00393 - Lat	1.000033
Node-00394 - Long	0.999992	Node-00394 - Long	1.000063	Node-00394 - Long	1.000047
Node-00394 - Lat	1.000095	Node-00394 - Lat	0.999999	Node-00394 - Lat	1.000007
Node-00395 - Long	NA	Node-00395 - Long	1.000078	Node-00395 - Long	1.00004
Node-00395 - Lat	NA	Node-00395 - Lat	1.000132	Node-00395 - Lat	0.999986
Node-00396 - Long	0.999992	Node-00396 - Long	NA	Node-00396 - Long	NA
Node-00396 - Lat	0.999993	Node-00396 - Lat	NA	Node-00396 - Lat	NA
Node-00397 - Long	NA	Node-00397 - Long	0.999989	Node-00397 - Long	0.999996
Node-00397 - Lat	NA	Node-00397 - Lat	1.000025	Node-00397 - Lat	1.00012
Node-00398 - Long	NA	Node-00398 - Long	NA	Node-00398 - Long	NA
Node-00398 - Lat	NA	Node-00398 - Lat	NA	Node-00398 - Lat	NA
Node-00399 - Long	NA	Node-00399 - Long	NA	Node-00399 - Long	NA
Node-00399 - Lat	NA	Node-00399 - Lat	NA	Node-00399 - Lat	NA
Node-00400 - Long	NA	Node-00400 - Long	NA	Node-00400 - Long	NA
Node-00400 - Lat	NA	Node-00400 - Lat	NA	Node-00400 - Lat	NA
Node-00401 - Long	1.000009	Node-00401 - Long	NA	Node-00401 - Long	NA
Node-00401 - Lat	0.999964	Node-00401 - Lat	NA	Node-00401 - Lat	NA
Node-00402 - Long	1.000077	Node-00402 - Long	0.999978	Node-00402 - Long	1.000001
Node-00402 - Lat	1.000007	Node-00402 - Lat	1.000009	Node-00402 - Lat	0.999995
Node-00403 - Long	0.99998	Node-00403 - Long	0.999988	Node-00403 - Long	1.000046
Node-00403 - Lat	1.000024	Node-00403 - Lat	1.000007	Node-00403 - Lat	1.00007
Node-00404 - Long	0.999984	Node-00404 - Long	0.999968	Node-00404 - Long	1.000004

Node-00404 - Lat	0.999979	Node-00404 - Lat	1.000003	Node-00404 - Lat	1.000031
Node-00405 - Long	NA	Node-00405 - Long	0.999984	Node-00405 - Long	1.000007
Node-00405 - Lat	NA	Node-00405 - Lat	1.000017	Node-00405 - Lat	0.999991
Node-00406 - Long	NA	Node-00406 - Long	NA	Node-00406 - Long	NA
Node-00406 - Lat	NA	Node-00406 - Lat	NA	Node-00406 - Lat	NA
Node-00407 - Long	NA	Node-00407 - Long	NA	Node-00407 - Long	NA
Node-00407 - Lat	NA	Node-00407 - Lat	NA	Node-00407 - Lat	NA
Node-00408 - Long	0.999969	Node-00408 - Long	NA	Node-00408 - Long	NA
Node-00408 - Lat	1.000016	Node-00408 - Lat	NA	Node-00408 - Lat	NA
Node-00409 - Long	NA	Node-00409 - Long	1.000036	Node-00409 - Long	1.000129
Node-00409 - Lat	NA	Node-00409 - Lat	1.000027	Node-00409 - Lat	1.000022
Node-00410 - Long	NA	Node-00410 - Long	NA	Node-00410 - Long	NA
Node-00410 - Lat	NA	Node-00410 - Lat	NA	Node-00410 - Lat	NA
Node-00411 - Long	NA	Node-00411 - Long	NA	Node-00411 - Long	NA
Node-00411 - Lat	NA	Node-00411 - Lat	NA	Node-00411 - Lat	NA
Node-00412 - Long	1.000038	Node-00412 - Long	NA	Node-00412 - Long	NA
Node-00412 - Lat	0.999965	Node-00412 - Lat	NA	Node-00412 - Lat	NA
Node-00413 - Long	1.000044	Node-00413 - Long	1.000026	Node-00413 - Long	1.000015
Node-00413 - Lat	0.999968	Node-00413 - Lat	0.999984	Node-00413 - Lat	1.000111
Node-00414 - Long	0.999986	Node-00414 - Long	1.000025	Node-00414 - Long	1.000061
Node-00414 - Lat	0.999966	Node-00414 - Lat	0.999993	Node-00414 - Lat	1.000068
Node-00415 - Long	0.999999	Node-00415 - Long	1.000037	Node-00415 - Long	1.000057
Node-00415 - Lat	0.999971	Node-00415 - Lat	1.000007	Node-00415 - Lat	1.000028
Node-00416 - Long	1.000007	Node-00416 - Long	1.000036	Node-00416 - Long	1.000008
Node-00416 - Lat	1.000045	Node-00416 - Lat	1.000009	Node-00416 - Lat	1.000062
Node-00417 - Long	0.99997	Node-00417 - Long	1.000004	Node-00417 - Long	1.000003
Node-00417 - Lat	1.000013	Node-00417 - Lat	1.00005	Node-00417 - Lat	1.000039
Node-00418 - Long	0.99998	Node-00418 - Long	0.999984	Node-00418 - Long	0.999978
Node-00418 - Lat	0.999984	Node-00418 - Lat	1.000022	Node-00418 - Lat	1.00007
Node-00419 - Long	1.000007	Node-00419 - Long	1.000009	Node-00419 - Long	0.999984
Node-00419 - Lat	1	Node-00419 - Lat	0.999984	Node-00419 - Lat	0.999982
Node-00420 - Long	0.999996	Node-00420 - Long	1.000014	Node-00420 - Long	1.000002
Node-00420 - Lat	1.000011	Node-00420 - Lat	1.000008	Node-00420 - Lat	1.000043
Node-00421 - Long	1.000019	Node-00421 - Long	0.99999	Node-00421 - Long	0.999998
Node-00421 - Lat	0.999983	Node-00421 - Lat	1.000069	Node-00421 - Lat	1.000049
Node-00422 - Long	0.999985	Node-00422 - Long	0.999973	Node-00422 - Long	0.999987
Node-00422 - Lat	0.999979	Node-00422 - Lat	1.000067	Node-00422 - Lat	1.000037
Node-00423 - Long	NA	Node-00423 - Long	1.000003	Node-00423 - Long	1.000043
Node-00423 - Lat	NA	Node-00423 - Lat	1.000012	Node-00423 - Lat	1.000002
Node-00424 - Long	1.000045	Node-00424 - Long	NA	Node-00424 - Long	NA
Node-00424 - Lat	1.00001	Node-00424 - Lat	NA	Node-00424 - Lat	NA
Node-00425 - Long	NA	Node-00425 - Long	1.000014	Node-00425 - Long	1.000032
Node-00425 - Lat	NA	Node-00425 - Lat	1.000038	Node-00425 - Lat	1.000003
Node-00426 - Long	NA	Node-00426 - Long	NA	Node-00426 - Long	NA
Node-00426 - Lat	NA	Node-00426 - Lat	NA	Node-00426 - Lat	NA
Node-00427 - Long	1.000028	Node-00427 - Long	NA	Node-00427 - Long	NA
Node-00427 - Lat	1.000009	Node-00427 - Lat	NA	Node-00427 - Lat	NA
Node-00428 - Long	1.000002	Node-00428 - Long	1.000034	Node-00428 - Long	0.99999

Node-00428 - Lat	1.000009	Node-00428 - Lat	1.000041	Node-00428 - Lat	1.000006
Node-00429 - Long	0.999982	Node-00429 - Long	1.00006	Node-00429 - Long	1.00003
Node-00429 - Lat	1.000001	Node-00429 - Lat	1.000091	Node-00429 - Lat	0.99997
Node-00430 - Long	1.000048	Node-00430 - Long	1.000059	Node-00430 - Long	1.000031
Node-00430 - Lat	0.999996	Node-00430 - Lat	1.000007	Node-00430 - Lat	0.999976
Node-00431 - Long	NA	Node-00431 - Long	0.99999	Node-00431 - Long	1.000045
Node-00431 - Lat	NA	Node-00431 - Lat	1.000086	Node-00431 - Lat	0.999981
Node-00432 - Long	NA	Node-00432 - Long	NA	Node-00432 - Long	NA
Node-00432 - Lat	NA	Node-00432 - Lat	NA	Node-00432 - Lat	NA
Node-00433 - Long	NA	Node-00433 - Long	NA	Node-00433 - Long	NA
Node-00433 - Lat	NA	Node-00433 - Lat	NA	Node-00433 - Lat	NA
Node-00434 - Long	0.999995	Node-00434 - Long	NA	Node-00434 - Long	NA
Node-00434 - Lat	1.000011	Node-00434 - Lat	NA	Node-00434 - Lat	NA
Node-00435 - Long	0.999995	Node-00435 - Long	1.000051	Node-00435 - Long	0.999995
Node-00435 - Lat	0.999984	Node-00435 - Lat	1.000078	Node-00435 - Lat	0.999983
Node-00436 - Long	0.999962	Node-00436 - Long	0.999994	Node-00436 - Long	1.000037
Node-00436 - Lat	1.000011	Node-00436 - Lat	1.000164	Node-00436 - Lat	0.999997
Node-00437 - Long	1.000045	Node-00437 - Long	0.999984	Node-00437 - Long	1.000006
Node-00437 - Lat	1.000001	Node-00437 - Lat	1.000069	Node-00437 - Lat	1.000007
Node-00438 - Long	1.000017	Node-00438 - Long	1	Node-00438 - Long	1.000051
Node-00438 - Lat	1.000033	Node-00438 - Lat	1.000204	Node-00438 - Lat	1.000035
Node-00439 - Long	0.999993	Node-00439 - Long	0.999987	Node-00439 - Long	1.000004
Node-00439 - Lat	0.999987	Node-00439 - Lat	1.000028	Node-00439 - Lat	1.000069
Node-00440 - Long	0.999996	Node-00440 - Long	0.999981	Node-00440 - Long	1.00005
Node-00440 - Lat	1.000017	Node-00440 - Lat	1.000029	Node-00440 - Lat	1.000006
Node-00441 - Long	1.000043	Node-00441 - Long	1.000014	Node-00441 - Long	1.000084
Node-00441 - Lat	1.000106	Node-00441 - Lat	0.999979	Node-00441 - Lat	1.00005
Node-00442 - Long	1.000006	Node-00442 - Long	1.000064	Node-00442 - Long	0.999982
Node-00442 - Lat	1.000035	Node-00442 - Lat	1.000024	Node-00442 - Lat	1.000016
Node-00443 - Long	1.000038	Node-00443 - Long	1.000013	Node-00443 - Long	1.000025
Node-00443 - Lat	1.000001	Node-00443 - Lat	0.99999	Node-00443 - Lat	1.000028
Node-00444 - Long	1.000062	Node-00444 - Long	1.000007	Node-00444 - Long	1.000045
Node-00444 - Lat	1.000005	Node-00444 - Lat	0.999983	Node-00444 - Lat	1.000042
Node-00445 - Long	1.000052	Node-00445 - Long	1.000051	Node-00445 - Long	1.000011
Node-00445 - Lat	0.999979	Node-00445 - Lat	1.000002	Node-00445 - Lat	1.000015
Node-00446 - Long	1.000021	Node-00446 - Long	1.000042	Node-00446 - Long	0.999973
Node-00446 - Lat	0.999978	Node-00446 - Lat	1.000012	Node-00446 - Lat	1.000023
Node-00447 - Long	1.000015	Node-00447 - Long	1.00002	Node-00447 - Long	0.999983
Node-00447 - Lat	1.000001	Node-00447 - Lat	1	Node-00447 - Lat	1.000021
Node-00448 - Long	NA	Node-00448 - Long	1.000108	Node-00448 - Long	1.000092
Node-00448 - Lat	NA	Node-00448 - Lat	1.000028	Node-00448 - Lat	1.000052
Node-00449 - Long	NA	Node-00449 - Long	NA	Node-00449 - Long	NA
Node-00449 - Lat	NA	Node-00449 - Lat	NA	Node-00449 - Lat	NA
Node-00450 - Long	1.000061	Node-00450 - Long	NA	Node-00450 - Long	NA
Node-00450 - Lat	0.999996	Node-00450 - Lat	NA	Node-00450 - Lat	NA
Node-00451 - Long	1.000008	Node-00451 - Long	1.000062	Node-00451 - Long	0.999966
Node-00451 - Lat	1.000001	Node-00451 - Lat	0.999982	Node-00451 - Lat	1.000057
Node-00452 - Long	NA	Node-00452 - Long	1.000041	Node-00452 - Long	1.000022

Node-00452 - Lat	NA	Node-00452 - Lat	1.000003	Node-00452 - Lat	1.000031
Node-00453 - Long	1.000012	Node-00453 - Long	NA	Node-00453 - Long	NA
Node-00453 - Lat	1.000016	Node-00453 - Lat	NA	Node-00453 - Lat	NA
Node-00454 - Long	NA	Node-00454 - Long	1.000139	Node-00454 - Long	1.000079
Node-00454 - Lat	NA	Node-00454 - Lat	0.99997	Node-00454 - Lat	1.000031
Node-00455 - Long	0.999985	Node-00455 - Long	NA	Node-00455 - Long	NA
Node-00455 - Lat	0.999967	Node-00455 - Lat	NA	Node-00455 - Lat	NA
Node-00456 - Long	0.999967	Node-00456 - Long	1.000013	Node-00456 - Long	1.000002
Node-00456 - Lat	1.000002	Node-00456 - Lat	1.000043	Node-00456 - Lat	0.999996
Node-00457 - Long	NA	Node-00457 - Long	1.000055	Node-00457 - Long	0.999977
Node-00457 - Lat	NA	Node-00457 - Lat	1.000053	Node-00457 - Lat	0.999987
Node-00458 - Long	1.000105	Node-00458 - Long	NA	Node-00458 - Long	NA
Node-00458 - Lat	1.000016	Node-00458 - Lat	NA	Node-00458 - Lat	NA
Node-00459 - Long	1.000008	Node-00459 - Long	1.000072	Node-00459 - Long	1.000002
Node-00459 - Lat	1.000008	Node-00459 - Lat	0.999988	Node-00459 - Lat	1.000038
Node-00460 - Long	NA	Node-00460 - Long	0.999981	Node-00460 - Long	0.999984
Node-00460 - Lat	NA	Node-00460 - Lat	0.999981	Node-00460 - Lat	0.999984
Node-00461 - Long	1.000002	Node-00461 - Long	NA	Node-00461 - Long	NA
Node-00461 - Lat	1.000127	Node-00461 - Lat	NA	Node-00461 - Lat	NA
Node-00462 - Long	NA	Node-00462 - Long	1.000003	Node-00462 - Long	1.000023
Node-00462 - Lat	NA	Node-00462 - Lat	1.000004	Node-00462 - Lat	0.999993
Node-00463 - Long	0.999975	Node-00463 - Long	NA	Node-00463 - Long	NA
Node-00463 - Lat	0.999988	Node-00463 - Lat	NA	Node-00463 - Lat	NA
Node-00464 - Long	1.000031	Node-00464 - Long	1.0001	Node-00464 - Long	1.000008
Node-00464 - Lat	1.000073	Node-00464 - Lat	1.000186	Node-00464 - Lat	1.000014
Node-00465 - Long	NA	Node-00465 - Long	1.000052	Node-00465 - Long	0.999977
Node-00465 - Lat	NA	Node-00465 - Lat	1.000029	Node-00465 - Lat	0.999989
Node-00466 - Long	0.999998	Node-00466 - Long	NA	Node-00466 - Long	NA
Node-00466 - Lat	1.000005	Node-00466 - Lat	NA	Node-00466 - Lat	NA
Node-00467 - Long	1.00004	Node-00467 - Long	0.999968	Node-00467 - Long	0.999988
Node-00467 - Lat	1.000068	Node-00467 - Lat	0.999991	Node-00467 - Lat	0.999995
Node-00468 - Long	NA	Node-00468 - Long	1.000099	Node-00468 - Long	1.000066
Node-00468 - Lat	NA	Node-00468 - Lat	1.000065	Node-00468 - Lat	1.000099
Node-00469 - Long	1.000042	Node-00469 - Long	NA	Node-00469 - Long	NA
Node-00469 - Lat	1.000042	Node-00469 - Lat	NA	Node-00469 - Lat	NA
Node-00470 - Long	NA	Node-00470 - Long	1.000047	Node-00470 - Long	1.000016
Node-00470 - Lat	NA	Node-00470 - Lat	1.000047	Node-00470 - Lat	1.000016
Node-00471 - Long	NA	Node-00471 - Long	NA	Node-00471 - Long	NA
Node-00471 - Lat	NA	Node-00471 - Lat	NA	Node-00471 - Lat	NA
Node-00472 - Long	1.000038	Node-00472 - Long	NA	Node-00472 - Long	NA
Node-00472 - Lat	1.000038	Node-00472 - Lat	NA	Node-00472 - Lat	NA
Node-00473 - Long	NA	Node-00473 - Long	1.000119	Node-00473 - Long	0.999969
Node-00473 - Lat	NA	Node-00473 - Lat	1.000119	Node-00473 - Lat	0.999969
Node-00474 - Long	0.999977	Node-00474 - Long	NA	Node-00474 - Long	NA
Node-00474 - Lat	1.000039	Node-00474 - Lat	NA	Node-00474 - Lat	NA
Node-00475 - Long	NA	Node-00475 - Long	1.000067	Node-00475 - Long	1.000006
Node-00475 - Lat	NA	Node-00475 - Lat	1.00007	Node-00475 - Lat	0.999991
Node-00476 - Long	NA	Node-00476 - Long	NA	Node-00476 - Long	NA

Node-00476 - Lat	NA	Node-00476 - Lat	NA	Node-00476 - Lat	NA
Node-00477 - Long	NA	Node-00477 - Long	NA	Node-00477 - Long	NA
Node-00477 - Lat	NA	Node-00477 - Lat	NA	Node-00477 - Lat	NA
Node-00478 - Long	NA	Node-00478 - Long	NA	Node-00478 - Long	NA
Node-00478 - Lat	NA	Node-00478 - Lat	NA	Node-00478 - Lat	NA
Node-00479 - Long	0.999986	Node-00479 - Long	NA	Node-00479 - Long	NA
Node-00479 - Lat	0.999988	Node-00479 - Lat	NA	Node-00479 - Lat	NA
Node-00480 - Long	NA	Node-00480 - Long	1.000043	Node-00480 - Long	1.000002
Node-00480 - Lat	NA	Node-00480 - Lat	1.000069	Node-00480 - Lat	1.000096
Node-00481 - Long	0.999958	Node-00481 - Long	NA	Node-00481 - Long	NA
Node-00481 - Lat	0.999958	Node-00481 - Lat	NA	Node-00481 - Lat	NA
Node-00482 - Long	1.000057	Node-00482 - Long	1.000009	Node-00482 - Long	0.99999
Node-00482 - Lat	1.000042	Node-00482 - Lat	1.000009	Node-00482 - Lat	0.99999
Node-00483 - Long	0.999997	Node-00483 - Long	1.000011	Node-00483 - Long	1.000112
Node-00483 - Lat	0.999977	Node-00483 - Lat	1.000085	Node-00483 - Lat	1.000039
Node-00484 - Long	NA	Node-00484 - Long	1.000011	Node-00484 - Long	1.000204
Node-00484 - Lat	NA	Node-00484 - Lat	1.000039	Node-00484 - Lat	1.000109
Node-00485 - Long	NA	Node-00485 - Long	NA	Node-00485 - Long	NA
Node-00485 - Lat	NA	Node-00485 - Lat	NA	Node-00485 - Lat	NA
Node-00486 - Long	1.000103	Node-00486 - Long	NA	Node-00486 - Long	NA
Node-00486 - Lat	1.000012	Node-00486 - Lat	NA	Node-00486 - Lat	NA
Node-00487 - Long	NA	Node-00487 - Long	1.000116	Node-00487 - Long	1.000086
Node-00487 - Lat	NA	Node-00487 - Lat	1.000003	Node-00487 - Lat	0.999974
Node-00488 - Long	NA	Node-00488 - Long	NA	Node-00488 - Long	NA
Node-00488 - Lat	NA	Node-00488 - Lat	NA	Node-00488 - Lat	NA
Node-00489 - Long	1.000067	Node-00489 - Long	NA	Node-00489 - Long	NA
Node-00489 - Lat	1.00004	Node-00489 - Lat	NA	Node-00489 - Lat	NA
Node-00490 - Long	1.000091	Node-00490 - Long	0.999999	Node-00490 - Long	1.000009
Node-00490 - Lat	1.000078	Node-00490 - Lat	1.000035	Node-00490 - Lat	1.000004
Node-00491 - Long	0.999973	Node-00491 - Long	1.000033	Node-00491 - Long	0.999967
Node-00491 - Lat	1.00006	Node-00491 - Lat	0.999987	Node-00491 - Lat	1.000108
Node-00492 - Long	NA	Node-00492 - Long	1.00001	Node-00492 - Long	1.000026
Node-00492 - Lat	NA	Node-00492 - Lat	0.99999	Node-00492 - Lat	1.000004
Node-00493 - Long	1.000024	Node-00493 - Long	NA	Node-00493 - Long	NA
Node-00493 - Lat	1.00005	Node-00493 - Lat	NA	Node-00493 - Lat	NA
Node-00494 - Long	1.000096	Node-00494 - Long	1.000045	Node-00494 - Long	0.999966
Node-00494 - Lat	1.000016	Node-00494 - Lat	1.000029	Node-00494 - Lat	1.000102
Node-00495 - Long	NA	Node-00495 - Long	0.99999	Node-00495 - Long	0.999981
Node-00495 - Lat	NA	Node-00495 - Lat	0.999975	Node-00495 - Lat	1.000045
Node-00496 - Long	1.000029	Node-00496 - Long	NA	Node-00496 - Long	NA
Node-00496 - Lat	1.000001	Node-00496 - Lat	NA	Node-00496 - Lat	NA
Node-00497 - Long	NA	Node-00497 - Long	1.000041	Node-00497 - Long	1.00002
Node-00497 - Lat	NA	Node-00497 - Lat	1.000037	Node-00497 - Lat	1.000022
Node-00498 - Long	NA	Node-00498 - Long	NA	Node-00498 - Long	NA
Node-00498 - Lat	NA	Node-00498 - Lat	NA	Node-00498 - Lat	NA
Node-00499 - Long	NA	Node-00499 - Long	NA	Node-00499 - Long	NA
Node-00499 - Lat	NA	Node-00499 - Lat	NA	Node-00499 - Lat	NA
Node-00500 - Long	NA	Node-00500 - Long	NA	Node-00500 - Long	NA

Node-00500 - Lat	NA	Node-00500 - Lat	NA	Node-00500 - Lat	NA
Node-00501 - Long	NA	Node-00501 - Long	NA	Node-00501 - Long	NA
Node-00501 - Lat	NA	Node-00501 - Lat	NA	Node-00501 - Lat	NA
Node-00502 - Long	1.000071	Node-00502 - Long	NA	Node-00502 - Long	NA
Node-00502 - Lat	1.000071	Node-00502 - Lat	NA	Node-00502 - Lat	NA
Node-00503 - Long	NA	Node-00503 - Long	1.000033	Node-00503 - Long	1.000003
Node-00503 - Lat	NA	Node-00503 - Lat	1.000033	Node-00503 - Lat	1.000003
Node-00504 - Long	NA	Node-00504 - Long	NA	Node-00504 - Long	NA
Node-00504 - Lat	NA	Node-00504 - Lat	NA	Node-00504 - Lat	NA
Node-00505 - Long	1.000026	Node-00505 - Long	NA	Node-00505 - Long	NA
Node-00505 - Lat	1.000022	Node-00505 - Lat	NA	Node-00505 - Lat	NA
Node-00506 - Long	NA	Node-00506 - Long	1.000039	Node-00506 - Long	0.999993
Node-00506 - Lat	NA	Node-00506 - Lat	1.000075	Node-00506 - Lat	0.999976
Node-00507 - Long	NA	Node-00507 - Long	NA	Node-00507 - Long	NA
Node-00507 - Lat	NA	Node-00507 - Lat	NA	Node-00507 - Lat	NA
Node-00508 - Long	1.000108	Node-00508 - Long	NA	Node-00508 - Long	NA
Node-00508 - Lat	1.000038	Node-00508 - Lat	NA	Node-00508 - Lat	NA
Node-00509 - Long	NA	Node-00509 - Long	0.999982	Node-00509 - Long	1.000059
Node-00509 - Lat	NA	Node-00509 - Lat	1.000009	Node-00509 - Lat	1.000033
Node-00510 - Long	NA	Node-00510 - Long	NA	Node-00510 - Long	NA
Node-00510 - Lat	NA	Node-00510 - Lat	NA	Node-00510 - Lat	NA
Node-00511 - Long	0.999997	Node-00511 - Long	NA	Node-00511 - Long	NA
Node-00511 - Lat	0.999976	Node-00511 - Lat	NA	Node-00511 - Lat	NA
Node-00512 - Long	1.000017	Node-00512 - Long	1.000037	Node-00512 - Long	1.000018
Node-00512 - Lat	0.999992	Node-00512 - Lat	0.999967	Node-00512 - Lat	1.000027
Node-00513 - Long	1.000094	Node-00513 - Long	1.000025	Node-00513 - Long	1.000054
Node-00513 - Lat	0.999996	Node-00513 - Lat	0.999999	Node-00513 - Lat	1.000006
Node-00514 - Long	0.999983	Node-00514 - Long	1.000078	Node-00514 - Long	1.000011
Node-00514 - Lat	1.000004	Node-00514 - Lat	1	Node-00514 - Lat	1.000002
Node-00515 - Long	NA	Node-00515 - Long	0.999997	Node-00515 - Long	0.999976
Node-00515 - Lat	NA	Node-00515 - Lat	0.999989	Node-00515 - Lat	0.999984
Node-00516 - Long	NA	Node-00516 - Long	NA	Node-00516 - Long	NA
Node-00516 - Lat	NA	Node-00516 - Lat	NA	Node-00516 - Lat	NA
Node-00517 - Long	NA	Node-00517 - Long	NA	Node-00517 - Long	NA
Node-00517 - Lat	NA	Node-00517 - Lat	NA	Node-00517 - Lat	NA
Node-00518 - Long	NA	Node-00518 - Long	NA	Node-00518 - Long	NA
Node-00518 - Lat	NA	Node-00518 - Lat	NA	Node-00518 - Lat	NA
Node-00519 - Long	NA	Node-00519 - Long	NA	Node-00519 - Long	NA
Node-00519 - Lat	NA	Node-00519 - Lat	NA	Node-00519 - Lat	NA
Node-00520 - Long	NA	Node-00520 - Long	NA	Node-00520 - Long	NA
Node-00520 - Lat	NA	Node-00520 - Lat	NA	Node-00520 - Lat	NA
Node-00521 - Long	1.000038	Node-00521 - Long	NA	Node-00521 - Long	NA
Node-00521 - Lat	0.99999	Node-00521 - Lat	NA	Node-00521 - Lat	NA
Node-00522 - Long	1.000021	Node-00522 - Long	1.000003	Node-00522 - Long	1.000024
Node-00522 - Lat	0.999968	Node-00522 - Lat	0.999983	Node-00522 - Lat	0.99999
Node-00523 - Long	1.000098	Node-00523 - Long	1.000031	Node-00523 - Long	1.000047
Node-00523 - Lat	1.000075	Node-00523 - Lat	0.999963	Node-00523 - Lat	1.000008
Node-00524 - Long	1.000045	Node-00524 - Long	1.000089	Node-00524 - Long	1.000073

Node-00524 - Lat	1.000035	Node-00524 - Lat	0.999994	Node-00524 - Lat	0.999988
Node-00525 - Long	NA	Node-00525 - Long	0.999997	Node-00525 - Long	1.000068
Node-00525 - Lat	NA	Node-00525 - Lat	0.999991	Node-00525 - Lat	1.000026
Node-00526 - Long	1.00004	Node-00526 - Long	NA	Node-00526 - Long	NA
Node-00526 - Lat	1.000032	Node-00526 - Lat	NA	Node-00526 - Lat	NA
Node-00527 - Long	NA	Node-00527 - Long	1.000007	Node-00527 - Long	0.999984
Node-00527 - Lat	NA	Node-00527 - Lat	1.000037	Node-00527 - Lat	1
Node-00528 - Long	NA	Node-00528 - Long	NA	Node-00528 - Long	NA
Node-00528 - Lat	NA	Node-00528 - Lat	NA	Node-00528 - Lat	NA
Node-00529 - Long	0.999969	Node-00529 - Long	NA	Node-00529 - Long	NA
Node-00529 - Lat	0.99997	Node-00529 - Lat	NA	Node-00529 - Lat	NA
Node-00530 - Long	NA	Node-00530 - Long	0.999961	Node-00530 - Long	1.000009
Node-00530 - Lat	NA	Node-00530 - Lat	0.999972	Node-00530 - Lat	0.999983
Node-00531 - Long	NA	Node-00531 - Long	NA	Node-00531 - Long	NA
Node-00531 - Lat	NA	Node-00531 - Lat	NA	Node-00531 - Lat	NA
Node-00532 - Long	1.000055	Node-00532 - Long	NA	Node-00532 - Long	NA
Node-00532 - Lat	1	Node-00532 - Lat	NA	Node-00532 - Lat	NA
Node-00533 - Long	NA	Node-00533 - Long	0.999985	Node-00533 - Long	1.000017
Node-00533 - Lat	NA	Node-00533 - Lat	1.000004	Node-00533 - Lat	1
Node-00534 - Long	1.000098	Node-00534 - Long	NA	Node-00534 - Long	NA
Node-00534 - Lat	1.000027	Node-00534 - Lat	NA	Node-00534 - Lat	NA
Node-00535 - Long	NA	Node-00535 - Long	1.000002	Node-00535 - Long	1.000129
Node-00535 - Lat	NA	Node-00535 - Lat	0.999994	Node-00535 - Lat	1.000034
Node-00536 - Long	NA	Node-00536 - Long	NA	Node-00536 - Long	NA
Node-00536 - Lat	NA	Node-00536 - Lat	NA	Node-00536 - Lat	NA
Node-00537 - Long	NA	Node-00537 - Long	NA	Node-00537 - Long	NA
Node-00537 - Lat	NA	Node-00537 - Lat	NA	Node-00537 - Lat	NA
Node-00538 - Long	1.000067	Node-00538 - Long	NA	Node-00538 - Long	NA
Node-00538 - Lat	0.999973	Node-00538 - Lat	NA	Node-00538 - Lat	NA
Node-00539 - Long	NA	Node-00539 - Long	1.000034	Node-00539 - Long	1.000012
Node-00539 - Lat	NA	Node-00539 - Lat	1.00001	Node-00539 - Lat	0.999993
Node-00540 - Long	NA	Node-00540 - Long	NA	Node-00540 - Long	NA
Node-00540 - Lat	NA	Node-00540 - Lat	NA	Node-00540 - Lat	NA
Node-00541 - Long	1.000055	Node-00541 - Long	NA	Node-00541 - Long	NA
Node-00541 - Lat	1.000041	Node-00541 - Lat	NA	Node-00541 - Lat	NA
Node-00542 - Long	1.000007	Node-00542 - Long	1.000043	Node-00542 - Long	0.999983
Node-00542 - Lat	1.000016	Node-00542 - Lat	1.000054	Node-00542 - Lat	1.000006
Node-00543 - Long	1.000059	Node-00543 - Long	0.999994	Node-00543 - Long	0.999986
Node-00543 - Lat	0.999983	Node-00543 - Lat	1.000001	Node-00543 - Lat	1.000012
Node-00544 - Long	1.000036	Node-00544 - Long	1.000001	Node-00544 - Long	1.00001
Node-00544 - Lat	1.000014	Node-00544 - Lat	0.999995	Node-00544 - Lat	0.999996
Node-00545 - Long	1.000064	Node-00545 - Long	1.000076	Node-00545 - Long	1.000004
Node-00545 - Lat	1.000007	Node-00545 - Lat	1.00002	Node-00545 - Lat	1.000062
Node-00546 - Long	1.000099	Node-00546 - Long	1.000061	Node-00546 - Long	1.000002
Node-00546 - Lat	1.000006	Node-00546 - Lat	1.000039	Node-00546 - Lat	1.000039
Node-00547 - Long	1.00004	Node-00547 - Long	1.000041	Node-00547 - Long	0.999978
Node-00547 - Lat	0.999971	Node-00547 - Lat	1.000037	Node-00547 - Lat	1.000054
Node-00548 - Long	1.000017	Node-00548 - Long	1.000043	Node-00548 - Long	1.000052

Node-00548 - Lat	1.000023	Node-00548 - Lat	1.000051	Node-00548 - Lat	1.000068
Node-00549 - Long	1.000006	Node-00549 - Long	1.000031	Node-00549 - Long	1.000066
Node-00549 - Lat	0.99999	Node-00549 - Lat	0.999975	Node-00549 - Lat	0.999998
Node-00550 - Long	0.999998	Node-00550 - Long	1.000038	Node-00550 - Long	1.000037
Node-00550 - Lat	0.999969	Node-00550 - Lat	1.000008	Node-00550 - Lat	1.000004
Node-00551 - Long	1.000017	Node-00551 - Long	0.999992	Node-00551 - Long	1.000079
Node-00551 - Lat	1.000014	Node-00551 - Lat	1.000015	Node-00551 - Lat	1.000004
Node-00552 - Long	1.00004	Node-00552 - Long	1.000149	Node-00552 - Long	1.000027
Node-00552 - Lat	1.000016	Node-00552 - Lat	1.000056	Node-00552 - Lat	1.000026
Node-00553 - Long	1.000009	Node-00553 - Long	0.999962	Node-00553 - Long	1.000083
Node-00553 - Lat	1.000002	Node-00553 - Lat	1	Node-00553 - Lat	1.000017
Node-00554 - Long	1.000045	Node-00554 - Long	1.000054	Node-00554 - Long	1.000034
Node-00554 - Lat	1.000048	Node-00554 - Lat	1.000053	Node-00554 - Lat	1.000042
Node-00555 - Long	1.000031	Node-00555 - Long	1.000111	Node-00555 - Long	1.000047
Node-00555 - Lat	1.000006	Node-00555 - Lat	1.000008	Node-00555 - Lat	1.000017
Node-00556 - Long	1.000006	Node-00556 - Long	1.000065	Node-00556 - Long	1.000011
Node-00556 - Lat	0.999982	Node-00556 - Lat	0.999985	Node-00556 - Lat	1.000022
Node-00557 - Long	NA	Node-00557 - Long	1.000013	Node-00557 - Long	1.000012
Node-00557 - Lat	NA	Node-00557 - Lat	1	Node-00557 - Lat	1.000004
Node-00558 - Long	0.999969	Node-00558 - Long	NA	Node-00558 - Long	NA
Node-00558 - Lat	0.999969	Node-00558 - Lat	NA	Node-00558 - Lat	NA
Node-00559 - Long	NA	Node-00559 - Long	0.999984	Node-00559 - Long	1.000007
Node-00559 - Lat	NA	Node-00559 - Lat	0.999984	Node-00559 - Lat	1.000007
Node-00560 - Long	0.999972	Node-00560 - Long	NA	Node-00560 - Long	NA
Node-00560 - Lat	1.000037	Node-00560 - Lat	NA	Node-00560 - Lat	NA
Node-00561 - Long	1.000015	Node-00561 - Long	0.999995	Node-00561 - Long	0.999981
Node-00561 - Lat	1.000007	Node-00561 - Lat	0.999999	Node-00561 - Lat	1.000037
Node-00562 - Long	0.999983	Node-00562 - Long	1.000046	Node-00562 - Long	0.999973
Node-00562 - Lat	0.999978	Node-00562 - Lat	1.000129	Node-00562 - Lat	1.000022
Node-00563 - Long	1.000034	Node-00563 - Long	1.000059	Node-00563 - Long	1.000097
Node-00563 - Lat	1.000036	Node-00563 - Lat	1.000032	Node-00563 - Lat	1.000004
Node-00564 - Long	1.000055	Node-00564 - Long	1.000075	Node-00564 - Long	1.000046
Node-00564 - Lat	1.000038	Node-00564 - Lat	1.000058	Node-00564 - Lat	1.000047
Node-00565 - Long	1.000002	Node-00565 - Long	1.000174	Node-00565 - Long	0.999994
Node-00565 - Lat	0.999987	Node-00565 - Lat	1.00008	Node-00565 - Lat	1.000018
Node-00566 - Long	NA	Node-00566 - Long	1.000246	Node-00566 - Long	0.999981
Node-00566 - Lat	NA	Node-00566 - Lat	1.000079	Node-00566 - Lat	1.000002
Node-00567 - Long	1.000002	Node-00567 - Long	NA	Node-00567 - Long	NA
Node-00567 - Lat	1.000029	Node-00567 - Lat	NA	Node-00567 - Lat	NA
Node-00568 - Long	NA	Node-00568 - Long	1.000006	Node-00568 - Long	1.000042
Node-00568 - Lat	NA	Node-00568 - Lat	1.00002	Node-00568 - Lat	1.000091
Node-00569 - Long	1.000059	Node-00569 - Long	NA	Node-00569 - Long	NA
Node-00569 - Lat	1.000008	Node-00569 - Lat	NA	Node-00569 - Lat	NA
Node-00570 - Long	NA	Node-00570 - Long	0.999992	Node-00570 - Long	0.999991
Node-00570 - Lat	NA	Node-00570 - Lat	1.000034	Node-00570 - Lat	1.000002
Node-00571 - Long	NA	Node-00571 - Long	NA	Node-00571 - Long	NA
Node-00571 - Lat	NA	Node-00571 - Lat	NA	Node-00571 - Lat	NA
Node-00572 - Long	1.00009	Node-00572 - Long	NA	Node-00572 - Long	NA

Node-00572 - Lat	1.00009	Node-00572 - Lat	NA	Node-00572 - Lat	NA
Node-00573 - Long	1.000022	Node-00573 - Long	1.000067	Node-00573 - Long	1.000052
Node-00573 - Lat	1.000012	Node-00573 - Lat	1.000067	Node-00573 - Lat	1.000052
Node-00574 - Long	NA	Node-00574 - Long	1.000006	Node-00574 - Long	0.999984
Node-00574 - Lat	NA	Node-00574 - Lat	1.000032	Node-00574 - Lat	1.000033
Node-00575 - Long	1.000056	Node-00575 - Long	NA	Node-00575 - Long	NA
Node-00575 - Lat	0.999963	Node-00575 - Lat	NA	Node-00575 - Lat	NA
Node-00576 - Long	NA	Node-00576 - Long	NA	Node-00576 - Long	0.999993
Node-00576 - Lat	NA	Node-00576 - Lat	NA	Node-00576 - Lat	1.000019
Node-00577 - Long	NA	Node-00577 - Long	NA	Node-00577 - Long	NA
Node-00577 - Lat	NA	Node-00577 - Lat	NA	Node-00577 - Lat	NA
Node-00578 - Long	1.000046	Node-00578 - Long	1.000016	Node-00578 - Long	NA
Node-00578 - Lat	1.000046	Node-00578 - Lat	1.000016	Node-00578 - Lat	NA
Node-00579 - Long	NA	Node-00579 - Long	NA	Node-00579 - Long	1.000007
Node-00579 - Lat	NA	Node-00579 - Lat	NA	Node-00579 - Lat	1.000007
Node-00580 - Long	0.999986	Node-00580 - Long	0.999998	Node-00580 - Long	NA
Node-00580 - Lat	0.999998	Node-00580 - Lat	1.000101	Node-00580 - Lat	NA
Node-00581 - Long	0.999994	Node-00581 - Long	0.999973	Node-00581 - Long	1.000001
Node-00581 - Lat	0.999983	Node-00581 - Lat	1.0001	Node-00581 - Lat	1.00008
Node-00582 - Long	NA	Node-00582 - Long	NA	Node-00582 - Long	1.000042
Node-00582 - Lat	NA	Node-00582 - Lat	NA	Node-00582 - Lat	1.00007
Node-00583 - Long	1.00001	Node-00583 - Long	1.000057	Node-00583 - Long	NA
Node-00583 - Lat	0.999979	Node-00583 - Lat	0.999992	Node-00583 - Lat	NA
Node-00584 - Long	0.99999	Node-00584 - Long	1.00007	Node-00584 - Long	0.999993
Node-00584 - Lat	0.999983	Node-00584 - Lat	0.999991	Node-00584 - Lat	1.000021
Node-00585 - Long	1.000049	Node-00585 - Long	1.000036	Node-00585 - Long	0.999978
Node-00585 - Lat	0.999986	Node-00585 - Lat	0.999975	Node-00585 - Lat	1.000098
Node-00586 - Long	1.000005	Node-00586 - Long	0.999993	Node-00586 - Long	1.000006
Node-00586 - Lat	0.999996	Node-00586 - Lat	0.999974	Node-00586 - Lat	1.000013
Node-00587 - Long	NA	Node-00587 - Long	NA	Node-00587 - Long	0.999976
Node-00587 - Lat	NA	Node-00587 - Lat	NA	Node-00587 - Lat	1.00005
Node-00588 - Long	NA	Node-00588 - Long	NA	Node-00588 - Long	NA
Node-00588 - Lat	NA	Node-00588 - Lat	NA	Node-00588 - Lat	NA
Node-00589 - Long	NA	Node-00589 - Long	NA	Node-00589 - Long	NA
Node-00589 - Lat	NA	Node-00589 - Lat	NA	Node-00589 - Lat	NA
Node-00590 - Long	1.0002	Node-00590 - Long	1.000048	Node-00590 - Long	NA
Node-00590 - Lat	1.00002	Node-00590 - Lat	1.000057	Node-00590 - Lat	NA
Node-00591 - Long	NA	Node-00591 - Long	NA	Node-00591 - Long	1.000008
Node-00591 - Lat	NA	Node-00591 - Lat	NA	Node-00591 - Lat	1.000029
Node-00592 - Long	0.999971	Node-00592 - Long	1.000005	Node-00592 - Long	NA
Node-00592 - Lat	1.000004	Node-00592 - Lat	1.000007	Node-00592 - Lat	NA
Node-00593 - Long	1.000021	Node-00593 - Long	0.999952	Node-00593 - Long	1.000004
Node-00593 - Lat	1.000072	Node-00593 - Lat	1.000055	Node-00593 - Lat	0.999982
Node-00594 - Long	1.000079	Node-00594 - Long	0.999992	Node-00594 - Long	1.000047
Node-00594 - Lat	1.000003	Node-00594 - Lat	1.000042	Node-00594 - Lat	1.000062
Node-00595 - Long	NA	Node-00595 - Long	NA	Node-00595 - Long	1.000017
Node-00595 - Lat	NA	Node-00595 - Lat	NA	Node-00595 - Lat	1.000036
Node-00596 - Long	0.999983	Node-00596 - Long	1.000028	Node-00596 - Long	NA

Node-00596 - Lat	1.000013	Node-00596 - Lat	0.999996	Node-00596 - Lat	NA
Node-00597 - Long	1.000028	Node-00597 - Long	0.999969	Node-00597 - Long	0.999994
Node-00597 - Lat	0.999972	Node-00597 - Lat	0.999973	Node-00597 - Lat	1.000072
Node-00598 - Long	1.000015	Node-00598 - Long	1.000015	Node-00598 - Long	1.000026
Node-00598 - Lat	0.999986	Node-00598 - Lat	0.999999	Node-00598 - Lat	1.000016
Node-00599 - Long	1.00005	Node-00599 - Long	0.999994	Node-00599 - Long	1.000051
Node-00599 - Lat	0.999992	Node-00599 - Lat	0.999999	Node-00599 - Lat	1.00009
Node-00600 - Long	NA	Node-00600 - Long	NA	Node-00600 - Long	1.000047
Node-00600 - Lat	NA	Node-00600 - Lat	NA	Node-00600 - Lat	0.999993
Node-00601 - Long	NA	Node-00601 - Long	NA	Node-00601 - Long	NA
Node-00601 - Lat	NA	Node-00601 - Lat	NA	Node-00601 - Lat	NA
Node-00602 - Long	NA	Node-00602 - Long	NA	Node-00602 - Long	NA
Node-00602 - Lat	NA	Node-00602 - Lat	NA	Node-00602 - Lat	NA
Node-00603 - Long	NA	Node-00603 - Long	NA	Node-00603 - Long	NA
Node-00603 - Lat	NA	Node-00603 - Lat	NA	Node-00603 - Lat	NA
Node-00604 - Long	NA	Node-00604 - Long	NA	Node-00604 - Long	NA
Node-00604 - Lat	NA	Node-00604 - Lat	NA	Node-00604 - Lat	NA
Node-00605 - Long	1	Node-00605 - Long	1.000134	Node-00605 - Long	NA
Node-00605 - Lat	1.000073	Node-00605 - Lat	1.000035	Node-00605 - Lat	NA
Node-00606 - Long	1.000019	Node-00606 - Long	1.000054	Node-00606 - Long	0.999998
Node-00606 - Lat	1.00004	Node-00606 - Lat	1.000163	Node-00606 - Lat	0.999959
Node-00607 - Long	1.000028	Node-00607 - Long	1.000042	Node-00607 - Long	1.000083
Node-00607 - Lat	1.000039	Node-00607 - Lat	1.000125	Node-00607 - Lat	1.000022
Node-00608 - Long	NA	Node-00608 - Long	NA	Node-00608 - Long	1.000061
Node-00608 - Lat	NA	Node-00608 - Lat	NA	Node-00608 - Lat	0.999992
Node-00609 - Long	1.000028	Node-00609 - Long	1.000062	Node-00609 - Long	NA
Node-00609 - Lat	1.000081	Node-00609 - Lat	1.000074	Node-00609 - Lat	NA
Node-00610 - Long	NA	Node-00610 - Long	NA	Node-00610 - Long	1.000116
Node-00610 - Lat	NA	Node-00610 - Lat	NA	Node-00610 - Lat	1.000004
Node-00611 - Long	NA	Node-00611 - Long	NA	Node-00611 - Long	NA
Node-00611 - Lat	NA	Node-00611 - Lat	NA	Node-00611 - Lat	NA
Node-00612 - Long	NA	Node-00612 - Long	NA	Node-00612 - Long	NA
Node-00612 - Lat	NA	Node-00612 - Lat	NA	Node-00612 - Lat	NA
Node-00613 - Long	1.000034	Node-00613 - Long	1	Node-00613 - Long	NA
Node-00613 - Lat	1.000004	Node-00613 - Lat	0.999989	Node-00613 - Lat	NA
Node-00614 - Long	NA	Node-00614 - Long	NA	Node-00614 - Long	0.999991
Node-00614 - Lat	NA	Node-00614 - Lat	NA	Node-00614 - Lat	1
Node-00615 - Long	NA	Node-00615 - Long	NA	Node-00615 - Long	NA
Node-00615 - Lat	NA	Node-00615 - Lat	NA	Node-00615 - Lat	NA
Node-00616 - Long	0.999997	Node-00616 - Long	1.000005	Node-00616 - Long	NA
Node-00616 - Lat	1.000069	Node-00616 - Lat	1.000003	Node-00616 - Lat	NA
Node-00617 - Long	1.000032	Node-00617 - Long	1.000004	Node-00617 - Long	1.000003
Node-00617 - Lat	1.000031	Node-00617 - Lat	1.000011	Node-00617 - Lat	1.000062
Node-00618 - Long	1.000104	Node-00618 - Long	1	Node-00618 - Long	1.000063
Node-00618 - Lat	1.000239	Node-00618 - Lat	1.000114	Node-00618 - Lat	0.999988
Node-00619 - Long	1.00008	Node-00619 - Long	1.000024	Node-00619 - Long	1.000008
Node-00619 - Lat	1.00007	Node-00619 - Lat	1.000048	Node-00619 - Lat	1.000188
Node-00620 - Long	NA	Node-00620 - Long	NA	Node-00620 - Long	0.999996

Node-00620 - Lat	NA	Node-00620 - Lat	NA	Node-00620 - Lat	1.000026
Node-00621 - Long	NA	Node-00621 - Long	NA	Node-00621 - Long	NA
Node-00621 - Lat	NA	Node-00621 - Lat	NA	Node-00621 - Lat	NA
Node-00622 - Long	1.000025	Node-00622 - Long	1.000053	Node-00622 - Long	NA
Node-00622 - Lat	1.00005	Node-00622 - Lat	1.00005	Node-00622 - Lat	NA
Node-00623 - Long	1.00003	Node-00623 - Long	0.999965	Node-00623 - Long	0.999998
Node-00623 - Lat	1	Node-00623 - Lat	1.000008	Node-00623 - Lat	1.000066
Node-00624 - Long	1.004118	Node-00624 - Long	1.003755	Node-00624 - Long	0.999969
Node-00624 - Lat	1.003988	Node-00624 - Lat	1.003759	Node-00624 - Lat	1.000039
Node-00625 - Long	1.000044	Node-00625 - Long	1.000035	Node-00625 - Long	1.002908
Node-00625 - Lat	1.000017	Node-00625 - Lat	1.000036	Node-00625 - Lat	1.002972
Node-00626 - Long	NA	Node-00626 - Long	NA	Node-00626 - Long	0.999991
Node-00626 - Lat	NA	Node-00626 - Lat	NA	Node-00626 - Lat	1.000075
Node-00627 - Long	NA	Node-00627 - Long	NA	Node-00627 - Long	NA
Node-00627 - Lat	NA	Node-00627 - Lat	NA	Node-00627 - Lat	NA
Node-00628 - Long	NA	Node-00628 - Long	NA	Node-00628 - Long	NA
Node-00628 - Lat	NA	Node-00628 - Lat	NA	Node-00628 - Lat	NA
Node-00629 - Long	NA	Node-00629 - Long	NA	Node-00629 - Long	NA
Node-00629 - Lat	NA	Node-00629 - Lat	NA	Node-00629 - Lat	NA
Node-00630 - Long	0.999973	Node-00630 - Long	0.999977	Node-00630 - Long	NA
Node-00630 - Lat	1.000135	Node-00630 - Lat	1.000023	Node-00630 - Lat	NA
Node-00631 - Long	NA	Node-00631 - Long	NA	Node-00631 - Long	1.000013
Node-00631 - Lat	NA	Node-00631 - Lat	NA	Node-00631 - Lat	1.000006
Node-00632 - Long	NA	Node-00632 - Long	NA	Node-00632 - Long	NA
Node-00632 - Lat	NA	Node-00632 - Lat	NA	Node-00632 - Lat	NA
Node-00633 - Long	NA	Node-00633 - Long	NA	Node-00633 - Long	NA
Node-00633 - Lat	NA	Node-00633 - Lat	NA	Node-00633 - Lat	NA
Node-00634 - Long	NA	Node-00634 - Long	NA	Node-00634 - Long	NA
Node-00634 - Lat	NA	Node-00634 - Lat	NA	Node-00634 - Lat	NA
Node-00635 - Long	0.999981	Node-00635 - Long	1.000012	Node-00635 - Long	NA
Node-00635 - Lat	0.999995	Node-00635 - Lat	1.000042	Node-00635 - Lat	NA
Node-00636 - Long	0.999983	Node-00636 - Long	1.000033	Node-00636 - Long	1.000031
Node-00636 - Lat	0.999983	Node-00636 - Lat	1.000033	Node-00636 - Lat	1.000016
Node-00637 - Long	1.000064	Node-00637 - Long	1.000032	Node-00637 - Long	1.000052
Node-00637 - Lat	1	Node-00637 - Lat	0.999991	Node-00637 - Lat	1.000052
Node-00638 - Long	NA	Node-00638 - Long	NA	Node-00638 - Long	1.000064
Node-00638 - Lat	NA	Node-00638 - Lat	NA	Node-00638 - Lat	0.999998
Node-00639 - Long	1.000133	Node-00639 - Long	1.000514	Node-00639 - Long	NA
Node-00639 - Lat	1.000083	Node-00639 - Lat	1.000571	Node-00639 - Lat	NA
Node-00640 - Long	0.999982	Node-00640 - Long	1.000168	Node-00640 - Long	1.000409
Node-00640 - Lat	1.000016	Node-00640 - Lat	0.999972	Node-00640 - Lat	1.000381
Node-00641 - Long	1.000005	Node-00641 - Long	0.999974	Node-00641 - Long	0.999999
Node-00641 - Lat	1.000032	Node-00641 - Lat	0.999965	Node-00641 - Lat	1.000033
Node-00642 - Long	1.00001	Node-00642 - Long	1.000017	Node-00642 - Long	1.000041
Node-00642 - Lat	1.000002	Node-00642 - Lat	1.000005	Node-00642 - Lat	1.000031
Node-00643 - Long	NA	Node-00643 - Long	NA	Node-00643 - Long	1.000028
Node-00643 - Lat	NA	Node-00643 - Lat	NA	Node-00643 - Lat	1.000089
Node-00644 - Long	NA	Node-00644 - Long	NA	Node-00644 - Long	NA

Node-00644 - Lat	NA	Node-00644 - Lat	NA	Node-00644 - Lat	NA
Node-00645 - Long	NA	Node-00645 - Long	NA	Node-00645 - Long	NA
Node-00645 - Lat	NA	Node-00645 - Lat	NA	Node-00645 - Lat	NA
Node-00646 - Long	1.000029	Node-00646 - Long	0.999991	Node-00646 - Long	NA
Node-00646 - Lat	1.000029	Node-00646 - Lat	0.999991	Node-00646 - Lat	NA
Node-00647 - Long	NA	Node-00647 - Long	NA	Node-00647 - Long	0.999991
Node-00647 - Lat	NA	Node-00647 - Lat	NA	Node-00647 - Lat	0.999991
Node-00648 - Long	1.000025	Node-00648 - Long	1.000011	Node-00648 - Long	NA
Node-00648 - Lat	0.999997	Node-00648 - Lat	1.000006	Node-00648 - Lat	NA
Node-00649 - Long	NA	Node-00649 - Long	NA	Node-00649 - Long	1.000111
Node-00649 - Lat	NA	Node-00649 - Lat	NA	Node-00649 - Lat	1.000017
Node-00650 - Long	0.999971	Node-00650 - Long	0.999992	Node-00650 - Long	NA
Node-00650 - Lat	0.999967	Node-00650 - Lat	0.999986	Node-00650 - Lat	NA
Node-00651 - Long	NA	Node-00651 - Long	NA	Node-00651 - Long	1.000022
Node-00651 - Lat	NA	Node-00651 - Lat	NA	Node-00651 - Lat	1.000028
Node-00652 - Long	1.000064	Node-00652 - Long	0.999996	Node-00652 - Long	NA
Node-00652 - Lat	1.000024	Node-00652 - Lat	1.000034	Node-00652 - Lat	NA
Node-00653 - Long	1.000066	Node-00653 - Long	1.000046	Node-00653 - Long	1.000073
Node-00653 - Lat	1.000041	Node-00653 - Lat	1	Node-00653 - Lat	0.999982
Node-00654 - Long	1	Node-00654 - Long	0.999995	Node-00654 - Long	0.99998
Node-00654 - Lat	1.000016	Node-00654 - Lat	1	Node-00654 - Lat	0.999982
Node-00655 - Long	1.000004	Node-00655 - Long	0.999994	Node-00655 - Long	1.00004
Node-00655 - Lat	1.000077	Node-00655 - Lat	1.000036	Node-00655 - Lat	0.999992
Node-00656 - Long	0.999966	Node-00656 - Long	1.000011	Node-00656 - Long	1.000054
Node-00656 - Lat	1.000054	Node-00656 - Lat	1.000098	Node-00656 - Lat	1.000001
Node-00657 - Long	0.999988	Node-00657 - Long	1.000048	Node-00657 - Long	1.000071
Node-00657 - Lat	0.999982	Node-00657 - Lat	1.000087	Node-00657 - Lat	0.999996
Node-00658 - Long	1.000052	Node-00658 - Long	1.000012	Node-00658 - Long	1.000073
Node-00658 - Lat	0.99998	Node-00658 - Lat	1.000187	Node-00658 - Lat	0.999995
Node-00659 - Long	NA	Node-00659 - Long	NA	Node-00659 - Long	1.000037
Node-00659 - Lat	NA	Node-00659 - Lat	NA	Node-00659 - Lat	1.000055
Node-00660 - Long	1.000035	Node-00660 - Long	1.000013	Node-00660 - Long	NA
Node-00660 - Lat	1.000042	Node-00660 - Lat	1.000073	Node-00660 - Lat	NA
Node-00661 - Long	0.999998	Node-00661 - Long	1.000001	Node-00661 - Long	1.00001
Node-00661 - Lat	1.000036	Node-00661 - Lat	0.999977	Node-00661 - Lat	0.999996
Node-00662 - Long	NA	Node-00662 - Long	NA	Node-00662 - Long	1.000063
Node-00662 - Lat	NA	Node-00662 - Lat	NA	Node-00662 - Lat	0.999995
Node-00663 - Long	NA	Node-00663 - Long	NA	Node-00663 - Long	NA
Node-00663 - Lat	NA	Node-00663 - Lat	NA	Node-00663 - Lat	NA
Node-00664 - Long	NA	Node-00664 - Long	NA	Node-00664 - Long	NA
Node-00664 - Lat	NA	Node-00664 - Lat	NA	Node-00664 - Lat	NA
Node-00665 - Long	NA	Node-00665 - Long	NA	Node-00665 - Long	NA
Node-00665 - Lat	NA	Node-00665 - Lat	NA	Node-00665 - Lat	NA
Node-00666 - Long	1.000004	Node-00666 - Long	1.000048	Node-00666 - Long	NA
Node-00666 - Lat	0.999989	Node-00666 - Lat	1.000054	Node-00666 - Lat	NA
Node-00667 - Long	1.000006	Node-00667 - Long	0.999975	Node-00667 - Long	1.000023
Node-00667 - Lat	0.999989	Node-00667 - Lat	0.999978	Node-00667 - Lat	1.000007
Node-00668 - Long	NA	Node-00668 - Long	NA	Node-00668 - Long	0.999977

Node-00668 - Lat	NA	Node-00668 - Lat	NA	Node-00668 - Lat	1.00005
Node-00669 - Long	NA	Node-00669 - Long	NA	Node-00669 - Long	NA
Node-00669 - Lat	NA	Node-00669 - Lat	NA	Node-00669 - Lat	NA
Node-00670 - Long	1.000114	Node-00670 - Long	1.000065	Node-00670 - Long	NA
Node-00670 - Lat	1.000008	Node-00670 - Lat	1	Node-00670 - Lat	NA
Node-00671 - Long	NA	Node-00671 - Long	NA	Node-00671 - Long	1.000048
Node-00671 - Lat	NA	Node-00671 - Lat	NA	Node-00671 - Lat	0.99998
Node-00672 - Long	1.000059	Node-00672 - Long	0.999992	Node-00672 - Long	NA
Node-00672 - Lat	1.000039	Node-00672 - Lat	1.000032	Node-00672 - Lat	NA
Node-00673 - Long	1.000019	Node-00673 - Long	1.000003	Node-00673 - Long	1.000043
Node-00673 - Lat	1.000087	Node-00673 - Lat	1.000114	Node-00673 - Lat	1.00002
Node-00674 - Long	0.999976	Node-00674 - Long	1	Node-00674 - Long	0.999983
Node-00674 - Lat	1.000029	Node-00674 - Lat	1.000119	Node-00674 - Lat	1.000004
Node-00675 - Long	1.000012	Node-00675 - Long	1.00003	Node-00675 - Long	0.99999
Node-00675 - Lat	1.000075	Node-00675 - Lat	1.000025	Node-00675 - Lat	0.999987
Node-00676 - Long	0.999996	Node-00676 - Long	0.999987	Node-00676 - Long	1.000033
Node-00676 - Lat	1.000118	Node-00676 - Lat	1.000101	Node-00676 - Lat	0.999984
Node-00677 - Long	1.00008	Node-00677 - Long	0.999989	Node-00677 - Long	1.000062
Node-00677 - Lat	1.00008	Node-00677 - Lat	0.999989	Node-00677 - Lat	1.000053
Node-00678 - Long	NA	Node-00678 - Long	NA	Node-00678 - Long	1.00001
Node-00678 - Lat	NA	Node-00678 - Lat	NA	Node-00678 - Lat	1.00001
Node-00679 - Long	0.999996	Node-00679 - Long	0.999983	Node-00679 - Long	NA
Node-00679 - Lat	1.000003	Node-00679 - Lat	1.000025	Node-00679 - Lat	NA
Node-00680 - Long	NA	Node-00680 - Long	NA	Node-00680 - Long	1.000009
Node-00680 - Lat	NA	Node-00680 - Lat	NA	Node-00680 - Lat	0.999984
Node-00681 - Long	NA	Node-00681 - Long	NA	Node-00681 - Long	NA
Node-00681 - Lat	NA	Node-00681 - Lat	NA	Node-00681 - Lat	NA
Node-00682 - Long	NA	Node-00682 - Long	NA	Node-00682 - Long	NA
Node-00682 - Lat	NA	Node-00682 - Lat	NA	Node-00682 - Lat	NA
Node-00683 - Long	NA	Node-00683 - Long	NA	Node-00683 - Long	NA
Node-00683 - Lat	NA	Node-00683 - Lat	NA	Node-00683 - Lat	NA
Node-00684 - Long	NA	Node-00684 - Long	NA	Node-00684 - Long	NA
Node-00684 - Lat	NA	Node-00684 - Lat	NA	Node-00684 - Lat	NA
Node-00685 - Long	NA	Node-00685 - Long	NA	Node-00685 - Long	NA
Node-00685 - Lat	NA	Node-00685 - Lat	NA	Node-00685 - Lat	NA
Node-00686 - Long	NA	Node-00686 - Long	NA	Node-00686 - Long	NA
Node-00686 - Lat	NA	Node-00686 - Lat	NA	Node-00686 - Lat	NA
Node-00687 - Long	1.000026	Node-00687 - Long	1.000004	Node-00687 - Long	NA
Node-00687 - Lat	1.000033	Node-00687 - Lat	1.00001	Node-00687 - Lat	NA
Node-00688 - Long	NA	Node-00688 - Long	NA	Node-00688 - Long	1.000014
Node-00688 - Lat	NA	Node-00688 - Lat	NA	Node-00688 - Lat	1.000052
Node-00689 - Long	NA	Node-00689 - Long	NA	Node-00689 - Long	NA
Node-00689 - Lat	NA	Node-00689 - Lat	NA	Node-00689 - Lat	NA
Node-00690 - Long	1.000024	Node-00690 - Long	1.000037	Node-00690 - Long	NA
Node-00690 - Lat	1.000054	Node-00690 - Lat	1.000046	Node-00690 - Lat	NA
Node-00691 - Long	1.000049	Node-00691 - Long	1.00001	Node-00691 - Long	1.000027
Node-00691 - Lat	1.000057	Node-00691 - Lat	1.000123	Node-00691 - Lat	1.000005
Node-00692 - Long	NA	Node-00692 - Long	NA	Node-00692 - Long	1.000022

Node-00692 - Lat	NA	Node-00692 - Lat	NA	Node-00692 - Lat	0.99999
Node-00693 - Long	1.000043	Node-00693 - Long	1.000029	Node-00693 - Long	NA
Node-00693 - Lat	1.000018	Node-00693 - Lat	1.00006	Node-00693 - Lat	NA
Node-00694 - Long	0.999991	Node-00694 - Long	0.999984	Node-00694 - Long	1.000054
Node-00694 - Lat	1.000044	Node-00694 - Lat	1.000012	Node-00694 - Lat	1.000009
Node-00695 - Long	1.000014	Node-00695 - Long	0.999987	Node-00695 - Long	0.999986
Node-00695 - Lat	1.000029	Node-00695 - Lat	1.000039	Node-00695 - Lat	1.000001
Node-00696 - Long	0.999964	Node-00696 - Long	1.000156	Node-00696 - Long	1.000082
Node-00696 - Lat	1.000021	Node-00696 - Lat	1.000099	Node-00696 - Lat	1.000047
Node-00697 - Long	1.000004	Node-00697 - Long	0.999978	Node-00697 - Long	1.000004
Node-00697 - Lat	0.999992	Node-00697 - Lat	0.999991	Node-00697 - Lat	0.999999
Node-00698 - Long	NA	Node-00698 - Long	NA	Node-00698 - Long	0.999973
Node-00698 - Lat	NA	Node-00698 - Lat	NA	Node-00698 - Lat	0.999967
Node-00699 - Long	NA	Node-00699 - Long	NA	Node-00699 - Long	NA
Node-00699 - Lat	NA	Node-00699 - Lat	NA	Node-00699 - Lat	NA
Node-00700 - Long	0.999997	Node-00700 - Long	1.000021	Node-00700 - Long	NA
Node-00700 - Lat	1.000057	Node-00700 - Lat	1.000054	Node-00700 - Lat	NA
Node-00701 - Long	1.000044	Node-00701 - Long	0.999996	Node-00701 - Long	1.000034
Node-00701 - Lat	1.000009	Node-00701 - Lat	1.000044	Node-00701 - Lat	1.000113
Node-00702 - Long	1.000229	Node-00702 - Long	1.000041	Node-00702 - Long	0.99996
Node-00702 - Lat	1.000229	Node-00702 - Lat	1.000041	Node-00702 - Lat	1.000011
Node-00703 - Long	NA	Node-00703 - Long	NA	Node-00703 - Long	1.000318
Node-00703 - Lat	NA	Node-00703 - Lat	NA	Node-00703 - Lat	1.000318
Node-00704 - Long	NA	Node-00704 - Long	NA	Node-00704 - Long	NA
Node-00704 - Lat	NA	Node-00704 - Lat	NA	Node-00704 - Lat	NA
Node-00705 - Long	0.999987	Node-00705 - Long	1.000025	Node-00705 - Long	NA
Node-00705 - Lat	1.000019	Node-00705 - Lat	1.000059	Node-00705 - Lat	NA
Node-00706 - Long	1.00002	Node-00706 - Long	1.000054	Node-00706 - Long	0.999988
Node-00706 - Lat	1.000051	Node-00706 - Lat	1.000006	Node-00706 - Lat	1
Node-00707 - Long	1.000024	Node-00707 - Long	1.000032	Node-00707 - Long	1
Node-00707 - Lat	1.000031	Node-00707 - Lat	1.000017	Node-00707 - Lat	1.000018
Node-00708 - Long	0.999991	Node-00708 - Long	1.000023	Node-00708 - Long	1
Node-00708 - Lat	1.000023	Node-00708 - Lat	1.00009	Node-00708 - Lat	1.000004
Node-00709 - Long	0.999975	Node-00709 - Long	0.999976	Node-00709 - Long	1.000036
Node-00709 - Lat	1.000047	Node-00709 - Lat	1.000036	Node-00709 - Lat	1.000025
Node-00710 - Long	0.999956	Node-00710 - Long	0.999981	Node-00710 - Long	0.999978
Node-00710 - Lat	0.999983	Node-00710 - Lat	1.000002	Node-00710 - Lat	1.000084
Node-00711 - Long	1.000007	Node-00711 - Long	1.000048	Node-00711 - Long	1.000024
Node-00711 - Lat	1.000038	Node-00711 - Lat	1.000034	Node-00711 - Lat	1.000042
Node-00712 - Long	0.999997	Node-00712 - Long	1.000012	Node-00712 - Long	0.999967
Node-00712 - Lat	0.999984	Node-00712 - Lat	1.000013	Node-00712 - Lat	0.999979
Node-00713 - Long	1.000075	Node-00713 - Long	1.000035	Node-00713 - Long	1.000033
Node-00713 - Lat	0.999963	Node-00713 - Lat	1.000047	Node-00713 - Lat	1.000025
Node-00714 - Long	0.99998	Node-00714 - Long	1.000007	Node-00714 - Long	0.999997
Node-00714 - Lat	1.000012	Node-00714 - Lat	0.999982	Node-00714 - Lat	1.000016
Node-00715 - Long	0.999989	Node-00715 - Long	1.000042	Node-00715 - Long	0.999999
Node-00715 - Lat	0.999974	Node-00715 - Lat	1.000017	Node-00715 - Lat	0.999982
Node-00716 - Long	0.999977	Node-00716 - Long	0.999976	Node-00716 - Long	1.000022

Node-00716 - Lat	1.000019	Node-00716 - Lat	0.999981	Node-00716 - Lat	0.999979
Node-00717 - Long	NA	Node-00717 - Long	NA	Node-00717 - Long	1.000054
Node-00717 - Lat	NA	Node-00717 - Lat	NA	Node-00717 - Lat	0.99999
Node-00718 - Long	1.000091	Node-00718 - Long	1.000022	Node-00718 - Long	NA
Node-00718 - Lat	1.000021	Node-00718 - Lat	1.000016	Node-00718 - Lat	NA
Node-00719 - Long	1.000114	Node-00719 - Long	1.000026	Node-00719 - Long	1.000043
Node-00719 - Lat	1.000114	Node-00719 - Lat	1.000026	Node-00719 - Lat	1.000006
Node-00720 - Long	NA	Node-00720 - Long	NA	Node-00720 - Long	1.000018
Node-00720 - Lat	NA	Node-00720 - Lat	NA	Node-00720 - Lat	1.000018
Node-00721 - Long	1.000072	Node-00721 - Long	1.000019	Node-00721 - Long	NA
Node-00721 - Lat	1.000025	Node-00721 - Lat	1	Node-00721 - Lat	NA
Node-00722 - Long	1.000025	Node-00722 - Long	1.000014	Node-00722 - Long	1.000002
Node-00722 - Lat	1.000025	Node-00722 - Lat	1.000014	Node-00722 - Lat	1.000131
Node-00723 - Long	NA	Node-00723 - Long	NA	Node-00723 - Long	1.000068
Node-00723 - Lat	NA	Node-00723 - Lat	NA	Node-00723 - Lat	1.000068
Node-00724 - Long	1.000003	Node-00724 - Long	1.000032	Node-00724 - Long	NA
Node-00724 - Lat	0.999997	Node-00724 - Lat	1.000069	Node-00724 - Lat	NA
Node-00725 - Long	1.000004	Node-00725 - Long	0.99999	Node-00725 - Long	1.00003
Node-00725 - Lat	1.000026	Node-00725 - Lat	1.000001	Node-00725 - Lat	0.999999
Node-00726 - Long	NA	Node-00726 - Long	NA	Node-00726 - Long	1.000005
Node-00726 - Lat	NA	Node-00726 - Lat	NA	Node-00726 - Lat	0.999998
Node-00727 - Long	NA	Node-00727 - Long	NA	Node-00727 - Long	NA
Node-00727 - Lat	NA	Node-00727 - Lat	NA	Node-00727 - Lat	NA
Node-00728 - Long	0.999976	Node-00728 - Long	1.000009	Node-00728 - Long	NA
Node-00728 - Lat	0.999989	Node-00728 - Lat	1.000038	Node-00728 - Lat	NA
Node-00729 - Long	NA	Node-00729 - Long	NA	Node-00729 - Long	0.999987
Node-00729 - Lat	NA	Node-00729 - Lat	NA	Node-00729 - Lat	1.000089
Node-00730 - Long	NA	Node-00730 - Long	NA	Node-00730 - Long	NA
Node-00730 - Lat	NA	Node-00730 - Lat	NA	Node-00730 - Lat	NA
Node-00731 - Long	1.001171	Node-00731 - Long	1.000429	Node-00731 - Long	NA
Node-00731 - Lat	1.000055	Node-00731 - Lat	0.99998	Node-00731 - Lat	NA
Node-00732 - Long	NA	Node-00732 - Long	NA	Node-00732 - Long	1.001092
Node-00732 - Lat	NA	Node-00732 - Lat	NA	Node-00732 - Lat	1.000047
Node-00733 - Long	1.000005	Node-00733 - Long	1.000022	Node-00733 - Long	NA
Node-00733 - Lat	0.999988	Node-00733 - Lat	1.000018	Node-00733 - Lat	NA
Node-00734 - Long	NA	Node-00734 - Long	NA	Node-00734 - Long	1.000046
Node-00734 - Lat	NA	Node-00734 - Lat	NA	Node-00734 - Lat	1.000006
Node-00735 - Long	0.999983	Node-00735 - Long	1.000079	Node-00735 - Long	NA
Node-00735 - Lat	1.000004	Node-00735 - Lat	0.999991	Node-00735 - Lat	NA
Node-00736 - Long	NA	Node-00736 - Long	NA	Node-00736 - Long	0.999982
Node-00736 - Lat	NA	Node-00736 - Lat	NA	Node-00736 - Lat	1.000048
Node-00737 - Long	NA	Node-00737 - Long	NA	Node-00737 - Long	NA
Node-00737 - Lat	NA	Node-00737 - Lat	NA	Node-00737 - Lat	NA
Node-00738 - Long	NA	Node-00738 - Long	NA	Node-00738 - Long	NA
Node-00738 - Lat	NA	Node-00738 - Lat	NA	Node-00738 - Lat	NA
Node-00739 - Long	1.000052	Node-00739 - Long	0.999974	Node-00739 - Long	NA
Node-00739 - Lat	1.000018	Node-00739 - Lat	1.000003	Node-00739 - Lat	NA
Node-00740 - Long	1.000044	Node-00740 - Long	1.000023	Node-00740 - Long	1.000033

Node-00740 - Lat	0.999978	Node-00740 - Lat	1.000032	Node-00740 - Lat	1.000007
Node-00741 - Long	NA	Node-00741 - Long	NA	Node-00741 - Long	0.999979
Node-00741 - Lat	NA	Node-00741 - Lat	NA	Node-00741 - Lat	1.000006
Node-00742 - Long	1.000012	Node-00742 - Long	0.99999	Node-00742 - Long	NA
Node-00742 - Lat	1.000017	Node-00742 - Lat	0.999985	Node-00742 - Lat	NA
Node-00743 - Long	NA	Node-00743 - Long	NA	Node-00743 - Long	0.999997
Node-00743 - Lat	NA	Node-00743 - Lat	NA	Node-00743 - Lat	0.999992
Node-00744 - Long	0.999996	Node-00744 - Long	0.999966	Node-00744 - Long	NA
Node-00744 - Lat	1.000072	Node-00744 - Lat	1.00003	Node-00744 - Lat	NA
Node-00745 - Long	0.999995	Node-00745 - Long	0.999985	Node-00745 - Long	1
Node-00745 - Lat	1.000095	Node-00745 - Lat	1.000066	Node-00745 - Lat	1.00003
Node-00746 - Long	NA	Node-00746 - Long	NA	Node-00746 - Long	0.999979
Node-00746 - Lat	NA	Node-00746 - Lat	NA	Node-00746 - Lat	1.000072
Node-00747 - Long	1.000017	Node-00747 - Long	1.000033	Node-00747 - Long	NA
Node-00747 - Lat	1.000065	Node-00747 - Lat	1.000033	Node-00747 - Lat	NA
Node-00748 - Long	NA	Node-00748 - Long	NA	Node-00748 - Long	1.000008
Node-00748 - Lat	NA	Node-00748 - Lat	NA	Node-00748 - Lat	1.000005
Node-00749 - Long	0.999994	Node-00749 - Long	1.000021	Node-00749 - Long	NA
Node-00749 - Lat	0.999996	Node-00749 - Lat	1.000096	Node-00749 - Lat	NA
Node-00750 - Long	NA	Node-00750 - Long	NA	Node-00750 - Long	1.000055
Node-00750 - Lat	NA	Node-00750 - Lat	NA	Node-00750 - Lat	0.999985
Node-00751 - Long	0.999985	Node-00751 - Long	0.999998	Node-00751 - Long	NA
Node-00751 - Lat	1.000021	Node-00751 - Lat	1.000076	Node-00751 - Lat	NA
Node-00752 - Long	1.000035	Node-00752 - Long	0.999985	Node-00752 - Long	1.000223
Node-00752 - Lat	1.000035	Node-00752 - Lat	0.999985	Node-00752 - Lat	1.000001
Node-00753 - Long	1.000033	Node-00753 - Long	0.999959	Node-00753 - Long	1.000039
Node-00753 - Lat	1.000075	Node-00753 - Lat	0.999968	Node-00753 - Lat	1.000039
				Node-00754 - Long	0.999983
				Node-00754 - Lat	1.000024

both_bm	both_bm
param	psrf
Lh	1.000013
Scale	0.999982
Node-00000 - Long	1.000031
Node-00000 - Lat	1.000042
Node-00001 - Long	0.999993
Node-00001 - Lat	1.000078
Node-00002 - Long	1.000109
Node-00002 - Lat	1.000164
Node-00003 - Long	1.00007
Node-00003 - Lat	1.000187
Node-00004 - Long	1.000024
Node-00004 - Lat	1.000089
Node-00005 - Long	1.000027
Node-00005 - Lat	1.000093
Node-00006 - Long	1.000039
Node-00006 - Lat	0.999987
Node-00007 - Long	0.999994
Node-00007 - Lat	1.000025
Node-00008 - Long	1.000062
Node-00008 - Lat	1.000023
Node-00009 - Long	1.000013
Node-00009 - Lat	1.000015
Node-00010 - Long	1.000021
Node-00010 - Lat	1.000017
Node-00011 - Long	0.999991
Node-00011 - Lat	1.00007
Node-00012 - Long	0.999998
Node-00012 - Lat	1.00011
Node-00013 - Long	0.999975
Node-00013 - Lat	1.000086
Node-00014 - Long	0.999982
Node-00014 - Lat	1.000027
Node-00015 - Long	1.000001
Node-00015 - Lat	1.000058
Node-00016 - Long	0.999984
Node-00016 - Lat	1.000017
Node-00017 - Long	1.00006
Node-00017 - Lat	0.999982
Node-00018 - Long	1.000012
Node-00018 - Lat	0.999998
Node-00019 - Long	1.000046
Node-00019 - Lat	0.999985
Node-00020 - Long	1.00006

Node-00020 - Lat	1
Node-00021 - Long	1.000057
Node-00021 - Lat	1.000052
Node-00022 - Long	1.000062
Node-00022 - Lat	0.999999
Node-00023 - Long	1.000033
Node-00023 - Lat	1.000042
Node-00024 - Long	1.000176
Node-00024 - Lat	0.999997
Node-00025 - Long	1.000052
Node-00025 - Lat	1.000019
Node-00026 - Long	1.000046
Node-00026 - Lat	1.000011
Node-00027 - Long	1.00005
Node-00027 - Lat	0.999985
Node-00028 - Long	0.99999
Node-00028 - Lat	0.999996
Node-00029 - Long	1.000014
Node-00029 - Lat	1.000003
Node-00030 - Long	1.000007
Node-00030 - Lat	1.000026
Node-00031 - Long	1.000028
Node-00031 - Lat	1.000002
Node-00032 - Long	1.000038
Node-00032 - Lat	1.000027
Node-00033 - Long	1.000037
Node-00033 - Lat	0.999997
Node-00034 - Long	1.000043
Node-00034 - Lat	1.00004
Node-00035 - Long	1.000003
Node-00035 - Lat	1.00013
Node-00036 - Long	1.000017
Node-00036 - Lat	1.00013
Node-00037 - Long	0.999999
Node-00037 - Lat	1.000056
Node-00038 - Long	1.000016
Node-00038 - Lat	1.000023
Node-00039 - Long	1.000066
Node-00039 - Lat	1.00002
Node-00040 - Long	1.000012
Node-00040 - Lat	1.000003
Node-00041 - Long	1.000035
Node-00041 - Lat	0.999996
Node-00042 - Long	1.000051
Node-00042 - Lat	1.000007
Node-00043 - Long	0.999978
Node-00043 - Lat	1.000031
Node-00044 - Long	1.000025

Node-00044 - Lat	1.000121
Node-00045 - Long	0.999972
Node-00045 - Lat	1.000066
Node-00046 - Long	0.999993
Node-00046 - Lat	1.000008
Node-00047 - Long	NA
Node-00047 - Lat	NA
Node-00048 - Long	NA
Node-00048 - Lat	NA
Node-00049 - Long	NA
Node-00049 - Lat	NA
Node-00050 - Long	1.000065
Node-00050 - Lat	1.000062
Node-00051 - Long	1.000072
Node-00051 - Lat	1.000001
Node-00052 - Long	0.999992
Node-00052 - Lat	0.999989
Node-00053 - Long	0.999998
Node-00053 - Lat	0.999981
Node-00054 - Long	0.999989
Node-00054 - Lat	0.999967
Node-00055 - Long	0.999984
Node-00055 - Lat	0.99998
Node-00056 - Long	1.000015
Node-00056 - Lat	0.99998
Node-00057 - Long	NA
Node-00057 - Lat	NA
Node-00058 - Long	NA
Node-00058 - Lat	NA
Node-00059 - Long	NA
Node-00059 - Lat	NA
Node-00060 - Long	0.999976
Node-00060 - Lat	0.999989
Node-00061 - Long	0.999997
Node-00061 - Lat	0.99998
Node-00062 - Long	1.000003
Node-00062 - Lat	0.999998
Node-00063 - Long	1.000014
Node-00063 - Lat	1.000064
Node-00064 - Long	1.000033
Node-00064 - Lat	1.000053
Node-00065 - Long	1.000089
Node-00065 - Lat	0.999986
Node-00066 - Long	NA
Node-00066 - Lat	NA
Node-00067 - Long	NA
Node-00067 - Lat	NA
Node-00068 - Long	NA

Node-00068 - Lat	NA
Node-00069 - Long	NA
Node-00069 - Lat	NA
Node-00070 - Long	NA
Node-00070 - Lat	NA
Node-00071 - Long	NA
Node-00071 - Lat	NA
Node-00072 - Long	1.000028
Node-00072 - Lat	1.000096
Node-00073 - Long	0.999989
Node-00073 - Lat	1.00004
Node-00074 - Long	1.000149
Node-00074 - Lat	1.000042
Node-00075 - Long	NA
Node-00075 - Lat	NA
Node-00076 - Long	NA
Node-00076 - Lat	NA
Node-00077 - Long	NA
Node-00077 - Lat	NA
Node-00078 - Long	NA
Node-00078 - Lat	NA
Node-00079 - Long	NA
Node-00079 - Lat	NA
Node-00080 - Long	NA
Node-00080 - Lat	NA
Node-00081 - Long	1.000016
Node-00081 - Lat	1.00001
Node-00082 - Long	1.00003
Node-00082 - Lat	1.000071
Node-00083 - Long	NA
Node-00083 - Lat	NA
Node-00084 - Long	NA
Node-00084 - Lat	NA
Node-00085 - Long	1.000018
Node-00085 - Lat	0.999994
Node-00086 - Long	NA
Node-00086 - Lat	NA
Node-00087 - Long	NA
Node-00087 - Lat	NA
Node-00088 - Long	NA
Node-00088 - Lat	NA
Node-00089 - Long	0.999961
Node-00089 - Lat	1.000021
Node-00090 - Long	NA
Node-00090 - Lat	NA
Node-00091 - Long	1.000082
Node-00091 - Lat	1.000028
Node-00092 - Long	NA

Node-00092 - Lat	NA
Node-00093 - Long	NA
Node-00093 - Lat	NA
Node-00094 - Long	1.000023
Node-00094 - Lat	1.00004
Node-00095 - Long	NA
Node-00095 - Lat	NA
Node-00096 - Long	NA
Node-00096 - Lat	NA
Node-00097 - Long	NA
Node-00097 - Lat	NA
Node-00098 - Long	1.000111
Node-00098 - Lat	0.999996
Node-00099 - Long	1.000069
Node-00099 - Lat	0.999992
Node-00100 - Long	1.001173
Node-00100 - Lat	1.000025
Node-00101 - Long	NA
Node-00101 - Lat	NA
Node-00102 - Long	NA
Node-00102 - Lat	NA
Node-00103 - Long	NA
Node-00103 - Lat	NA
Node-00104 - Long	0.999975
Node-00104 - Lat	0.999972
Node-00105 - Long	0.999958
Node-00105 - Lat	0.999958
Node-00106 - Long	1.000068
Node-00106 - Lat	1.00006
Node-00107 - Long	1.000023
Node-00107 - Lat	1.000043
Node-00108 - Long	1.000009
Node-00108 - Lat	0.999997
Node-00109 - Long	NA
Node-00109 - Lat	NA
Node-00110 - Long	0.999998
Node-00110 - Lat	0.999979
Node-00111 - Long	NA
Node-00111 - Lat	NA
Node-00112 - Long	1.000036
Node-00112 - Lat	1.000051
Node-00113 - Long	1.000017
Node-00113 - Lat	1.000005
Node-00114 - Long	0.999989
Node-00114 - Lat	1.000017
Node-00115 - Long	NA
Node-00115 - Lat	NA
Node-00116 - Long	NA

Node-00116 - Lat	NA
Node-00117 - Long	1.000051
Node-00117 - Lat	1.000018
Node-00118 - Long	0.999998
Node-00118 - Lat	1.000019
Node-00119 - Long	NA
Node-00119 - Lat	NA
Node-00120 - Long	NA
Node-00120 - Lat	NA
Node-00121 - Long	1.000011
Node-00121 - Lat	1.000054
Node-00122 - Long	NA
Node-00122 - Lat	NA
Node-00123 - Long	NA
Node-00123 - Lat	NA
Node-00124 - Long	1.000018
Node-00124 - Lat	1.000057
Node-00125 - Long	1.00001
Node-00125 - Lat	1.00001
Node-00126 - Long	NA
Node-00126 - Lat	NA
Node-00127 - Long	1.000036
Node-00127 - Lat	1.000007
Node-00128 - Long	0.999987
Node-00128 - Lat	0.999987
Node-00129 - Long	1.000062
Node-00129 - Lat	1.00007
Node-00130 - Long	NA
Node-00130 - Lat	NA
Node-00131 - Long	NA
Node-00131 - Lat	NA
Node-00132 - Long	1.000064
Node-00132 - Lat	1.000058
Node-00133 - Long	NA
Node-00133 - Lat	NA
Node-00134 - Long	1.000097
Node-00134 - Lat	1.000001
Node-00135 - Long	NA
Node-00135 - Lat	NA
Node-00136 - Long	NA
Node-00136 - Lat	NA
Node-00137 - Long	1.000036
Node-00137 - Lat	1.000063
Node-00138 - Long	NA
Node-00138 - Lat	NA
Node-00139 - Long	NA
Node-00139 - Lat	NA
Node-00140 - Long	1.00012

Node-00140 - Lat	1.000041
Node-00141 - Long	NA
Node-00141 - Lat	NA
Node-00142 - Long	NA
Node-00142 - Lat	NA
Node-00143 - Long	1.000054
Node-00143 - Lat	1.000045
Node-00144 - Long	NA
Node-00144 - Lat	NA
Node-00145 - Long	NA
Node-00145 - Lat	NA
Node-00146 - Long	1.000046
Node-00146 - Lat	1.000022
Node-00147 - Long	1.000004
Node-00147 - Lat	0.999995
Node-00148 - Long	1.000098
Node-00148 - Lat	1.000151
Node-00149 - Long	NA
Node-00149 - Lat	NA
Node-00150 - Long	NA
Node-00150 - Lat	NA
Node-00151 - Long	NA
Node-00151 - Lat	NA
Node-00152 - Long	NA
Node-00152 - Lat	NA
Node-00153 - Long	NA
Node-00153 - Lat	NA
Node-00154 - Long	1.000052
Node-00154 - Lat	1.000003
Node-00155 - Long	NA
Node-00155 - Lat	NA
Node-00156 - Long	NA
Node-00156 - Lat	NA
Node-00157 - Long	0.999998
Node-00157 - Lat	0.999996
Node-00158 - Long	1.000004
Node-00158 - Lat	1.000043
Node-00159 - Long	NA
Node-00159 - Lat	NA
Node-00160 - Long	0.999976
Node-00160 - Lat	1.000018
Node-00161 - Long	0.999972
Node-00161 - Lat	1.000041
Node-00162 - Long	0.99999
Node-00162 - Lat	0.999999
Node-00163 - Long	1.000022
Node-00163 - Lat	0.999985
Node-00164 - Long	1.000023

Node-00164 - Lat	0.99999
Node-00165 - Long	1.000001
Node-00165 - Lat	1
Node-00166 - Long	1.000006
Node-00166 - Lat	1.000005
Node-00167 - Long	0.999986
Node-00167 - Lat	1.000025
Node-00168 - Long	0.999969
Node-00168 - Lat	1.000045
Node-00169 - Long	0.999996
Node-00169 - Lat	0.999995
Node-00170 - Long	0.999989
Node-00170 - Lat	0.999993
Node-00171 - Long	1.000002
Node-00171 - Lat	1.000087
Node-00172 - Long	1.000001
Node-00172 - Lat	1.000077
Node-00173 - Long	1.000018
Node-00173 - Lat	0.99999
Node-00174 - Long	1.000031
Node-00174 - Lat	1.000024
Node-00175 - Long	NA
Node-00175 - Lat	NA
Node-00176 - Long	0.999998
Node-00176 - Lat	1.000048
Node-00177 - Long	NA
Node-00177 - Lat	NA
Node-00178 - Long	NA
Node-00178 - Lat	NA
Node-00179 - Long	1.000038
Node-00179 - Lat	0.999992
Node-00180 - Long	0.999974
Node-00180 - Lat	1.00003
Node-00181 - Long	NA
Node-00181 - Lat	NA
Node-00182 - Long	NA
Node-00182 - Lat	NA
Node-00183 - Long	NA
Node-00183 - Lat	NA
Node-00184 - Long	NA
Node-00184 - Lat	NA
Node-00185 - Long	NA
Node-00185 - Lat	NA
Node-00186 - Long	1.000004
Node-00186 - Lat	1.000028
Node-00187 - Long	NA
Node-00187 - Lat	NA
Node-00188 - Long	1.000004

Node-00188 - Lat	0.999997
Node-00189 - Long	1.000019
Node-00189 - Lat	1.000075
Node-00190 - Long	NA
Node-00190 - Lat	NA
Node-00191 - Long	NA
Node-00191 - Lat	NA
Node-00192 - Long	1.000005
Node-00192 - Lat	1.000018
Node-00193 - Long	0.999976
Node-00193 - Lat	1.000039
Node-00194 - Long	1.00003
Node-00194 - Lat	1.000005
Node-00195 - Long	0.999985
Node-00195 - Lat	1.000001
Node-00196 - Long	0.999997
Node-00196 - Lat	1.00003
Node-00197 - Long	NA
Node-00197 - Lat	NA
Node-00198 - Long	1.002915
Node-00198 - Lat	1.002843
Node-00199 - Long	NA
Node-00199 - Lat	NA
Node-00200 - Long	NA
Node-00200 - Lat	NA
Node-00201 - Long	1.000032
Node-00201 - Lat	1.000004
Node-00202 - Long	1.000018
Node-00202 - Lat	1.000018
Node-00203 - Long	NA
Node-00203 - Lat	NA
Node-00204 - Long	NA
Node-00204 - Lat	NA
Node-00205 - Long	1.000036
Node-00205 - Lat	1.000036
Node-00206 - Long	NA
Node-00206 - Lat	NA
Node-00207 - Long	NA
Node-00207 - Lat	NA
Node-00208 - Long	NA
Node-00208 - Lat	NA
Node-00209 - Long	NA
Node-00209 - Lat	NA
Node-00210 - Long	0.999992
Node-00210 - Lat	0.999992
Node-00211 - Long	NA
Node-00211 - Lat	NA
Node-00212 - Long	1.000059

Node-00212 - Lat	0.999979
Node-00213 - Long	1.000086
Node-00213 - Lat	0.999975
Node-00214 - Long	0.999996
Node-00214 - Lat	0.999967
Node-00215 - Long	1.000036
Node-00215 - Lat	1.00003
Node-00216 - Long	NA
Node-00216 - Lat	NA
Node-00217 - Long	NA
Node-00217 - Lat	NA
Node-00218 - Long	1.000036
Node-00218 - Lat	1.000016
Node-00219 - Long	NA
Node-00219 - Lat	NA
Node-00220 - Long	0.999965
Node-00220 - Lat	0.999965
Node-00221 - Long	NA
Node-00221 - Lat	NA
Node-00222 - Long	NA
Node-00222 - Lat	NA
Node-00223 - Long	NA
Node-00223 - Lat	NA
Node-00224 - Long	NA
Node-00224 - Lat	NA
Node-00225 - Long	1.000059
Node-00225 - Lat	1.000065
Node-00226 - Long	1.000028
Node-00226 - Lat	1.000075
Node-00227 - Long	1.000002
Node-00227 - Lat	1.000047
Node-00228 - Long	0.999995
Node-00228 - Lat	1.000006
Node-00229 - Long	1.000083
Node-00229 - Lat	1.000024
Node-00230 - Long	1.000066
Node-00230 - Lat	1.000052
Node-00231 - Long	1.000015
Node-00231 - Lat	1.000043
Node-00232 - Long	1.000115
Node-00232 - Lat	1.00003
Node-00233 - Long	1.000019
Node-00233 - Lat	1.000143
Node-00234 - Long	0.999992
Node-00234 - Lat	1.000015
Node-00235 - Long	1.000009
Node-00235 - Lat	1.000114
Node-00236 - Long	1.000008

Node-00236 - Lat	1.00007
Node-00237 - Long	1.000004
Node-00237 - Lat	1.000074
Node-00238 - Long	0.999994
Node-00238 - Lat	1.000131
Node-00239 - Long	1.000041
Node-00239 - Lat	1.000042
Node-00240 - Long	1.000016
Node-00240 - Lat	1.000004
Node-00241 - Long	NA
Node-00241 - Lat	NA
Node-00242 - Long	NA
Node-00242 - Lat	NA
Node-00243 - Long	NA
Node-00243 - Lat	NA
Node-00244 - Long	1.000068
Node-00244 - Lat	0.999972
Node-00245 - Long	NA
Node-00245 - Lat	NA
Node-00246 - Long	NA
Node-00246 - Lat	NA
Node-00247 - Long	0.999988
Node-00247 - Lat	0.999988
Node-00248 - Long	NA
Node-00248 - Lat	NA
Node-00249 - Long	1.000019
Node-00249 - Lat	1.000066
Node-00250 - Long	0.999998
Node-00250 - Lat	1.000138
Node-00251 - Long	NA
Node-00251 - Lat	NA
Node-00252 - Long	1.000043
Node-00252 - Lat	1.000043
Node-00253 - Long	0.999969
Node-00253 - Lat	1.000045
Node-00254 - Long	NA
Node-00254 - Lat	NA
Node-00255 - Long	1.000007
Node-00255 - Lat	1.000041
Node-00256 - Long	NA
Node-00256 - Lat	NA
Node-00257 - Long	1.000022
Node-00257 - Lat	1.000022
Node-00258 - Long	1.00006
Node-00258 - Lat	1.000029
Node-00259 - Long	NA
Node-00259 - Lat	NA
Node-00260 - Long	NA

Node-00260 - Lat	NA
Node-00261 - Long	NA
Node-00261 - Lat	NA
Node-00262 - Long	0.999996
Node-00262 - Lat	1.000012
Node-00263 - Long	NA
Node-00263 - Lat	NA
Node-00264 - Long	NA
Node-00264 - Lat	NA
Node-00265 - Long	NA
Node-00265 - Lat	NA
Node-00266 - Long	NA
Node-00266 - Lat	NA
Node-00267 - Long	NA
Node-00267 - Lat	NA
Node-00268 - Long	NA
Node-00268 - Lat	NA
Node-00269 - Long	NA
Node-00269 - Lat	NA
Node-00270 - Long	NA
Node-00270 - Lat	NA
Node-00271 - Long	1.000021
Node-00271 - Lat	0.999986
Node-00272 - Long	NA
Node-00272 - Lat	NA
Node-00273 - Long	NA
Node-00273 - Lat	NA
Node-00274 - Long	1.000019
Node-00274 - Lat	1.000023
Node-00275 - Long	0.999987
Node-00275 - Lat	1.000002
Node-00276 - Long	1.000026
Node-00276 - Lat	1.000037
Node-00277 - Long	0.99999
Node-00277 - Lat	1.000021
Node-00278 - Long	1.000017
Node-00278 - Lat	0.999999
Node-00279 - Long	1.000054
Node-00279 - Lat	1.000044
Node-00280 - Long	NA
Node-00280 - Lat	NA
Node-00281 - Long	0.999993
Node-00281 - Lat	0.999994
Node-00282 - Long	0.999993
Node-00282 - Lat	NA
Node-00283 - Long	1.000032
Node-00283 - Lat	0.999991
Node-00284 - Long	NA

Node-00284 - Lat	NA
Node-00285 - Long	1.000039
Node-00285 - Lat	1.000032
Node-00286 - Long	0.999993
Node-00286 - Lat	0.999996
Node-00287 - Long	NA
Node-00287 - Lat	NA
Node-00288 - Long	0.999984
Node-00288 - Lat	1.000026
Node-00289 - Long	1.000032
Node-00289 - Lat	1.000062
Node-00290 - Long	1.000077
Node-00290 - Lat	1.000088
Node-00291 - Long	0.999999
Node-00291 - Lat	1.000025
Node-00292 - Long	NA
Node-00292 - Lat	NA
Node-00293 - Long	1
Node-00293 - Lat	1.000032
Node-00294 - Long	NA
Node-00294 - Lat	NA
Node-00295 - Long	1.000018
Node-00295 - Lat	1.000034
Node-00296 - Long	1.000045
Node-00296 - Lat	0.999986
Node-00297 - Long	0.999995
Node-00297 - Lat	0.999995
Node-00298 - Long	0.999998
Node-00298 - Lat	1.000018
Node-00299 - Long	NA
Node-00299 - Lat	NA
Node-00300 - Long	1.000026
Node-00300 - Lat	1.000016
Node-00301 - Long	1.000044
Node-00301 - Lat	1.000025
Node-00302 - Long	NA
Node-00302 - Lat	NA
Node-00303 - Long	1.000076
Node-00303 - Lat	0.999985
Node-00304 - Long	NA
Node-00304 - Lat	NA
Node-00305 - Long	NA
Node-00305 - Lat	NA
Node-00306 - Long	NA
Node-00306 - Lat	NA
Node-00307 - Long	NA
Node-00307 - Lat	NA
Node-00308 - Long	1.000064

Node-00308 - Lat	1.000041
Node-00309 - Long	1.000044
Node-00309 - Lat	1.000044
Node-00310 - Long	NA
Node-00310 - Lat	NA
Node-00311 - Long	1.000028
Node-00311 - Lat	1.000016
Node-00312 - Long	1.000009
Node-00312 - Lat	1.000051
Node-00313 - Long	1.00004
Node-00313 - Lat	1
Node-00314 - Long	1.000068
Node-00314 - Lat	1.000052
Node-00315 - Long	1.000046
Node-00315 - Lat	0.999994
Node-00316 - Long	1.000581
Node-00316 - Lat	1.000612
Node-00317 - Long	NA
Node-00317 - Lat	NA
Node-00318 - Long	NA
Node-00318 - Lat	NA
Node-00319 - Long	NA
Node-00319 - Lat	NA
Node-00320 - Long	1.000005
Node-00320 - Lat	1.000013
Node-00321 - Long	NA
Node-00321 - Lat	NA
Node-00322 - Long	NA
Node-00322 - Lat	NA
Node-00323 - Long	NA
Node-00323 - Lat	NA
Node-00324 - Long	0.999994
Node-00324 - Lat	1.000043
Node-00325 - Long	NA
Node-00325 - Lat	NA
Node-00326 - Long	NA
Node-00326 - Lat	NA
Node-00327 - Long	1.000043
Node-00327 - Lat	1.00001
Node-00328 - Long	1.000014
Node-00328 - Lat	1.000012
Node-00329 - Long	1.00004
Node-00329 - Lat	1.000019
Node-00330 - Long	NA
Node-00330 - Lat	NA
Node-00331 - Long	NA
Node-00331 - Lat	NA
Node-00332 - Long	NA

Node-00332 - Lat	NA
Node-00333 - Long	NA
Node-00333 - Lat	NA
Node-00334 - Long	NA
Node-00334 - Lat	NA
Node-00335 - Long	1.00007
Node-00335 - Lat	1.000023
Node-00336 - Long	NA
Node-00336 - Lat	NA
Node-00337 - Long	1.000094
Node-00337 - Lat	1.000128
Node-00338 - Long	NA
Node-00338 - Lat	NA
Node-00339 - Long	NA
Node-00339 - Lat	NA
Node-00340 - Long	1.000008
Node-00340 - Lat	0.999966
Node-00341 - Long	1.000006
Node-00341 - Lat	0.999971
Node-00342 - Long	1.000023
Node-00342 - Lat	1.000007
Node-00343 - Long	1.000047
Node-00343 - Lat	1.000119
Node-00344 - Long	1.000089
Node-00344 - Lat	0.999986
Node-00345 - Long	1.000015
Node-00345 - Lat	1.000012
Node-00346 - Long	1.000007
Node-00346 - Lat	0.999994
Node-00347 - Long	0.999972
Node-00347 - Lat	0.999994
Node-00348 - Long	1.000003
Node-00348 - Lat	0.999977
Node-00349 - Long	0.999984
Node-00349 - Lat	0.999991
Node-00350 - Long	0.999969
Node-00350 - Lat	0.999991
Node-00351 - Long	1.000046
Node-00351 - Lat	1.000055
Node-00352 - Long	1.000039
Node-00352 - Lat	1.000018
Node-00353 - Long	0.999988
Node-00353 - Lat	1.000043
Node-00354 - Long	NA
Node-00354 - Lat	NA
Node-00355 - Long	1.000041
Node-00355 - Lat	1.000022
Node-00356 - Long	0.999996

Node-00356 - Lat	1.000019
Node-00357 - Long	0.999999
Node-00357 - Lat	1.000013
Node-00358 - Long	1.000013
Node-00358 - Lat	1.000013
Node-00359 - Long	NA
Node-00359 - Lat	NA
Node-00360 - Long	0.999981
Node-00360 - Lat	0.999981
Node-00361 - Long	NA
Node-00361 - Lat	NA
Node-00362 - Long	1.00001
Node-00362 - Lat	0.999971
Node-00363 - Long	1.000058
Node-00363 - Lat	1.000012
Node-00364 - Long	NA
Node-00364 - Lat	NA
Node-00365 - Long	0.999985
Node-00365 - Lat	0.999985
Node-00366 - Long	1.000036
Node-00366 - Lat	1.000008
Node-00367 - Long	NA
Node-00367 - Lat	NA
Node-00368 - Long	0.999993
Node-00368 - Lat	1.000039
Node-00369 - Long	NA
Node-00369 - Lat	NA
Node-00370 - Long	1.000239
Node-00370 - Lat	1.000083
Node-00371 - Long	NA
Node-00371 - Lat	NA
Node-00372 - Long	1.000008
Node-00372 - Lat	1.000012
Node-00373 - Long	1.00005
Node-00373 - Lat	1.000052
Node-00374 - Long	1.000025
Node-00374 - Lat	1.000055
Node-00375 - Long	0.999977
Node-00375 - Lat	0.999978
Node-00376 - Long	0.999986
Node-00376 - Lat	0.999994
Node-00377 - Long	1.00005
Node-00377 - Lat	1.000008
Node-00378 - Long	NA
Node-00378 - Lat	NA
Node-00379 - Long	0.99999
Node-00379 - Lat	1.000006
Node-00380 - Long	1.000016

Node-00380 - Lat	0.999974
Node-00381 - Long	1.000014
Node-00381 - Lat	0.999974
Node-00382 - Long	NA
Node-00382 - Lat	NA
Node-00383 - Long	1.000086
Node-00383 - Lat	1.000023
Node-00384 - Long	NA
Node-00384 - Lat	NA
Node-00385 - Long	0.999987
Node-00385 - Lat	0.999978
Node-00386 - Long	1.000029
Node-00386 - Lat	1.000012
Node-00387 - Long	1.001338
Node-00387 - Lat	1.002032
Node-00388 - Long	1.000016
Node-00388 - Lat	1.000068
Node-00389 - Long	1.00001
Node-00389 - Lat	0.999989
Node-00390 - Long	1.000068
Node-00390 - Lat	1.000057
Node-00391 - Long	NA
Node-00391 - Lat	NA
Node-00392 - Long	1.000012
Node-00392 - Lat	1.000026
Node-00393 - Long	1.000016
Node-00393 - Lat	0.999982
Node-00394 - Long	0.999999
Node-00394 - Lat	1.000002
Node-00395 - Long	1.000044
Node-00395 - Lat	1.000009
Node-00396 - Long	1.000049
Node-00396 - Lat	0.999999
Node-00397 - Long	NA
Node-00397 - Lat	NA
Node-00398 - Long	1.000042
Node-00398 - Lat	0.999973
Node-00399 - Long	NA
Node-00399 - Lat	NA
Node-00400 - Long	NA
Node-00400 - Lat	NA
Node-00401 - Long	NA
Node-00401 - Lat	NA
Node-00402 - Long	NA
Node-00402 - Lat	NA
Node-00403 - Long	0.999985
Node-00403 - Lat	1.000041
Node-00404 - Long	1.000012

Node-00404 - Lat	1.000016
Node-00405 - Long	1.000014
Node-00405 - Lat	0.999986
Node-00406 - Long	0.999991
Node-00406 - Lat	1.00001
Node-00407 - Long	NA
Node-00407 - Lat	NA
Node-00408 - Long	NA
Node-00408 - Lat	NA
Node-00409 - Long	NA
Node-00409 - Lat	NA
Node-00410 - Long	0.999997
Node-00410 - Lat	1.000013
Node-00411 - Long	NA
Node-00411 - Lat	NA
Node-00412 - Long	NA
Node-00412 - Lat	NA
Node-00413 - Long	NA
Node-00413 - Lat	NA
Node-00414 - Long	1.000042
Node-00414 - Lat	0.999986
Node-00415 - Long	1.000038
Node-00415 - Lat	1.000005
Node-00416 - Long	1.000032
Node-00416 - Lat	0.999991
Node-00417 - Long	0.99999
Node-00417 - Lat	1.000012
Node-00418 - Long	0.999974
Node-00418 - Lat	1.000002
Node-00419 - Long	1.000016
Node-00419 - Lat	1.000022
Node-00420 - Long	0.999973
Node-00420 - Lat	1.000051
Node-00421 - Long	0.999993
Node-00421 - Lat	1.000078
Node-00422 - Long	0.999993
Node-00422 - Lat	1.000071
Node-00423 - Long	1.000024
Node-00423 - Lat	1.000002
Node-00424 - Long	1.000061
Node-00424 - Lat	1
Node-00425 - Long	NA
Node-00425 - Lat	NA
Node-00426 - Long	1.000001
Node-00426 - Lat	0.999992
Node-00427 - Long	NA
Node-00427 - Lat	NA
Node-00428 - Long	NA

Node-00428 - Lat	NA
Node-00429 - Long	1.000087
Node-00429 - Lat	0.999983
Node-00430 - Long	1
Node-00430 - Lat	0.999985
Node-00431 - Long	1.000004
Node-00431 - Lat	1.000029
Node-00432 - Long	1.000023
Node-00432 - Lat	1.000008
Node-00433 - Long	NA
Node-00433 - Lat	NA
Node-00434 - Long	NA
Node-00434 - Lat	NA
Node-00435 - Long	NA
Node-00435 - Lat	NA
Node-00436 - Long	1.000043
Node-00436 - Lat	0.999989
Node-00437 - Long	1.000025
Node-00437 - Lat	1.000016
Node-00438 - Long	1.000027
Node-00438 - Lat	0.999992
Node-00439 - Long	1.000007
Node-00439 - Lat	0.999986
Node-00440 - Long	0.999997
Node-00440 - Lat	0.999981
Node-00441 - Long	0.999977
Node-00441 - Lat	1.000009
Node-00442 - Long	1.000068
Node-00442 - Lat	1.000005
Node-00443 - Long	1.00005
Node-00443 - Lat	0.999987
Node-00444 - Long	0.999992
Node-00444 - Lat	0.999972
Node-00445 - Long	1.000024
Node-00445 - Lat	1.000012
Node-00446 - Long	1.000014
Node-00446 - Lat	1.000039
Node-00447 - Long	1.000011
Node-00447 - Lat	1.000009
Node-00448 - Long	1.000022
Node-00448 - Lat	1.000027
Node-00449 - Long	1.000053
Node-00449 - Lat	1.000012
Node-00450 - Long	NA
Node-00450 - Lat	NA
Node-00451 - Long	NA
Node-00451 - Lat	NA
Node-00452 - Long	0.999977

Node-00452 - Lat	1.000062
Node-00453 - Long	0.999996
Node-00453 - Lat	0.999982
Node-00454 - Long	NA
Node-00454 - Lat	NA
Node-00455 - Long	1.000027
Node-00455 - Lat	1.000001
Node-00456 - Long	NA
Node-00456 - Lat	NA
Node-00457 - Long	0.999984
Node-00457 - Lat	1
Node-00458 - Long	1.000017
Node-00458 - Lat	0.999967
Node-00459 - Long	NA
Node-00459 - Lat	NA
Node-00460 - Long	1.000017
Node-00460 - Lat	1.000054
Node-00461 - Long	0.999991
Node-00461 - Lat	0.999991
Node-00462 - Long	NA
Node-00462 - Lat	NA
Node-00463 - Long	1.000014
Node-00463 - Lat	0.999989
Node-00464 - Long	NA
Node-00464 - Lat	NA
Node-00465 - Long	0.999995
Node-00465 - Lat	1.000036
Node-00466 - Long	0.999992
Node-00466 - Lat	1.000022
Node-00467 - Long	NA
Node-00467 - Lat	NA
Node-00468 - Long	0.999995
Node-00468 - Lat	0.999982
Node-00469 - Long	1.000003
Node-00469 - Lat	1.000022
Node-00470 - Long	NA
Node-00470 - Lat	NA
Node-00471 - Long	0.999969
Node-00471 - Lat	0.999969
Node-00472 - Long	NA
Node-00472 - Lat	NA
Node-00473 - Long	NA
Node-00473 - Lat	NA
Node-00474 - Long	0.999991
Node-00474 - Lat	0.999991
Node-00475 - Long	NA
Node-00475 - Lat	NA
Node-00476 - Long	1.000104

Node-00476 - Lat	1.000016
Node-00477 - Long	NA
Node-00477 - Lat	NA
Node-00478 - Long	NA
Node-00478 - Lat	NA
Node-00479 - Long	NA
Node-00479 - Lat	NA
Node-00480 - Long	NA
Node-00480 - Lat	NA
Node-00481 - Long	1.000051
Node-00481 - Lat	1.000006
Node-00482 - Long	NA
Node-00482 - Lat	NA
Node-00483 - Long	1.000009
Node-00483 - Lat	1.000009
Node-00484 - Long	0.999963
Node-00484 - Lat	1.000003
Node-00485 - Long	1.000022
Node-00485 - Lat	0.999991
Node-00486 - Long	NA
Node-00486 - Lat	NA
Node-00487 - Long	NA
Node-00487 - Lat	NA
Node-00488 - Long	1.000045
Node-00488 - Lat	1.000027
Node-00489 - Long	NA
Node-00489 - Lat	NA
Node-00490 - Long	NA
Node-00490 - Lat	NA
Node-00491 - Long	0.999985
Node-00491 - Lat	1.000011
Node-00492 - Long	0.999994
Node-00492 - Lat	1.000038
Node-00493 - Long	1.000081
Node-00493 - Lat	0.999998
Node-00494 - Long	NA
Node-00494 - Lat	NA
Node-00495 - Long	1.000016
Node-00495 - Lat	1.000031
Node-00496 - Long	1.000071
Node-00496 - Lat	0.999986
Node-00497 - Long	NA
Node-00497 - Lat	NA
Node-00498 - Long	1.000026
Node-00498 - Lat	0.999975
Node-00499 - Long	NA
Node-00499 - Lat	NA
Node-00500 - Long	NA

Node-00500 - Lat	NA
Node-00501 - Long	NA
Node-00501 - Lat	NA
Node-00502 - Long	NA
Node-00502 - Lat	NA
Node-00503 - Long	NA
Node-00503 - Lat	NA
Node-00504 - Long	0.999993
Node-00504 - Lat	0.999993
Node-00505 - Long	NA
Node-00505 - Lat	NA
Node-00506 - Long	NA
Node-00506 - Lat	NA
Node-00507 - Long	1.000045
Node-00507 - Lat	1.00017
Node-00508 - Long	NA
Node-00508 - Lat	NA
Node-00509 - Long	NA
Node-00509 - Lat	NA
Node-00510 - Long	0.999991
Node-00510 - Lat	1.00002
Node-00511 - Long	NA
Node-00511 - Lat	NA
Node-00512 - Long	NA
Node-00512 - Lat	NA
Node-00513 - Long	1.000114
Node-00513 - Lat	1.000072
Node-00514 - Long	1.000053
Node-00514 - Lat	1.000097
Node-00515 - Long	0.999993
Node-00515 - Lat	1.00005
Node-00516 - Long	1
Node-00516 - Lat	0.999988
Node-00517 - Long	NA
Node-00517 - Lat	NA
Node-00518 - Long	NA
Node-00518 - Lat	NA
Node-00519 - Long	NA
Node-00519 - Lat	NA
Node-00520 - Long	NA
Node-00520 - Lat	NA
Node-00521 - Long	NA
Node-00521 - Lat	NA
Node-00522 - Long	NA
Node-00522 - Lat	NA
Node-00523 - Long	0.999993
Node-00523 - Lat	0.999983
Node-00524 - Long	0.999987

Node-00524 - Lat	0.999994
Node-00525 - Long	1.000106
Node-00525 - Lat	1.000105
Node-00526 - Long	1.000044
Node-00526 - Lat	1.000017
Node-00527 - Long	NA
Node-00527 - Lat	NA
Node-00528 - Long	0.999972
Node-00528 - Lat	0.999973
Node-00529 - Long	NA
Node-00529 - Lat	NA
Node-00530 - Long	NA
Node-00530 - Lat	NA
Node-00531 - Long	1.000006
Node-00531 - Lat	0.999993
Node-00532 - Long	NA
Node-00532 - Lat	NA
Node-00533 - Long	NA
Node-00533 - Lat	NA
Node-00534 - Long	1.000055
Node-00534 - Lat	1.000026
Node-00535 - Long	NA
Node-00535 - Lat	NA
Node-00536 - Long	1.000083
Node-00536 - Lat	1.000033
Node-00537 - Long	NA
Node-00537 - Lat	NA
Node-00538 - Long	NA
Node-00538 - Lat	NA
Node-00539 - Long	NA
Node-00539 - Lat	NA
Node-00540 - Long	0.999978
Node-00540 - Lat	1.000142
Node-00541 - Long	NA
Node-00541 - Lat	NA
Node-00542 - Long	NA
Node-00542 - Lat	NA
Node-00543 - Long	1.00006
Node-00543 - Lat	1.000004
Node-00544 - Long	1.00009
Node-00544 - Lat	1.000035
Node-00545 - Long	1.000138
Node-00545 - Lat	0.999995
Node-00546 - Long	1.000114
Node-00546 - Lat	1.00005
Node-00547 - Long	1.000058
Node-00547 - Lat	1.000003
Node-00548 - Long	1.000048

Node-00548 - Lat	1.000058
Node-00549 - Long	0.999989
Node-00549 - Lat	1.000051
Node-00550 - Long	0.999967
Node-00550 - Lat	1.000066
Node-00551 - Long	0.999975
Node-00551 - Lat	1.000052
Node-00552 - Long	0.999995
Node-00552 - Lat	1.000015
Node-00553 - Long	1.000001
Node-00553 - Lat	1.000013
Node-00554 - Long	1.000003
Node-00554 - Lat	0.999998
Node-00555 - Long	1.000034
Node-00555 - Lat	0.999966
Node-00556 - Long	1.000018
Node-00556 - Lat	1.000003
Node-00557 - Long	1.000005
Node-00557 - Lat	0.999967
Node-00558 - Long	0.999987
Node-00558 - Lat	1.000011
Node-00559 - Long	NA
Node-00559 - Lat	NA
Node-00560 - Long	0.99997
Node-00560 - Lat	0.99997
Node-00561 - Long	NA
Node-00561 - Lat	NA
Node-00562 - Long	0.999998
Node-00562 - Lat	1.00001
Node-00563 - Long	1.000051
Node-00563 - Lat	0.999977
Node-00564 - Long	1.00001
Node-00564 - Lat	1.000034
Node-00565 - Long	0.999991
Node-00565 - Lat	1.000042
Node-00566 - Long	1.00007
Node-00566 - Lat	1.000079
Node-00567 - Long	1.00001
Node-00567 - Lat	0.999973
Node-00568 - Long	NA
Node-00568 - Lat	NA
Node-00569 - Long	0.999999
Node-00569 - Lat	1.000009
Node-00570 - Long	NA
Node-00570 - Lat	NA
Node-00571 - Long	1.000013
Node-00571 - Lat	1.000055
Node-00572 - Long	NA

Node-00572 - Lat	NA
Node-00573 - Long	NA
Node-00573 - Lat	NA
Node-00574 - Long	1.000016
Node-00574 - Lat	1.000016
Node-00575 - Long	0.99999
Node-00575 - Lat	0.999981
Node-00576 - Long	NA
Node-00576 - Lat	NA
Node-00577 - Long	1.000104
Node-00577 - Lat	0.999994
Node-00578 - Long	NA
Node-00578 - Lat	NA
Node-00579 - Long	NA
Node-00579 - Lat	NA
Node-00580 - Long	0.99999
Node-00580 - Lat	0.99999
Node-00581 - Long	NA
Node-00581 - Lat	NA
Node-00582 - Long	1.000072
Node-00582 - Lat	1.000112
Node-00583 - Long	1.000008
Node-00583 - Lat	1.000077
Node-00584 - Long	NA
Node-00584 - Lat	NA
Node-00585 - Long	1.000021
Node-00585 - Lat	0.999992
Node-00586 - Long	0.999989
Node-00586 - Lat	1.000001
Node-00587 - Long	1.000011
Node-00587 - Lat	0.999984
Node-00588 - Long	1.000087
Node-00588 - Lat	1.000046
Node-00589 - Long	NA
Node-00589 - Lat	NA
Node-00590 - Long	NA
Node-00590 - Lat	NA
Node-00591 - Long	NA
Node-00591 - Lat	NA
Node-00592 - Long	1.000101
Node-00592 - Lat	1.00006
Node-00593 - Long	NA
Node-00593 - Lat	NA
Node-00594 - Long	0.999973
Node-00594 - Lat	1.000044
Node-00595 - Long	1.000038
Node-00595 - Lat	1.000036
Node-00596 - Long	1.000031

Node-00596 - Lat	1.000073
Node-00597 - Long	NA
Node-00597 - Lat	NA
Node-00598 - Long	0.999997
Node-00598 - Lat	1.000099
Node-00599 - Long	1.000029
Node-00599 - Lat	1.000058
Node-00600 - Long	1.000041
Node-00600 - Lat	1.000028
Node-00601 - Long	0.999998
Node-00601 - Lat	1.000065
Node-00602 - Long	NA
Node-00602 - Lat	NA
Node-00603 - Long	NA
Node-00603 - Lat	NA
Node-00604 - Long	NA
Node-00604 - Lat	NA
Node-00605 - Long	NA
Node-00605 - Lat	NA
Node-00606 - Long	NA
Node-00606 - Lat	NA
Node-00607 - Long	1.000121
Node-00607 - Lat	1
Node-00608 - Long	1.000026
Node-00608 - Lat	1.000059
Node-00609 - Long	0.999996
Node-00609 - Lat	0.999997
Node-00610 - Long	NA
Node-00610 - Lat	NA
Node-00611 - Long	1.000063
Node-00611 - Lat	1.000044
Node-00612 - Long	NA
Node-00612 - Lat	NA
Node-00613 - Long	NA
Node-00613 - Lat	NA
Node-00614 - Long	NA
Node-00614 - Lat	NA
Node-00615 - Long	1.000009
Node-00615 - Lat	0.999986
Node-00616 - Long	NA
Node-00616 - Lat	NA
Node-00617 - Long	NA
Node-00617 - Lat	NA
Node-00618 - Long	1.00004
Node-00618 - Lat	0.999971
Node-00619 - Long	1.000056
Node-00619 - Lat	1.000001
Node-00620 - Long	1.000037

Node-00620 - Lat	1.000321
Node-00621 - Long	1.000042
Node-00621 - Lat	1.000059
Node-00622 - Long	NA
Node-00622 - Lat	NA
Node-00623 - Long	NA
Node-00623 - Lat	NA
Node-00624 - Long	1.000044
Node-00624 - Lat	1.000022
Node-00625 - Long	1.000012
Node-00625 - Lat	0.999996
Node-00626 - Long	1.005112
Node-00626 - Lat	1.005179
Node-00627 - Long	1.000098
Node-00627 - Lat	1.000027
Node-00628 - Long	NA
Node-00628 - Lat	NA
Node-00629 - Long	NA
Node-00629 - Lat	NA
Node-00630 - Long	NA
Node-00630 - Lat	NA
Node-00631 - Long	NA
Node-00631 - Lat	NA
Node-00632 - Long	1.000067
Node-00632 - Lat	1.000024
Node-00633 - Long	NA
Node-00633 - Lat	NA
Node-00634 - Long	NA
Node-00634 - Lat	NA
Node-00635 - Long	NA
Node-00635 - Lat	NA
Node-00636 - Long	NA
Node-00636 - Lat	NA
Node-00637 - Long	1.000087
Node-00637 - Lat	1.000083
Node-00638 - Long	1.000025
Node-00638 - Lat	1.000025
Node-00639 - Long	1.000074
Node-00639 - Lat	1.000011
Node-00640 - Long	NA
Node-00640 - Lat	NA
Node-00641 - Long	1.000127
Node-00641 - Lat	1.000128
Node-00642 - Long	1.000003
Node-00642 - Lat	0.999965
Node-00643 - Long	1.00011
Node-00643 - Lat	1.00009
Node-00644 - Long	0.999975

Node-00644 - Lat	0.999979
Node-00645 - Long	NA
Node-00645 - Lat	NA
Node-00646 - Long	NA
Node-00646 - Lat	NA
Node-00647 - Long	NA
Node-00647 - Lat	NA
Node-00648 - Long	0.99999
Node-00648 - Lat	0.99999
Node-00649 - Long	NA
Node-00649 - Lat	NA
Node-00650 - Long	0.999991
Node-00650 - Lat	1.000042
Node-00651 - Long	NA
Node-00651 - Lat	NA
Node-00652 - Long	0.99997
Node-00652 - Lat	0.999971
Node-00653 - Long	NA
Node-00653 - Lat	NA
Node-00654 - Long	1.000065
Node-00654 - Lat	1.000027
Node-00655 - Long	1.000003
Node-00655 - Lat	1.000011
Node-00656 - Long	1.000007
Node-00656 - Lat	1.000037
Node-00657 - Long	0.999993
Node-00657 - Lat	0.99999
Node-00658 - Long	1.00003
Node-00658 - Lat	1.000012
Node-00659 - Long	1.000032
Node-00659 - Lat	0.999992
Node-00660 - Long	1.000036
Node-00660 - Lat	0.999976
Node-00661 - Long	NA
Node-00661 - Lat	NA
Node-00662 - Long	1.00005
Node-00662 - Lat	0.999997
Node-00663 - Long	1.000127
Node-00663 - Lat	1.000005
Node-00664 - Long	NA
Node-00664 - Lat	NA
Node-00665 - Long	NA
Node-00665 - Lat	NA
Node-00666 - Long	NA
Node-00666 - Lat	NA
Node-00667 - Long	NA
Node-00667 - Lat	NA
Node-00668 - Long	0.999972

Node-00668 - Lat	1.000002
Node-00669 - Long	0.999993
Node-00669 - Lat	0.99998
Node-00670 - Long	NA
Node-00670 - Lat	NA
Node-00671 - Long	NA
Node-00671 - Lat	NA
Node-00672 - Long	0.999976
Node-00672 - Lat	0.999998
Node-00673 - Long	NA
Node-00673 - Lat	NA
Node-00674 - Long	1.000003
Node-00674 - Lat	1.000034
Node-00675 - Long	0.999991
Node-00675 - Lat	0.999985
Node-00676 - Long	1.000026
Node-00676 - Lat	1.000025
Node-00677 - Long	0.999979
Node-00677 - Lat	0.999972
Node-00678 - Long	0.999964
Node-00678 - Lat	1.000132
Node-00679 - Long	1.000013
Node-00679 - Lat	1.000013
Node-00680 - Long	NA
Node-00680 - Lat	NA
Node-00681 - Long	1.00003
Node-00681 - Lat	0.999993
Node-00682 - Long	NA
Node-00682 - Lat	NA
Node-00683 - Long	NA
Node-00683 - Lat	NA
Node-00684 - Long	NA
Node-00684 - Lat	NA
Node-00685 - Long	NA
Node-00685 - Lat	NA
Node-00686 - Long	NA
Node-00686 - Lat	NA
Node-00687 - Long	NA
Node-00687 - Lat	NA
Node-00688 - Long	NA
Node-00688 - Lat	NA
Node-00689 - Long	1.000042
Node-00689 - Lat	1.000003
Node-00690 - Long	NA
Node-00690 - Lat	NA
Node-00691 - Long	NA
Node-00691 - Lat	NA
Node-00692 - Long	1.000028

Node-00692 - Lat	1.000052
Node-00693 - Long	1.00007
Node-00693 - Lat	1.000136
Node-00694 - Long	NA
Node-00694 - Lat	NA
Node-00695 - Long	1.000064
Node-00695 - Lat	1.000127
Node-00696 - Long	1.000038
Node-00696 - Lat	1.000038
Node-00697 - Long	1.000062
Node-00697 - Lat	1.000029
Node-00698 - Long	1.00004
Node-00698 - Lat	0.999982
Node-00699 - Long	0.999971
Node-00699 - Lat	1.000029
Node-00700 - Long	NA
Node-00700 - Lat	NA
Node-00701 - Long	NA
Node-00701 - Lat	NA
Node-00702 - Long	1.000026
Node-00702 - Lat	1.0001
Node-00703 - Long	0.999997
Node-00703 - Lat	0.999997
Node-00704 - Long	1.000471
Node-00704 - Lat	1.000471
Node-00705 - Long	NA
Node-00705 - Lat	NA
Node-00706 - Long	NA
Node-00706 - Lat	NA
Node-00707 - Long	1.000012
Node-00707 - Lat	0.999971
Node-00708 - Long	1.000045
Node-00708 - Lat	1.000016
Node-00709 - Long	1.000094
Node-00709 - Lat	1.000008
Node-00710 - Long	1.000029
Node-00710 - Lat	1.000032
Node-00711 - Long	1.000025
Node-00711 - Lat	1.00005
Node-00712 - Long	1.000058
Node-00712 - Lat	1.000039
Node-00713 - Long	0.99998
Node-00713 - Lat	0.99999
Node-00714 - Long	1.000061
Node-00714 - Lat	1.000019
Node-00715 - Long	1.000009
Node-00715 - Lat	1.000011
Node-00716 - Long	0.999993

Node-00716 - Lat	1.000069
Node-00717 - Long	0.999987
Node-00717 - Lat	0.999995
Node-00718 - Long	0.999976
Node-00718 - Lat	0.999993
Node-00719 - Long	NA
Node-00719 - Lat	NA
Node-00720 - Long	1.000101
Node-00720 - Lat	1.000004
Node-00721 - Long	1.000008
Node-00721 - Lat	1.000008
Node-00722 - Long	NA
Node-00722 - Lat	NA
Node-00723 - Long	0.999991
Node-00723 - Lat	1.000019
Node-00724 - Long	1.000153
Node-00724 - Lat	1.000153
Node-00725 - Long	NA
Node-00725 - Lat	NA
Node-00726 - Long	1.000023
Node-00726 - Lat	0.999999
Node-00727 - Long	1.000038
Node-00727 - Lat	1.000061
Node-00728 - Long	NA
Node-00728 - Lat	NA
Node-00729 - Long	NA
Node-00729 - Lat	NA
Node-00730 - Long	1.000026
Node-00730 - Lat	1.000091
Node-00731 - Long	NA
Node-00731 - Lat	NA
Node-00732 - Long	NA
Node-00732 - Lat	NA
Node-00733 - Long	1.000186
Node-00733 - Lat	0.999984
Node-00734 - Long	NA
Node-00734 - Lat	NA
Node-00735 - Long	0.999978
Node-00735 - Lat	1.000001
Node-00736 - Long	NA
Node-00736 - Lat	NA
Node-00737 - Long	0.99998
Node-00737 - Lat	1.000015
Node-00738 - Long	NA
Node-00738 - Lat	NA
Node-00739 - Long	NA
Node-00739 - Lat	NA
Node-00740 - Long	NA

Node-00740 - Lat	NA
Node-00741 - Long	0.999988
Node-00741 - Lat	1.000001
Node-00742 - Long	0.999979
Node-00742 - Lat	1.000038
Node-00743 - Long	NA
Node-00743 - Lat	NA
Node-00744 - Long	1.000008
Node-00744 - Lat	1.000004
Node-00745 - Long	NA
Node-00745 - Lat	NA
Node-00746 - Long	1.000048
Node-00746 - Lat	1.000046
Node-00747 - Long	1.000003
Node-00747 - Lat	1.000084
Node-00748 - Long	NA
Node-00748 - Lat	NA
Node-00749 - Long	1.000007
Node-00749 - Lat	1.000011
Node-00750 - Long	NA
Node-00750 - Lat	NA
Node-00751 - Long	1.000016
Node-00751 - Lat	1.000009
Node-00752 - Long	NA
Node-00752 - Lat	NA
Node-00753 - Long	0.999992
Node-00753 - Lat	1.000006
Node-00754 - Long	1.000025
Node-00754 - Lat	1.000025
Node-00755 - Long	1.000029
Node-00755 - Lat	1.000004

Table S3: Potential scale reduction factors of ancestral state estimates across our alternative topologies under variable rates

trad_bm param	trad_bm psrf	lag_bm param	lag_bm psrf	sil_bm param	sil_bm psrf
Lh	3.39867	Lh	2.825656	Lh	2.825656
Scale	1.023488	Scale	1.00939	Scale	1.00939
Node-00000 - Long	1.000762	Node-00000 - Long	1.000487	Node-00000 - Long	1.000487
Node-00000 - Lat	1.001379	Node-00000 - Lat	1.000443	Node-00000 - Lat	1.000443
Node-00001 - Long	1.001159	Node-00001 - Long	1.000944	Node-00001 - Long	1.000944
Node-00001 - Lat	1.002549	Node-00001 - Lat	1.000918	Node-00001 - Lat	1.000918
Node-00002 - Long	1.001841	Node-00002 - Long	1.001117	Node-00002 - Long	1.001117
Node-00002 - Lat	1.004512	Node-00002 - Lat	1.001581	Node-00002 - Lat	1.001581
Node-00003 - Long	1.003262	Node-00003 - Long	1.002315	Node-00003 - Long	1.002315
Node-00003 - Lat	1.103352	Node-00003 - Lat	1.07111	Node-00003 - Lat	1.07111
Node-00004 - Long	1.004491	Node-00004 - Long	1.002905	Node-00004 - Long	1.002905
Node-00004 - Lat	1.121793	Node-00004 - Lat	1.07678	Node-00004 - Lat	1.07678
Node-00005 - Long	1.006446	Node-00005 - Long	1.004278	Node-00005 - Long	1.004278
Node-00005 - Lat	1.132737	Node-00005 - Lat	1.088425	Node-00005 - Lat	1.088425
Node-00006 - Long	1.005229	Node-00006 - Long	1.003497	Node-00006 - Long	1.003497
Node-00006 - Lat	1.13205	Node-00006 - Lat	1.082359	Node-00006 - Lat	1.082359
Node-00007 - Long	1.006299	Node-00007 - Long	1.00389	Node-00007 - Long	1.00389
Node-00007 - Lat	1.137509	Node-00007 - Lat	1.088614	Node-00007 - Lat	1.088614
Node-00008 - Long	1.010206	Node-00008 - Long	1.004446	Node-00008 - Long	1.004446
Node-00008 - Lat	1.150417	Node-00008 - Lat	1.109626	Node-00008 - Lat	1.109626
Node-00009 - Long	1.006996	Node-00009 - Long	1.008125	Node-00009 - Long	1.008125
Node-00009 - Lat	1.014442	Node-00009 - Lat	1.008499	Node-00009 - Lat	1.008499
Node-00010 - Long	1.026109	Node-00010 - Long	1.029972	Node-00010 - Long	1.029972
Node-00010 - Lat	1.035461	Node-00010 - Lat	1.008024	Node-00010 - Lat	1.008024
Node-00011 - Long	1.049054	Node-00011 - Long	1.058739	Node-00011 - Long	1.058739
Node-00011 - Lat	1.060056	Node-00011 - Lat	1.010852	Node-00011 - Lat	1.010852
Node-00012 - Long	1.078478	Node-00012 - Long	1.093742	Node-00012 - Long	1.093742
Node-00012 - Lat	1.062759	Node-00012 - Lat	1.015724	Node-00012 - Lat	1.015724
Node-00013 - Long	1.101023	Node-00013 - Long	1.124017	Node-00013 - Long	1.124017
Node-00013 - Lat	1.094281	Node-00013 - Lat	1.02536	Node-00013 - Lat	1.02536
Node-00014 - Long	1.130605	Node-00014 - Long	1.176184	Node-00014 - Long	1.176184
Node-00014 - Lat	1.153125	Node-00014 - Lat	1.067372	Node-00014 - Lat	1.067372
Node-00015 - Long	2.921573	Node-00015 - Long	2.342916	Node-00015 - Long	2.342916
Node-00015 - Lat	8.467172	Node-00015 - Lat	3.322843	Node-00015 - Lat	3.322843
Node-00016 - Long	2.898657	Node-00016 - Long	2.311229	Node-00016 - Long	2.311229
Node-00016 - Lat	8.878744	Node-00016 - Lat	3.357545	Node-00016 - Lat	3.357545
Node-00017 - Long	3.10401	Node-00017 - Long	2.303697	Node-00017 - Long	2.303697
Node-00017 - Lat	9.014218	Node-00017 - Lat	3.33919	Node-00017 - Lat	3.33919
Node-00018 - Long	1.005128	Node-00018 - Long	1.020148	Node-00018 - Long	1.020148
Node-00018 - Lat	1.003292	Node-00018 - Lat	1.021575	Node-00018 - Lat	1.021575
Node-00019 - Long	1.003564	Node-00019 - Long	1.013983	Node-00019 - Long	1.013983
Node-00019 - Lat	1.004266	Node-00019 - Lat	1.031088	Node-00019 - Lat	1.031088
Node-00020 - Long	1.002101	Node-00020 - Long	1.01165	Node-00020 - Long	1.01165

Node-00020 - Lat	1.002476	Node-00020 - Lat	1.00296	Node-00020 - Lat	1.00296
Node-00021 - Long	1.001555	Node-00021 - Long	1.012771	Node-00021 - Long	1.012771
Node-00021 - Lat	1.001724	Node-00021 - Lat	1.001911	Node-00021 - Lat	1.001911
Node-00022 - Long	1.001509	Node-00022 - Long	1.015371	Node-00022 - Long	1.015371
Node-00022 - Lat	1.001286	Node-00022 - Lat	1.000867	Node-00022 - Lat	1.000867
Node-00023 - Long	1.002092	Node-00023 - Long	1.008309	Node-00023 - Long	1.008309
Node-00023 - Lat	1.000977	Node-00023 - Lat	1.000797	Node-00023 - Lat	1.000797
Node-00024 - Long	1.002832	Node-00024 - Long	1.004136	Node-00024 - Long	1.004136
Node-00024 - Lat	1.001047	Node-00024 - Lat	1.000854	Node-00024 - Lat	1.000854
Node-00025 - Long	1.004285	Node-00025 - Long	1.007306	Node-00025 - Long	1.007306
Node-00025 - Lat	1.00136	Node-00025 - Lat	1.00259	Node-00025 - Lat	1.00259
Node-00026 - Long	1.009408	Node-00026 - Long	1.014133	Node-00026 - Long	1.014133
Node-00026 - Lat	1.003591	Node-00026 - Lat	1.005769	Node-00026 - Lat	1.005769
Node-00027 - Long	1.016123	Node-00027 - Long	1.025283	Node-00027 - Long	1.025283
Node-00027 - Lat	1.006491	Node-00027 - Lat	1.010597	Node-00027 - Lat	1.010597
Node-00028 - Long	1.027029	Node-00028 - Long	1.039936	Node-00028 - Long	1.039936
Node-00028 - Lat	1.011106	Node-00028 - Lat	1.016391	Node-00028 - Lat	1.016391
Node-00029 - Long	1.038176	Node-00029 - Long	1.154112	Node-00029 - Long	1.154112
Node-00029 - Lat	1.015875	Node-00029 - Lat	1.278634	Node-00029 - Lat	1.278634
Node-00030 - Long	1.080004	Node-00030 - Long	2.502164	Node-00030 - Long	2.502164
Node-00030 - Lat	1.139524	Node-00030 - Lat	17.08226	Node-00030 - Lat	17.08226
Node-00031 - Long	2.567674	Node-00031 - Long	3.120902	Node-00031 - Long	3.120902
Node-00031 - Lat	16.109	Node-00031 - Lat	22.15213	Node-00031 - Lat	22.15213
Node-00032 - Long	4.472983	Node-00032 - Long	1.531765	Node-00032 - Long	1.531765
Node-00032 - Lat	31.93824	Node-00032 - Lat	18.53923	Node-00032 - Lat	18.53923
Node-00033 - Long	1.39657	Node-00033 - Long	1.058352	Node-00033 - Long	1.058352
Node-00033 - Lat	17.85614	Node-00033 - Lat	19.30794	Node-00033 - Lat	19.30794
Node-00034 - Long	1.030921	Node-00034 - Long	1.700391	Node-00034 - Long	1.700391
Node-00034 - Lat	18.52353	Node-00034 - Lat	28.23104	Node-00034 - Lat	28.23104
Node-00035 - Long	2.305548	Node-00035 - Long	1.035907	Node-00035 - Long	1.035907
Node-00035 - Lat	40.11986	Node-00035 - Lat	1.072316	Node-00035 - Lat	1.072316
Node-00036 - Long	1.027189	Node-00036 - Long	1.028237	Node-00036 - Long	1.028237
Node-00036 - Lat	1.038497	Node-00036 - Lat	1.045638	Node-00036 - Lat	1.045638
Node-00037 - Long	1.024483	Node-00037 - Long	1.02316	Node-00037 - Long	1.02316
Node-00037 - Lat	1.027335	Node-00037 - Lat	1.035334	Node-00037 - Lat	1.035334
Node-00038 - Long	1.020845	Node-00038 - Long	1.023338	Node-00038 - Long	1.023338
Node-00038 - Lat	1.023476	Node-00038 - Lat	1.030511	Node-00038 - Lat	1.030511
Node-00039 - Long	1.020047	Node-00039 - Long	1.016668	Node-00039 - Long	1.016668
Node-00039 - Lat	1.021299	Node-00039 - Lat	1.026495	Node-00039 - Lat	1.026495
Node-00040 - Long	1.012877	Node-00040 - Long	1.012241	Node-00040 - Long	1.012241
Node-00040 - Lat	1.018984	Node-00040 - Lat	1.015915	Node-00040 - Lat	1.015915
Node-00041 - Long	1.008998	Node-00041 - Long	1.009045	Node-00041 - Long	1.009045
Node-00041 - Lat	1.01279	Node-00041 - Lat	1.010182	Node-00041 - Lat	1.010182
Node-00042 - Long	1.006404	Node-00042 - Long	1.006567	Node-00042 - Long	1.006567
Node-00042 - Lat	1.007869	Node-00042 - Lat	1.006613	Node-00042 - Lat	1.006613
Node-00043 - Long	1.005144	Node-00043 - Long	1.004745	Node-00043 - Long	1.004745
Node-00043 - Lat	1.005312	Node-00043 - Lat	1.005772	Node-00043 - Lat	1.005772
Node-00044 - Long	1.003688	Node-00044 - Long	1.003479	Node-00044 - Long	1.003479

Node-00044 - Lat	1.004571	Node-00044 - Lat	1.003065	Node-00044 - Lat	1.003065
Node-00045 - Long	1.003212	Node-00045 - Long	1.002158	Node-00045 - Long	1.002158
Node-00045 - Lat	1.002747	Node-00045 - Lat	1.001614	Node-00045 - Lat	1.001614
Node-00046 - Long	1.001646	Node-00046 - Long	1.000672	Node-00046 - Long	1.000672
Node-00046 - Lat	1.001882	Node-00046 - Lat	1.000269	Node-00046 - Lat	1.000269
Node-00047 - Long	1.000343	Node-00047 - Long	1.000134	Node-00047 - Long	1.000134
Node-00047 - Lat	1.00056	Node-00047 - Lat	1.000155	Node-00047 - Lat	1.000155
Node-00048 - Long	1.000173	Node-00048 - Long	NA	Node-00048 - Long	NA
Node-00048 - Lat	1.000129	Node-00048 - Lat	NA	Node-00048 - Lat	NA
Node-00049 - Long	NA	Node-00049 - Long	NA	Node-00049 - Long	NA
Node-00049 - Lat	NA	Node-00049 - Lat	NA	Node-00049 - Lat	NA
Node-00050 - Long	NA	Node-00050 - Long	NA	Node-00050 - Long	NA
Node-00050 - Lat	NA	Node-00050 - Lat	NA	Node-00050 - Lat	NA
Node-00051 - Long	NA	Node-00051 - Long	1.001019	Node-00051 - Long	1.001019
Node-00051 - Lat	NA	Node-00051 - Lat	1.001134	Node-00051 - Lat	1.001134
Node-00052 - Long	1.000703	Node-00052 - Long	1.000562	Node-00052 - Long	1.000562
Node-00052 - Lat	1.000927	Node-00052 - Lat	1.000877	Node-00052 - Lat	1.000877
Node-00053 - Long	1.000395	Node-00053 - Long	1.000269	Node-00053 - Long	1.000269
Node-00053 - Lat	1.000518	Node-00053 - Lat	1.000606	Node-00053 - Lat	1.000606
Node-00054 - Long	1.000155	Node-00054 - Long	1.000098	Node-00054 - Long	1.000098
Node-00054 - Lat	1.000256	Node-00054 - Lat	1.000288	Node-00054 - Lat	1.000288
Node-00055 - Long	1.000206	Node-00055 - Long	1.000772	Node-00055 - Long	1.000772
Node-00055 - Lat	1.000459	Node-00055 - Lat	1.00195	Node-00055 - Lat	1.00195
Node-00056 - Long	1.002975	Node-00056 - Long	1.0004	Node-00056 - Long	1.0004
Node-00056 - Lat	1.003689	Node-00056 - Lat	1.001658	Node-00056 - Lat	1.001658
Node-00057 - Long	1.001774	Node-00057 - Long	1.000217	Node-00057 - Long	1.000217
Node-00057 - Lat	1.002396	Node-00057 - Lat	1.00027	Node-00057 - Lat	1.00027
Node-00058 - Long	1.000051	Node-00058 - Long	NA	Node-00058 - Long	NA
Node-00058 - Lat	1.000116	Node-00058 - Lat	NA	Node-00058 - Lat	NA
Node-00059 - Long	NA	Node-00059 - Long	NA	Node-00059 - Long	NA
Node-00059 - Lat	NA	Node-00059 - Lat	NA	Node-00059 - Lat	NA
Node-00060 - Long	NA	Node-00060 - Long	NA	Node-00060 - Long	NA
Node-00060 - Lat	NA	Node-00060 - Lat	NA	Node-00060 - Lat	NA
Node-00061 - Long	NA	Node-00061 - Long	1.001467	Node-00061 - Long	1.001467
Node-00061 - Lat	NA	Node-00061 - Lat	1.003066	Node-00061 - Lat	1.003066
Node-00062 - Long	1.001385	Node-00062 - Long	1.001466	Node-00062 - Long	1.001466
Node-00062 - Lat	1.002661	Node-00062 - Lat	1.002189	Node-00062 - Lat	1.002189
Node-00063 - Long	1.000995	Node-00063 - Long	1.001506	Node-00063 - Long	1.001506
Node-00063 - Lat	1.002087	Node-00063 - Lat	1.002401	Node-00063 - Lat	1.002401
Node-00064 - Long	1.000652	Node-00064 - Long	1.000779	Node-00064 - Long	1.000779
Node-00064 - Lat	1.001782	Node-00064 - Lat	1.000935	Node-00064 - Lat	1.000935
Node-00065 - Long	1.00028	Node-00065 - Long	1.000594	Node-00065 - Long	1.000594
Node-00065 - Lat	1.000748	Node-00065 - Lat	1.000722	Node-00065 - Lat	1.000722
Node-00066 - Long	1.000351	Node-00066 - Long	1.00049	Node-00066 - Long	1.00049
Node-00066 - Lat	1.00061	Node-00066 - Lat	1.000853	Node-00066 - Lat	1.000853
Node-00067 - Long	1.000281	Node-00067 - Long	NA	Node-00067 - Long	NA
Node-00067 - Lat	1.000621	Node-00067 - Lat	NA	Node-00067 - Lat	NA
Node-00068 - Long	NA	Node-00068 - Long	NA	Node-00068 - Long	NA

Node-00068 - Lat	NA	Node-00068 - Lat	NA	Node-00068 - Lat	NA
Node-00069 - Long	NA	Node-00069 - Long	NA	Node-00069 - Long	NA
Node-00069 - Lat	NA	Node-00069 - Lat	NA	Node-00069 - Lat	NA
Node-00070 - Long	NA	Node-00070 - Long	NA	Node-00070 - Long	NA
Node-00070 - Lat	NA	Node-00070 - Lat	NA	Node-00070 - Lat	NA
Node-00071 - Long	NA	Node-00071 - Long	NA	Node-00071 - Long	NA
Node-00071 - Lat	NA	Node-00071 - Lat	NA	Node-00071 - Lat	NA
Node-00072 - Long	NA	Node-00072 - Long	NA	Node-00072 - Long	NA
Node-00072 - Lat	NA	Node-00072 - Lat	NA	Node-00072 - Lat	NA
Node-00073 - Long	NA	Node-00073 - Long	1.001166	Node-00073 - Long	1.001166
Node-00073 - Lat	NA	Node-00073 - Lat	1.002828	Node-00073 - Lat	1.002828
Node-00074 - Long	1.001125	Node-00074 - Long	1.00058	Node-00074 - Long	1.00058
Node-00074 - Lat	1.002429	Node-00074 - Lat	1.001084	Node-00074 - Lat	1.001084
Node-00075 - Long	1.000788	Node-00075 - Long	1.000213	Node-00075 - Long	1.000213
Node-00075 - Lat	1.001673	Node-00075 - Lat	1.000287	Node-00075 - Lat	1.000287
Node-00076 - Long	1.000086	Node-00076 - Long	NA	Node-00076 - Long	NA
Node-00076 - Lat	1.000139	Node-00076 - Lat	NA	Node-00076 - Lat	NA
Node-00077 - Long	NA	Node-00077 - Long	NA	Node-00077 - Long	NA
Node-00077 - Lat	NA	Node-00077 - Lat	NA	Node-00077 - Lat	NA
Node-00078 - Long	NA	Node-00078 - Long	NA	Node-00078 - Long	NA
Node-00078 - Lat	NA	Node-00078 - Lat	NA	Node-00078 - Lat	NA
Node-00079 - Long	NA	Node-00079 - Long	NA	Node-00079 - Long	NA
Node-00079 - Lat	NA	Node-00079 - Lat	NA	Node-00079 - Lat	NA
Node-00080 - Long	NA	Node-00080 - Long	NA	Node-00080 - Long	NA
Node-00080 - Lat	NA	Node-00080 - Lat	NA	Node-00080 - Lat	NA
Node-00081 - Long	NA	Node-00081 - Long	NA	Node-00081 - Long	NA
Node-00081 - Lat	NA	Node-00081 - Lat	NA	Node-00081 - Lat	NA
Node-00082 - Long	NA	Node-00082 - Long	1.290808	Node-00082 - Long	1.290808
Node-00082 - Lat	NA	Node-00082 - Lat	1.290892	Node-00082 - Lat	1.290892
Node-00083 - Long	1.160066	Node-00083 - Long	1.290751	Node-00083 - Long	1.290751
Node-00083 - Lat	1.206404	Node-00083 - Lat	1.290723	Node-00083 - Lat	1.290723
Node-00084 - Long	1.198295	Node-00084 - Long	NA	Node-00084 - Long	NA
Node-00084 - Lat	1.19133	Node-00084 - Lat	NA	Node-00084 - Lat	NA
Node-00085 - Long	NA	Node-00085 - Long	NA	Node-00085 - Long	NA
Node-00085 - Lat	NA	Node-00085 - Lat	NA	Node-00085 - Lat	NA
Node-00086 - Long	NA	Node-00086 - Long	1.001109	Node-00086 - Long	1.001109
Node-00086 - Lat	NA	Node-00086 - Lat	1.000178	Node-00086 - Lat	1.000178
Node-00087 - Long	1.000733	Node-00087 - Long	NA	Node-00087 - Long	NA
Node-00087 - Lat	1.000079	Node-00087 - Lat	NA	Node-00087 - Lat	NA
Node-00088 - Long	NA	Node-00088 - Long	NA	Node-00088 - Long	NA
Node-00088 - Lat	NA	Node-00088 - Lat	NA	Node-00088 - Lat	NA
Node-00089 - Long	NA	Node-00089 - Long	NA	Node-00089 - Long	NA
Node-00089 - Lat	NA	Node-00089 - Lat	NA	Node-00089 - Lat	NA
Node-00090 - Long	NA	Node-00090 - Long	1.000279	Node-00090 - Long	1.000279
Node-00090 - Lat	NA	Node-00090 - Lat	1.000561	Node-00090 - Lat	1.000561
Node-00091 - Long	1.000125	Node-00091 - Long	NA	Node-00091 - Long	NA
Node-00091 - Lat	1.00014	Node-00091 - Lat	NA	Node-00091 - Lat	NA
Node-00092 - Long	NA	Node-00092 - Long	0.999971	Node-00092 - Long	0.999971

Node-00092 - Lat	NA	Node-00092 - Lat	0.999987	Node-00092 - Lat	0.999987
Node-00093 - Long	1.00002	Node-00093 - Long	NA	Node-00093 - Long	NA
Node-00093 - Lat	1.000016	Node-00093 - Lat	NA	Node-00093 - Lat	NA
Node-00094 - Long	NA	Node-00094 - Long	NA	Node-00094 - Long	NA
Node-00094 - Lat	NA	Node-00094 - Lat	NA	Node-00094 - Lat	NA
Node-00095 - Long	NA	Node-00095 - Long	1.002842	Node-00095 - Long	1.002842
Node-00095 - Lat	NA	Node-00095 - Lat	1.001787	Node-00095 - Lat	1.001787
Node-00096 - Long	1.002565	Node-00096 - Long	NA	Node-00096 - Long	NA
Node-00096 - Lat	1.001573	Node-00096 - Lat	NA	Node-00096 - Lat	NA
Node-00097 - Long	NA	Node-00097 - Long	NA	Node-00097 - Long	NA
Node-00097 - Lat	NA	Node-00097 - Lat	NA	Node-00097 - Lat	NA
Node-00098 - Long	NA	Node-00098 - Long	NA	Node-00098 - Long	NA
Node-00098 - Lat	NA	Node-00098 - Lat	NA	Node-00098 - Lat	NA
Node-00099 - Long	NA	Node-00099 - Long	1.00379	Node-00099 - Long	1.00379
Node-00099 - Lat	NA	Node-00099 - Lat	1.00332	Node-00099 - Lat	1.00332
Node-00100 - Long	1.002625	Node-00100 - Long	1.15877	Node-00100 - Long	1.15877
Node-00100 - Lat	1.002103	Node-00100 - Lat	1.150235	Node-00100 - Lat	1.150235
Node-00101 - Long	1.290395	Node-00101 - Long	1.222686	Node-00101 - Long	1.222686
Node-00101 - Lat	1.290376	Node-00101 - Lat	1.179223	Node-00101 - Lat	1.179223
Node-00102 - Long	1.289247	Node-00102 - Long	NA	Node-00102 - Long	NA
Node-00102 - Lat	1.290458	Node-00102 - Lat	NA	Node-00102 - Lat	NA
Node-00103 - Long	NA	Node-00103 - Long	NA	Node-00103 - Long	NA
Node-00103 - Lat	NA	Node-00103 - Lat	NA	Node-00103 - Lat	NA
Node-00104 - Long	NA	Node-00104 - Long	NA	Node-00104 - Long	NA
Node-00104 - Lat	NA	Node-00104 - Lat	NA	Node-00104 - Lat	NA
Node-00105 - Long	NA	Node-00105 - Long	1.000935	Node-00105 - Long	1.000935
Node-00105 - Lat	NA	Node-00105 - Lat	1.000858	Node-00105 - Lat	1.000858
Node-00106 - Long	1.000289	Node-00106 - Long	1.000214	Node-00106 - Long	1.000214
Node-00106 - Lat	1.000359	Node-00106 - Lat	1.000214	Node-00106 - Lat	1.000214
Node-00107 - Long	1.000198	Node-00107 - Long	1.020477	Node-00107 - Long	1.020477
Node-00107 - Lat	1.000198	Node-00107 - Lat	1.020953	Node-00107 - Lat	1.020953
Node-00108 - Long	1.017416	Node-00108 - Long	1.01765	Node-00108 - Long	1.01765
Node-00108 - Lat	1.015275	Node-00108 - Lat	1.018125	Node-00108 - Lat	1.018125
Node-00109 - Long	1.014391	Node-00109 - Long	1.017786	Node-00109 - Long	1.017786
Node-00109 - Lat	1.013008	Node-00109 - Lat	1.017547	Node-00109 - Lat	1.017547
Node-00110 - Long	1.013608	Node-00110 - Long	NA	Node-00110 - Long	NA
Node-00110 - Lat	1.012072	Node-00110 - Lat	NA	Node-00110 - Lat	NA
Node-00111 - Long	NA	Node-00111 - Long	1.030472	Node-00111 - Long	1.030472
Node-00111 - Lat	NA	Node-00111 - Lat	1.021306	Node-00111 - Lat	1.021306
Node-00112 - Long	1.019242	Node-00112 - Long	NA	Node-00112 - Long	NA
Node-00112 - Lat	1.014624	Node-00112 - Lat	NA	Node-00112 - Lat	NA
Node-00113 - Long	NA	Node-00113 - Long	1.00553	Node-00113 - Long	1.00553
Node-00113 - Lat	NA	Node-00113 - Lat	1.00139	Node-00113 - Lat	1.00139
Node-00114 - Long	1.005034	Node-00114 - Long	1.001761	Node-00114 - Long	1.001761
Node-00114 - Lat	1.001986	Node-00114 - Lat	1.000219	Node-00114 - Lat	1.000219
Node-00115 - Long	1.001689	Node-00115 - Long	1.001032	Node-00115 - Long	1.001032
Node-00115 - Lat	1.0006	Node-00115 - Lat	1.000015	Node-00115 - Lat	1.000015
Node-00116 - Long	1.000802	Node-00116 - Long	NA	Node-00116 - Long	NA

Node-00116 - Lat	1.000184	Node-00116 - Lat	NA	Node-00116 - Lat	NA
Node-00117 - Long	NA	Node-00117 - Long	NA	Node-00117 - Long	NA
Node-00117 - Lat	NA	Node-00117 - Lat	NA	Node-00117 - Lat	NA
Node-00118 - Long	NA	Node-00118 - Long	1.000292	Node-00118 - Long	1.000292
Node-00118 - Lat	NA	Node-00118 - Lat	1.000052	Node-00118 - Lat	1.000052
Node-00119 - Long	1.000215	Node-00119 - Long	1.000067	Node-00119 - Long	1.000067
Node-00119 - Lat	1.000186	Node-00119 - Lat	1.000077	Node-00119 - Lat	1.000077
Node-00120 - Long	1.00002	Node-00120 - Long	NA	Node-00120 - Long	NA
Node-00120 - Lat	1.000017	Node-00120 - Lat	NA	Node-00120 - Lat	NA
Node-00121 - Long	NA	Node-00121 - Long	NA	Node-00121 - Long	NA
Node-00121 - Lat	NA	Node-00121 - Lat	NA	Node-00121 - Lat	NA
Node-00122 - Long	NA	Node-00122 - Long	1.006969	Node-00122 - Long	1.006969
Node-00122 - Lat	NA	Node-00122 - Lat	1.012244	Node-00122 - Lat	1.012244
Node-00123 - Long	1.004993	Node-00123 - Long	NA	Node-00123 - Long	NA
Node-00123 - Lat	1.009431	Node-00123 - Lat	NA	Node-00123 - Lat	NA
Node-00124 - Long	NA	Node-00124 - Long	NA	Node-00124 - Long	NA
Node-00124 - Lat	NA	Node-00124 - Lat	NA	Node-00124 - Lat	NA
Node-00125 - Long	NA	Node-00125 - Long	1.005593	Node-00125 - Long	1.005593
Node-00125 - Lat	NA	Node-00125 - Lat	1.010052	Node-00125 - Lat	1.010052
Node-00126 - Long	1.004002	Node-00126 - Long	1.000151	Node-00126 - Long	1.000151
Node-00126 - Lat	1.006129	Node-00126 - Lat	1.000151	Node-00126 - Lat	1.000151
Node-00127 - Long	1.000447	Node-00127 - Long	NA	Node-00127 - Long	NA
Node-00127 - Lat	1.000447	Node-00127 - Lat	NA	Node-00127 - Lat	NA
Node-00128 - Long	NA	Node-00128 - Long	1.09721	Node-00128 - Long	1.09721
Node-00128 - Lat	NA	Node-00128 - Lat	1.095468	Node-00128 - Lat	1.095468
Node-00129 - Long	1.266028	Node-00129 - Long	1.000463	Node-00129 - Long	1.000463
Node-00129 - Lat	1.275971	Node-00129 - Lat	1.000463	Node-00129 - Lat	1.000463
Node-00130 - Long	1.00021	Node-00130 - Long	1.105154	Node-00130 - Long	1.105154
Node-00130 - Lat	1.00021	Node-00130 - Lat	1.120742	Node-00130 - Lat	1.120742
Node-00131 - Long	1.254552	Node-00131 - Long	NA	Node-00131 - Long	NA
Node-00131 - Lat	1.258607	Node-00131 - Lat	NA	Node-00131 - Lat	NA
Node-00132 - Long	NA	Node-00132 - Long	NA	Node-00132 - Long	NA
Node-00132 - Lat	NA	Node-00132 - Lat	NA	Node-00132 - Lat	NA
Node-00133 - Long	NA	Node-00133 - Long	1.093063	Node-00133 - Long	1.093063
Node-00133 - Lat	NA	Node-00133 - Lat	1.092582	Node-00133 - Lat	1.092582
Node-00134 - Long	1.268829	Node-00134 - Long	NA	Node-00134 - Long	NA
Node-00134 - Lat	1.262204	Node-00134 - Lat	NA	Node-00134 - Lat	NA
Node-00135 - Long	NA	Node-00135 - Long	1.159206	Node-00135 - Long	1.159206
Node-00135 - Lat	NA	Node-00135 - Lat	1.196086	Node-00135 - Lat	1.196086
Node-00136 - Long	1.416647	Node-00136 - Long	NA	Node-00136 - Long	NA
Node-00136 - Lat	1.486579	Node-00136 - Lat	NA	Node-00136 - Lat	NA
Node-00137 - Long	NA	Node-00137 - Long	NA	Node-00137 - Long	NA
Node-00137 - Lat	NA	Node-00137 - Lat	NA	Node-00137 - Lat	NA
Node-00138 - Long	NA	Node-00138 - Long	1.047052	Node-00138 - Long	1.047052
Node-00138 - Lat	NA	Node-00138 - Lat	1.059712	Node-00138 - Lat	1.059712
Node-00139 - Long	1.155564	Node-00139 - Long	NA	Node-00139 - Long	NA
Node-00139 - Lat	1.185945	Node-00139 - Lat	NA	Node-00139 - Lat	NA
Node-00140 - Long	NA	Node-00140 - Long	NA	Node-00140 - Long	NA

Node-00140 - Lat	NA	Node-00140 - Lat	NA	Node-00140 - Lat	NA
Node-00141 - Long	NA	Node-00141 - Long	1.105363	Node-00141 - Long	1.105363
Node-00141 - Lat	NA	Node-00141 - Lat	1.131326	Node-00141 - Lat	1.131326
Node-00142 - Long	1.137312	Node-00142 - Long	NA	Node-00142 - Long	NA
Node-00142 - Lat	1.250369	Node-00142 - Lat	NA	Node-00142 - Lat	NA
Node-00143 - Long	NA	Node-00143 - Long	NA	Node-00143 - Long	NA
Node-00143 - Lat	NA	Node-00143 - Lat	NA	Node-00143 - Lat	NA
Node-00144 - Long	NA	Node-00144 - Long	1.069085	Node-00144 - Long	1.069085
Node-00144 - Lat	NA	Node-00144 - Lat	1.096441	Node-00144 - Lat	1.096441
Node-00145 - Long	1.051404	Node-00145 - Long	NA	Node-00145 - Long	NA
Node-00145 - Lat	1.089233	Node-00145 - Lat	NA	Node-00145 - Lat	NA
Node-00146 - Long	NA	Node-00146 - Long	NA	Node-00146 - Long	NA
Node-00146 - Lat	NA	Node-00146 - Lat	NA	Node-00146 - Lat	NA
Node-00147 - Long	NA	Node-00147 - Long	1.008261	Node-00147 - Long	1.008261
Node-00147 - Lat	NA	Node-00147 - Lat	1.002283	Node-00147 - Lat	1.002283
Node-00148 - Long	1.008481	Node-00148 - Long	1.003482	Node-00148 - Long	1.003482
Node-00148 - Lat	1.003565	Node-00148 - Lat	1.001286	Node-00148 - Lat	1.001286
Node-00149 - Long	1.004241	Node-00149 - Long	1.002907	Node-00149 - Long	1.002907
Node-00149 - Lat	1.001878	Node-00149 - Lat	1.001403	Node-00149 - Lat	1.001403
Node-00150 - Long	1.00474	Node-00150 - Long	NA	Node-00150 - Long	NA
Node-00150 - Lat	1.001575	Node-00150 - Lat	NA	Node-00150 - Lat	NA
Node-00151 - Long	NA	Node-00151 - Long	NA	Node-00151 - Long	NA
Node-00151 - Lat	NA	Node-00151 - Lat	NA	Node-00151 - Lat	NA
Node-00152 - Long	NA	Node-00152 - Long	NA	Node-00152 - Long	NA
Node-00152 - Lat	NA	Node-00152 - Lat	NA	Node-00152 - Lat	NA
Node-00153 - Long	NA	Node-00153 - Long	NA	Node-00153 - Long	NA
Node-00153 - Lat	NA	Node-00153 - Lat	NA	Node-00153 - Lat	NA
Node-00154 - Long	NA	Node-00154 - Long	NA	Node-00154 - Long	NA
Node-00154 - Lat	NA	Node-00154 - Lat	NA	Node-00154 - Lat	NA
Node-00155 - Long	NA	Node-00155 - Long	1.000885	Node-00155 - Long	1.000885
Node-00155 - Lat	NA	Node-00155 - Lat	1.000318	Node-00155 - Lat	1.000318
Node-00156 - Long	1.00146	Node-00156 - Long	NA	Node-00156 - Long	NA
Node-00156 - Lat	1.00035	Node-00156 - Lat	NA	Node-00156 - Lat	NA
Node-00157 - Long	NA	Node-00157 - Long	NA	Node-00157 - Long	NA
Node-00157 - Lat	NA	Node-00157 - Lat	NA	Node-00157 - Lat	NA
Node-00158 - Long	NA	Node-00158 - Long	1.00139	Node-00158 - Long	1.00139
Node-00158 - Lat	NA	Node-00158 - Lat	1.000787	Node-00158 - Lat	1.000787
Node-00159 - Long	1.001806	Node-00159 - Long	1.000447	Node-00159 - Long	1.000447
Node-00159 - Lat	1.000631	Node-00159 - Lat	1.011446	Node-00159 - Lat	1.011446
Node-00160 - Long	1.000961	Node-00160 - Long	NA	Node-00160 - Long	NA
Node-00160 - Lat	1.035432	Node-00160 - Lat	NA	Node-00160 - Lat	NA
Node-00161 - Long	NA	Node-00161 - Long	1.00054	Node-00161 - Long	1.00054
Node-00161 - Lat	NA	Node-00161 - Lat	1.008707	Node-00161 - Lat	1.008707
Node-00162 - Long	1.000535	Node-00162 - Long	1.002461	Node-00162 - Long	1.002461
Node-00162 - Lat	1.028297	Node-00162 - Lat	1.004272	Node-00162 - Lat	1.004272
Node-00163 - Long	1.004031	Node-00163 - Long	1.002296	Node-00163 - Long	1.002296
Node-00163 - Lat	1.006017	Node-00163 - Lat	1.004565	Node-00163 - Lat	1.004565
Node-00164 - Long	1.003663	Node-00164 - Long	1.002223	Node-00164 - Long	1.002223

Node-00164 - Lat	1.005899	Node-00164 - Lat	1.004651	Node-00164 - Lat	1.004651
Node-00165 - Long	1.003539	Node-00165 - Long	1.001577	Node-00165 - Long	1.001577
Node-00165 - Lat	1.006041	Node-00165 - Lat	1.006445	Node-00165 - Lat	1.006445
Node-00166 - Long	1.003239	Node-00166 - Long	1.001025	Node-00166 - Long	1.001025
Node-00166 - Lat	1.006445	Node-00166 - Lat	1.009672	Node-00166 - Lat	1.009672
Node-00167 - Long	1.001888	Node-00167 - Long	1.005879	Node-00167 - Long	1.005879
Node-00167 - Lat	1.007941	Node-00167 - Lat	1.004682	Node-00167 - Lat	1.004682
Node-00168 - Long	1.00516	Node-00168 - Long	1.006847	Node-00168 - Long	1.006847
Node-00168 - Lat	1.005044	Node-00168 - Lat	1.005644	Node-00168 - Lat	1.005644
Node-00169 - Long	1.006284	Node-00169 - Long	1.008306	Node-00169 - Long	1.008306
Node-00169 - Lat	1.007157	Node-00169 - Lat	1.00672	Node-00169 - Lat	1.00672
Node-00170 - Long	1.007954	Node-00170 - Long	1.011935	Node-00170 - Long	1.011935
Node-00170 - Lat	1.0089	Node-00170 - Lat	1.008025	Node-00170 - Lat	1.008025
Node-00171 - Long	1.011312	Node-00171 - Long	1.016459	Node-00171 - Long	1.016459
Node-00171 - Lat	1.012421	Node-00171 - Lat	1.012222	Node-00171 - Lat	1.012222
Node-00172 - Long	1.0155	Node-00172 - Long	1.006427	Node-00172 - Long	1.006427
Node-00172 - Lat	1.016636	Node-00172 - Lat	1.005603	Node-00172 - Lat	1.005603
Node-00173 - Long	1.005542	Node-00173 - Long	1.005908	Node-00173 - Long	1.005908
Node-00173 - Lat	1.005977	Node-00173 - Lat	1.004318	Node-00173 - Lat	1.004318
Node-00174 - Long	1.005453	Node-00174 - Long	1.000126	Node-00174 - Long	1.000126
Node-00174 - Lat	1.004527	Node-00174 - Lat	1.000072	Node-00174 - Lat	1.000072
Node-00175 - Long	1.001073	Node-00175 - Long	1.000051	Node-00175 - Long	1.000051
Node-00175 - Lat	1.000229	Node-00175 - Lat	1.000037	Node-00175 - Lat	1.000037
Node-00176 - Long	1.000579	Node-00176 - Long	NA	Node-00176 - Long	NA
Node-00176 - Lat	1.0002	Node-00176 - Lat	NA	Node-00176 - Lat	NA
Node-00177 - Long	NA	Node-00177 - Long	1.000034	Node-00177 - Long	1.000034
Node-00177 - Lat	NA	Node-00177 - Lat	1.000347	Node-00177 - Lat	1.000347
Node-00178 - Long	1.000231	Node-00178 - Long	NA	Node-00178 - Long	NA
Node-00178 - Lat	1.000654	Node-00178 - Lat	NA	Node-00178 - Lat	NA
Node-00179 - Long	NA	Node-00179 - Long	NA	Node-00179 - Long	NA
Node-00179 - Lat	NA	Node-00179 - Lat	NA	Node-00179 - Lat	NA
Node-00180 - Long	NA	Node-00180 - Long	1.000093	Node-00180 - Long	1.000093
Node-00180 - Lat	NA	Node-00180 - Lat	1.000127	Node-00180 - Lat	1.000127
Node-00181 - Long	1.000492	Node-00181 - Long	1.000484	Node-00181 - Long	1.000484
Node-00181 - Lat	1.000161	Node-00181 - Lat	1.00003	Node-00181 - Lat	1.00003
Node-00182 - Long	1.000953	Node-00182 - Long	NA	Node-00182 - Long	NA
Node-00182 - Lat	1.000262	Node-00182 - Lat	NA	Node-00182 - Lat	NA
Node-00183 - Long	NA	Node-00183 - Long	NA	Node-00183 - Long	NA
Node-00183 - Lat	NA	Node-00183 - Lat	NA	Node-00183 - Lat	NA
Node-00184 - Long	NA	Node-00184 - Long	NA	Node-00184 - Long	NA
Node-00184 - Lat	NA	Node-00184 - Lat	NA	Node-00184 - Lat	NA
Node-00185 - Long	NA	Node-00185 - Long	NA	Node-00185 - Long	NA
Node-00185 - Lat	NA	Node-00185 - Lat	NA	Node-00185 - Lat	NA
Node-00186 - Long	NA	Node-00186 - Long	NA	Node-00186 - Long	NA
Node-00186 - Lat	NA	Node-00186 - Lat	NA	Node-00186 - Lat	NA
Node-00187 - Long	NA	Node-00187 - Long	0.999996	Node-00187 - Long	0.999996
Node-00187 - Lat	NA	Node-00187 - Lat	1.000062	Node-00187 - Lat	1.000062
Node-00188 - Long	1.000154	Node-00188 - Long	NA	Node-00188 - Long	NA

Node-00188 - Lat	1.0001	Node-00188 - Lat	NA	Node-00188 - Lat	NA
Node-00189 - Long	NA	Node-00189 - Long	1.001578	Node-00189 - Long	1.001578
Node-00189 - Lat	NA	Node-00189 - Lat	1.000291	Node-00189 - Lat	1.000291
Node-00190 - Long	1.000523	Node-00190 - Long	1.000024	Node-00190 - Long	1.000024
Node-00190 - Lat	1.00017	Node-00190 - Lat	1.000019	Node-00190 - Lat	1.000019
Node-00191 - Long	1.000116	Node-00191 - Long	NA	Node-00191 - Long	NA
Node-00191 - Lat	1.000075	Node-00191 - Lat	NA	Node-00191 - Lat	NA
Node-00192 - Long	NA	Node-00192 - Long	NA	Node-00192 - Long	NA
Node-00192 - Lat	NA	Node-00192 - Lat	NA	Node-00192 - Lat	NA
Node-00193 - Long	NA	Node-00193 - Long	1.000294	Node-00193 - Long	1.000294
Node-00193 - Lat	NA	Node-00193 - Lat	1.000302	Node-00193 - Lat	1.000302
Node-00194 - Long	1.000061	Node-00194 - Long	1.000061	Node-00194 - Long	1.000061
Node-00194 - Lat	1.000162	Node-00194 - Lat	1.000174	Node-00194 - Lat	1.000174
Node-00195 - Long	1.000151	Node-00195 - Long	1.000105	Node-00195 - Long	1.000105
Node-00195 - Lat	1.000171	Node-00195 - Lat	1.00001	Node-00195 - Lat	1.00001
Node-00196 - Long	1.000072	Node-00196 - Long	1.000019	Node-00196 - Long	1.000019
Node-00196 - Lat	1.000111	Node-00196 - Lat	1.000298	Node-00196 - Lat	1.000298
Node-00197 - Long	1.000037	Node-00197 - Long	1.000058	Node-00197 - Long	1.000058
Node-00197 - Lat	1.000188	Node-00197 - Lat	1.000239	Node-00197 - Lat	1.000239
Node-00198 - Long	1.000057	Node-00198 - Long	NA	Node-00198 - Long	NA
Node-00198 - Lat	1.000192	Node-00198 - Lat	NA	Node-00198 - Lat	NA
Node-00199 - Long	NA	Node-00199 - Long	1.001454	Node-00199 - Long	1.001454
Node-00199 - Lat	NA	Node-00199 - Lat	1.001673	Node-00199 - Lat	1.001673
Node-00200 - Long	1.002663	Node-00200 - Long	NA	Node-00200 - Long	NA
Node-00200 - Lat	1.001996	Node-00200 - Lat	NA	Node-00200 - Lat	NA
Node-00201 - Long	NA	Node-00201 - Long	NA	Node-00201 - Long	NA
Node-00201 - Lat	NA	Node-00201 - Lat	NA	Node-00201 - Lat	NA
Node-00202 - Long	NA	Node-00202 - Long	1.000106	Node-00202 - Long	1.000106
Node-00202 - Lat	NA	Node-00202 - Lat	1.000177	Node-00202 - Lat	1.000177
Node-00203 - Long	1.00003	Node-00203 - Long	1.000005	Node-00203 - Long	1.000005
Node-00203 - Lat	1.000064	Node-00203 - Lat	1.000005	Node-00203 - Lat	1.000005
Node-00204 - Long	1.000131	Node-00204 - Long	NA	Node-00204 - Long	NA
Node-00204 - Lat	1.000131	Node-00204 - Lat	NA	Node-00204 - Lat	NA
Node-00205 - Long	NA	Node-00205 - Long	NA	Node-00205 - Long	NA
Node-00205 - Lat	NA	Node-00205 - Lat	NA	Node-00205 - Lat	NA
Node-00206 - Long	NA	Node-00206 - Long	1.000003	Node-00206 - Long	1.000003
Node-00206 - Lat	NA	Node-00206 - Lat	1.000003	Node-00206 - Lat	1.000003
Node-00207 - Long	0.999967	Node-00207 - Long	NA	Node-00207 - Long	NA
Node-00207 - Lat	0.999967	Node-00207 - Lat	NA	Node-00207 - Lat	NA
Node-00208 - Long	NA	Node-00208 - Long	NA	Node-00208 - Long	NA
Node-00208 - Lat	NA	Node-00208 - Lat	NA	Node-00208 - Lat	NA
Node-00209 - Long	NA	Node-00209 - Long	NA	Node-00209 - Long	NA
Node-00209 - Lat	NA	Node-00209 - Lat	NA	Node-00209 - Lat	NA
Node-00210 - Long	NA	Node-00210 - Long	NA	Node-00210 - Long	NA
Node-00210 - Lat	NA	Node-00210 - Lat	NA	Node-00210 - Lat	NA
Node-00211 - Long	NA	Node-00211 - Long	0.999971	Node-00211 - Long	0.999971
Node-00211 - Lat	NA	Node-00211 - Lat	0.999971	Node-00211 - Lat	0.999971
Node-00212 - Long	1.000155	Node-00212 - Long	NA	Node-00212 - Long	NA

Node-00212 - Lat	1.000155	Node-00212 - Lat	NA	Node-00212 - Lat	NA
Node-00213 - Long	NA	Node-00213 - Long	1.000414	Node-00213 - Long	1.000414
Node-00213 - Lat	NA	Node-00213 - Lat	1.000334	Node-00213 - Lat	1.000334
Node-00214 - Long	1.00032	Node-00214 - Long	1.000259	Node-00214 - Long	1.000259
Node-00214 - Lat	1.000291	Node-00214 - Lat	1.000212	Node-00214 - Lat	1.000212
Node-00215 - Long	1.000194	Node-00215 - Long	1.000161	Node-00215 - Long	1.000161
Node-00215 - Lat	1.000064	Node-00215 - Lat	1.000156	Node-00215 - Lat	1.000156
Node-00216 - Long	1.000161	Node-00216 - Long	1.000134	Node-00216 - Long	1.000134
Node-00216 - Lat	1.000027	Node-00216 - Lat	1.000124	Node-00216 - Lat	1.000124
Node-00217 - Long	1.0001	Node-00217 - Long	NA	Node-00217 - Long	NA
Node-00217 - Lat	1.000015	Node-00217 - Lat	NA	Node-00217 - Lat	NA
Node-00218 - Long	NA	Node-00218 - Long	NA	Node-00218 - Long	NA
Node-00218 - Lat	NA	Node-00218 - Lat	NA	Node-00218 - Lat	NA
Node-00219 - Long	NA	Node-00219 - Long	1.000061	Node-00219 - Long	1.000061
Node-00219 - Lat	NA	Node-00219 - Lat	1.000048	Node-00219 - Lat	1.000048
Node-00220 - Long	1.000049	Node-00220 - Long	NA	Node-00220 - Long	NA
Node-00220 - Lat	1.000045	Node-00220 - Lat	NA	Node-00220 - Lat	NA
Node-00221 - Long	NA	Node-00221 - Long	0.99997	Node-00221 - Long	0.99997
Node-00221 - Lat	NA	Node-00221 - Lat	0.99997	Node-00221 - Lat	0.99997
Node-00222 - Long	1.000054	Node-00222 - Long	NA	Node-00222 - Long	NA
Node-00222 - Lat	1.000054	Node-00222 - Lat	NA	Node-00222 - Lat	NA
Node-00223 - Long	NA	Node-00223 - Long	NA	Node-00223 - Long	NA
Node-00223 - Lat	NA	Node-00223 - Lat	NA	Node-00223 - Lat	NA
Node-00224 - Long	NA	Node-00224 - Long	NA	Node-00224 - Long	NA
Node-00224 - Lat	NA	Node-00224 - Lat	NA	Node-00224 - Lat	NA
Node-00225 - Long	NA	Node-00225 - Long	NA	Node-00225 - Long	NA
Node-00225 - Lat	NA	Node-00225 - Lat	NA	Node-00225 - Lat	NA
Node-00226 - Long	NA	Node-00226 - Long	1.0049	Node-00226 - Long	1.0049
Node-00226 - Lat	NA	Node-00226 - Lat	1.000899	Node-00226 - Lat	1.000899
Node-00227 - Long	1.001517	Node-00227 - Long	1.002015	Node-00227 - Long	1.002015
Node-00227 - Lat	1.000968	Node-00227 - Lat	1.000541	Node-00227 - Lat	1.000541
Node-00228 - Long	1.397536	Node-00228 - Long	1.001434	Node-00228 - Long	1.001434
Node-00228 - Lat	1.368156	Node-00228 - Lat	1.000623	Node-00228 - Lat	1.000623
Node-00229 - Long	1.755068	Node-00229 - Long	1.000716	Node-00229 - Long	1.000716
Node-00229 - Lat	1.475074	Node-00229 - Lat	1.000795	Node-00229 - Lat	1.000795
Node-00230 - Long	1.538747	Node-00230 - Long	1.000431	Node-00230 - Long	1.000431
Node-00230 - Lat	1.878949	Node-00230 - Lat	1.000645	Node-00230 - Lat	1.000645
Node-00231 - Long	NA	Node-00231 - Long	1.000512	Node-00231 - Long	1.000512
Node-00231 - Lat	NA	Node-00231 - Lat	1.000614	Node-00231 - Lat	1.000614
Node-00232 - Long	NA	Node-00232 - Long	1.000397	Node-00232 - Long	1.000397
Node-00232 - Lat	NA	Node-00232 - Lat	1.00053	Node-00232 - Lat	1.00053
Node-00233 - Long	NA	Node-00233 - Long	1.000446	Node-00233 - Long	1.000446
Node-00233 - Lat	NA	Node-00233 - Lat	1.000412	Node-00233 - Lat	1.000412
Node-00234 - Long	1.002201	Node-00234 - Long	1.000044	Node-00234 - Long	1.000044
Node-00234 - Lat	1.001803	Node-00234 - Lat	1.000205	Node-00234 - Lat	1.000205
Node-00235 - Long	NA	Node-00235 - Long	1.000085	Node-00235 - Long	1.000085
Node-00235 - Lat	NA	Node-00235 - Lat	1.000045	Node-00235 - Lat	1.000045
Node-00236 - Long	NA	Node-00236 - Long	1.000136	Node-00236 - Long	1.000136

Node-00236 - Lat	NA	Node-00236 - Lat	1.000243	Node-00236 - Lat	1.000243
Node-00237 - Long	1.000033	Node-00237 - Long	1.000042	Node-00237 - Long	1.000042
Node-00237 - Lat	1.000007	Node-00237 - Lat	1.000035	Node-00237 - Lat	1.000035
Node-00238 - Long	1.000601	Node-00238 - Long	1.000087	Node-00238 - Long	1.000087
Node-00238 - Lat	1.000834	Node-00238 - Lat	1.000015	Node-00238 - Lat	1.000015
Node-00239 - Long	1.000548	Node-00239 - Long	1.378107	Node-00239 - Long	1.378107
Node-00239 - Lat	1.000574	Node-00239 - Lat	1.365827	Node-00239 - Lat	1.365827
Node-00240 - Long	1.000067	Node-00240 - Long	1.506546	Node-00240 - Long	1.506546
Node-00240 - Lat	1.000159	Node-00240 - Lat	1.493388	Node-00240 - Lat	1.493388
Node-00241 - Long	NA	Node-00241 - Long	1.234883	Node-00241 - Long	1.234883
Node-00241 - Lat	NA	Node-00241 - Lat	1.192424	Node-00241 - Lat	1.192424
Node-00242 - Long	0.999995	Node-00242 - Long	NA	Node-00242 - Long	NA
Node-00242 - Lat	0.999995	Node-00242 - Lat	NA	Node-00242 - Lat	NA
Node-00243 - Long	1.000182	Node-00243 - Long	NA	Node-00243 - Long	NA
Node-00243 - Lat	1.000582	Node-00243 - Lat	NA	Node-00243 - Lat	NA
Node-00244 - Long	NA	Node-00244 - Long	NA	Node-00244 - Long	NA
Node-00244 - Lat	NA	Node-00244 - Lat	NA	Node-00244 - Lat	NA
Node-00245 - Long	0.999971	Node-00245 - Long	1.000003	Node-00245 - Long	1.000003
Node-00245 - Lat	0.999971	Node-00245 - Lat	1.00002	Node-00245 - Lat	1.00002
Node-00246 - Long	0.999959	Node-00246 - Long	NA	Node-00246 - Long	NA
Node-00246 - Lat	0.999959	Node-00246 - Lat	NA	Node-00246 - Lat	NA
Node-00247 - Long	NA	Node-00247 - Long	NA	Node-00247 - Long	NA
Node-00247 - Lat	NA	Node-00247 - Lat	NA	Node-00247 - Lat	NA
Node-00248 - Long	NA	Node-00248 - Long	1.06174	Node-00248 - Long	1.06174
Node-00248 - Lat	NA	Node-00248 - Lat	1.06174	Node-00248 - Lat	1.06174
Node-00249 - Long	1.00077	Node-00249 - Long	NA	Node-00249 - Long	NA
Node-00249 - Lat	1.000496	Node-00249 - Lat	NA	Node-00249 - Lat	NA
Node-00250 - Long	1.000604	Node-00250 - Long	1.000189	Node-00250 - Long	1.000189
Node-00250 - Lat	1.000257	Node-00250 - Lat	1.000035	Node-00250 - Lat	1.000035
Node-00251 - Long	1.000419	Node-00251 - Long	1.000114	Node-00251 - Long	1.000114
Node-00251 - Lat	1.000239	Node-00251 - Lat	1.000216	Node-00251 - Lat	1.000216
Node-00252 - Long	NA	Node-00252 - Long	NA	Node-00252 - Long	NA
Node-00252 - Lat	NA	Node-00252 - Lat	NA	Node-00252 - Lat	NA
Node-00253 - Long	1.000152	Node-00253 - Long	0.999977	Node-00253 - Long	0.999977
Node-00253 - Lat	1.000089	Node-00253 - Lat	0.999977	Node-00253 - Lat	0.999977
Node-00254 - Long	1.000111	Node-00254 - Long	1.00013	Node-00254 - Long	1.00013
Node-00254 - Lat	1.00008	Node-00254 - Lat	1.00003	Node-00254 - Lat	1.00003
Node-00255 - Long	1.000332	Node-00255 - Long	NA	Node-00255 - Long	NA
Node-00255 - Lat	1.000193	Node-00255 - Lat	NA	Node-00255 - Lat	NA
Node-00256 - Long	NA	Node-00256 - Long	1.000011	Node-00256 - Long	1.000011
Node-00256 - Lat	NA	Node-00256 - Lat	1.00003	Node-00256 - Lat	1.00003
Node-00257 - Long	NA	Node-00257 - Long	NA	Node-00257 - Long	NA
Node-00257 - Lat	NA	Node-00257 - Lat	NA	Node-00257 - Lat	NA
Node-00258 - Long	1.000104	Node-00258 - Long	1.000135	Node-00258 - Long	1.000135
Node-00258 - Lat	1.000059	Node-00258 - Lat	1.000135	Node-00258 - Lat	1.000135
Node-00259 - Long	0.999985	Node-00259 - Long	1.000666	Node-00259 - Long	1.000666
Node-00259 - Lat	1.000058	Node-00259 - Lat	1.000469	Node-00259 - Lat	1.000469
Node-00260 - Long	0.999984	Node-00260 - Long	NA	Node-00260 - Long	NA

Node-00260 - Lat	1.000001	Node-00260 - Lat	NA	Node-00260 - Lat	NA
Node-00261 - Long	0.999983	Node-00261 - Long	NA	Node-00261 - Long	NA
Node-00261 - Lat	0.999983	Node-00261 - Lat	NA	Node-00261 - Lat	NA
Node-00262 - Long	NA	Node-00262 - Long	NA	Node-00262 - Long	NA
Node-00262 - Lat	NA	Node-00262 - Lat	NA	Node-00262 - Lat	NA
Node-00263 - Long	NA	Node-00263 - Long	1.000219	Node-00263 - Long	1.000219
Node-00263 - Lat	NA	Node-00263 - Lat	1.00051	Node-00263 - Lat	1.00051
Node-00264 - Long	NA	Node-00264 - Long	NA	Node-00264 - Long	NA
Node-00264 - Lat	NA	Node-00264 - Lat	NA	Node-00264 - Lat	NA
Node-00265 - Long	1.00005	Node-00265 - Long	NA	Node-00265 - Long	NA
Node-00265 - Lat	1.00004	Node-00265 - Lat	NA	Node-00265 - Lat	NA
Node-00266 - Long	NA	Node-00266 - Long	NA	Node-00266 - Long	NA
Node-00266 - Lat	NA	Node-00266 - Lat	NA	Node-00266 - Lat	NA
Node-00267 - Long	NA	Node-00267 - Long	NA	Node-00267 - Long	NA
Node-00267 - Lat	NA	Node-00267 - Lat	NA	Node-00267 - Lat	NA
Node-00268 - Long	NA	Node-00268 - Long	NA	Node-00268 - Long	NA
Node-00268 - Lat	NA	Node-00268 - Lat	NA	Node-00268 - Lat	NA
Node-00269 - Long	NA	Node-00269 - Long	NA	Node-00269 - Long	NA
Node-00269 - Lat	NA	Node-00269 - Lat	NA	Node-00269 - Lat	NA
Node-00270 - Long	NA	Node-00270 - Long	NA	Node-00270 - Long	NA
Node-00270 - Lat	NA	Node-00270 - Lat	NA	Node-00270 - Lat	NA
Node-00271 - Long	1.000066	Node-00271 - Long	NA	Node-00271 - Long	NA
Node-00271 - Lat	1.00038	Node-00271 - Lat	NA	Node-00271 - Lat	NA
Node-00272 - Long	NA	Node-00272 - Long	1.004056	Node-00272 - Long	1.004056
Node-00272 - Lat	NA	Node-00272 - Lat	1.004251	Node-00272 - Lat	1.004251
Node-00273 - Long	NA	Node-00273 - Long	NA	Node-00273 - Long	NA
Node-00273 - Lat	NA	Node-00273 - Lat	NA	Node-00273 - Lat	NA
Node-00274 - Long	1.000316	Node-00274 - Long	NA	Node-00274 - Long	NA
Node-00274 - Lat	1.000409	Node-00274 - Lat	NA	Node-00274 - Lat	NA
Node-00275 - Long	1.000299	Node-00275 - Long	1.002682	Node-00275 - Long	1.002682
Node-00275 - Lat	1.000122	Node-00275 - Lat	1.00076	Node-00275 - Lat	1.00076
Node-00276 - Long	1.000268	Node-00276 - Long	1.001823	Node-00276 - Long	1.001823
Node-00276 - Lat	1.000154	Node-00276 - Lat	1.00032	Node-00276 - Lat	1.00032
Node-00277 - Long	1.00033	Node-00277 - Long	1.001396	Node-00277 - Long	1.001396
Node-00277 - Lat	1.000104	Node-00277 - Lat	1.000438	Node-00277 - Lat	1.000438
Node-00278 - Long	1.000648	Node-00278 - Long	1.001493	Node-00278 - Long	1.001493
Node-00278 - Lat	1.000019	Node-00278 - Lat	1.000431	Node-00278 - Lat	1.000431
Node-00279 - Long	NA	Node-00279 - Long	1.000418	Node-00279 - Long	1.000418
Node-00279 - Lat	NA	Node-00279 - Lat	1.000431	Node-00279 - Lat	1.000431
Node-00280 - Long	NA	Node-00280 - Long	NA	Node-00280 - Long	NA
Node-00280 - Lat	NA	Node-00280 - Lat	NA	Node-00280 - Lat	NA
Node-00281 - Long	NA	Node-00281 - Long	NA	Node-00281 - Long	NA
Node-00281 - Lat	NA	Node-00281 - Lat	NA	Node-00281 - Lat	NA
Node-00282 - Long	NA	Node-00282 - Long	NA	Node-00282 - Long	NA
Node-00282 - Lat	NA	Node-00282 - Lat	NA	Node-00282 - Lat	NA
Node-00283 - Long	1.000035	Node-00283 - Long	NA	Node-00283 - Long	NA
Node-00283 - Lat	1.000063	Node-00283 - Lat	NA	Node-00283 - Lat	NA
Node-00284 - Long	NA	Node-00284 - Long	1.003005	Node-00284 - Long	1.003005

Node-00284 - Lat	NA	Node-00284 - Lat	1.000661	Node-00284 - Lat	1.000661
Node-00285 - Long	NA	Node-00285 - Long	NA	Node-00285 - Long	NA
Node-00285 - Lat	NA	Node-00285 - Lat	NA	Node-00285 - Lat	NA
Node-00286 - Long	NA	Node-00286 - Long	NA	Node-00286 - Long	NA
Node-00286 - Lat	NA	Node-00286 - Lat	NA	Node-00286 - Lat	NA
Node-00287 - Long	1.069555	Node-00287 - Long	NA	Node-00287 - Long	NA
Node-00287 - Lat	1.065165	Node-00287 - Lat	NA	Node-00287 - Lat	NA
Node-00288 - Long	1.008159	Node-00288 - Long	1.074722	Node-00288 - Long	1.074722
Node-00288 - Lat	1.008625	Node-00288 - Lat	1.168289	Node-00288 - Lat	1.168289
Node-00289 - Long	1.000453	Node-00289 - Long	1.003022	Node-00289 - Long	1.003022
Node-00289 - Lat	1.000314	Node-00289 - Lat	1.000514	Node-00289 - Lat	1.000514
Node-00290 - Long	1.000228	Node-00290 - Long	1.000441	Node-00290 - Long	1.000441
Node-00290 - Lat	1.000275	Node-00290 - Lat	1.000354	Node-00290 - Lat	1.000354
Node-00291 - Long	1.000139	Node-00291 - Long	1.000293	Node-00291 - Long	1.000293
Node-00291 - Lat	1.000299	Node-00291 - Lat	1.000252	Node-00291 - Lat	1.000252
Node-00292 - Long	NA	Node-00292 - Long	1.000191	Node-00292 - Long	1.000191
Node-00292 - Lat	NA	Node-00292 - Lat	1.000256	Node-00292 - Lat	1.000256
Node-00293 - Long	1.000204	Node-00293 - Long	NA	Node-00293 - Long	NA
Node-00293 - Lat	1.000211	Node-00293 - Lat	NA	Node-00293 - Lat	NA
Node-00294 - Long	1.000201	Node-00294 - Long	1.000035	Node-00294 - Long	1.000035
Node-00294 - Lat	NA	Node-00294 - Lat	1.000016	Node-00294 - Lat	1.000016
Node-00295 - Long	1.000588	Node-00295 - Long	1.000043	Node-00295 - Long	1.000043
Node-00295 - Lat	1.000281	Node-00295 - Lat	NA	Node-00295 - Lat	NA
Node-00296 - Long	NA	Node-00296 - Long	1.000356	Node-00296 - Long	1.000356
Node-00296 - Lat	NA	Node-00296 - Lat	1.000132	Node-00296 - Lat	1.000132
Node-00297 - Long	1.000565	Node-00297 - Long	NA	Node-00297 - Long	NA
Node-00297 - Lat	1.000306	Node-00297 - Lat	NA	Node-00297 - Lat	NA
Node-00298 - Long	1.000607	Node-00298 - Long	1.000262	Node-00298 - Long	1.000262
Node-00298 - Lat	1.000607	Node-00298 - Lat	1.000162	Node-00298 - Lat	1.000162
Node-00299 - Long	NA	Node-00299 - Long	1.000287	Node-00299 - Long	1.000287
Node-00299 - Lat	NA	Node-00299 - Lat	1.000287	Node-00299 - Lat	1.000287
Node-00300 - Long	1.279321	Node-00300 - Long	NA	Node-00300 - Long	NA
Node-00300 - Lat	1.26031	Node-00300 - Lat	NA	Node-00300 - Lat	NA
Node-00301 - Long	1.010356	Node-00301 - Long	1.246674	Node-00301 - Long	1.246674
Node-00301 - Lat	1.00999	Node-00301 - Lat	1.267002	Node-00301 - Lat	1.267002
Node-00302 - Long	1.012582	Node-00302 - Long	1.000092	Node-00302 - Long	1.000092
Node-00302 - Lat	1.013647	Node-00302 - Lat	1.000034	Node-00302 - Lat	1.000034
Node-00303 - Long	1.008246	Node-00303 - Long	1.000031	Node-00303 - Long	1.000031
Node-00303 - Lat	1.009813	Node-00303 - Lat	1.000029	Node-00303 - Lat	1.000029
Node-00304 - Long	NA	Node-00304 - Long	1.000067	Node-00304 - Long	1.000067
Node-00304 - Lat	NA	Node-00304 - Lat	1.00007	Node-00304 - Lat	1.00007
Node-00305 - Long	1.713473	Node-00305 - Long	NA	Node-00305 - Long	NA
Node-00305 - Lat	1.234639	Node-00305 - Lat	NA	Node-00305 - Lat	NA
Node-00306 - Long	NA	Node-00306 - Long	1.603105	Node-00306 - Long	1.603105
Node-00306 - Lat	NA	Node-00306 - Lat	2.466189	Node-00306 - Lat	2.466189
Node-00307 - Long	1.748732	Node-00307 - Long	NA	Node-00307 - Long	NA
Node-00307 - Lat	1.467726	Node-00307 - Lat	NA	Node-00307 - Lat	NA
Node-00308 - Long	1.9282	Node-00308 - Long	2.41735	Node-00308 - Long	2.41735

Node-00308 - Lat	1.57442	Node-00308 - Lat	2.269074	Node-00308 - Lat	2.269074
Node-00309 - Long	NA	Node-00309 - Long	2.884728	Node-00309 - Long	2.884728
Node-00309 - Lat	NA	Node-00309 - Lat	1.128565	Node-00309 - Lat	1.128565
Node-00310 - Long	NA	Node-00310 - Long	NA	Node-00310 - Long	NA
Node-00310 - Lat	NA	Node-00310 - Lat	NA	Node-00310 - Lat	NA
Node-00311 - Long	NA	Node-00311 - Long	NA	Node-00311 - Long	NA
Node-00311 - Lat	NA	Node-00311 - Lat	NA	Node-00311 - Lat	NA
Node-00312 - Long	1.049441	Node-00312 - Long	NA	Node-00312 - Long	NA
Node-00312 - Lat	1.045487	Node-00312 - Lat	NA	Node-00312 - Lat	NA
Node-00313 - Long	10.56107	Node-00313 - Long	1.000058	Node-00313 - Long	1.000058
Node-00313 - Lat	37.09408	Node-00313 - Lat	0.999996	Node-00313 - Lat	0.999996
Node-00314 - Long	NA	Node-00314 - Long	0.999986	Node-00314 - Long	0.999986
Node-00314 - Lat	NA	Node-00314 - Lat	1.00006	Node-00314 - Lat	1.00006
Node-00315 - Long	1.020395	Node-00315 - Long	NA	Node-00315 - Long	NA
Node-00315 - Lat	1.025021	Node-00315 - Lat	NA	Node-00315 - Lat	NA
Node-00316 - Long	NA	Node-00316 - Long	1.000063	Node-00316 - Long	1.000063
Node-00316 - Lat	NA	Node-00316 - Lat	1.000013	Node-00316 - Lat	1.000013
Node-00317 - Long	NA	Node-00317 - Long	NA	Node-00317 - Long	NA
Node-00317 - Lat	NA	Node-00317 - Lat	NA	Node-00317 - Lat	NA
Node-00318 - Long	NA	Node-00318 - Long	NA	Node-00318 - Long	NA
Node-00318 - Lat	NA	Node-00318 - Lat	NA	Node-00318 - Lat	NA
Node-00319 - Long	NA	Node-00319 - Long	NA	Node-00319 - Long	NA
Node-00319 - Lat	NA	Node-00319 - Lat	NA	Node-00319 - Lat	NA
Node-00320 - Long	1.263774	Node-00320 - Long	NA	Node-00320 - Long	NA
Node-00320 - Lat	1.254269	Node-00320 - Lat	NA	Node-00320 - Lat	NA
Node-00321 - Long	NA	Node-00321 - Long	1.265915	Node-00321 - Long	1.265915
Node-00321 - Lat	NA	Node-00321 - Lat	1.25378	Node-00321 - Lat	1.25378
Node-00322 - Long	NA	Node-00322 - Long	NA	Node-00322 - Long	NA
Node-00322 - Lat	NA	Node-00322 - Lat	NA	Node-00322 - Lat	NA
Node-00323 - Long	NA	Node-00323 - Long	NA	Node-00323 - Long	NA
Node-00323 - Lat	NA	Node-00323 - Lat	NA	Node-00323 - Lat	NA
Node-00324 - Long	NA	Node-00324 - Long	NA	Node-00324 - Long	NA
Node-00324 - Lat	NA	Node-00324 - Lat	NA	Node-00324 - Lat	NA
Node-00325 - Long	1.003398	Node-00325 - Long	NA	Node-00325 - Long	NA
Node-00325 - Lat	1.0007	Node-00325 - Lat	NA	Node-00325 - Lat	NA
Node-00326 - Long	1.004367	Node-00326 - Long	1.006143	Node-00326 - Long	1.006143
Node-00326 - Lat	1.000542	Node-00326 - Lat	1.002221	Node-00326 - Lat	1.002221
Node-00327 - Long	1.002317	Node-00327 - Long	1.005899	Node-00327 - Long	1.005899
Node-00327 - Lat	1.000436	Node-00327 - Lat	1.001531	Node-00327 - Lat	1.001531
Node-00328 - Long	NA	Node-00328 - Long	1.002698	Node-00328 - Long	1.002698
Node-00328 - Lat	NA	Node-00328 - Lat	1.00011	Node-00328 - Lat	1.00011
Node-00329 - Long	NA	Node-00329 - Long	NA	Node-00329 - Long	NA
Node-00329 - Lat	NA	Node-00329 - Lat	NA	Node-00329 - Lat	NA
Node-00330 - Long	NA	Node-00330 - Long	NA	Node-00330 - Long	NA
Node-00330 - Lat	NA	Node-00330 - Lat	NA	Node-00330 - Lat	NA
Node-00331 - Long	NA	Node-00331 - Long	NA	Node-00331 - Long	NA
Node-00331 - Lat	NA	Node-00331 - Lat	NA	Node-00331 - Lat	NA
Node-00332 - Long	NA	Node-00332 - Long	NA	Node-00332 - Long	NA

Node-00332 - Lat	NA	Node-00332 - Lat	NA	Node-00332 - Lat	NA
Node-00333 - Long	1.013884	Node-00333 - Long	NA	Node-00333 - Long	NA
Node-00333 - Lat	1.014423	Node-00333 - Lat	NA	Node-00333 - Lat	NA
Node-00334 - Long	NA	Node-00334 - Long	1.021231	Node-00334 - Long	1.021231
Node-00334 - Lat	NA	Node-00334 - Lat	1.023964	Node-00334 - Lat	1.023964
Node-00335 - Long	1.005558	Node-00335 - Long	NA	Node-00335 - Long	NA
Node-00335 - Lat	1.004371	Node-00335 - Lat	NA	Node-00335 - Lat	NA
Node-00336 - Long	NA	Node-00336 - Long	1.007016	Node-00336 - Long	1.007016
Node-00336 - Lat	NA	Node-00336 - Lat	1.005376	Node-00336 - Lat	1.005376
Node-00337 - Long	NA	Node-00337 - Long	NA	Node-00337 - Long	NA
Node-00337 - Lat	NA	Node-00337 - Lat	NA	Node-00337 - Lat	NA
Node-00338 - Long	2.277714	Node-00338 - Long	NA	Node-00338 - Long	NA
Node-00338 - Lat	8.23973	Node-00338 - Lat	NA	Node-00338 - Lat	NA
Node-00339 - Long	2.030434	Node-00339 - Long	2.022961	Node-00339 - Long	2.022961
Node-00339 - Lat	8.812902	Node-00339 - Lat	3.352257	Node-00339 - Lat	3.352257
Node-00340 - Long	1.912499	Node-00340 - Long	1.882385	Node-00340 - Long	1.882385
Node-00340 - Lat	10.27822	Node-00340 - Lat	3.401258	Node-00340 - Lat	3.401258
Node-00341 - Long	1.772907	Node-00341 - Long	1.825279	Node-00341 - Long	1.825279
Node-00341 - Lat	9.583064	Node-00341 - Lat	3.641844	Node-00341 - Lat	3.641844
Node-00342 - Long	2.993024	Node-00342 - Long	1.643459	Node-00342 - Long	1.643459
Node-00342 - Lat	4.304872	Node-00342 - Lat	3.587225	Node-00342 - Lat	3.587225
Node-00343 - Long	3.526792	Node-00343 - Long	2.410203	Node-00343 - Long	2.410203
Node-00343 - Lat	4.669966	Node-00343 - Lat	3.761494	Node-00343 - Lat	3.761494
Node-00344 - Long	5.492031	Node-00344 - Long	3.079955	Node-00344 - Long	3.079955
Node-00344 - Lat	9.694958	Node-00344 - Lat	4.223526	Node-00344 - Lat	4.223526
Node-00345 - Long	6.37567	Node-00345 - Long	4.888922	Node-00345 - Long	4.888922
Node-00345 - Lat	10.81411	Node-00345 - Lat	8.109307	Node-00345 - Lat	8.109307
Node-00346 - Long	7.056052	Node-00346 - Long	5.598804	Node-00346 - Long	5.598804
Node-00346 - Lat	12.04743	Node-00346 - Lat	8.901412	Node-00346 - Lat	8.901412
Node-00347 - Long	6.632179	Node-00347 - Long	6.491739	Node-00347 - Long	6.491739
Node-00347 - Lat	11.79345	Node-00347 - Lat	10.34271	Node-00347 - Lat	10.34271
Node-00348 - Long	1.291298	Node-00348 - Long	6.335372	Node-00348 - Long	6.335372
Node-00348 - Lat	2.351725	Node-00348 - Lat	9.741025	Node-00348 - Lat	9.741025
Node-00349 - Long	1.002484	Node-00349 - Long	1.138346	Node-00349 - Long	1.138346
Node-00349 - Lat	1.000409	Node-00349 - Lat	1.39389	Node-00349 - Lat	1.39389
Node-00350 - Long	1.002878	Node-00350 - Long	1.002452	Node-00350 - Long	1.002452
Node-00350 - Lat	1.000212	Node-00350 - Lat	1.000281	Node-00350 - Lat	1.000281
Node-00351 - Long	1.002362	Node-00351 - Long	1.002629	Node-00351 - Long	1.002629
Node-00351 - Lat	1.00018	Node-00351 - Lat	1.0004	Node-00351 - Lat	1.0004
Node-00352 - Long	NA	Node-00352 - Long	1.002157	Node-00352 - Long	1.002157
Node-00352 - Lat	NA	Node-00352 - Lat	1.00039	Node-00352 - Lat	1.00039
Node-00353 - Long	1.002797	Node-00353 - Long	NA	Node-00353 - Long	NA
Node-00353 - Lat	1.000076	Node-00353 - Lat	NA	Node-00353 - Lat	NA
Node-00354 - Long	1.003195	Node-00354 - Long	1.002142	Node-00354 - Long	1.002142
Node-00354 - Lat	1.000269	Node-00354 - Lat	1.000253	Node-00354 - Lat	1.000253
Node-00355 - Long	1.001922	Node-00355 - Long	1.00212	Node-00355 - Long	1.00212
Node-00355 - Lat	1.000796	Node-00355 - Lat	1.000455	Node-00355 - Lat	1.000455
Node-00356 - Long	1.002033	Node-00356 - Long	1.001246	Node-00356 - Long	1.001246

Node-00356 - Lat	1.002033	Node-00356 - Lat	1.00027	Node-00356 - Lat	1.00027
Node-00357 - Long	NA	Node-00357 - Long	1.00281	Node-00357 - Long	1.00281
Node-00357 - Lat	NA	Node-00357 - Lat	1.00281	Node-00357 - Lat	1.00281
Node-00358 - Long	1.001681	Node-00358 - Long	NA	Node-00358 - Long	NA
Node-00358 - Lat	1.001681	Node-00358 - Lat	NA	Node-00358 - Lat	NA
Node-00359 - Long	NA	Node-00359 - Long	1.002239	Node-00359 - Long	1.002239
Node-00359 - Lat	NA	Node-00359 - Lat	1.002239	Node-00359 - Lat	1.002239
Node-00360 - Long	2.531326	Node-00360 - Long	NA	Node-00360 - Long	NA
Node-00360 - Lat	3.075747	Node-00360 - Lat	NA	Node-00360 - Lat	NA
Node-00361 - Long	1.812298	Node-00361 - Long	1.494548	Node-00361 - Long	1.494548
Node-00361 - Lat	1.245902	Node-00361 - Lat	1.906932	Node-00361 - Lat	1.906932
Node-00362 - Long	NA	Node-00362 - Long	1.660888	Node-00362 - Long	1.660888
Node-00362 - Lat	NA	Node-00362 - Lat	1.803487	Node-00362 - Lat	1.803487
Node-00363 - Long	NA	Node-00363 - Long	NA	Node-00363 - Long	NA
Node-00363 - Lat	NA	Node-00363 - Lat	NA	Node-00363 - Lat	NA
Node-00364 - Long	NA	Node-00364 - Long	NA	Node-00364 - Long	NA
Node-00364 - Lat	NA	Node-00364 - Lat	NA	Node-00364 - Lat	NA
Node-00365 - Long	NA	Node-00365 - Long	NA	Node-00365 - Long	NA
Node-00365 - Lat	NA	Node-00365 - Lat	NA	Node-00365 - Lat	NA
Node-00366 - Long	6.791263	Node-00366 - Long	NA	Node-00366 - Long	NA
Node-00366 - Lat	10.99173	Node-00366 - Lat	NA	Node-00366 - Lat	NA
Node-00367 - Long	NA	Node-00367 - Long	5.989649	Node-00367 - Long	5.989649
Node-00367 - Lat	NA	Node-00367 - Lat	8.985907	Node-00367 - Lat	8.985907
Node-00368 - Long	1.198023	Node-00368 - Long	NA	Node-00368 - Long	NA
Node-00368 - Lat	1.186793	Node-00368 - Lat	NA	Node-00368 - Lat	NA
Node-00369 - Long	NA	Node-00369 - Long	1.343335	Node-00369 - Long	1.343335
Node-00369 - Lat	NA	Node-00369 - Lat	1.284535	Node-00369 - Lat	1.284535
Node-00370 - Long	1.082115	Node-00370 - Long	NA	Node-00370 - Long	NA
Node-00370 - Lat	1.01439	Node-00370 - Lat	NA	Node-00370 - Lat	NA
Node-00371 - Long	1.053843	Node-00371 - Long	1.080161	Node-00371 - Long	1.080161
Node-00371 - Lat	1.002603	Node-00371 - Lat	1.021144	Node-00371 - Lat	1.021144
Node-00372 - Long	1.001697	Node-00372 - Long	1.092795	Node-00372 - Long	1.092795
Node-00372 - Lat	1.001485	Node-00372 - Lat	1.015303	Node-00372 - Lat	1.015303
Node-00373 - Long	1.000122	Node-00373 - Long	1.001396	Node-00373 - Long	1.001396
Node-00373 - Lat	1.000116	Node-00373 - Lat	1.000731	Node-00373 - Lat	1.000731
Node-00374 - Long	1.000143	Node-00374 - Long	0.999983	Node-00374 - Long	0.999983
Node-00374 - Lat	1.00015	Node-00374 - Lat	0.999982	Node-00374 - Lat	0.999982
Node-00375 - Long	1.146698	Node-00375 - Long	0.999993	Node-00375 - Long	0.999993
Node-00375 - Lat	1.004728	Node-00375 - Lat	0.999999	Node-00375 - Lat	0.999999
Node-00376 - Long	NA	Node-00376 - Long	1.707876	Node-00376 - Long	1.707876
Node-00376 - Lat	NA	Node-00376 - Lat	1.012952	Node-00376 - Lat	1.012952
Node-00377 - Long	2.642437	Node-00377 - Long	NA	Node-00377 - Long	NA
Node-00377 - Lat	1.319742	Node-00377 - Lat	NA	Node-00377 - Lat	NA
Node-00378 - Long	1.078928	Node-00378 - Long	5658364	Node-00378 - Long	5658364
Node-00378 - Lat	1.001552	Node-00378 - Lat	200263.7	Node-00378 - Lat	200263.7
Node-00379 - Long	1.03175	Node-00379 - Long	1.530274	Node-00379 - Long	1.530274
Node-00379 - Lat	1.000242	Node-00379 - Lat	1.005203	Node-00379 - Lat	1.005203
Node-00380 - Long	NA	Node-00380 - Long	1.21802	Node-00380 - Long	1.21802

Node-00380 - Lat	NA	Node-00380 - Lat	1.002493	Node-00380 - Lat	1.002493
Node-00381 - Long	1.011842	Node-00381 - Long	NA	Node-00381 - Long	NA
Node-00381 - Lat	1.000799	Node-00381 - Lat	NA	Node-00381 - Lat	NA
Node-00382 - Long	NA	Node-00382 - Long	1.0704	Node-00382 - Long	1.0704
Node-00382 - Lat	NA	Node-00382 - Lat	1.005442	Node-00382 - Lat	1.005442
Node-00383 - Long	12427.59	Node-00383 - Long	NA	Node-00383 - Long	NA
Node-00383 - Lat	1392.834	Node-00383 - Lat	NA	Node-00383 - Lat	NA
Node-00384 - Long	24901.4	Node-00384 - Long	7209149	Node-00384 - Long	7209149
Node-00384 - Lat	2177.183	Node-00384 - Lat	423555.7	Node-00384 - Lat	423555.7
Node-00385 - Long	Inf	Node-00385 - Long	10386746	Node-00385 - Long	10386746
Node-00385 - Lat	Inf	Node-00385 - Lat	408471.3	Node-00385 - Lat	408471.3
Node-00386 - Long	Inf	Node-00386 - Long	Inf	Node-00386 - Long	Inf
Node-00386 - Lat	Inf	Node-00386 - Lat	Inf	Node-00386 - Lat	Inf
Node-00387 - Long	2.075552	Node-00387 - Long	Inf	Node-00387 - Long	Inf
Node-00387 - Lat	1.663777	Node-00387 - Lat	Inf	Node-00387 - Lat	Inf
Node-00388 - Long	1.019756	Node-00388 - Long	Inf	Node-00388 - Long	Inf
Node-00388 - Lat	1.020411	Node-00388 - Lat	Inf	Node-00388 - Lat	Inf
Node-00389 - Long	NA	Node-00389 - Long	1.01769	Node-00389 - Long	1.01769
Node-00389 - Lat	NA	Node-00389 - Lat	1.017096	Node-00389 - Lat	1.017096
Node-00390 - Long	1.025813	Node-00390 - Long	NA	Node-00390 - Long	NA
Node-00390 - Lat	1.008753	Node-00390 - Lat	NA	Node-00390 - Lat	NA
Node-00391 - Long	1.156822	Node-00391 - Long	1.017387	Node-00391 - Long	1.017387
Node-00391 - Lat	1.00735	Node-00391 - Lat	1.00534	Node-00391 - Lat	1.00534
Node-00392 - Long	1.674532	Node-00392 - Long	1.10145	Node-00392 - Long	1.10145
Node-00392 - Lat	10.05094	Node-00392 - Lat	1.008866	Node-00392 - Lat	1.008866
Node-00393 - Long	1.57969	Node-00393 - Long	1.617761	Node-00393 - Long	1.617761
Node-00393 - Lat	8.810091	Node-00393 - Lat	3.593938	Node-00393 - Lat	3.593938
Node-00394 - Long	1.766794	Node-00394 - Long	1.579881	Node-00394 - Long	1.579881
Node-00394 - Lat	7.103498	Node-00394 - Lat	3.550747	Node-00394 - Lat	3.550747
Node-00395 - Long	NA	Node-00395 - Long	1.656563	Node-00395 - Long	1.656563
Node-00395 - Lat	NA	Node-00395 - Lat	3.118858	Node-00395 - Lat	3.118858
Node-00396 - Long	1.010601	Node-00396 - Long	NA	Node-00396 - Long	NA
Node-00396 - Lat	1.009065	Node-00396 - Lat	NA	Node-00396 - Lat	NA
Node-00397 - Long	NA	Node-00397 - Long	1.005732	Node-00397 - Long	1.005732
Node-00397 - Lat	NA	Node-00397 - Lat	1.005669	Node-00397 - Lat	1.005669
Node-00398 - Long	NA	Node-00398 - Long	NA	Node-00398 - Long	NA
Node-00398 - Lat	NA	Node-00398 - Lat	NA	Node-00398 - Lat	NA
Node-00399 - Long	NA	Node-00399 - Long	NA	Node-00399 - Long	NA
Node-00399 - Lat	NA	Node-00399 - Lat	NA	Node-00399 - Lat	NA
Node-00400 - Long	NA	Node-00400 - Long	NA	Node-00400 - Long	NA
Node-00400 - Lat	NA	Node-00400 - Lat	NA	Node-00400 - Lat	NA
Node-00401 - Long	1.505646	Node-00401 - Long	NA	Node-00401 - Long	NA
Node-00401 - Lat	9.751827	Node-00401 - Lat	NA	Node-00401 - Lat	NA
Node-00402 - Long	1.363158	Node-00402 - Long	1.487614	Node-00402 - Long	1.487614
Node-00402 - Lat	2.707602	Node-00402 - Lat	3.531082	Node-00402 - Lat	3.531082
Node-00403 - Long	1.029102	Node-00403 - Long	1.278764	Node-00403 - Long	1.278764
Node-00403 - Lat	1.016031	Node-00403 - Lat	1.551413	Node-00403 - Lat	1.551413
Node-00404 - Long	1.014155	Node-00404 - Long	1.015032	Node-00404 - Long	1.015032

Node-00404 - Lat	1.008525	Node-00404 - Lat	1.007576	Node-00404 - Lat	1.007576
Node-00405 - Long	NA	Node-00405 - Long	1.008423	Node-00405 - Long	1.008423
Node-00405 - Lat	NA	Node-00405 - Lat	1.004684	Node-00405 - Lat	1.004684
Node-00406 - Long	NA	Node-00406 - Long	NA	Node-00406 - Long	NA
Node-00406 - Lat	NA	Node-00406 - Lat	NA	Node-00406 - Lat	NA
Node-00407 - Long	NA	Node-00407 - Long	NA	Node-00407 - Long	NA
Node-00407 - Lat	NA	Node-00407 - Lat	NA	Node-00407 - Lat	NA
Node-00408 - Long	1.029229	Node-00408 - Long	NA	Node-00408 - Long	NA
Node-00408 - Lat	2.646854	Node-00408 - Lat	NA	Node-00408 - Lat	NA
Node-00409 - Long	NA	Node-00409 - Long	1.037793	Node-00409 - Long	1.037793
Node-00409 - Lat	NA	Node-00409 - Lat	1.531535	Node-00409 - Lat	1.531535
Node-00410 - Long	NA	Node-00410 - Long	NA	Node-00410 - Long	NA
Node-00410 - Lat	NA	Node-00410 - Lat	NA	Node-00410 - Lat	NA
Node-00411 - Long	NA	Node-00411 - Long	NA	Node-00411 - Long	NA
Node-00411 - Lat	NA	Node-00411 - Lat	NA	Node-00411 - Lat	NA
Node-00412 - Long	1.547934	Node-00412 - Long	NA	Node-00412 - Long	NA
Node-00412 - Lat	7.97783	Node-00412 - Lat	NA	Node-00412 - Lat	NA
Node-00413 - Long	1.242635	Node-00413 - Long	1.52923	Node-00413 - Long	1.52923
Node-00413 - Lat	7.291134	Node-00413 - Lat	3.460609	Node-00413 - Lat	3.460609
Node-00414 - Long	1.053141	Node-00414 - Long	1.260143	Node-00414 - Long	1.260143
Node-00414 - Lat	6.825882	Node-00414 - Lat	3.368536	Node-00414 - Lat	3.368536
Node-00415 - Long	1.008452	Node-00415 - Long	1.074417	Node-00415 - Long	1.074417
Node-00415 - Lat	6.867037	Node-00415 - Lat	3.312024	Node-00415 - Lat	3.312024
Node-00416 - Long	1.230738	Node-00416 - Long	1.009624	Node-00416 - Long	1.009624
Node-00416 - Lat	1.22152	Node-00416 - Lat	3.275423	Node-00416 - Lat	3.275423
Node-00417 - Long	1.000365	Node-00417 - Long	1.28547	Node-00417 - Long	1.28547
Node-00417 - Lat	1.000387	Node-00417 - Lat	1.289767	Node-00417 - Lat	1.289767
Node-00418 - Long	1.001212	Node-00418 - Long	1.000132	Node-00418 - Long	1.000132
Node-00418 - Lat	1.000924	Node-00418 - Lat	1.000171	Node-00418 - Lat	1.000171
Node-00419 - Long	1.001849	Node-00419 - Long	1.000467	Node-00419 - Long	1.000467
Node-00419 - Lat	1.001462	Node-00419 - Lat	1.00039	Node-00419 - Lat	1.00039
Node-00420 - Long	1.001039	Node-00420 - Long	1.001339	Node-00420 - Long	1.001339
Node-00420 - Lat	1.001842	Node-00420 - Lat	1.000217	Node-00420 - Lat	1.000217
Node-00421 - Long	1.000649	Node-00421 - Long	1.001262	Node-00421 - Long	1.001262
Node-00421 - Lat	1.002352	Node-00421 - Lat	1.000326	Node-00421 - Lat	1.000326
Node-00422 - Long	1.000387	Node-00422 - Long	1.000732	Node-00422 - Long	1.000732
Node-00422 - Lat	1.002105	Node-00422 - Lat	1.000374	Node-00422 - Lat	1.000374
Node-00423 - Long	NA	Node-00423 - Long	1.000111	Node-00423 - Long	1.000111
Node-00423 - Lat	NA	Node-00423 - Lat	1.000409	Node-00423 - Lat	1.000409
Node-00424 - Long	1.000465	Node-00424 - Long	NA	Node-00424 - Long	NA
Node-00424 - Lat	1.000871	Node-00424 - Lat	NA	Node-00424 - Lat	NA
Node-00425 - Long	NA	Node-00425 - Long	1.000054	Node-00425 - Long	1.000054
Node-00425 - Lat	NA	Node-00425 - Lat	1.000123	Node-00425 - Lat	1.000123
Node-00426 - Long	NA	Node-00426 - Long	NA	Node-00426 - Long	NA
Node-00426 - Lat	NA	Node-00426 - Lat	NA	Node-00426 - Lat	NA
Node-00427 - Long	1.00045	Node-00427 - Long	NA	Node-00427 - Long	NA
Node-00427 - Lat	1.002272	Node-00427 - Lat	NA	Node-00427 - Lat	NA
Node-00428 - Long	1.000121	Node-00428 - Long	1.000485	Node-00428 - Long	1.000485

Node-00428 - Lat	1.001022	Node-00428 - Lat	1.000542	Node-00428 - Lat	1.000542
Node-00429 - Long	1.000691	Node-00429 - Long	1.000224	Node-00429 - Long	1.000224
Node-00429 - Lat	1.000889	Node-00429 - Lat	1.000775	Node-00429 - Lat	1.000775
Node-00430 - Long	1.000346	Node-00430 - Long	1.000168	Node-00430 - Long	1.000168
Node-00430 - Lat	1.001141	Node-00430 - Lat	1.000153	Node-00430 - Lat	1.000153
Node-00431 - Long	NA	Node-00431 - Long	1.00016	Node-00431 - Long	1.00016
Node-00431 - Lat	NA	Node-00431 - Lat	1.00005	Node-00431 - Lat	1.00005
Node-00432 - Long	NA	Node-00432 - Long	NA	Node-00432 - Long	NA
Node-00432 - Lat	NA	Node-00432 - Lat	NA	Node-00432 - Lat	NA
Node-00433 - Long	NA	Node-00433 - Long	NA	Node-00433 - Long	NA
Node-00433 - Lat	NA	Node-00433 - Lat	NA	Node-00433 - Lat	NA
Node-00434 - Long	1.000119	Node-00434 - Long	NA	Node-00434 - Long	NA
Node-00434 - Lat	1.000931	Node-00434 - Lat	NA	Node-00434 - Lat	NA
Node-00435 - Long	1.000234	Node-00435 - Long	1.000155	Node-00435 - Long	1.000155
Node-00435 - Lat	1.000936	Node-00435 - Lat	1.000825	Node-00435 - Lat	1.000825
Node-00436 - Long	1.000226	Node-00436 - Long	1.000228	Node-00436 - Long	1.000228
Node-00436 - Lat	1.001031	Node-00436 - Lat	1.000812	Node-00436 - Lat	1.000812
Node-00437 - Long	1.000114	Node-00437 - Long	1.000071	Node-00437 - Long	1.000071
Node-00437 - Lat	1.000922	Node-00437 - Lat	1.000818	Node-00437 - Lat	1.000818
Node-00438 - Long	1.000233	Node-00438 - Long	1.000008	Node-00438 - Long	1.000008
Node-00438 - Lat	1.00096	Node-00438 - Lat	1.000638	Node-00438 - Lat	1.000638
Node-00439 - Long	1.000318	Node-00439 - Long	1.000074	Node-00439 - Long	1.000074
Node-00439 - Lat	1.000995	Node-00439 - Lat	1.000631	Node-00439 - Lat	1.000631
Node-00440 - Long	1.00051	Node-00440 - Long	1.000058	Node-00440 - Long	1.000058
Node-00440 - Lat	1.003915	Node-00440 - Lat	1.000677	Node-00440 - Lat	1.000677
Node-00441 - Long	1.001065	Node-00441 - Long	1.000405	Node-00441 - Long	1.000405
Node-00441 - Lat	1.005253	Node-00441 - Lat	1.002701	Node-00441 - Lat	1.002701
Node-00442 - Long	1.003934	Node-00442 - Long	1.00042	Node-00442 - Long	1.00042
Node-00442 - Lat	1.007017	Node-00442 - Lat	1.002678	Node-00442 - Lat	1.002678
Node-00443 - Long	1.007667	Node-00443 - Long	1.00141	Node-00443 - Long	1.00141
Node-00443 - Lat	1.008554	Node-00443 - Lat	1.003428	Node-00443 - Lat	1.003428
Node-00444 - Long	1.008108	Node-00444 - Long	1.002996	Node-00444 - Long	1.002996
Node-00444 - Lat	1.009366	Node-00444 - Lat	1.00445	Node-00444 - Lat	1.00445
Node-00445 - Long	1.008889	Node-00445 - Long	1.002567	Node-00445 - Long	1.002567
Node-00445 - Lat	1.00999	Node-00445 - Lat	1.004689	Node-00445 - Lat	1.004689
Node-00446 - Long	1.01394	Node-00446 - Long	1.002769	Node-00446 - Long	1.002769
Node-00446 - Lat	1.01738	Node-00446 - Lat	1.005136	Node-00446 - Lat	1.005136
Node-00447 - Long	1.001803	Node-00447 - Long	1.006877	Node-00447 - Long	1.006877
Node-00447 - Lat	1.001399	Node-00447 - Lat	1.009904	Node-00447 - Lat	1.009904
Node-00448 - Long	NA	Node-00448 - Long	1.000276	Node-00448 - Long	1.000276
Node-00448 - Lat	NA	Node-00448 - Lat	1.000357	Node-00448 - Lat	1.000357
Node-00449 - Long	NA	Node-00449 - Long	NA	Node-00449 - Long	NA
Node-00449 - Lat	NA	Node-00449 - Lat	NA	Node-00449 - Lat	NA
Node-00450 - Long	1.000383	Node-00450 - Long	NA	Node-00450 - Long	NA
Node-00450 - Lat	1.000602	Node-00450 - Lat	NA	Node-00450 - Lat	NA
Node-00451 - Long	1.000405	Node-00451 - Long	1.000338	Node-00451 - Long	1.000338
Node-00451 - Lat	1.000677	Node-00451 - Lat	1.001016	Node-00451 - Lat	1.001016
Node-00452 - Long	NA	Node-00452 - Long	1.000733	Node-00452 - Long	1.000733

Node-00452 - Lat	NA	Node-00452 - Lat	1.000248	Node-00452 - Lat	1.000248
Node-00453 - Long	1.000284	Node-00453 - Long	NA	Node-00453 - Long	NA
Node-00453 - Lat	1.000607	Node-00453 - Lat	NA	Node-00453 - Lat	NA
Node-00454 - Long	NA	Node-00454 - Long	1.00082	Node-00454 - Long	1.00082
Node-00454 - Lat	NA	Node-00454 - Lat	1.000385	Node-00454 - Lat	1.000385
Node-00455 - Long	1.003529	Node-00455 - Long	NA	Node-00455 - Long	NA
Node-00455 - Lat	1.008125	Node-00455 - Lat	NA	Node-00455 - Lat	NA
Node-00456 - Long	1.006689	Node-00456 - Long	1.007228	Node-00456 - Long	1.007228
Node-00456 - Lat	1.008607	Node-00456 - Lat	1.022536	Node-00456 - Lat	1.022536
Node-00457 - Long	NA	Node-00457 - Long	1.017778	Node-00457 - Long	1.017778
Node-00457 - Lat	NA	Node-00457 - Lat	1.023466	Node-00457 - Lat	1.023466
Node-00458 - Long	1.000092	Node-00458 - Long	NA	Node-00458 - Long	NA
Node-00458 - Lat	1.000064	Node-00458 - Lat	NA	Node-00458 - Lat	NA
Node-00459 - Long	1.000432	Node-00459 - Long	1.000104	Node-00459 - Long	1.000104
Node-00459 - Lat	1.000432	Node-00459 - Lat	1.000038	Node-00459 - Lat	1.000038
Node-00460 - Long	NA	Node-00460 - Long	1.000351	Node-00460 - Long	1.000351
Node-00460 - Lat	NA	Node-00460 - Lat	1.000351	Node-00460 - Lat	1.000351
Node-00461 - Long	1.00913	Node-00461 - Long	NA	Node-00461 - Long	NA
Node-00461 - Lat	1.003826	Node-00461 - Lat	NA	Node-00461 - Lat	NA
Node-00462 - Long	NA	Node-00462 - Long	1.003964	Node-00462 - Long	1.003964
Node-00462 - Lat	NA	Node-00462 - Lat	1.000925	Node-00462 - Lat	1.000925
Node-00463 - Long	1.000263	Node-00463 - Long	NA	Node-00463 - Long	NA
Node-00463 - Lat	1.000082	Node-00463 - Lat	NA	Node-00463 - Lat	NA
Node-00464 - Long	1.000047	Node-00464 - Long	1.000249	Node-00464 - Long	1.000249
Node-00464 - Lat	1.000063	Node-00464 - Lat	1.000254	Node-00464 - Lat	1.000254
Node-00465 - Long	NA	Node-00465 - Long	1.000213	Node-00465 - Long	1.000213
Node-00465 - Lat	NA	Node-00465 - Lat	1.000337	Node-00465 - Lat	1.000337
Node-00466 - Long	1.000108	Node-00466 - Long	NA	Node-00466 - Long	NA
Node-00466 - Lat	1.000125	Node-00466 - Lat	NA	Node-00466 - Lat	NA
Node-00467 - Long	1.000043	Node-00467 - Long	1.000072	Node-00467 - Long	1.000072
Node-00467 - Lat	1.000025	Node-00467 - Lat	1.000053	Node-00467 - Lat	1.000053
Node-00468 - Long	NA	Node-00468 - Long	1.000141	Node-00468 - Long	1.000141
Node-00468 - Lat	NA	Node-00468 - Lat	1.000077	Node-00468 - Lat	1.000077
Node-00469 - Long	0.999996	Node-00469 - Long	NA	Node-00469 - Long	NA
Node-00469 - Lat	0.999996	Node-00469 - Lat	NA	Node-00469 - Lat	NA
Node-00470 - Long	NA	Node-00470 - Long	1.000225	Node-00470 - Long	1.000225
Node-00470 - Lat	NA	Node-00470 - Lat	1.000225	Node-00470 - Lat	1.000225
Node-00471 - Long	NA	Node-00471 - Long	NA	Node-00471 - Long	NA
Node-00471 - Lat	NA	Node-00471 - Lat	NA	Node-00471 - Lat	NA
Node-00472 - Long	1.005311	Node-00472 - Long	NA	Node-00472 - Long	NA
Node-00472 - Lat	1.005311	Node-00472 - Lat	NA	Node-00472 - Lat	NA
Node-00473 - Long	NA	Node-00473 - Long	1.002111	Node-00473 - Long	1.002111
Node-00473 - Lat	NA	Node-00473 - Lat	1.002111	Node-00473 - Lat	1.002111
Node-00474 - Long	1.000579	Node-00474 - Long	NA	Node-00474 - Long	NA
Node-00474 - Lat	1.002821	Node-00474 - Lat	NA	Node-00474 - Lat	NA
Node-00475 - Long	NA	Node-00475 - Long	1.000173	Node-00475 - Long	1.000173
Node-00475 - Lat	NA	Node-00475 - Lat	1.001424	Node-00475 - Lat	1.001424
Node-00476 - Long	NA	Node-00476 - Long	NA	Node-00476 - Long	NA

Node-00476 - Lat	NA	Node-00476 - Lat	NA	Node-00476 - Lat	NA
Node-00477 - Long	NA	Node-00477 - Long	NA	Node-00477 - Long	NA
Node-00477 - Lat	NA	Node-00477 - Lat	NA	Node-00477 - Lat	NA
Node-00478 - Long	NA	Node-00478 - Long	NA	Node-00478 - Long	NA
Node-00478 - Lat	NA	Node-00478 - Lat	NA	Node-00478 - Lat	NA
Node-00479 - Long	1.000024	Node-00479 - Long	NA	Node-00479 - Long	NA
Node-00479 - Lat	1.000141	Node-00479 - Lat	NA	Node-00479 - Lat	NA
Node-00480 - Long	NA	Node-00480 - Long	1.000004	Node-00480 - Long	1.000004
Node-00480 - Lat	NA	Node-00480 - Lat	1.000078	Node-00480 - Lat	1.000078
Node-00481 - Long	1.000053	Node-00481 - Long	NA	Node-00481 - Long	NA
Node-00481 - Lat	1.000053	Node-00481 - Lat	NA	Node-00481 - Lat	NA
Node-00482 - Long	1.000045	Node-00482 - Long	1.000001	Node-00482 - Long	1.000001
Node-00482 - Lat	1.000332	Node-00482 - Lat	1.000001	Node-00482 - Lat	1.000001
Node-00483 - Long	1.000092	Node-00483 - Long	1.000012	Node-00483 - Long	1.000012
Node-00483 - Lat	1.000233	Node-00483 - Lat	1.000262	Node-00483 - Lat	1.000262
Node-00484 - Long	NA	Node-00484 - Long	1.000011	Node-00484 - Long	1.000011
Node-00484 - Lat	NA	Node-00484 - Lat	1.000238	Node-00484 - Lat	1.000238
Node-00485 - Long	NA	Node-00485 - Long	NA	Node-00485 - Long	NA
Node-00485 - Lat	NA	Node-00485 - Lat	NA	Node-00485 - Lat	NA
Node-00486 - Long	1.000146	Node-00486 - Long	NA	Node-00486 - Long	NA
Node-00486 - Lat	1.000285	Node-00486 - Lat	NA	Node-00486 - Lat	NA
Node-00487 - Long	NA	Node-00487 - Long	1.000011	Node-00487 - Long	1.000011
Node-00487 - Lat	NA	Node-00487 - Lat	1.000235	Node-00487 - Lat	1.000235
Node-00488 - Long	NA	Node-00488 - Long	NA	Node-00488 - Long	NA
Node-00488 - Lat	NA	Node-00488 - Lat	NA	Node-00488 - Lat	NA
Node-00489 - Long	1.000114	Node-00489 - Long	NA	Node-00489 - Long	NA
Node-00489 - Lat	1.000372	Node-00489 - Lat	NA	Node-00489 - Lat	NA
Node-00490 - Long	1.000056	Node-00490 - Long	1.000151	Node-00490 - Long	1.000151
Node-00490 - Lat	1.00025	Node-00490 - Lat	1.000366	Node-00490 - Lat	1.000366
Node-00491 - Long	1.000044	Node-00491 - Long	1.000043	Node-00491 - Long	1.000043
Node-00491 - Lat	1.000067	Node-00491 - Lat	1.000137	Node-00491 - Lat	1.000137
Node-00492 - Long	NA	Node-00492 - Long	1.00004	Node-00492 - Long	1.00004
Node-00492 - Lat	NA	Node-00492 - Lat	0.999999	Node-00492 - Lat	0.999999
Node-00493 - Long	1.000026	Node-00493 - Long	NA	Node-00493 - Long	NA
Node-00493 - Lat	1.000099	Node-00493 - Lat	NA	Node-00493 - Lat	NA
Node-00494 - Long	1.000048	Node-00494 - Long	0.999984	Node-00494 - Long	0.999984
Node-00494 - Lat	1.000093	Node-00494 - Lat	0.999991	Node-00494 - Lat	0.999991
Node-00495 - Long	NA	Node-00495 - Long	0.99999	Node-00495 - Long	0.99999
Node-00495 - Lat	NA	Node-00495 - Lat	1.000282	Node-00495 - Lat	1.000282
Node-00496 - Long	1.000035	Node-00496 - Long	NA	Node-00496 - Long	NA
Node-00496 - Lat	1.000055	Node-00496 - Lat	NA	Node-00496 - Lat	NA
Node-00497 - Long	NA	Node-00497 - Long	1.000008	Node-00497 - Long	1.000008
Node-00497 - Lat	NA	Node-00497 - Lat	1.000048	Node-00497 - Lat	1.000048
Node-00498 - Long	NA	Node-00498 - Long	NA	Node-00498 - Long	NA
Node-00498 - Lat	NA	Node-00498 - Lat	NA	Node-00498 - Lat	NA
Node-00499 - Long	NA	Node-00499 - Long	NA	Node-00499 - Long	NA
Node-00499 - Lat	NA	Node-00499 - Lat	NA	Node-00499 - Lat	NA
Node-00500 - Long	NA	Node-00500 - Long	NA	Node-00500 - Long	NA

Node-00500 - Lat	NA	Node-00500 - Lat	NA	Node-00500 - Lat	NA
Node-00501 - Long	NA	Node-00501 - Long	NA	Node-00501 - Long	NA
Node-00501 - Lat	NA	Node-00501 - Lat	NA	Node-00501 - Lat	NA
Node-00502 - Long	1.000011	Node-00502 - Long	NA	Node-00502 - Long	NA
Node-00502 - Lat	1.000011	Node-00502 - Lat	NA	Node-00502 - Lat	NA
Node-00503 - Long	NA	Node-00503 - Long	1.000035	Node-00503 - Long	1.000035
Node-00503 - Lat	NA	Node-00503 - Lat	1.000035	Node-00503 - Lat	1.000035
Node-00504 - Long	NA	Node-00504 - Long	NA	Node-00504 - Long	NA
Node-00504 - Lat	NA	Node-00504 - Lat	NA	Node-00504 - Lat	NA
Node-00505 - Long	1.00018	Node-00505 - Long	NA	Node-00505 - Long	NA
Node-00505 - Lat	1.000576	Node-00505 - Lat	NA	Node-00505 - Lat	NA
Node-00506 - Long	NA	Node-00506 - Long	1.000049	Node-00506 - Long	1.000049
Node-00506 - Lat	NA	Node-00506 - Lat	1.000705	Node-00506 - Lat	1.000705
Node-00507 - Long	NA	Node-00507 - Long	NA	Node-00507 - Long	NA
Node-00507 - Lat	NA	Node-00507 - Lat	NA	Node-00507 - Lat	NA
Node-00508 - Long	1.21805	Node-00508 - Long	NA	Node-00508 - Long	NA
Node-00508 - Lat	1.218399	Node-00508 - Lat	NA	Node-00508 - Lat	NA
Node-00509 - Long	NA	Node-00509 - Long	1.288112	Node-00509 - Long	1.288112
Node-00509 - Lat	NA	Node-00509 - Lat	1.288391	Node-00509 - Lat	1.288391
Node-00510 - Long	NA	Node-00510 - Long	NA	Node-00510 - Long	NA
Node-00510 - Lat	NA	Node-00510 - Lat	NA	Node-00510 - Lat	NA
Node-00511 - Long	1.045483	Node-00511 - Long	NA	Node-00511 - Long	NA
Node-00511 - Lat	1.221468	Node-00511 - Lat	NA	Node-00511 - Lat	NA
Node-00512 - Long	1.029766	Node-00512 - Long	1.093633	Node-00512 - Long	1.093633
Node-00512 - Lat	1.155939	Node-00512 - Lat	1.438545	Node-00512 - Lat	1.438545
Node-00513 - Long	1.044302	Node-00513 - Long	1.059398	Node-00513 - Long	1.059398
Node-00513 - Lat	1.011841	Node-00513 - Lat	1.3147	Node-00513 - Lat	1.3147
Node-00514 - Long	0.999977	Node-00514 - Long	1.053192	Node-00514 - Long	1.053192
Node-00514 - Lat	0.999985	Node-00514 - Lat	1.014182	Node-00514 - Lat	1.014182
Node-00515 - Long	NA	Node-00515 - Long	1.000013	Node-00515 - Long	1.000013
Node-00515 - Lat	NA	Node-00515 - Lat	0.999968	Node-00515 - Lat	0.999968
Node-00516 - Long	NA	Node-00516 - Long	NA	Node-00516 - Long	NA
Node-00516 - Lat	NA	Node-00516 - Lat	NA	Node-00516 - Lat	NA
Node-00517 - Long	NA	Node-00517 - Long	NA	Node-00517 - Long	NA
Node-00517 - Lat	NA	Node-00517 - Lat	NA	Node-00517 - Lat	NA
Node-00518 - Long	NA	Node-00518 - Long	NA	Node-00518 - Long	NA
Node-00518 - Lat	NA	Node-00518 - Lat	NA	Node-00518 - Lat	NA
Node-00519 - Long	NA	Node-00519 - Long	NA	Node-00519 - Long	NA
Node-00519 - Lat	NA	Node-00519 - Lat	NA	Node-00519 - Lat	NA
Node-00520 - Long	NA	Node-00520 - Long	NA	Node-00520 - Long	NA
Node-00520 - Lat	NA	Node-00520 - Lat	NA	Node-00520 - Lat	NA
Node-00521 - Long	1.031737	Node-00521 - Long	NA	Node-00521 - Long	NA
Node-00521 - Lat	1.071623	Node-00521 - Lat	NA	Node-00521 - Lat	NA
Node-00522 - Long	1.031378	Node-00522 - Long	1.050244	Node-00522 - Long	1.050244
Node-00522 - Lat	1.048769	Node-00522 - Lat	1.105009	Node-00522 - Lat	1.105009
Node-00523 - Long	1.006895	Node-00523 - Long	1.050734	Node-00523 - Long	1.050734
Node-00523 - Lat	1.028933	Node-00523 - Lat	1.063566	Node-00523 - Lat	1.063566
Node-00524 - Long	1.002745	Node-00524 - Long	1.01031	Node-00524 - Long	1.01031

Node-00524 - Lat	1.010605	Node-00524 - Lat	1.02997	Node-00524 - Lat	1.02997
Node-00525 - Long	NA	Node-00525 - Long	1.004719	Node-00525 - Long	1.004719
Node-00525 - Lat	NA	Node-00525 - Lat	1.011031	Node-00525 - Lat	1.011031
Node-00526 - Long	1.000086	Node-00526 - Long	NA	Node-00526 - Long	NA
Node-00526 - Lat	1.000443	Node-00526 - Lat	NA	Node-00526 - Lat	NA
Node-00527 - Long	NA	Node-00527 - Long	1.000016	Node-00527 - Long	1.000016
Node-00527 - Lat	NA	Node-00527 - Lat	1.00001	Node-00527 - Lat	1.00001
Node-00528 - Long	NA	Node-00528 - Long	NA	Node-00528 - Long	NA
Node-00528 - Lat	NA	Node-00528 - Lat	NA	Node-00528 - Lat	NA
Node-00529 - Long	1.000201	Node-00529 - Long	NA	Node-00529 - Long	NA
Node-00529 - Lat	1.002518	Node-00529 - Lat	NA	Node-00529 - Lat	NA
Node-00530 - Long	NA	Node-00530 - Long	1.000099	Node-00530 - Long	1.000099
Node-00530 - Lat	NA	Node-00530 - Lat	1.001562	Node-00530 - Lat	1.001562
Node-00531 - Long	NA	Node-00531 - Long	NA	Node-00531 - Long	NA
Node-00531 - Lat	NA	Node-00531 - Lat	NA	Node-00531 - Lat	NA
Node-00532 - Long	1.380524	Node-00532 - Long	NA	Node-00532 - Long	NA
Node-00532 - Lat	3.683686	Node-00532 - Lat	NA	Node-00532 - Lat	NA
Node-00533 - Long	NA	Node-00533 - Long	1.181736	Node-00533 - Long	1.181736
Node-00533 - Lat	NA	Node-00533 - Lat	2.330039	Node-00533 - Lat	2.330039
Node-00534 - Long	1.061495	Node-00534 - Long	NA	Node-00534 - Long	NA
Node-00534 - Lat	1.000378	Node-00534 - Lat	NA	Node-00534 - Lat	NA
Node-00535 - Long	NA	Node-00535 - Long	1.037446	Node-00535 - Long	1.037446
Node-00535 - Lat	NA	Node-00535 - Lat	1.000086	Node-00535 - Lat	1.000086
Node-00536 - Long	NA	Node-00536 - Long	NA	Node-00536 - Long	NA
Node-00536 - Lat	NA	Node-00536 - Lat	NA	Node-00536 - Lat	NA
Node-00537 - Long	NA	Node-00537 - Long	NA	Node-00537 - Long	NA
Node-00537 - Lat	NA	Node-00537 - Lat	NA	Node-00537 - Lat	NA
Node-00538 - Long	1.06258	Node-00538 - Long	NA	Node-00538 - Long	NA
Node-00538 - Lat	4.507725	Node-00538 - Lat	NA	Node-00538 - Lat	NA
Node-00539 - Long	NA	Node-00539 - Long	1.099332	Node-00539 - Long	1.099332
Node-00539 - Lat	NA	Node-00539 - Lat	3.016703	Node-00539 - Lat	3.016703
Node-00540 - Long	NA	Node-00540 - Long	NA	Node-00540 - Long	NA
Node-00540 - Lat	NA	Node-00540 - Lat	NA	Node-00540 - Lat	NA
Node-00541 - Long	1.514995	Node-00541 - Long	NA	Node-00541 - Long	NA
Node-00541 - Lat	1.549794	Node-00541 - Lat	NA	Node-00541 - Lat	NA
Node-00542 - Long	1.434835	Node-00542 - Long	1.637951	Node-00542 - Long	1.637951
Node-00542 - Lat	1.417192	Node-00542 - Lat	1.77036	Node-00542 - Lat	1.77036
Node-00543 - Long	1.500337	Node-00543 - Long	1.623317	Node-00543 - Long	1.623317
Node-00543 - Lat	1.475582	Node-00543 - Lat	1.599752	Node-00543 - Lat	1.599752
Node-00544 - Long	1.006969	Node-00544 - Long	1.657059	Node-00544 - Long	1.657059
Node-00544 - Lat	1.007665	Node-00544 - Lat	1.585875	Node-00544 - Lat	1.585875
Node-00545 - Long	1.006953	Node-00545 - Long	1.005011	Node-00545 - Long	1.005011
Node-00545 - Lat	1.006217	Node-00545 - Lat	1.00628	Node-00545 - Lat	1.00628
Node-00546 - Long	1.00396	Node-00546 - Long	1.004638	Node-00546 - Long	1.004638
Node-00546 - Lat	1.00464	Node-00546 - Lat	1.005527	Node-00546 - Lat	1.005527
Node-00547 - Long	1.186624	Node-00547 - Long	1.00264	Node-00547 - Long	1.00264
Node-00547 - Lat	1.228395	Node-00547 - Lat	1.003656	Node-00547 - Lat	1.003656
Node-00548 - Long	1.158242	Node-00548 - Long	1.157313	Node-00548 - Long	1.157313

Node-00548 - Lat	1.22496	Node-00548 - Lat	1.170907	Node-00548 - Lat	1.170907
Node-00549 - Long	NA	Node-00549 - Long	1.16538	Node-00549 - Long	1.16538
Node-00549 - Lat	NA	Node-00549 - Lat	1.1647	Node-00549 - Lat	1.1647
Node-00550 - Long	NA	Node-00550 - Long	NA	Node-00550 - Long	NA
Node-00550 - Lat	NA	Node-00550 - Lat	NA	Node-00550 - Lat	NA
Node-00551 - Long	1.003022	Node-00551 - Long	NA	Node-00551 - Long	NA
Node-00551 - Lat	1.000249	Node-00551 - Lat	NA	Node-00551 - Lat	NA
Node-00552 - Long	1.005073	Node-00552 - Long	1.00597	Node-00552 - Long	1.00597
Node-00552 - Lat	1.000709	Node-00552 - Lat	1.000251	Node-00552 - Lat	1.000251
Node-00553 - Long	1.006143	Node-00553 - Long	1.011128	Node-00553 - Long	1.011128
Node-00553 - Lat	1.002228	Node-00553 - Lat	1.002112	Node-00553 - Lat	1.002112
Node-00554 - Long	1.008977	Node-00554 - Long	1.012159	Node-00554 - Long	1.012159
Node-00554 - Lat	1.005866	Node-00554 - Lat	1.004611	Node-00554 - Lat	1.004611
Node-00555 - Long	1.023033	Node-00555 - Long	1.015517	Node-00555 - Long	1.015517
Node-00555 - Lat	1.027734	Node-00555 - Lat	1.011275	Node-00555 - Lat	1.011275
Node-00556 - Long	4.033518	Node-00556 - Long	1.039422	Node-00556 - Long	1.039422
Node-00556 - Lat	3.862095	Node-00556 - Lat	1.044034	Node-00556 - Lat	1.044034
Node-00557 - Long	NA	Node-00557 - Long	18.87613	Node-00557 - Long	18.87613
Node-00557 - Lat	NA	Node-00557 - Lat	12.86279	Node-00557 - Lat	12.86279
Node-00558 - Long	4.158621	Node-00558 - Long	NA	Node-00558 - Long	NA
Node-00558 - Lat	4.158621	Node-00558 - Lat	NA	Node-00558 - Lat	NA
Node-00559 - Long	NA	Node-00559 - Long	Inf	Node-00559 - Long	Inf
Node-00559 - Lat	NA	Node-00559 - Lat	Inf	Node-00559 - Lat	Inf
Node-00560 - Long	1.005524	Node-00560 - Long	NA	Node-00560 - Long	NA
Node-00560 - Lat	1.003574	Node-00560 - Lat	NA	Node-00560 - Lat	NA
Node-00561 - Long	1.004208	Node-00561 - Long	1.011265	Node-00561 - Long	1.011265
Node-00561 - Lat	1.003249	Node-00561 - Lat	1.009014	Node-00561 - Lat	1.009014
Node-00562 - Long	1.003359	Node-00562 - Long	1.008826	Node-00562 - Long	1.008826
Node-00562 - Lat	1.00163	Node-00562 - Lat	1.005504	Node-00562 - Lat	1.005504
Node-00563 - Long	1.001525	Node-00563 - Long	1.007793	Node-00563 - Long	1.007793
Node-00563 - Lat	1.000225	Node-00563 - Lat	1.003787	Node-00563 - Lat	1.003787
Node-00564 - Long	1.001202	Node-00564 - Long	1.002796	Node-00564 - Long	1.002796
Node-00564 - Lat	1.000283	Node-00564 - Lat	1.000052	Node-00564 - Lat	1.000052
Node-00565 - Long	1.000867	Node-00565 - Long	1.002111	Node-00565 - Long	1.002111
Node-00565 - Lat	1.000214	Node-00565 - Lat	1.00006	Node-00565 - Lat	1.00006
Node-00566 - Long	NA	Node-00566 - Long	1.001936	Node-00566 - Long	1.001936
Node-00566 - Lat	NA	Node-00566 - Lat	1.000088	Node-00566 - Lat	1.000088
Node-00567 - Long	0.999968	Node-00567 - Long	NA	Node-00567 - Long	NA
Node-00567 - Lat	0.999992	Node-00567 - Lat	NA	Node-00567 - Lat	NA
Node-00568 - Long	NA	Node-00568 - Long	1.00001	Node-00568 - Long	1.00001
Node-00568 - Lat	NA	Node-00568 - Lat	0.999993	Node-00568 - Lat	0.999993
Node-00569 - Long	1.002406	Node-00569 - Long	NA	Node-00569 - Long	NA
Node-00569 - Lat	1.003675	Node-00569 - Lat	NA	Node-00569 - Lat	NA
Node-00570 - Long	NA	Node-00570 - Long	1.002125	Node-00570 - Long	1.002125
Node-00570 - Lat	NA	Node-00570 - Lat	1.003116	Node-00570 - Lat	1.003116
Node-00571 - Long	NA	Node-00571 - Long	NA	Node-00571 - Long	NA
Node-00571 - Lat	NA	Node-00571 - Lat	NA	Node-00571 - Lat	NA
Node-00572 - Long	0.999993	Node-00572 - Long	NA	Node-00572 - Long	NA

Node-00572 - Lat	0.999993	Node-00572 - Lat	NA	Node-00572 - Lat	NA
Node-00573 - Long	1.009987	Node-00573 - Long	1.00002	Node-00573 - Long	1.00002
Node-00573 - Lat	1.013823	Node-00573 - Lat	1.00002	Node-00573 - Lat	1.00002
Node-00574 - Long	NA	Node-00574 - Long	1.008294	Node-00574 - Long	1.008294
Node-00574 - Lat	NA	Node-00574 - Lat	1.011516	Node-00574 - Lat	1.011516
Node-00575 - Long	1.057188	Node-00575 - Long	NA	Node-00575 - Long	NA
Node-00575 - Lat	1.163017	Node-00575 - Lat	NA	Node-00575 - Lat	NA
Node-00576 - Long	NA	Node-00576 - Long	1.14721	Node-00576 - Long	1.14721
Node-00576 - Lat	NA	Node-00576 - Lat	1.252707	Node-00576 - Lat	1.252707
Node-00577 - Long	NA	Node-00577 - Long	NA	Node-00577 - Long	NA
Node-00577 - Lat	NA	Node-00577 - Lat	NA	Node-00577 - Lat	NA
Node-00578 - Long	1.000494	Node-00578 - Long	NA	Node-00578 - Long	NA
Node-00578 - Lat	1.000494	Node-00578 - Lat	NA	Node-00578 - Lat	NA
Node-00579 - Long	NA	Node-00579 - Long	1.000258	Node-00579 - Long	1.000258
Node-00579 - Lat	NA	Node-00579 - Lat	1.000258	Node-00579 - Lat	1.000258
Node-00580 - Long	1.032817	Node-00580 - Long	NA	Node-00580 - Long	NA
Node-00580 - Lat	1.089588	Node-00580 - Lat	NA	Node-00580 - Lat	NA
Node-00581 - Long	1.008466	Node-00581 - Long	1.058579	Node-00581 - Long	1.058579
Node-00581 - Lat	1.013353	Node-00581 - Lat	1.100025	Node-00581 - Lat	1.100025
Node-00582 - Long	NA	Node-00582 - Long	1.009838	Node-00582 - Long	1.009838
Node-00582 - Lat	NA	Node-00582 - Lat	1.021759	Node-00582 - Lat	1.021759
Node-00583 - Long	1.012528	Node-00583 - Long	NA	Node-00583 - Long	NA
Node-00583 - Lat	1.004554	Node-00583 - Lat	NA	Node-00583 - Lat	NA
Node-00584 - Long	1.015022	Node-00584 - Long	1.015645	Node-00584 - Long	1.015645
Node-00584 - Lat	1.003951	Node-00584 - Lat	1.005656	Node-00584 - Lat	1.005656
Node-00585 - Long	1.006498	Node-00585 - Long	1.019152	Node-00585 - Long	1.019152
Node-00585 - Lat	1.001413	Node-00585 - Lat	1.00462	Node-00585 - Lat	1.00462
Node-00586 - Long	1.00276	Node-00586 - Long	1.007017	Node-00586 - Long	1.007017
Node-00586 - Lat	1.00012	Node-00586 - Lat	1.002042	Node-00586 - Lat	1.002042
Node-00587 - Long	NA	Node-00587 - Long	1.002284	Node-00587 - Long	1.002284
Node-00587 - Lat	NA	Node-00587 - Lat	1.000078	Node-00587 - Lat	1.000078
Node-00588 - Long	NA	Node-00588 - Long	NA	Node-00588 - Long	NA
Node-00588 - Lat	NA	Node-00588 - Lat	NA	Node-00588 - Lat	NA
Node-00589 - Long	NA	Node-00589 - Long	NA	Node-00589 - Long	NA
Node-00589 - Lat	NA	Node-00589 - Lat	NA	Node-00589 - Lat	NA
Node-00590 - Long	1.006202	Node-00590 - Long	NA	Node-00590 - Long	NA
Node-00590 - Lat	1.005221	Node-00590 - Lat	NA	Node-00590 - Lat	NA
Node-00591 - Long	NA	Node-00591 - Long	1.013849	Node-00591 - Long	1.013849
Node-00591 - Lat	NA	Node-00591 - Lat	1.008024	Node-00591 - Lat	1.008024
Node-00592 - Long	1.000092	Node-00592 - Long	NA	Node-00592 - Long	NA
Node-00592 - Lat	1.000028	Node-00592 - Lat	NA	Node-00592 - Lat	NA
Node-00593 - Long	1.009676	Node-00593 - Long	1.000131	Node-00593 - Long	1.000131
Node-00593 - Lat	1.014797	Node-00593 - Lat	1.000012	Node-00593 - Lat	1.000012
Node-00594 - Long	1.002977	Node-00594 - Long	1.034216	Node-00594 - Long	1.034216
Node-00594 - Lat	1.000114	Node-00594 - Lat	1.046061	Node-00594 - Lat	1.046061
Node-00595 - Long	NA	Node-00595 - Long	1.116916	Node-00595 - Long	1.116916
Node-00595 - Lat	NA	Node-00595 - Lat	1.026859	Node-00595 - Lat	1.026859
Node-00596 - Long	1.000056	Node-00596 - Long	NA	Node-00596 - Long	NA

Node-00596 - Lat	1.000068	Node-00596 - Lat	NA	Node-00596 - Lat	NA
Node-00597 - Long	1.000047	Node-00597 - Long	1.00021	Node-00597 - Long	1.00021
Node-00597 - Lat	1.000113	Node-00597 - Lat	1.000119	Node-00597 - Lat	1.000119
Node-00598 - Long	1.000134	Node-00598 - Long	1.000098	Node-00598 - Long	1.000098
Node-00598 - Lat	1.000085	Node-00598 - Lat	1.00007	Node-00598 - Lat	1.00007
Node-00599 - Long	1.000082	Node-00599 - Long	1.000063	Node-00599 - Long	1.000063
Node-00599 - Lat	1.000046	Node-00599 - Lat	1.000028	Node-00599 - Lat	1.000028
Node-00600 - Long	NA	Node-00600 - Long	1.00013	Node-00600 - Long	1.00013
Node-00600 - Lat	NA	Node-00600 - Lat	1.000025	Node-00600 - Lat	1.000025
Node-00601 - Long	NA	Node-00601 - Long	NA	Node-00601 - Long	NA
Node-00601 - Lat	NA	Node-00601 - Lat	NA	Node-00601 - Lat	NA
Node-00602 - Long	NA	Node-00602 - Long	NA	Node-00602 - Long	NA
Node-00602 - Lat	NA	Node-00602 - Lat	NA	Node-00602 - Lat	NA
Node-00603 - Long	NA	Node-00603 - Long	NA	Node-00603 - Long	NA
Node-00603 - Lat	NA	Node-00603 - Lat	NA	Node-00603 - Lat	NA
Node-00604 - Long	NA	Node-00604 - Long	NA	Node-00604 - Long	NA
Node-00604 - Lat	NA	Node-00604 - Lat	NA	Node-00604 - Lat	NA
Node-00605 - Long	1.017909	Node-00605 - Long	NA	Node-00605 - Long	NA
Node-00605 - Lat	1.011521	Node-00605 - Lat	NA	Node-00605 - Lat	NA
Node-00606 - Long	1.051698	Node-00606 - Long	1.047555	Node-00606 - Long	1.047555
Node-00606 - Lat	1.105428	Node-00606 - Lat	1.043582	Node-00606 - Lat	1.043582
Node-00607 - Long	1.028605	Node-00607 - Long	1.060856	Node-00607 - Long	1.060856
Node-00607 - Lat	1.09467	Node-00607 - Lat	1.0357	Node-00607 - Lat	1.0357
Node-00608 - Long	NA	Node-00608 - Long	1.032943	Node-00608 - Long	1.032943
Node-00608 - Lat	NA	Node-00608 - Lat	1.033262	Node-00608 - Lat	1.033262
Node-00609 - Long	1.023623	Node-00609 - Long	NA	Node-00609 - Long	NA
Node-00609 - Lat	1.018592	Node-00609 - Lat	NA	Node-00609 - Lat	NA
Node-00610 - Long	NA	Node-00610 - Long	1.025614	Node-00610 - Long	1.025614
Node-00610 - Lat	NA	Node-00610 - Lat	1.014211	Node-00610 - Lat	1.014211
Node-00611 - Long	NA	Node-00611 - Long	NA	Node-00611 - Long	NA
Node-00611 - Lat	NA	Node-00611 - Lat	NA	Node-00611 - Lat	NA
Node-00612 - Long	NA	Node-00612 - Long	NA	Node-00612 - Long	NA
Node-00612 - Lat	NA	Node-00612 - Lat	NA	Node-00612 - Lat	NA
Node-00613 - Long	1.01039	Node-00613 - Long	NA	Node-00613 - Long	NA
Node-00613 - Lat	1.032065	Node-00613 - Lat	NA	Node-00613 - Lat	NA
Node-00614 - Long	NA	Node-00614 - Long	1.011883	Node-00614 - Long	1.011883
Node-00614 - Lat	NA	Node-00614 - Lat	1.010769	Node-00614 - Lat	1.010769
Node-00615 - Long	NA	Node-00615 - Long	NA	Node-00615 - Long	NA
Node-00615 - Lat	NA	Node-00615 - Lat	NA	Node-00615 - Lat	NA
Node-00616 - Long	1.036858	Node-00616 - Long	NA	Node-00616 - Long	NA
Node-00616 - Lat	1.008901	Node-00616 - Lat	NA	Node-00616 - Lat	NA
Node-00617 - Long	1.030211	Node-00617 - Long	1.024578	Node-00617 - Long	1.024578
Node-00617 - Lat	1.006824	Node-00617 - Lat	1.005994	Node-00617 - Lat	1.005994
Node-00618 - Long	1.020735	Node-00618 - Long	1.020872	Node-00618 - Long	1.020872
Node-00618 - Lat	1.005397	Node-00618 - Lat	1.004381	Node-00618 - Lat	1.004381
Node-00619 - Long	1.018926	Node-00619 - Long	1.014072	Node-00619 - Long	1.014072
Node-00619 - Lat	1.003491	Node-00619 - Lat	1.013344	Node-00619 - Lat	1.013344
Node-00620 - Long	NA	Node-00620 - Long	1.013074	Node-00620 - Long	1.013074

Node-00620 - Lat	NA	Node-00620 - Lat	1.005504	Node-00620 - Lat	1.005504
Node-00621 - Long	NA	Node-00621 - Long	NA	Node-00621 - Long	NA
Node-00621 - Lat	NA	Node-00621 - Lat	NA	Node-00621 - Lat	NA
Node-00622 - Long	1.000002	Node-00622 - Long	NA	Node-00622 - Long	NA
Node-00622 - Lat	0.999969	Node-00622 - Lat	NA	Node-00622 - Lat	NA
Node-00623 - Long	1.000021	Node-00623 - Long	1.000031	Node-00623 - Long	1.000031
Node-00623 - Lat	1.00002	Node-00623 - Lat	1.00007	Node-00623 - Lat	1.00007
Node-00624 - Long	1.024144	Node-00624 - Long	1.000035	Node-00624 - Long	1.000035
Node-00624 - Lat	1.022902	Node-00624 - Lat	0.99998	Node-00624 - Lat	0.99998
Node-00625 - Long	1.027588	Node-00625 - Long	1.108012	Node-00625 - Long	1.108012
Node-00625 - Lat	1.007018	Node-00625 - Lat	1.108587	Node-00625 - Lat	1.108587
Node-00626 - Long	NA	Node-00626 - Long	1.020824	Node-00626 - Long	1.020824
Node-00626 - Lat	NA	Node-00626 - Lat	1.005529	Node-00626 - Lat	1.005529
Node-00627 - Long	NA	Node-00627 - Long	NA	Node-00627 - Long	NA
Node-00627 - Lat	NA	Node-00627 - Lat	NA	Node-00627 - Lat	NA
Node-00628 - Long	NA	Node-00628 - Long	NA	Node-00628 - Long	NA
Node-00628 - Lat	NA	Node-00628 - Lat	NA	Node-00628 - Lat	NA
Node-00629 - Long	NA	Node-00629 - Long	NA	Node-00629 - Long	NA
Node-00629 - Lat	NA	Node-00629 - Lat	NA	Node-00629 - Lat	NA
Node-00630 - Long	1.00883	Node-00630 - Long	NA	Node-00630 - Long	NA
Node-00630 - Lat	1.040807	Node-00630 - Lat	NA	Node-00630 - Lat	NA
Node-00631 - Long	NA	Node-00631 - Long	1.00969	Node-00631 - Long	1.00969
Node-00631 - Lat	NA	Node-00631 - Lat	1.016543	Node-00631 - Lat	1.016543
Node-00632 - Long	NA	Node-00632 - Long	NA	Node-00632 - Long	NA
Node-00632 - Lat	NA	Node-00632 - Lat	NA	Node-00632 - Lat	NA
Node-00633 - Long	NA	Node-00633 - Long	NA	Node-00633 - Long	NA
Node-00633 - Lat	NA	Node-00633 - Lat	NA	Node-00633 - Lat	NA
Node-00634 - Long	NA	Node-00634 - Long	NA	Node-00634 - Long	NA
Node-00634 - Lat	NA	Node-00634 - Lat	NA	Node-00634 - Lat	NA
Node-00635 - Long	1.003347	Node-00635 - Long	NA	Node-00635 - Long	NA
Node-00635 - Lat	1.004094	Node-00635 - Lat	NA	Node-00635 - Lat	NA
Node-00636 - Long	0.999998	Node-00636 - Long	1.002325	Node-00636 - Long	1.002325
Node-00636 - Lat	0.999998	Node-00636 - Lat	1.002873	Node-00636 - Lat	1.002873
Node-00637 - Long	1.000813	Node-00637 - Long	1.000065	Node-00637 - Long	1.000065
Node-00637 - Lat	1.003	Node-00637 - Lat	1.000065	Node-00637 - Lat	1.000065
Node-00638 - Long	NA	Node-00638 - Long	1.0006	Node-00638 - Long	1.0006
Node-00638 - Lat	NA	Node-00638 - Lat	1.0018	Node-00638 - Lat	1.0018
Node-00639 - Long	1.000178	Node-00639 - Long	NA	Node-00639 - Long	NA
Node-00639 - Lat	1.00019	Node-00639 - Lat	NA	Node-00639 - Lat	NA
Node-00640 - Long	1.0023	Node-00640 - Long	1.000094	Node-00640 - Long	1.000094
Node-00640 - Lat	1.007069	Node-00640 - Lat	1.000105	Node-00640 - Lat	1.000105
Node-00641 - Long	1.000057	Node-00641 - Long	1.001713	Node-00641 - Long	1.001713
Node-00641 - Lat	1.000045	Node-00641 - Lat	1.005244	Node-00641 - Lat	1.005244
Node-00642 - Long	1.000586	Node-00642 - Long	1.000047	Node-00642 - Long	1.000047
Node-00642 - Lat	1.003099	Node-00642 - Lat	1.000046	Node-00642 - Lat	1.000046
Node-00643 - Long	NA	Node-00643 - Long	1.000124	Node-00643 - Long	1.000124
Node-00643 - Lat	NA	Node-00643 - Lat	1.001147	Node-00643 - Lat	1.001147
Node-00644 - Long	NA	Node-00644 - Long	NA	Node-00644 - Long	NA

Node-00644 - Lat	NA	Node-00644 - Lat	NA	Node-00644 - Lat	NA
Node-00645 - Long	NA	Node-00645 - Long	NA	Node-00645 - Long	NA
Node-00645 - Lat	NA	Node-00645 - Lat	NA	Node-00645 - Lat	NA
Node-00646 - Long	1.000082	Node-00646 - Long	NA	Node-00646 - Long	NA
Node-00646 - Lat	1.000082	Node-00646 - Lat	NA	Node-00646 - Lat	NA
Node-00647 - Long	NA	Node-00647 - Long	1.000057	Node-00647 - Long	1.000057
Node-00647 - Lat	NA	Node-00647 - Lat	1.000057	Node-00647 - Lat	1.000057
Node-00648 - Long	1.000746	Node-00648 - Long	NA	Node-00648 - Long	NA
Node-00648 - Lat	1.005611	Node-00648 - Lat	NA	Node-00648 - Lat	NA
Node-00649 - Long	NA	Node-00649 - Long	1.000753	Node-00649 - Long	1.000753
Node-00649 - Lat	NA	Node-00649 - Lat	1.001846	Node-00649 - Lat	1.001846
Node-00650 - Long	1.000386	Node-00650 - Long	NA	Node-00650 - Long	NA
Node-00650 - Lat	1.000314	Node-00650 - Lat	NA	Node-00650 - Lat	NA
Node-00651 - Long	NA	Node-00651 - Long	1.00044	Node-00651 - Long	1.00044
Node-00651 - Lat	NA	Node-00651 - Lat	1.000498	Node-00651 - Lat	1.000498
Node-00652 - Long	1.014448	Node-00652 - Long	NA	Node-00652 - Long	NA
Node-00652 - Lat	1.093158	Node-00652 - Lat	NA	Node-00652 - Lat	NA
Node-00653 - Long	1.001067	Node-00653 - Long	1.041077	Node-00653 - Long	1.041077
Node-00653 - Lat	1.00266	Node-00653 - Lat	1.204129	Node-00653 - Lat	1.204129
Node-00654 - Long	1.000899	Node-00654 - Long	1.003425	Node-00654 - Long	1.003425
Node-00654 - Lat	1.004101	Node-00654 - Lat	1.008382	Node-00654 - Lat	1.008382
Node-00655 - Long	1.00122	Node-00655 - Long	1.002703	Node-00655 - Long	1.002703
Node-00655 - Lat	1.006681	Node-00655 - Lat	1.01432	Node-00655 - Lat	1.01432
Node-00656 - Long	1.009764	Node-00656 - Long	1.003853	Node-00656 - Long	1.003853
Node-00656 - Lat	1.099459	Node-00656 - Lat	1.022747	Node-00656 - Lat	1.022747
Node-00657 - Long	1.008574	Node-00657 - Long	1.021933	Node-00657 - Long	1.021933
Node-00657 - Lat	1.099344	Node-00657 - Lat	1.315574	Node-00657 - Lat	1.315574
Node-00658 - Long	1.007731	Node-00658 - Long	1.018613	Node-00658 - Long	1.018613
Node-00658 - Lat	1.053549	Node-00658 - Lat	1.316181	Node-00658 - Lat	1.316181
Node-00659 - Long	NA	Node-00659 - Long	1.024107	Node-00659 - Long	1.024107
Node-00659 - Lat	NA	Node-00659 - Lat	1.145283	Node-00659 - Lat	1.145283
Node-00660 - Long	1.023396	Node-00660 - Long	NA	Node-00660 - Long	NA
Node-00660 - Lat	1.023846	Node-00660 - Lat	NA	Node-00660 - Lat	NA
Node-00661 - Long	1.011611	Node-00661 - Long	1.056702	Node-00661 - Long	1.056702
Node-00661 - Lat	1.006433	Node-00661 - Lat	1.063423	Node-00661 - Lat	1.063423
Node-00662 - Long	NA	Node-00662 - Long	1.027225	Node-00662 - Long	1.027225
Node-00662 - Lat	NA	Node-00662 - Lat	1.019377	Node-00662 - Lat	1.019377
Node-00663 - Long	NA	Node-00663 - Long	NA	Node-00663 - Long	NA
Node-00663 - Lat	NA	Node-00663 - Lat	NA	Node-00663 - Lat	NA
Node-00664 - Long	NA	Node-00664 - Long	NA	Node-00664 - Long	NA
Node-00664 - Lat	NA	Node-00664 - Lat	NA	Node-00664 - Lat	NA
Node-00665 - Long	NA	Node-00665 - Long	NA	Node-00665 - Long	NA
Node-00665 - Lat	NA	Node-00665 - Lat	NA	Node-00665 - Lat	NA
Node-00666 - Long	1.289643	Node-00666 - Long	NA	Node-00666 - Long	NA
Node-00666 - Lat	1.289658	Node-00666 - Lat	NA	Node-00666 - Lat	NA
Node-00667 - Long	1.288483	Node-00667 - Long	1.131578	Node-00667 - Long	1.131578
Node-00667 - Lat	1.28909	Node-00667 - Lat	1.197285	Node-00667 - Lat	1.197285
Node-00668 - Long	NA	Node-00668 - Long	1.15719	Node-00668 - Long	1.15719

Node-00668 - Lat	NA	Node-00668 - Lat	1.17194	Node-00668 - Lat	1.17194
Node-00669 - Long	NA	Node-00669 - Long	NA	Node-00669 - Long	NA
Node-00669 - Lat	NA	Node-00669 - Lat	NA	Node-00669 - Lat	NA
Node-00670 - Long	1.000098	Node-00670 - Long	NA	Node-00670 - Long	NA
Node-00670 - Lat	1.000133	Node-00670 - Lat	NA	Node-00670 - Lat	NA
Node-00671 - Long	NA	Node-00671 - Long	1.000099	Node-00671 - Long	1.000099
Node-00671 - Lat	NA	Node-00671 - Lat	1.000128	Node-00671 - Lat	1.000128
Node-00672 - Long	1.001269	Node-00672 - Long	NA	Node-00672 - Long	NA
Node-00672 - Lat	1.005633	Node-00672 - Lat	NA	Node-00672 - Lat	NA
Node-00673 - Long	1.002012	Node-00673 - Long	1.00073	Node-00673 - Long	1.00073
Node-00673 - Lat	1.006504	Node-00673 - Lat	1.005124	Node-00673 - Lat	1.005124
Node-00674 - Long	1.000089	Node-00674 - Long	1.001305	Node-00674 - Long	1.001305
Node-00674 - Lat	1.00028	Node-00674 - Lat	1.006166	Node-00674 - Lat	1.006166
Node-00675 - Long	1.000282	Node-00675 - Long	1.000153	Node-00675 - Long	1.000153
Node-00675 - Lat	1.000482	Node-00675 - Lat	1.000761	Node-00675 - Lat	1.000761
Node-00676 - Long	1.000335	Node-00676 - Long	1.000203	Node-00676 - Long	1.000203
Node-00676 - Lat	1.000592	Node-00676 - Lat	1.0007	Node-00676 - Lat	1.0007
Node-00677 - Long	0.99996	Node-00677 - Long	1.000233	Node-00677 - Long	1.000233
Node-00677 - Lat	0.99996	Node-00677 - Lat	1.004215	Node-00677 - Lat	1.004215
Node-00678 - Long	NA	Node-00678 - Long	0.999996	Node-00678 - Long	0.999996
Node-00678 - Lat	NA	Node-00678 - Lat	0.999996	Node-00678 - Lat	0.999996
Node-00679 - Long	1.000178	Node-00679 - Long	NA	Node-00679 - Long	NA
Node-00679 - Lat	1.000376	Node-00679 - Lat	NA	Node-00679 - Lat	NA
Node-00680 - Long	NA	Node-00680 - Long	1.000496	Node-00680 - Long	1.000496
Node-00680 - Lat	NA	Node-00680 - Lat	1.000134	Node-00680 - Lat	1.000134
Node-00681 - Long	NA	Node-00681 - Long	NA	Node-00681 - Long	NA
Node-00681 - Lat	NA	Node-00681 - Lat	NA	Node-00681 - Lat	NA
Node-00682 - Long	NA	Node-00682 - Long	NA	Node-00682 - Long	NA
Node-00682 - Lat	NA	Node-00682 - Lat	NA	Node-00682 - Lat	NA
Node-00683 - Long	NA	Node-00683 - Long	NA	Node-00683 - Long	NA
Node-00683 - Lat	NA	Node-00683 - Lat	NA	Node-00683 - Lat	NA
Node-00684 - Long	NA	Node-00684 - Long	NA	Node-00684 - Long	NA
Node-00684 - Lat	NA	Node-00684 - Lat	NA	Node-00684 - Lat	NA
Node-00685 - Long	NA	Node-00685 - Long	NA	Node-00685 - Long	NA
Node-00685 - Lat	NA	Node-00685 - Lat	NA	Node-00685 - Lat	NA
Node-00686 - Long	NA	Node-00686 - Long	NA	Node-00686 - Long	NA
Node-00686 - Lat	NA	Node-00686 - Lat	NA	Node-00686 - Lat	NA
Node-00687 - Long	1.030355	Node-00687 - Long	NA	Node-00687 - Long	NA
Node-00687 - Lat	1.09535	Node-00687 - Lat	NA	Node-00687 - Lat	NA
Node-00688 - Long	NA	Node-00688 - Long	1.075306	Node-00688 - Long	1.075306
Node-00688 - Lat	NA	Node-00688 - Lat	1.204411	Node-00688 - Lat	1.204411
Node-00689 - Long	NA	Node-00689 - Long	NA	Node-00689 - Long	NA
Node-00689 - Lat	NA	Node-00689 - Lat	NA	Node-00689 - Lat	NA
Node-00690 - Long	1.001874	Node-00690 - Long	NA	Node-00690 - Long	NA
Node-00690 - Lat	1.007191	Node-00690 - Lat	NA	Node-00690 - Lat	NA
Node-00691 - Long	1.000343	Node-00691 - Long	1.000708	Node-00691 - Long	1.000708
Node-00691 - Lat	1.000427	Node-00691 - Lat	1.001631	Node-00691 - Lat	1.001631
Node-00692 - Long	NA	Node-00692 - Long	1.000578	Node-00692 - Long	1.000578

Node-00692 - Lat	NA	Node-00692 - Lat	1.001974	Node-00692 - Lat	1.001974
Node-00693 - Long	1.001792	Node-00693 - Long	NA	Node-00693 - Long	NA
Node-00693 - Lat	1.002796	Node-00693 - Lat	NA	Node-00693 - Lat	NA
Node-00694 - Long	297902.5	Node-00694 - Long	1.002973	Node-00694 - Long	1.002973
Node-00694 - Lat	9595.523	Node-00694 - Lat	1.005083	Node-00694 - Lat	1.005083
Node-00695 - Long	502371.9	Node-00695 - Long	298487.3	Node-00695 - Long	298487.3
Node-00695 - Lat	12764.31	Node-00695 - Lat	7942.373	Node-00695 - Lat	7942.373
Node-00696 - Long	Inf	Node-00696 - Long	491513.3	Node-00696 - Long	491513.3
Node-00696 - Lat	Inf	Node-00696 - Lat	15148.71	Node-00696 - Lat	15148.71
Node-00697 - Long	Inf	Node-00697 - Long	Inf	Node-00697 - Long	Inf
Node-00697 - Lat	Inf	Node-00697 - Lat	Inf	Node-00697 - Lat	Inf
Node-00698 - Long	NA	Node-00698 - Long	Inf	Node-00698 - Long	Inf
Node-00698 - Lat	NA	Node-00698 - Lat	Inf	Node-00698 - Lat	Inf
Node-00699 - Long	NA	Node-00699 - Long	NA	Node-00699 - Long	NA
Node-00699 - Lat	NA	Node-00699 - Lat	NA	Node-00699 - Lat	NA
Node-00700 - Long	1.00009	Node-00700 - Long	NA	Node-00700 - Long	NA
Node-00700 - Lat	1.000113	Node-00700 - Lat	NA	Node-00700 - Lat	NA
Node-00701 - Long	1.000038	Node-00701 - Long	1.000065	Node-00701 - Long	1.000065
Node-00701 - Lat	1.000091	Node-00701 - Lat	1.000264	Node-00701 - Lat	1.000264
Node-00702 - Long	1.03172	Node-00702 - Long	1.000072	Node-00702 - Long	1.000072
Node-00702 - Lat	1.03172	Node-00702 - Lat	1.00014	Node-00702 - Lat	1.00014
Node-00703 - Long	NA	Node-00703 - Long	1.04476	Node-00703 - Long	1.04476
Node-00703 - Lat	NA	Node-00703 - Lat	1.04476	Node-00703 - Lat	1.04476
Node-00704 - Long	NA	Node-00704 - Long	NA	Node-00704 - Long	NA
Node-00704 - Lat	NA	Node-00704 - Lat	NA	Node-00704 - Lat	NA
Node-00705 - Long	1.000581	Node-00705 - Long	NA	Node-00705 - Long	NA
Node-00705 - Lat	1.001197	Node-00705 - Lat	NA	Node-00705 - Lat	NA
Node-00706 - Long	1.000181	Node-00706 - Long	1.000248	Node-00706 - Long	1.000248
Node-00706 - Lat	1.000566	Node-00706 - Lat	1.000629	Node-00706 - Lat	1.000629
Node-00707 - Long	1.000246	Node-00707 - Long	1.000164	Node-00707 - Long	1.000164
Node-00707 - Lat	1.000356	Node-00707 - Lat	1.000579	Node-00707 - Lat	1.000579
Node-00708 - Long	1.000132	Node-00708 - Long	1.000095	Node-00708 - Long	1.000095
Node-00708 - Lat	1.000438	Node-00708 - Lat	1.000319	Node-00708 - Lat	1.000319
Node-00709 - Long	1.000464	Node-00709 - Long	1.000108	Node-00709 - Long	1.000108
Node-00709 - Lat	1.000747	Node-00709 - Lat	1.000294	Node-00709 - Lat	1.000294
Node-00710 - Long	1.000262	Node-00710 - Long	1.00008	Node-00710 - Long	1.00008
Node-00710 - Lat	1.001703	Node-00710 - Lat	1.000575	Node-00710 - Lat	1.000575
Node-00711 - Long	1.000172	Node-00711 - Long	1.000421	Node-00711 - Long	1.000421
Node-00711 - Lat	1.000177	Node-00711 - Lat	1.001541	Node-00711 - Lat	1.001541
Node-00712 - Long	1.000303	Node-00712 - Long	1.000055	Node-00712 - Long	1.000055
Node-00712 - Lat	1.001771	Node-00712 - Lat	1.000051	Node-00712 - Lat	1.000051
Node-00713 - Long	1.000231	Node-00713 - Long	1.000337	Node-00713 - Long	1.000337
Node-00713 - Lat	1.000576	Node-00713 - Lat	1.001948	Node-00713 - Lat	1.001948
Node-00714 - Long	1.003401	Node-00714 - Long	1.000265	Node-00714 - Long	1.000265
Node-00714 - Lat	1.002316	Node-00714 - Lat	1.000572	Node-00714 - Lat	1.000572
Node-00715 - Long	1.003682	Node-00715 - Long	1.001049	Node-00715 - Long	1.001049
Node-00715 - Lat	1.002248	Node-00715 - Lat	1.000749	Node-00715 - Lat	1.000749
Node-00716 - Long	1.000213	Node-00716 - Long	1.001257	Node-00716 - Long	1.001257

Node-00716 - Lat	1.000136	Node-00716 - Lat	1.000782	Node-00716 - Lat	1.000782
Node-00717 - Long	NA	Node-00717 - Long	1.000274	Node-00717 - Long	1.000274
Node-00717 - Lat	NA	Node-00717 - Lat	1.000097	Node-00717 - Lat	1.000097
Node-00718 - Long	1.000004	Node-00718 - Long	NA	Node-00718 - Long	NA
Node-00718 - Lat	1.000002	Node-00718 - Lat	NA	Node-00718 - Lat	NA
Node-00719 - Long	1.000048	Node-00719 - Long	0.999979	Node-00719 - Long	0.999979
Node-00719 - Lat	1.000048	Node-00719 - Lat	0.999972	Node-00719 - Lat	0.999972
Node-00720 - Long	NA	Node-00720 - Long	0.999984	Node-00720 - Long	0.999984
Node-00720 - Lat	NA	Node-00720 - Lat	0.999984	Node-00720 - Lat	0.999984
Node-00721 - Long	1.001952	Node-00721 - Long	NA	Node-00721 - Long	NA
Node-00721 - Lat	1.000944	Node-00721 - Lat	NA	Node-00721 - Lat	NA
Node-00722 - Long	1.0002	Node-00722 - Long	1.000127	Node-00722 - Long	1.000127
Node-00722 - Lat	1.0002	Node-00722 - Lat	1.000241	Node-00722 - Lat	1.000241
Node-00723 - Long	NA	Node-00723 - Long	0.999989	Node-00723 - Long	0.999989
Node-00723 - Lat	NA	Node-00723 - Lat	0.999989	Node-00723 - Lat	0.999989
Node-00724 - Long	1.0004	Node-00724 - Long	NA	Node-00724 - Long	NA
Node-00724 - Lat	1.002034	Node-00724 - Lat	NA	Node-00724 - Lat	NA
Node-00725 - Long	1.000118	Node-00725 - Long	1.000614	Node-00725 - Long	1.000614
Node-00725 - Lat	1.000601	Node-00725 - Lat	1.002248	Node-00725 - Lat	1.002248
Node-00726 - Long	NA	Node-00726 - Long	1.000124	Node-00726 - Long	1.000124
Node-00726 - Lat	NA	Node-00726 - Lat	1.000804	Node-00726 - Lat	1.000804
Node-00727 - Long	NA	Node-00727 - Long	NA	Node-00727 - Long	NA
Node-00727 - Lat	NA	Node-00727 - Lat	NA	Node-00727 - Lat	NA
Node-00728 - Long	1.000789	Node-00728 - Long	NA	Node-00728 - Long	NA
Node-00728 - Lat	1.002334	Node-00728 - Lat	NA	Node-00728 - Lat	NA
Node-00729 - Long	NA	Node-00729 - Long	1.000837	Node-00729 - Long	1.000837
Node-00729 - Lat	NA	Node-00729 - Lat	1.002314	Node-00729 - Lat	1.002314
Node-00730 - Long	NA	Node-00730 - Long	NA	Node-00730 - Long	NA
Node-00730 - Lat	NA	Node-00730 - Lat	NA	Node-00730 - Lat	NA
Node-00731 - Long	1.002039	Node-00731 - Long	NA	Node-00731 - Long	NA
Node-00731 - Lat	1.00001	Node-00731 - Lat	NA	Node-00731 - Lat	NA
Node-00732 - Long	NA	Node-00732 - Long	1.00201	Node-00732 - Long	1.00201
Node-00732 - Lat	NA	Node-00732 - Lat	0.999985	Node-00732 - Lat	0.999985
Node-00733 - Long	0.999995	Node-00733 - Long	NA	Node-00733 - Long	NA
Node-00733 - Lat	1.000007	Node-00733 - Lat	NA	Node-00733 - Lat	NA
Node-00734 - Long	NA	Node-00734 - Long	0.999991	Node-00734 - Long	0.999991
Node-00734 - Lat	NA	Node-00734 - Lat	1.000022	Node-00734 - Lat	1.000022
Node-00735 - Long	1.000186	Node-00735 - Long	NA	Node-00735 - Long	NA
Node-00735 - Lat	1.000151	Node-00735 - Lat	NA	Node-00735 - Lat	NA
Node-00736 - Long	NA	Node-00736 - Long	1.000131	Node-00736 - Long	1.000131
Node-00736 - Lat	NA	Node-00736 - Lat	1.000241	Node-00736 - Lat	1.000241
Node-00737 - Long	NA	Node-00737 - Long	NA	Node-00737 - Long	NA
Node-00737 - Lat	NA	Node-00737 - Lat	NA	Node-00737 - Lat	NA
Node-00738 - Long	NA	Node-00738 - Long	NA	Node-00738 - Long	NA
Node-00738 - Lat	NA	Node-00738 - Lat	NA	Node-00738 - Lat	NA
Node-00739 - Long	1.000223	Node-00739 - Long	NA	Node-00739 - Long	NA
Node-00739 - Lat	1.000467	Node-00739 - Lat	NA	Node-00739 - Lat	NA
Node-00740 - Long	1.000167	Node-00740 - Long	1.000545	Node-00740 - Long	1.000545

Node-00740 - Lat	1.000084	Node-00740 - Lat	1.000337	Node-00740 - Lat	1.000337
Node-00741 - Long	NA	Node-00741 - Long	1.000204	Node-00741 - Long	1.000204
Node-00741 - Lat	NA	Node-00741 - Lat	1.000295	Node-00741 - Lat	1.000295
Node-00742 - Long	0.999988	Node-00742 - Long	NA	Node-00742 - Long	NA
Node-00742 - Lat	0.999996	Node-00742 - Lat	NA	Node-00742 - Lat	NA
Node-00743 - Long	NA	Node-00743 - Long	0.999968	Node-00743 - Long	0.999968
Node-00743 - Lat	NA	Node-00743 - Lat	0.999972	Node-00743 - Lat	0.999972
Node-00744 - Long	1.235212	Node-00744 - Long	NA	Node-00744 - Long	NA
Node-00744 - Lat	1.233843	Node-00744 - Lat	NA	Node-00744 - Lat	NA
Node-00745 - Long	1.207065	Node-00745 - Long	1.275386	Node-00745 - Long	1.275386
Node-00745 - Lat	1.230091	Node-00745 - Lat	1.260213	Node-00745 - Lat	1.260213
Node-00746 - Long	NA	Node-00746 - Long	1.250625	Node-00746 - Long	1.250625
Node-00746 - Lat	NA	Node-00746 - Lat	1.248919	Node-00746 - Lat	1.248919
Node-00747 - Long	NA	Node-00747 - Long	NA	Node-00747 - Long	NA
Node-00747 - Lat	NA	Node-00747 - Lat	NA	Node-00747 - Lat	NA
Node-00748 - Long	NA	Node-00748 - Long	NA	Node-00748 - Long	NA
Node-00748 - Lat	NA	Node-00748 - Lat	NA	Node-00748 - Lat	NA
Node-00749 - Long	1.000106	Node-00749 - Long	NA	Node-00749 - Long	NA
Node-00749 - Lat	1.000523	Node-00749 - Lat	NA	Node-00749 - Lat	NA
Node-00750 - Long	NA	Node-00750 - Long	1.000229	Node-00750 - Long	1.000229
Node-00750 - Lat	NA	Node-00750 - Lat	1.000446	Node-00750 - Lat	1.000446
Node-00751 - Long	1.00035	Node-00751 - Long	NA	Node-00751 - Long	NA
Node-00751 - Lat	1.000435	Node-00751 - Lat	NA	Node-00751 - Lat	NA
Node-00752 - Long	1.000325	Node-00752 - Long	1.000086	Node-00752 - Long	1.000086
Node-00752 - Lat	1.000325	Node-00752 - Lat	1.000089	Node-00752 - Lat	1.000089
Node-00753 - Long	1.000254	Node-00753 - Long	1.000077	Node-00753 - Long	1.000077
Node-00753 - Lat	1.00055	Node-00753 - Lat	1.000077	Node-00753 - Lat	1.000077
		Node-00754 - Long	1.000145	Node-00754 - Long	1.000145
		Node-00754 - Lat	1.000158	Node-00754 - Lat	1.000158

both_bm	both_bm
param	psrf
Lh	2.674024
Scale	1.145709
Node-00000 - Long	1.00221
Node-00000 - Lat	1.004242
Node-00001 - Long	1.002944
Node-00001 - Lat	1.004982
Node-00002 - Long	1.004158
Node-00002 - Lat	1.006153
Node-00003 - Long	1.00555
Node-00003 - Lat	1.013305
Node-00004 - Long	1.007451
Node-00004 - Lat	1.01549
Node-00005 - Long	1.010829
Node-00005 - Lat	1.020564
Node-00006 - Long	1.008719
Node-00006 - Lat	1.022085
Node-00007 - Long	1.008431
Node-00007 - Lat	1.024498
Node-00008 - Long	1.006886
Node-00008 - Lat	1.026313
Node-00009 - Long	1.008479
Node-00009 - Lat	1.018809
Node-00010 - Long	1.006877
Node-00010 - Lat	1.016797
Node-00011 - Long	1.007921
Node-00011 - Lat	1.019242
Node-00012 - Long	1.007042
Node-00012 - Lat	1.016973
Node-00013 - Long	1.008933
Node-00013 - Lat	1.017598
Node-00014 - Long	1.00469
Node-00014 - Lat	1.010236
Node-00015 - Long	1.002564
Node-00015 - Lat	1.00655
Node-00016 - Long	1.0023
Node-00016 - Lat	1.004583
Node-00017 - Long	1.001551
Node-00017 - Lat	1.002425
Node-00018 - Long	1.003848
Node-00018 - Lat	1.000912
Node-00019 - Long	1.003148
Node-00019 - Lat	1.000504
Node-00020 - Long	1.004032

Node-00020 - Lat	1.000436
Node-00021 - Long	1.00566
Node-00021 - Lat	1.000277
Node-00022 - Long	1.004912
Node-00022 - Lat	1.00032
Node-00023 - Long	1.003003
Node-00023 - Lat	1.00039
Node-00024 - Long	1.00481
Node-00024 - Lat	1.001151
Node-00025 - Long	1.007728
Node-00025 - Lat	1.002601
Node-00026 - Long	1.012429
Node-00026 - Lat	1.005067
Node-00027 - Long	1.015615
Node-00027 - Lat	1.006676
Node-00028 - Long	1.034544
Node-00028 - Lat	1.05423
Node-00029 - Long	2.092361
Node-00029 - Lat	12.40743
Node-00030 - Long	4.898595
Node-00030 - Lat	34.96695
Node-00031 - Long	1.208257
Node-00031 - Lat	13.81643
Node-00032 - Long	1.014985
Node-00032 - Lat	14.18726
Node-00033 - Long	2.63695
Node-00033 - Lat	43.7785
Node-00034 - Long	1.011479
Node-00034 - Lat	1.020006
Node-00035 - Long	1.0105
Node-00035 - Lat	1.014102
Node-00036 - Long	1.010004
Node-00036 - Lat	1.011676
Node-00037 - Long	1.010216
Node-00037 - Lat	1.010195
Node-00038 - Long	1.006517
Node-00038 - Lat	1.009749
Node-00039 - Long	1.004621
Node-00039 - Lat	1.006526
Node-00040 - Long	1.003588
Node-00040 - Lat	1.003964
Node-00041 - Long	1.002406
Node-00041 - Lat	1.002834
Node-00042 - Long	1.001574
Node-00042 - Lat	1.002492
Node-00043 - Long	1.001208
Node-00043 - Lat	1.00164
Node-00044 - Long	1.000655

Node-00044 - Lat	1.001126
Node-00045 - Long	1.000381
Node-00045 - Lat	1.000258
Node-00046 - Long	1.000194
Node-00046 - Lat	1.000238
Node-00047 - Long	NA
Node-00047 - Lat	NA
Node-00048 - Long	NA
Node-00048 - Lat	NA
Node-00049 - Long	NA
Node-00049 - Lat	NA
Node-00050 - Long	1.000255
Node-00050 - Lat	1.000566
Node-00051 - Long	1.000157
Node-00051 - Lat	1.000343
Node-00052 - Long	1.000042
Node-00052 - Lat	1.000458
Node-00053 - Long	1.000103
Node-00053 - Lat	1.000283
Node-00054 - Long	1.000736
Node-00054 - Lat	1.000667
Node-00055 - Long	1.000396
Node-00055 - Lat	1.000525
Node-00056 - Long	1.000012
Node-00056 - Lat	1.000095
Node-00057 - Long	NA
Node-00057 - Lat	NA
Node-00058 - Long	NA
Node-00058 - Lat	NA
Node-00059 - Long	NA
Node-00059 - Lat	NA
Node-00060 - Long	1.000235
Node-00060 - Lat	1.00139
Node-00061 - Long	1.00021
Node-00061 - Lat	1.001174
Node-00062 - Long	1.000406
Node-00062 - Lat	1.001143
Node-00063 - Long	1.000076
Node-00063 - Lat	1.000617
Node-00064 - Long	1.000119
Node-00064 - Lat	1.000361
Node-00065 - Long	1.000197
Node-00065 - Lat	1.000504
Node-00066 - Long	NA
Node-00066 - Lat	NA
Node-00067 - Long	NA
Node-00067 - Lat	NA
Node-00068 - Long	NA

Node-00068 - Lat	NA
Node-00069 - Long	NA
Node-00069 - Lat	NA
Node-00070 - Long	NA
Node-00070 - Lat	NA
Node-00071 - Long	NA
Node-00071 - Lat	NA
Node-00072 - Long	1.00028
Node-00072 - Lat	1.000811
Node-00073 - Long	1.000183
Node-00073 - Lat	1.000474
Node-00074 - Long	1.000066
Node-00074 - Lat	1.000121
Node-00075 - Long	NA
Node-00075 - Lat	NA
Node-00076 - Long	NA
Node-00076 - Lat	NA
Node-00077 - Long	NA
Node-00077 - Lat	NA
Node-00078 - Long	NA
Node-00078 - Lat	NA
Node-00079 - Long	NA
Node-00079 - Lat	NA
Node-00080 - Long	NA
Node-00080 - Lat	NA
Node-00081 - Long	1.271304
Node-00081 - Lat	1.260768
Node-00082 - Long	1.274693
Node-00082 - Lat	1.276172
Node-00083 - Long	NA
Node-00083 - Lat	NA
Node-00084 - Long	NA
Node-00084 - Lat	NA
Node-00085 - Long	1.000105
Node-00085 - Lat	1.00004
Node-00086 - Long	NA
Node-00086 - Lat	NA
Node-00087 - Long	NA
Node-00087 - Lat	NA
Node-00088 - Long	NA
Node-00088 - Lat	NA
Node-00089 - Long	1.000218
Node-00089 - Lat	1.000257
Node-00090 - Long	NA
Node-00090 - Lat	NA
Node-00091 - Long	1.000017
Node-00091 - Lat	1.00002
Node-00092 - Long	NA

Node-00092 - Lat	NA
Node-00093 - Long	NA
Node-00093 - Lat	NA
Node-00094 - Long	1.001033
Node-00094 - Lat	1.000865
Node-00095 - Long	NA
Node-00095 - Lat	NA
Node-00096 - Long	NA
Node-00096 - Lat	NA
Node-00097 - Long	NA
Node-00097 - Lat	NA
Node-00098 - Long	1.00147
Node-00098 - Lat	1.0013
Node-00099 - Long	1.180809
Node-00099 - Lat	1.277369
Node-00100 - Long	1.188857
Node-00100 - Lat	1.157715
Node-00101 - Long	NA
Node-00101 - Lat	NA
Node-00102 - Long	NA
Node-00102 - Lat	NA
Node-00103 - Long	NA
Node-00103 - Lat	NA
Node-00104 - Long	1.000239
Node-00104 - Lat	1.00045
Node-00105 - Long	1.000192
Node-00105 - Lat	1.000192
Node-00106 - Long	1.008707
Node-00106 - Lat	1.007982
Node-00107 - Long	1.007886
Node-00107 - Lat	1.007375
Node-00108 - Long	1.007621
Node-00108 - Lat	1.007522
Node-00109 - Long	NA
Node-00109 - Lat	NA
Node-00110 - Long	1.011326
Node-00110 - Lat	1.008453
Node-00111 - Long	NA
Node-00111 - Lat	NA
Node-00112 - Long	1.002627
Node-00112 - Lat	1.001139
Node-00113 - Long	1.000901
Node-00113 - Lat	1.000337
Node-00114 - Long	1.000492
Node-00114 - Lat	1.000004
Node-00115 - Long	NA
Node-00115 - Lat	NA
Node-00116 - Long	NA

Node-00116 - Lat	NA
Node-00117 - Long	1.000629
Node-00117 - Lat	1.000445
Node-00118 - Long	1.000004
Node-00118 - Lat	1.000029
Node-00119 - Long	NA
Node-00119 - Lat	NA
Node-00120 - Long	NA
Node-00120 - Lat	NA
Node-00121 - Long	1.002772
Node-00121 - Lat	1.003993
Node-00122 - Long	NA
Node-00122 - Lat	NA
Node-00123 - Long	NA
Node-00123 - Lat	NA
Node-00124 - Long	1.001722
Node-00124 - Lat	1.003355
Node-00125 - Long	1.000268
Node-00125 - Lat	1.000268
Node-00126 - Long	NA
Node-00126 - Lat	NA
Node-00127 - Long	1.290056
Node-00127 - Lat	1.292235
Node-00128 - Long	1.000075
Node-00128 - Lat	1.000075
Node-00129 - Long	1.290559
Node-00129 - Lat	1.290455
Node-00130 - Long	NA
Node-00130 - Lat	NA
Node-00131 - Long	NA
Node-00131 - Lat	NA
Node-00132 - Long	1.446911
Node-00132 - Lat	1.435575
Node-00133 - Long	NA
Node-00133 - Lat	NA
Node-00134 - Long	1.627078
Node-00134 - Lat	1.71138
Node-00135 - Long	NA
Node-00135 - Lat	NA
Node-00136 - Long	NA
Node-00136 - Lat	NA
Node-00137 - Long	1.292055
Node-00137 - Lat	1.332572
Node-00138 - Long	NA
Node-00138 - Lat	NA
Node-00139 - Long	NA
Node-00139 - Lat	NA
Node-00140 - Long	1.100764

Node-00140 - Lat	1.373999
Node-00141 - Long	NA
Node-00141 - Lat	NA
Node-00142 - Long	NA
Node-00142 - Lat	NA
Node-00143 - Long	1.028948
Node-00143 - Lat	1.060722
Node-00144 - Long	NA
Node-00144 - Lat	NA
Node-00145 - Long	NA
Node-00145 - Lat	NA
Node-00146 - Long	1.004504
Node-00146 - Lat	1.001264
Node-00147 - Long	1.003841
Node-00147 - Lat	1.001135
Node-00148 - Long	1.005531
Node-00148 - Lat	1.00131
Node-00149 - Long	NA
Node-00149 - Lat	NA
Node-00150 - Long	NA
Node-00150 - Lat	NA
Node-00151 - Long	NA
Node-00151 - Lat	NA
Node-00152 - Long	NA
Node-00152 - Lat	NA
Node-00153 - Long	NA
Node-00153 - Lat	NA
Node-00154 - Long	1.000507
Node-00154 - Lat	1.000323
Node-00155 - Long	NA
Node-00155 - Lat	NA
Node-00156 - Long	NA
Node-00156 - Lat	NA
Node-00157 - Long	1.000783
Node-00157 - Lat	1.000973
Node-00158 - Long	1.000885
Node-00158 - Lat	1.006034
Node-00159 - Long	NA
Node-00159 - Lat	NA
Node-00160 - Long	1.00118
Node-00160 - Lat	1.004963
Node-00161 - Long	1.002136
Node-00161 - Lat	1.00243
Node-00162 - Long	1.00225
Node-00162 - Lat	1.002567
Node-00163 - Long	1.00217
Node-00163 - Lat	1.002647
Node-00164 - Long	1.002839

Node-00164 - Lat	1.00289
Node-00165 - Long	1.003071
Node-00165 - Lat	1.003583
Node-00166 - Long	1.00745
Node-00166 - Lat	1.00328
Node-00167 - Long	1.008514
Node-00167 - Lat	1.00376
Node-00168 - Long	1.00959
Node-00168 - Lat	1.004488
Node-00169 - Long	1.01154
Node-00169 - Lat	1.005531
Node-00170 - Long	1.014704
Node-00170 - Lat	1.007373
Node-00171 - Long	1.003212
Node-00171 - Lat	1.002658
Node-00172 - Long	1.002863
Node-00172 - Lat	1.002522
Node-00173 - Long	1.001045
Node-00173 - Lat	1.000372
Node-00174 - Long	1.000292
Node-00174 - Lat	1.000276
Node-00175 - Long	NA
Node-00175 - Lat	NA
Node-00176 - Long	1.000274
Node-00176 - Lat	1.000984
Node-00177 - Long	NA
Node-00177 - Lat	NA
Node-00178 - Long	NA
Node-00178 - Lat	NA
Node-00179 - Long	1.000689
Node-00179 - Lat	1.00023
Node-00180 - Long	1.000599
Node-00180 - Lat	1.000153
Node-00181 - Long	NA
Node-00181 - Lat	NA
Node-00182 - Long	NA
Node-00182 - Lat	NA
Node-00183 - Long	NA
Node-00183 - Lat	NA
Node-00184 - Long	NA
Node-00184 - Lat	NA
Node-00185 - Long	NA
Node-00185 - Lat	NA
Node-00186 - Long	0.99998
Node-00186 - Lat	0.999997
Node-00187 - Long	NA
Node-00187 - Lat	NA
Node-00188 - Long	1.001211

Node-00188 - Lat	1.000289
Node-00189 - Long	1.000139
Node-00189 - Lat	1.000099
Node-00190 - Long	NA
Node-00190 - Lat	NA
Node-00191 - Long	NA
Node-00191 - Lat	NA
Node-00192 - Long	1.000249
Node-00192 - Lat	1.000404
Node-00193 - Long	1.000169
Node-00193 - Lat	1.000207
Node-00194 - Long	1.00002
Node-00194 - Lat	1.000156
Node-00195 - Long	1.000111
Node-00195 - Lat	1.000217
Node-00196 - Long	1.000769
Node-00196 - Lat	1.000569
Node-00197 - Long	NA
Node-00197 - Lat	NA
Node-00198 - Long	1.006662
Node-00198 - Lat	1.005474
Node-00199 - Long	NA
Node-00199 - Lat	NA
Node-00200 - Long	NA
Node-00200 - Lat	NA
Node-00201 - Long	1.00014
Node-00201 - Lat	1.000254
Node-00202 - Long	1.000002
Node-00202 - Lat	1.000002
Node-00203 - Long	NA
Node-00203 - Lat	NA
Node-00204 - Long	NA
Node-00204 - Lat	NA
Node-00205 - Long	1.000037
Node-00205 - Lat	1.000037
Node-00206 - Long	NA
Node-00206 - Lat	NA
Node-00207 - Long	NA
Node-00207 - Lat	NA
Node-00208 - Long	NA
Node-00208 - Lat	NA
Node-00209 - Long	NA
Node-00209 - Lat	NA
Node-00210 - Long	1.000025
Node-00210 - Lat	1.000025
Node-00211 - Long	NA
Node-00211 - Lat	NA
Node-00212 - Long	1.000498

Node-00212 - Lat	1.000511
Node-00213 - Long	1.000331
Node-00213 - Lat	1.000387
Node-00214 - Long	1.000283
Node-00214 - Lat	1.000348
Node-00215 - Long	1.000173
Node-00215 - Lat	1.000201
Node-00216 - Long	NA
Node-00216 - Lat	NA
Node-00217 - Long	NA
Node-00217 - Lat	NA
Node-00218 - Long	1.000196
Node-00218 - Lat	1.000277
Node-00219 - Long	NA
Node-00219 - Lat	NA
Node-00220 - Long	0.99998
Node-00220 - Lat	0.99998
Node-00221 - Long	NA
Node-00221 - Lat	NA
Node-00222 - Long	NA
Node-00222 - Lat	NA
Node-00223 - Long	NA
Node-00223 - Lat	NA
Node-00224 - Long	NA
Node-00224 - Lat	NA
Node-00225 - Long	1.002204
Node-00225 - Lat	1.00054
Node-00226 - Long	1.00202
Node-00226 - Lat	1.00051
Node-00227 - Long	1.001789
Node-00227 - Lat	1.000388
Node-00228 - Long	1.001002
Node-00228 - Lat	1.000548
Node-00229 - Long	1.000717
Node-00229 - Lat	1.000404
Node-00230 - Long	1.00075
Node-00230 - Lat	1.000319
Node-00231 - Long	1.000685
Node-00231 - Lat	1.000251
Node-00232 - Long	1.000619
Node-00232 - Lat	1.000166
Node-00233 - Long	1.000049
Node-00233 - Lat	1.00007
Node-00234 - Long	1.000035
Node-00234 - Lat	1.000039
Node-00235 - Long	1.000117
Node-00235 - Lat	1.000018
Node-00236 - Long	1.000116

Node-00236 - Lat	1.000093
Node-00237 - Long	1.000152
Node-00237 - Lat	1.000095
Node-00238 - Long	1.129616
Node-00238 - Lat	1.544019
Node-00239 - Long	1.755144
Node-00239 - Lat	1.622066
Node-00240 - Long	1.774598
Node-00240 - Lat	1.194223
Node-00241 - Long	NA
Node-00241 - Lat	NA
Node-00242 - Long	NA
Node-00242 - Lat	NA
Node-00243 - Long	NA
Node-00243 - Lat	NA
Node-00244 - Long	1.000092
Node-00244 - Lat	1.000009
Node-00245 - Long	NA
Node-00245 - Lat	NA
Node-00246 - Long	NA
Node-00246 - Lat	NA
Node-00247 - Long	1.060643
Node-00247 - Lat	1.060643
Node-00248 - Long	NA
Node-00248 - Lat	NA
Node-00249 - Long	1.000108
Node-00249 - Lat	1.000049
Node-00250 - Long	1.000045
Node-00250 - Lat	1.000082
Node-00251 - Long	NA
Node-00251 - Lat	NA
Node-00252 - Long	1.000035
Node-00252 - Lat	1.000035
Node-00253 - Long	1.000116
Node-00253 - Lat	1.000073
Node-00254 - Long	NA
Node-00254 - Lat	NA
Node-00255 - Long	0.999988
Node-00255 - Lat	1.000062
Node-00256 - Long	NA
Node-00256 - Lat	NA
Node-00257 - Long	0.999981
Node-00257 - Lat	0.999981
Node-00258 - Long	1.000846
Node-00258 - Lat	1.000141
Node-00259 - Long	NA
Node-00259 - Lat	NA
Node-00260 - Long	NA

Node-00260 - Lat	NA
Node-00261 - Long	NA
Node-00261 - Lat	NA
Node-00262 - Long	1.00024
Node-00262 - Lat	1.000172
Node-00263 - Long	NA
Node-00263 - Lat	NA
Node-00264 - Long	NA
Node-00264 - Lat	NA
Node-00265 - Long	NA
Node-00265 - Lat	NA
Node-00266 - Long	NA
Node-00266 - Lat	NA
Node-00267 - Long	NA
Node-00267 - Lat	NA
Node-00268 - Long	NA
Node-00268 - Lat	NA
Node-00269 - Long	NA
Node-00269 - Lat	NA
Node-00270 - Long	NA
Node-00270 - Lat	NA
Node-00271 - Long	1.001246
Node-00271 - Lat	1.001083
Node-00272 - Long	NA
Node-00272 - Lat	NA
Node-00273 - Long	NA
Node-00273 - Lat	NA
Node-00274 - Long	1.000572
Node-00274 - Lat	1.000117
Node-00275 - Long	1.000245
Node-00275 - Lat	0.99999
Node-00276 - Long	1.000073
Node-00276 - Lat	1.000118
Node-00277 - Long	1.000045
Node-00277 - Lat	1.000068
Node-00278 - Long	1.000043
Node-00278 - Lat	1.000039
Node-00279 - Long	1.000025
Node-00279 - Lat	1.000013
Node-00280 - Long	NA
Node-00280 - Lat	NA
Node-00281 - Long	0.999982
Node-00281 - Lat	0.999979
Node-00282 - Long	1.000016
Node-00282 - Lat	NA
Node-00283 - Long	1.000075
Node-00283 - Lat	1.000008
Node-00284 - Long	NA

Node-00284 - Lat	NA
Node-00285 - Long	0.999994
Node-00285 - Lat	1.000019
Node-00286 - Long	0.999983
Node-00286 - Lat	0.999983
Node-00287 - Long	NA
Node-00287 - Lat	NA
Node-00288 - Long	1.291045
Node-00288 - Lat	1.295647
Node-00289 - Long	1.000106
Node-00289 - Lat	0.999994
Node-00290 - Long	1.000052
Node-00290 - Lat	1.000009
Node-00291 - Long	1.000017
Node-00291 - Lat	1.000015
Node-00292 - Long	NA
Node-00292 - Lat	NA
Node-00293 - Long	1.394665
Node-00293 - Lat	1.548282
Node-00294 - Long	NA
Node-00294 - Lat	NA
Node-00295 - Long	1.684753
Node-00295 - Lat	1.770363
Node-00296 - Long	1.644878
Node-00296 - Lat	1.585547
Node-00297 - Long	NA
Node-00297 - Lat	NA
Node-00298 - Long	NA
Node-00298 - Lat	NA
Node-00299 - Long	NA
Node-00299 - Lat	NA
Node-00300 - Long	1.000033
Node-00300 - Lat	0.999997
Node-00301 - Long	1.00003
Node-00301 - Lat	1.000024
Node-00302 - Long	NA
Node-00302 - Lat	NA
Node-00303 - Long	1.000028
Node-00303 - Lat	1.000036
Node-00304 - Long	NA
Node-00304 - Lat	NA
Node-00305 - Long	NA
Node-00305 - Lat	NA
Node-00306 - Long	NA
Node-00306 - Lat	NA
Node-00307 - Long	NA
Node-00307 - Lat	NA
Node-00308 - Long	1.296138

Node-00308 - Lat	1.296851
Node-00309 - Long	NA
Node-00309 - Lat	NA
Node-00310 - Long	NA
Node-00310 - Lat	NA
Node-00311 - Long	1.000049
Node-00311 - Lat	1.000003
Node-00312 - Long	1.000209
Node-00312 - Lat	1.000034
Node-00313 - Long	1.000329
Node-00313 - Lat	1.000054
Node-00314 - Long	1.000616
Node-00314 - Lat	1.000052
Node-00315 - Long	1.000322
Node-00315 - Lat	1.000141
Node-00316 - Long	NA
Node-00316 - Lat	NA
Node-00317 - Long	NA
Node-00317 - Lat	NA
Node-00318 - Long	NA
Node-00318 - Lat	NA
Node-00319 - Long	NA
Node-00319 - Lat	NA
Node-00320 - Long	1.000051
Node-00320 - Lat	1.000024
Node-00321 - Long	NA
Node-00321 - Lat	NA
Node-00322 - Long	NA
Node-00322 - Lat	NA
Node-00323 - Long	NA
Node-00323 - Lat	NA
Node-00324 - Long	1.000294
Node-00324 - Lat	1.000424
Node-00325 - Long	NA
Node-00325 - Lat	NA
Node-00326 - Long	NA
Node-00326 - Lat	NA
Node-00327 - Long	1.00215
Node-00327 - Lat	1.000261
Node-00328 - Long	1.002459
Node-00328 - Lat	1.000217
Node-00329 - Long	1.000695
Node-00329 - Lat	1.000191
Node-00330 - Long	NA
Node-00330 - Lat	NA
Node-00331 - Long	NA
Node-00331 - Lat	NA
Node-00332 - Long	NA

Node-00332 - Lat	NA
Node-00333 - Long	NA
Node-00333 - Lat	NA
Node-00334 - Long	NA
Node-00334 - Lat	NA
Node-00335 - Long	1.001075
Node-00335 - Lat	1.000873
Node-00336 - Long	NA
Node-00336 - Lat	NA
Node-00337 - Long	1.000956
Node-00337 - Lat	1.00029
Node-00338 - Long	NA
Node-00338 - Lat	NA
Node-00339 - Long	NA
Node-00339 - Lat	NA
Node-00340 - Long	1.001581
Node-00340 - Lat	1.002832
Node-00341 - Long	1.00195
Node-00341 - Lat	1.003136
Node-00342 - Long	1.002377
Node-00342 - Lat	1.003907
Node-00343 - Long	1.001613
Node-00343 - Lat	1.002116
Node-00344 - Long	1.000981
Node-00344 - Lat	1.000315
Node-00345 - Long	1.003924
Node-00345 - Lat	1.000833
Node-00346 - Long	1.006674
Node-00346 - Lat	1.001342
Node-00347 - Long	1.003454
Node-00347 - Lat	1.00052
Node-00348 - Long	1.00186
Node-00348 - Lat	1.000361
Node-00349 - Long	1.000575
Node-00349 - Lat	1.000107
Node-00350 - Long	1.75305
Node-00350 - Lat	2.064018
Node-00351 - Long	1.00007
Node-00351 - Lat	1.000051
Node-00352 - Long	1.000044
Node-00352 - Lat	1.000217
Node-00353 - Long	1.000047
Node-00353 - Lat	1.000156
Node-00354 - Long	NA
Node-00354 - Lat	NA
Node-00355 - Long	1.00005
Node-00355 - Lat	1.00019
Node-00356 - Long	1.000007

Node-00356 - Lat	1.000123
Node-00357 - Long	1.000005
Node-00357 - Lat	1.000028
Node-00358 - Long	1.002801
Node-00358 - Lat	1.002801
Node-00359 - Long	NA
Node-00359 - Lat	NA
Node-00360 - Long	1
Node-00360 - Lat	1
Node-00361 - Long	NA
Node-00361 - Lat	NA
Node-00362 - Long	1.754654
Node-00362 - Lat	1.852904
Node-00363 - Long	1.700709
Node-00363 - Lat	2.066403
Node-00364 - Long	NA
Node-00364 - Lat	NA
Node-00365 - Long	NA
Node-00365 - Lat	NA
Node-00366 - Long	NA
Node-00366 - Lat	NA
Node-00367 - Long	NA
Node-00367 - Lat	NA
Node-00368 - Long	1.000515
Node-00368 - Lat	1.000183
Node-00369 - Long	NA
Node-00369 - Lat	NA
Node-00370 - Long	1.000021
Node-00370 - Lat	1.000207
Node-00371 - Long	NA
Node-00371 - Lat	NA
Node-00372 - Long	1.022407
Node-00372 - Lat	1.003743
Node-00373 - Long	1.066892
Node-00373 - Lat	1.010792
Node-00374 - Long	1.000734
Node-00374 - Lat	1.001059
Node-00375 - Long	1.000041
Node-00375 - Lat	1.000036
Node-00376 - Long	0.999975
Node-00376 - Lat	1.000013
Node-00377 - Long	1.653935
Node-00377 - Lat	1.013227
Node-00378 - Long	NA
Node-00378 - Lat	NA
Node-00379 - Long	6.166928
Node-00379 - Lat	1.326309
Node-00380 - Long	1.515992

Node-00380 - Lat	1.004623
Node-00381 - Long	1.223595
Node-00381 - Lat	1.002268
Node-00382 - Long	NA
Node-00382 - Lat	NA
Node-00383 - Long	1.074136
Node-00383 - Lat	1.005517
Node-00384 - Long	NA
Node-00384 - Lat	NA
Node-00385 - Long	5292642
Node-00385 - Lat	321268.9
Node-00386 - Long	6632471
Node-00386 - Lat	383396.4
Node-00387 - Long	Inf
Node-00387 - Lat	Inf
Node-00388 - Long	Inf
Node-00388 - Lat	Inf
Node-00389 - Long	3.57732
Node-00389 - Lat	1.710659
Node-00390 - Long	1.003923
Node-00390 - Lat	1.002882
Node-00391 - Long	NA
Node-00391 - Lat	NA
Node-00392 - Long	0.999992
Node-00392 - Lat	0.999974
Node-00393 - Long	1.000557
Node-00393 - Lat	1.000152
Node-00394 - Long	1.001279
Node-00394 - Lat	1.003021
Node-00395 - Long	1.000302
Node-00395 - Lat	1.001129
Node-00396 - Long	1.001567
Node-00396 - Lat	1.036508
Node-00397 - Long	NA
Node-00397 - Lat	NA
Node-00398 - Long	0.999996
Node-00398 - Lat	1.000208
Node-00399 - Long	NA
Node-00399 - Lat	NA
Node-00400 - Long	NA
Node-00400 - Lat	NA
Node-00401 - Long	NA
Node-00401 - Lat	NA
Node-00402 - Long	NA
Node-00402 - Lat	NA
Node-00403 - Long	1.001605
Node-00403 - Lat	1.003969
Node-00404 - Long	1.00377

Node-00404 - Lat	1.037711
Node-00405 - Long	1.006565
Node-00405 - Lat	1.000137
Node-00406 - Long	1.001599
Node-00406 - Lat	1.000021
Node-00407 - Long	NA
Node-00407 - Lat	NA
Node-00408 - Long	NA
Node-00408 - Lat	NA
Node-00409 - Long	NA
Node-00409 - Lat	NA
Node-00410 - Long	1.015034
Node-00410 - Lat	1.037142
Node-00411 - Long	NA
Node-00411 - Lat	NA
Node-00412 - Long	NA
Node-00412 - Lat	NA
Node-00413 - Long	NA
Node-00413 - Lat	NA
Node-00414 - Long	1.001666
Node-00414 - Lat	1.025777
Node-00415 - Long	1.001191
Node-00415 - Lat	1.022102
Node-00416 - Long	1.000958
Node-00416 - Lat	1.020113
Node-00417 - Long	1.000631
Node-00417 - Lat	1.019175
Node-00418 - Long	1.190697
Node-00418 - Lat	1.156406
Node-00419 - Long	1.000004
Node-00419 - Lat	1.000035
Node-00420 - Long	1.000239
Node-00420 - Lat	1.000161
Node-00421 - Long	1.000348
Node-00421 - Lat	1.000221
Node-00422 - Long	1.000215
Node-00422 - Lat	1.000238
Node-00423 - Long	1.000177
Node-00423 - Lat	1.000235
Node-00424 - Long	1.000053
Node-00424 - Lat	1.000173
Node-00425 - Long	NA
Node-00425 - Lat	NA
Node-00426 - Long	1.00011
Node-00426 - Lat	1.00015
Node-00427 - Long	NA
Node-00427 - Lat	NA
Node-00428 - Long	NA

Node-00428 - Lat	NA
Node-00429 - Long	1.000176
Node-00429 - Lat	1.000236
Node-00430 - Long	1.000022
Node-00430 - Lat	1.00181
Node-00431 - Long	1.00047
Node-00431 - Lat	1.000745
Node-00432 - Long	1.000155
Node-00432 - Lat	1.000359
Node-00433 - Long	NA
Node-00433 - Lat	NA
Node-00434 - Long	NA
Node-00434 - Lat	NA
Node-00435 - Long	NA
Node-00435 - Lat	NA
Node-00436 - Long	1.000018
Node-00436 - Lat	1.001626
Node-00437 - Long	0.999985
Node-00437 - Lat	1.001435
Node-00438 - Long	1.000092
Node-00438 - Lat	1.001576
Node-00439 - Long	1.000121
Node-00439 - Lat	1.001654
Node-00440 - Long	1.000079
Node-00440 - Lat	1.001604
Node-00441 - Long	1.000092
Node-00441 - Lat	1.001658
Node-00442 - Long	1.000394
Node-00442 - Lat	1.002746
Node-00443 - Long	1.000968
Node-00443 - Lat	1.004253
Node-00444 - Long	1.003748
Node-00444 - Lat	1.00698
Node-00445 - Long	1.00626
Node-00445 - Lat	1.009462
Node-00446 - Long	1.006304
Node-00446 - Lat	1.010751
Node-00447 - Long	1.006786
Node-00447 - Lat	1.012128
Node-00448 - Long	1.01485
Node-00448 - Lat	1.025
Node-00449 - Long	1.000365
Node-00449 - Lat	1.000728
Node-00450 - Long	NA
Node-00450 - Lat	NA
Node-00451 - Long	NA
Node-00451 - Lat	NA
Node-00452 - Long	1.000291

Node-00452 - Lat	1.001635
Node-00453 - Long	1.000525
Node-00453 - Lat	1.001565
Node-00454 - Long	NA
Node-00454 - Lat	NA
Node-00455 - Long	1.000514
Node-00455 - Lat	1.001857
Node-00456 - Long	NA
Node-00456 - Lat	NA
Node-00457 - Long	1.005305
Node-00457 - Lat	1.013608
Node-00458 - Long	1.009943
Node-00458 - Lat	1.01379
Node-00459 - Long	NA
Node-00459 - Lat	NA
Node-00460 - Long	1.00043
Node-00460 - Lat	1.001131
Node-00461 - Long	1.002437
Node-00461 - Lat	1.002437
Node-00462 - Long	NA
Node-00462 - Lat	NA
Node-00463 - Long	1.006495
Node-00463 - Lat	1.00231
Node-00464 - Long	NA
Node-00464 - Lat	NA
Node-00465 - Long	1.000081
Node-00465 - Lat	1.000144
Node-00466 - Long	1.000096
Node-00466 - Lat	1.00026
Node-00467 - Long	NA
Node-00467 - Lat	NA
Node-00468 - Long	1.000248
Node-00468 - Lat	1.000065
Node-00469 - Long	1.000211
Node-00469 - Lat	1.000168
Node-00470 - Long	NA
Node-00470 - Lat	NA
Node-00471 - Long	1.000231
Node-00471 - Lat	1.000231
Node-00472 - Long	NA
Node-00472 - Lat	NA
Node-00473 - Long	NA
Node-00473 - Lat	NA
Node-00474 - Long	1.005223
Node-00474 - Lat	1.005223
Node-00475 - Long	NA
Node-00475 - Lat	NA
Node-00476 - Long	1.000337

Node-00476 - Lat	1.001104
Node-00477 - Long	NA
Node-00477 - Lat	NA
Node-00478 - Long	NA
Node-00478 - Lat	NA
Node-00479 - Long	NA
Node-00479 - Lat	NA
Node-00480 - Long	NA
Node-00480 - Lat	NA
Node-00481 - Long	1.000115
Node-00481 - Lat	1.000365
Node-00482 - Long	NA
Node-00482 - Lat	NA
Node-00483 - Long	1.000039
Node-00483 - Lat	1.000039
Node-00484 - Long	1.000074
Node-00484 - Lat	1.000728
Node-00485 - Long	1.000066
Node-00485 - Lat	1.000254
Node-00486 - Long	NA
Node-00486 - Lat	NA
Node-00487 - Long	NA
Node-00487 - Lat	NA
Node-00488 - Long	1.000232
Node-00488 - Lat	1.000066
Node-00489 - Long	NA
Node-00489 - Lat	NA
Node-00490 - Long	NA
Node-00490 - Lat	NA
Node-00491 - Long	0.999998
Node-00491 - Lat	1.000712
Node-00492 - Long	0.999993
Node-00492 - Lat	1.000462
Node-00493 - Long	1.000021
Node-00493 - Lat	1.000011
Node-00494 - Long	NA
Node-00494 - Lat	NA
Node-00495 - Long	0.999996
Node-00495 - Lat	1.000007
Node-00496 - Long	0.999984
Node-00496 - Lat	1.000395
Node-00497 - Long	NA
Node-00497 - Lat	NA
Node-00498 - Long	1.000098
Node-00498 - Lat	1.000009
Node-00499 - Long	NA
Node-00499 - Lat	NA
Node-00500 - Long	NA

Node-00500 - Lat	NA
Node-00501 - Long	NA
Node-00501 - Lat	NA
Node-00502 - Long	NA
Node-00502 - Lat	NA
Node-00503 - Long	NA
Node-00503 - Lat	NA
Node-00504 - Long	0.99999
Node-00504 - Lat	0.99999
Node-00505 - Long	NA
Node-00505 - Lat	NA
Node-00506 - Long	NA
Node-00506 - Lat	NA
Node-00507 - Long	1.000179
Node-00507 - Lat	1.000134
Node-00508 - Long	NA
Node-00508 - Lat	NA
Node-00509 - Long	NA
Node-00509 - Lat	NA
Node-00510 - Long	1.161691
Node-00510 - Lat	1.240434
Node-00511 - Long	NA
Node-00511 - Lat	NA
Node-00512 - Long	NA
Node-00512 - Lat	NA
Node-00513 - Long	1.000431
Node-00513 - Lat	1.010318
Node-00514 - Long	1.000355
Node-00514 - Lat	1.00601
Node-00515 - Long	1.000156
Node-00515 - Lat	1.00001
Node-00516 - Long	1.000001
Node-00516 - Lat	0.999984
Node-00517 - Long	NA
Node-00517 - Lat	NA
Node-00518 - Long	NA
Node-00518 - Lat	NA
Node-00519 - Long	NA
Node-00519 - Lat	NA
Node-00520 - Long	NA
Node-00520 - Lat	NA
Node-00521 - Long	NA
Node-00521 - Lat	NA
Node-00522 - Long	NA
Node-00522 - Lat	NA
Node-00523 - Long	1.000069
Node-00523 - Lat	1.000215
Node-00524 - Long	1.000043

Node-00524 - Lat	1.000088
Node-00525 - Long	1.00017
Node-00525 - Lat	1.000005
Node-00526 - Long	1.000014
Node-00526 - Lat	0.999982
Node-00527 - Long	NA
Node-00527 - Lat	NA
Node-00528 - Long	1.000019
Node-00528 - Lat	1.000054
Node-00529 - Long	NA
Node-00529 - Lat	NA
Node-00530 - Long	NA
Node-00530 - Lat	NA
Node-00531 - Long	0.999996
Node-00531 - Lat	1.000008
Node-00532 - Long	NA
Node-00532 - Lat	NA
Node-00533 - Long	NA
Node-00533 - Lat	NA
Node-00534 - Long	1.000132
Node-00534 - Lat	1.002339
Node-00535 - Long	NA
Node-00535 - Lat	NA
Node-00536 - Long	1.000037
Node-00536 - Lat	1.00015
Node-00537 - Long	NA
Node-00537 - Lat	NA
Node-00538 - Long	NA
Node-00538 - Lat	NA
Node-00539 - Long	NA
Node-00539 - Lat	NA
Node-00540 - Long	1.000375
Node-00540 - Lat	1.000785
Node-00541 - Long	NA
Node-00541 - Lat	NA
Node-00542 - Long	NA
Node-00542 - Lat	NA
Node-00543 - Long	1.00083
Node-00543 - Lat	1.000523
Node-00544 - Long	1.000704
Node-00544 - Lat	1.000174
Node-00545 - Long	1.002859
Node-00545 - Lat	1.001207
Node-00546 - Long	1.000127
Node-00546 - Lat	1.000009
Node-00547 - Long	1.000056
Node-00547 - Lat	1.000062
Node-00548 - Long	1.000031

Node-00548 - Lat	1.000056
Node-00549 - Long	1.282201
Node-00549 - Lat	1.286405
Node-00550 - Long	1.28717
Node-00550 - Lat	1.288932
Node-00551 - Long	NA
Node-00551 - Lat	NA
Node-00552 - Long	NA
Node-00552 - Lat	NA
Node-00553 - Long	1.004048
Node-00553 - Lat	1.000205
Node-00554 - Long	1.006334
Node-00554 - Lat	1.00132
Node-00555 - Long	1.008238
Node-00555 - Lat	1.00311
Node-00556 - Long	1.011607
Node-00556 - Lat	1.007968
Node-00557 - Long	1.029104
Node-00557 - Lat	1.03697
Node-00558 - Long	16.31864
Node-00558 - Lat	10.65357
Node-00559 - Long	NA
Node-00559 - Lat	NA
Node-00560 - Long	Inf
Node-00560 - Lat	Inf
Node-00561 - Long	NA
Node-00561 - Lat	NA
Node-00562 - Long	1.007723
Node-00562 - Lat	1.005268
Node-00563 - Long	1.005129
Node-00563 - Lat	1.003342
Node-00564 - Long	1.004793
Node-00564 - Lat	1.002931
Node-00565 - Long	1.001765
Node-00565 - Lat	1.000076
Node-00566 - Long	1.001124
Node-00566 - Lat	1.000014
Node-00567 - Long	1.000938
Node-00567 - Lat	1.000008
Node-00568 - Long	NA
Node-00568 - Lat	NA
Node-00569 - Long	0.999995
Node-00569 - Lat	1.000013
Node-00570 - Long	NA
Node-00570 - Lat	NA
Node-00571 - Long	1.000102
Node-00571 - Lat	1.000116
Node-00572 - Long	NA

Node-00572 - Lat	NA
Node-00573 - Long	NA
Node-00573 - Lat	NA
Node-00574 - Long	1.000109
Node-00574 - Lat	1.000109
Node-00575 - Long	0.999987
Node-00575 - Lat	1.00001
Node-00576 - Long	NA
Node-00576 - Lat	NA
Node-00577 - Long	1.001836
Node-00577 - Lat	1.000453
Node-00578 - Long	NA
Node-00578 - Lat	NA
Node-00579 - Long	NA
Node-00579 - Lat	NA
Node-00580 - Long	0.999981
Node-00580 - Lat	0.999981
Node-00581 - Long	NA
Node-00581 - Lat	NA
Node-00582 - Long	1.018857
Node-00582 - Lat	1.035748
Node-00583 - Long	1.000034
Node-00583 - Lat	1.000194
Node-00584 - Long	NA
Node-00584 - Lat	NA
Node-00585 - Long	1.000081
Node-00585 - Lat	1.000116
Node-00586 - Long	1.000083
Node-00586 - Lat	1.000122
Node-00587 - Long	0.999991
Node-00587 - Lat	0.999983
Node-00588 - Long	0.99998
Node-00588 - Lat	1.000004
Node-00589 - Long	NA
Node-00589 - Lat	NA
Node-00590 - Long	NA
Node-00590 - Lat	NA
Node-00591 - Long	NA
Node-00591 - Lat	NA
Node-00592 - Long	1.000137
Node-00592 - Lat	1.0001
Node-00593 - Long	NA
Node-00593 - Lat	NA
Node-00594 - Long	1.000054
Node-00594 - Lat	1.000004
Node-00595 - Long	1.011696
Node-00595 - Lat	1.01764
Node-00596 - Long	1.059326

Node-00596 - Lat	1.003269
Node-00597 - Long	NA
Node-00597 - Lat	NA
Node-00598 - Long	1.000081
Node-00598 - Lat	1.000122
Node-00599 - Long	1.00009
Node-00599 - Lat	0.999989
Node-00600 - Long	1.000081
Node-00600 - Lat	1.000051
Node-00601 - Long	1.000059
Node-00601 - Lat	1.000061
Node-00602 - Long	NA
Node-00602 - Lat	NA
Node-00603 - Long	NA
Node-00603 - Lat	NA
Node-00604 - Long	NA
Node-00604 - Lat	NA
Node-00605 - Long	NA
Node-00605 - Lat	NA
Node-00606 - Long	NA
Node-00606 - Lat	NA
Node-00607 - Long	1.01266
Node-00607 - Lat	1.019645
Node-00608 - Long	1.001431
Node-00608 - Lat	1.002982
Node-00609 - Long	1.000629
Node-00609 - Lat	1.001299
Node-00610 - Long	NA
Node-00610 - Lat	NA
Node-00611 - Long	1.000245
Node-00611 - Lat	1.000286
Node-00612 - Long	NA
Node-00612 - Lat	NA
Node-00613 - Long	NA
Node-00613 - Lat	NA
Node-00614 - Long	NA
Node-00614 - Lat	NA
Node-00615 - Long	1.002496
Node-00615 - Lat	1.027953
Node-00616 - Long	NA
Node-00616 - Lat	NA
Node-00617 - Long	NA
Node-00617 - Lat	NA
Node-00618 - Long	1.011595
Node-00618 - Lat	1.004142
Node-00619 - Long	1.015741
Node-00619 - Lat	1.004744
Node-00620 - Long	1.014838

Node-00620 - Lat	1.004313
Node-00621 - Long	1.013332
Node-00621 - Lat	1.004355
Node-00622 - Long	NA
Node-00622 - Lat	NA
Node-00623 - Long	NA
Node-00623 - Lat	NA
Node-00624 - Long	0.999994
Node-00624 - Lat	0.999987
Node-00625 - Long	0.999979
Node-00625 - Lat	0.999959
Node-00626 - Long	1.03124
Node-00626 - Lat	1.030958
Node-00627 - Long	1.023629
Node-00627 - Lat	1.00679
Node-00628 - Long	NA
Node-00628 - Lat	NA
Node-00629 - Long	NA
Node-00629 - Lat	NA
Node-00630 - Long	NA
Node-00630 - Lat	NA
Node-00631 - Long	NA
Node-00631 - Lat	NA
Node-00632 - Long	1.00524
Node-00632 - Lat	1.027217
Node-00633 - Long	NA
Node-00633 - Lat	NA
Node-00634 - Long	NA
Node-00634 - Lat	NA
Node-00635 - Long	NA
Node-00635 - Lat	NA
Node-00636 - Long	NA
Node-00636 - Lat	NA
Node-00637 - Long	1.002827
Node-00637 - Lat	1.004344
Node-00638 - Long	1.000035
Node-00638 - Lat	1.000035
Node-00639 - Long	1.000803
Node-00639 - Lat	1.006152
Node-00640 - Long	NA
Node-00640 - Lat	NA
Node-00641 - Long	1.000201
Node-00641 - Lat	1.000209
Node-00642 - Long	1.004057
Node-00642 - Lat	1.009817
Node-00643 - Long	1.000053
Node-00643 - Lat	1.000073
Node-00644 - Long	1.000947

Node-00644 - Lat	1.002126
Node-00645 - Long	NA
Node-00645 - Lat	NA
Node-00646 - Long	NA
Node-00646 - Lat	NA
Node-00647 - Long	NA
Node-00647 - Lat	NA
Node-00648 - Long	0.999974
Node-00648 - Lat	0.999974
Node-00649 - Long	NA
Node-00649 - Lat	NA
Node-00650 - Long	1.000815
Node-00650 - Lat	1.00153
Node-00651 - Long	NA
Node-00651 - Lat	NA
Node-00652 - Long	1.000526
Node-00652 - Lat	1.000513
Node-00653 - Long	NA
Node-00653 - Lat	NA
Node-00654 - Long	1.022702
Node-00654 - Lat	1.090698
Node-00655 - Long	1.001407
Node-00655 - Lat	1.000312
Node-00656 - Long	1.000378
Node-00656 - Lat	1.000767
Node-00657 - Long	1.000274
Node-00657 - Lat	1.003367
Node-00658 - Long	1.006026
Node-00658 - Lat	1.03358
Node-00659 - Long	1.005192
Node-00659 - Lat	1.033561
Node-00660 - Long	1.002615
Node-00660 - Lat	1.01418
Node-00661 - Long	NA
Node-00661 - Lat	NA
Node-00662 - Long	1.009126
Node-00662 - Lat	1.007442
Node-00663 - Long	1.004642
Node-00663 - Lat	1.001997
Node-00664 - Long	NA
Node-00664 - Lat	NA
Node-00665 - Long	NA
Node-00665 - Lat	NA
Node-00666 - Long	NA
Node-00666 - Lat	NA
Node-00667 - Long	NA
Node-00667 - Lat	NA
Node-00668 - Long	1.291154

Node-00668 - Lat	1.290927
Node-00669 - Long	1.290946
Node-00669 - Lat	1.290786
Node-00670 - Long	NA
Node-00670 - Lat	NA
Node-00671 - Long	NA
Node-00671 - Lat	NA
Node-00672 - Long	1.000075
Node-00672 - Lat	1.00066
Node-00673 - Long	NA
Node-00673 - Lat	NA
Node-00674 - Long	1.000374
Node-00674 - Lat	1.00256
Node-00675 - Long	1.000512
Node-00675 - Lat	1.003143
Node-00676 - Long	1.000046
Node-00676 - Lat	1.000429
Node-00677 - Long	1.000009
Node-00677 - Lat	1.000116
Node-00678 - Long	1.000002
Node-00678 - Lat	1.000072
Node-00679 - Long	0.999975
Node-00679 - Lat	0.999975
Node-00680 - Long	NA
Node-00680 - Lat	NA
Node-00681 - Long	1.000098
Node-00681 - Lat	1.000414
Node-00682 - Long	NA
Node-00682 - Lat	NA
Node-00683 - Long	NA
Node-00683 - Lat	NA
Node-00684 - Long	NA
Node-00684 - Lat	NA
Node-00685 - Long	NA
Node-00685 - Lat	NA
Node-00686 - Long	NA
Node-00686 - Lat	NA
Node-00687 - Long	NA
Node-00687 - Lat	NA
Node-00688 - Long	NA
Node-00688 - Lat	NA
Node-00689 - Long	1.04074
Node-00689 - Lat	1.091487
Node-00690 - Long	NA
Node-00690 - Lat	NA
Node-00691 - Long	NA
Node-00691 - Lat	NA
Node-00692 - Long	1.002213

Node-00692 - Lat	1.003144
Node-00693 - Long	1.003543
Node-00693 - Lat	1.001233
Node-00694 - Long	NA
Node-00694 - Lat	NA
Node-00695 - Long	1.007984
Node-00695 - Lat	1.004733
Node-00696 - Long	1574.101
Node-00696 - Lat	1036.884
Node-00697 - Long	3404.016
Node-00697 - Lat	1878.68
Node-00698 - Long	Inf
Node-00698 - Lat	Inf
Node-00699 - Long	Inf
Node-00699 - Lat	Inf
Node-00700 - Long	NA
Node-00700 - Lat	NA
Node-00701 - Long	NA
Node-00701 - Lat	NA
Node-00702 - Long	1.00003
Node-00702 - Lat	1.000009
Node-00703 - Long	1.00007
Node-00703 - Lat	1.000024
Node-00704 - Long	1.030518
Node-00704 - Lat	1.030518
Node-00705 - Long	NA
Node-00705 - Lat	NA
Node-00706 - Long	NA
Node-00706 - Lat	NA
Node-00707 - Long	1.000943
Node-00707 - Lat	1.001945
Node-00708 - Long	1.000546
Node-00708 - Lat	1.00091
Node-00709 - Long	1.000243
Node-00709 - Lat	1.000301
Node-00710 - Long	1.000198
Node-00710 - Lat	1.000265
Node-00711 - Long	1.000171
Node-00711 - Lat	1.000505
Node-00712 - Long	1.00046
Node-00712 - Lat	1.001098
Node-00713 - Long	1.000025
Node-00713 - Lat	1.000016
Node-00714 - Long	1.000364
Node-00714 - Lat	1.001022
Node-00715 - Long	1.000398
Node-00715 - Lat	1.000367
Node-00716 - Long	1.004635

Node-00716 - Lat	1.00268
Node-00717 - Long	1.005522
Node-00717 - Lat	1.003423
Node-00718 - Long	1.000172
Node-00718 - Lat	1.000175
Node-00719 - Long	NA
Node-00719 - Lat	NA
Node-00720 - Long	1.000134
Node-00720 - Lat	1.000121
Node-00721 - Long	0.99999
Node-00721 - Lat	0.99999
Node-00722 - Long	NA
Node-00722 - Lat	NA
Node-00723 - Long	1.000153
Node-00723 - Lat	1.000036
Node-00724 - Long	1.00001
Node-00724 - Lat	1.00001
Node-00725 - Long	NA
Node-00725 - Lat	NA
Node-00726 - Long	1.000458
Node-00726 - Lat	1.001104
Node-00727 - Long	1.000079
Node-00727 - Lat	1.000488
Node-00728 - Long	NA
Node-00728 - Lat	NA
Node-00729 - Long	NA
Node-00729 - Lat	NA
Node-00730 - Long	1.000516
Node-00730 - Lat	1.001178
Node-00731 - Long	NA
Node-00731 - Lat	NA
Node-00732 - Long	NA
Node-00732 - Lat	NA
Node-00733 - Long	1.007205
Node-00733 - Lat	1.000072
Node-00734 - Long	NA
Node-00734 - Lat	NA
Node-00735 - Long	0.999991
Node-00735 - Lat	1.000006
Node-00736 - Long	NA
Node-00736 - Lat	NA
Node-00737 - Long	1.000163
Node-00737 - Lat	1.000091
Node-00738 - Long	NA
Node-00738 - Lat	NA
Node-00739 - Long	NA
Node-00739 - Lat	NA
Node-00740 - Long	NA

Node-00740 - Lat	NA
Node-00741 - Long	1.000875
Node-00741 - Lat	1.001479
Node-00742 - Long	1.000026
Node-00742 - Lat	1.00014
Node-00743 - Long	NA
Node-00743 - Lat	NA
Node-00744 - Long	1.00001
Node-00744 - Lat	1.000008
Node-00745 - Long	NA
Node-00745 - Lat	NA
Node-00746 - Long	1.212064
Node-00746 - Lat	1.224691
Node-00747 - Long	1.260062
Node-00747 - Lat	1.215935
Node-00748 - Long	NA
Node-00748 - Lat	NA
Node-00749 - Long	NA
Node-00749 - Lat	NA
Node-00750 - Long	NA
Node-00750 - Lat	NA
Node-00751 - Long	1.000941
Node-00751 - Lat	1.006614
Node-00752 - Long	NA
Node-00752 - Lat	NA
Node-00753 - Long	1.000746
Node-00753 - Lat	1.001319
Node-00754 - Long	1.000036
Node-00754 - Lat	1.000036
Node-00755 - Long	1.000331
Node-00755 - Lat	1.000791
