# Supplementary material for: Landscape-explicit phylogeography illuminates the ecographic radiation of early archosauromorph reptiles
Source: Nat Ecol Evol. 2025 Jun 11;9(7):1138–52. doi: 10.1038/s41559-025-02739-y (PMC12240803; doi:10.1038/s41559-025-02739-y)
Supplement: Supplementary file 2 — Reporting Summary [file 41559_2025_2739_MOESM2_ESM.pdf]

nature portfolio

Corresponding author(s): Dr Joseph Flannery Sutherland

Last updated by author(s): Feb 5, 2025

Reporting Summary

Nature Portfolio wishes to improve the reproducibility of the work that we publish. This form provides structure for consistency and transparency in reporting. For further information on Nature Portfolio policies, see our Editorial Policies and the Editorial Policy Checklist.

Statistics

For all statistical analyses, confirm that the following items are present in the figure legend, table legend, main text, or Methods section.

n/a	Confirmed	
☐	☒	The exact sample size (*n*) for each experimental group/condition, given as a discrete number and unit of measurement
☐	☒	A statement on whether measurements were taken from distinct samples or whether the same sample was measured repeatedly
☐	☒	The statistical test(s) used AND whether they are one- or two-sided<br>*Only common tests should be described solely by name; describe more complex techniques in the Methods section.*
☒	☐	A description of all covariates tested
☒	☐	A description of any assumptions or corrections, such as tests of normality and adjustment for multiple comparisons
☐	☒	A full description of the statistical parameters including central tendency (e.g. means) or other basic estimates (e.g. regression coefficient) AND variation (e.g. standard deviation) or associated estimates of uncertainty (e.g. confidence intervals)
☒	☐	For null hypothesis testing, the test statistic (e.g. *F*, *t*, *r*) with confidence intervals, effect sizes, degrees of freedom and *P* value noted<br>*Give P values as exact values whenever suitable.*
☐	☒	For Bayesian analysis, information on the choice of priors and Markov chain Monte Carlo settings
☒	☐	For hierarchical and complex designs, identification of the appropriate level for tests and full reporting of outcomes
☐	☒	Estimates of effect sizes (e.g. Cohen's *d*, Pearson's *r*), indicating how they were calculated

Our web collection on statistics for biologists contains articles on many of the points above.

Software and code

Policy information about availability of computer code

Data collection	Occurrence data were collated using the Paleobiology Database API via R scripts. Climate data was acquired and processed using R scripts from the University of Bristol climate simulation repository.
Data analysis	Two sets of analyses were conducted in MrBayes (tree timescaling; v.3.3.7) and BayesTraits (geo model estimation; v.4.0). All other analyses were conducted in R using a mixture of standard packages and custom functions. All code for all analyses is provided in the supplement

For manuscripts utilizing custom algorithms or software that are central to the research but not yet described in published literature, software must be made available to editors and reviewers. We strongly encourage code deposition in a community repository (e.g. GitHub). See the Nature Portfolio guidelines for submitting code & software for further information.

Data

Policy information about availability of data

All manuscripts must include a data availability statement. This statement should provide the following information, where applicable:

- Accession codes, unique identifiers, or web links for publicly available datasets
- A description of any restrictions on data availability
- For clinical datasets or third party data, please ensure that the statement adheres to our policy

All data is available in the repository link in the Data Availability statement in the manuscript.

Research involving human participants, their data, or biological material

Policy information about studies with human participants or human data. See also policy information about sex, gender (identity/presentation), and sexual orientation and race, ethnicity and racism.

Reporting on sex and gender	NA
Reporting on race, ethnicity, or other socially relevant groupings	NA
Population characteristics	NA
Recruitment	NA
Ethics oversight	NA

Note that full information on the approval of the study protocol must also be provided in the manuscript.

Field-specific reporting

Please select the one below that is the best fit for your research. If you are not sure, read the appropriate sections before making your selection.

☐ Life sciences ☐ Behavioural & social sciences ☒ Ecological, evolutionary & environmental sciences

For a reference copy of the document with all sections, see nature.com/documents/nr-reporting-summary-flat.pdf

Ecological, evolutionary & environmental sciences study design

All studies must disclose on these points even when the disclosure is negative.

Study description	Geographic and climatic niche ancestral estimation for early archosauromorph reptiles using a novel landscape connectivity approach
Research sample	A time-calibrated phylogeny and fossil occurrence data for 392 early archosauromorph taxa, ranging primarily from the Middle Permian to the Late Triassic. Climate simulations and topographic data for the same interval
Sampling strategy	All fossil occurrences were downloaded, then filtered in R
Data collection	Occurrences downloaded via the Palaeobiology Database using R
Timing and spatial scale	Fossil occurrence data ranging primarily from the Middle Permian to the Late Triassic, global scale dataset
Data exclusions	Only a few occurrences were excluded as taxonomically suspect. All exclusions are fully documented in the ESM
Reproducibility	Every single analysis and data processing protocol is provided in the ESM using R, bash, MrBayes and BayesTraits scripts
Randomization	Not relevant
Blinding	Not relevant

Did the study involve field work? ☐ Yes ☒ No

Reporting for specific materials, systems and methods

We require information from authors about some types of materials, experimental systems and methods used in many studies. Here, indicate whether each material, system or method listed is relevant to your study. If you are not sure if a list item applies to your research, read the appropriate section before selecting a response.

Materials & experimental systems

n/a	Involved in the study
☒	☐ Antibodies
☒	☐ Eukaryotic cell lines
☒	☐ Palaeontology and archaeology
☒	☐ Animals and other organisms
☒	☐ Clinical data
☒	☐ Dual use research of concern
☒	☐ Plants

Methods

n/a	Involved in the study
☒	☐ ChIP-seq
☒	☐ Flow cytometry
☒	☐ MRI-based neuroimaging

Plants

Seed stocks	NA
Novel plant genotypes	NA
Authentication	NA
